# Metabolomics and transcriptomics based multi-omics integration reveals radiation-induced altered pathway networking and underlying mechanism

**DOI:** 10.1038/s41540-023-00305-5

**Published:** 2023-09-09

**Authors:** Kiran Maan, Ruchi Baghel, Seema Dhariwal, Apoorva Sharma, Radhika Bakhshi, Poonam Rana

**Affiliations:** 1 Metabolomics Research Facility, Institute of Nuclear Medicine and Allied Sciences (INMAS), DRDO, Delhi, India; 2 Department of Biomedical Science, Shaheed Rajguru College of Applied Sciences for Women, University of Delhi, Delhi, India

**Keywords:** Biomarkers, Biochemical networks

## Abstract

Recent advancement in integrated multi-omics has significantly contributed to many areas of the biomedical field. Radiation research has also grasped uprising omics technologies in biomarker identification to aid in triage management. Herein, we have used a combinatorial multi-omics approach based on transcriptomics together with metabolomics and lipidomics of blood from murine exposed to 1 Gy (LD) and 7.5 Gy (HD) of total-body irradiation (TBI) for a comprehensive understanding of biological processes through integrated pathways and networking. Both omics displayed demarcation of HD group from controls using multivariate analysis. Dysregulated amino acids, various PC, PE and carnitine were observed along with many dysregulated genes (Nos2, Hmgcs2, Oxct2a, etc.). Joint-Pathway Analysis and STITCH interaction showed radiation exposure resulted in changes in amino acid, carbohydrate, lipid, nucleotide, and fatty acid metabolism. Elicited immune response was also observed by Gene Ontology. BioPAN has predicted Elovl5, Elovl6 and Fads2 for fatty acid pathways, only in HD group. Collectively, the combined omics approach facilitated a better understanding of processes uncovering metabolic pathways. Presumably, this is the first in radiation metabolomics that utilized an integrated omics approach following TBI in mice. Our work showed that omics integration could be a valuable tool for better comprehending the mechanism as well as molecular interactions.

## Introduction

The last decade has shown remarkable research progress towards the development of high throughput biomarkers. Technical advancements, especially in analytical tools and bioinformatics have made remarkable development not only in biomarker research but also in comprehending deep insight into pathophysiological disturbances associated with diseased state. Radiation research has also explored all new dimensions of omics for the development of high throughput biomarkers for radiation triage. Recently published series of studies are direct evidence of the role of a metabolomics-based panel of metabolites as radiation biomarkers for radiation exposure^1–3^. Further, there is an urgent need for the identification of universal biomarkers for total or partial body irradiation^4^. However, uncertainty over the specificity of metabolomic biomarkers for radiation (dose and severity) still looms around. This ambiguity in biomarker identification reinforces the belief that there isn’t a single, ideal technique of evaluation that can account for all dosages and levels of exposure. Therefore, complementary and surrogate approaches are the need of the hour in radiation dose assessment studies.

Multi-omics approach could provide a solution through identified and validated biomarkers to some of the complex scenarios like radiation exposure and help in triage management. In line with this, recent literature also supports the applicability of transcriptomics and metabolomics in biomarker identification and pathology elucidation. Transcriptomics is a powerful method for determining the function of important genes and comparing gene expression under various stress circumstances by carrying out qualitative or quantitative analysis of mRNA using high-throughput microarray analysis^5^. On the other hand, metabolomics is described as the study of changes in endogenous metabolites in biological systems (biofluids/cells/tissues) in response to any kind of stressor, whereas lipidomics is a sub-branch of metabolomics.

Although metabolic profiling is an effective approach, metabolites/lipid changes may not accurately identify pathways altered by radiation exposure. This gap can be addressed by integrating omics technologies which will offer a more comprehensive view of alterations in complex biological processes induced by any stress. The combined metabolomics and transcriptomics approach will generate extensive data addressing the inter-related metabolic and transcriptomic changes. Further, this data integration will help in identifying the associations between enzymes/proteins and metabolites, uncovering molecular mechanisms based on high throughput data.

The integrated-omics approach will allow simultaneous analysis of mRNAs and metabolites providing whole transcriptome and metabolic changes after radiation exposure which would help in mapping complex relationships^6^. Recently, Meeks et al. have used transcriptomic and metabolomic data to assess radiation-induced damage in parotid salivary glands on the fifth day following 5 Gy targeted exposure to head and neck cancer. Their findings supported the notion of altered energy metabolism as a radiation-induced response^7^. Another study on HBE cells indicated that integration of metabolomic and transcriptomic dataset has shown that p53 regulates various genes that are associated with nitrogen, glutathione, arachidonic acid metabolism and also with glycolysis or gluconeogenesis in response to ionizing radiation (IR), showing potential targets of p53^8^.

A recent study on obese breast cancer revealed 7 novel enriched pathways compared to non-obese breast cancer patients after omics integration^9^. Similarly, in another study as well, integrative analysis has helped in confirming the correlation of metabolic changes with immunoregulation along with mitochondrial translation in active tuberculosis children patients and has also substantiated the association of various inflammatory pathways and has thus improved the elucidation of biomarkers^10^. The metabolome and transcriptome together offered credible information on pathological processes and the related metabolic pathways along with possible indicators and targets involved in particular stress conditions, laying the basis for further research on the mechanistic explanation^9–11^. To the best of our knowledge, integrated metabolomic and transcriptomic analysis has not been explored so far in total-body irradiation.

We have previously reported comprehensive metabolic profiling in urine using Nuclear Magnetic Resonance (NMR) spectroscopy and Liquid chromatography-mass spectrometry (LC-MS) distinguishing TBI (total-body irradiation) from PBI (partial-body irradiation) as well as sub-lethal (5 Gy) from lethal (7.5 Gy) doses of IR^3,12^. In the present study, we have used a combinatorial multi-omics approach based on transcriptomics analysis along with mass spectrometry-based metabolomics and lipidomics of plasma samples from rodent models exposed to total-body radiation. This has enabled an improved understanding of biological processes in their entirety. The advancement of omics approaches has greatly aided in the identification of critical players and their management in biological and pathological conditions, which is critical for the development of new therapeutic agents. Our study thus not only provides a comprehensive understanding of biological processes after radiation exposure but also enhances the validation of potential diagnostic biomarkers of radiation exposure.

## Results

**Fig. 1:**
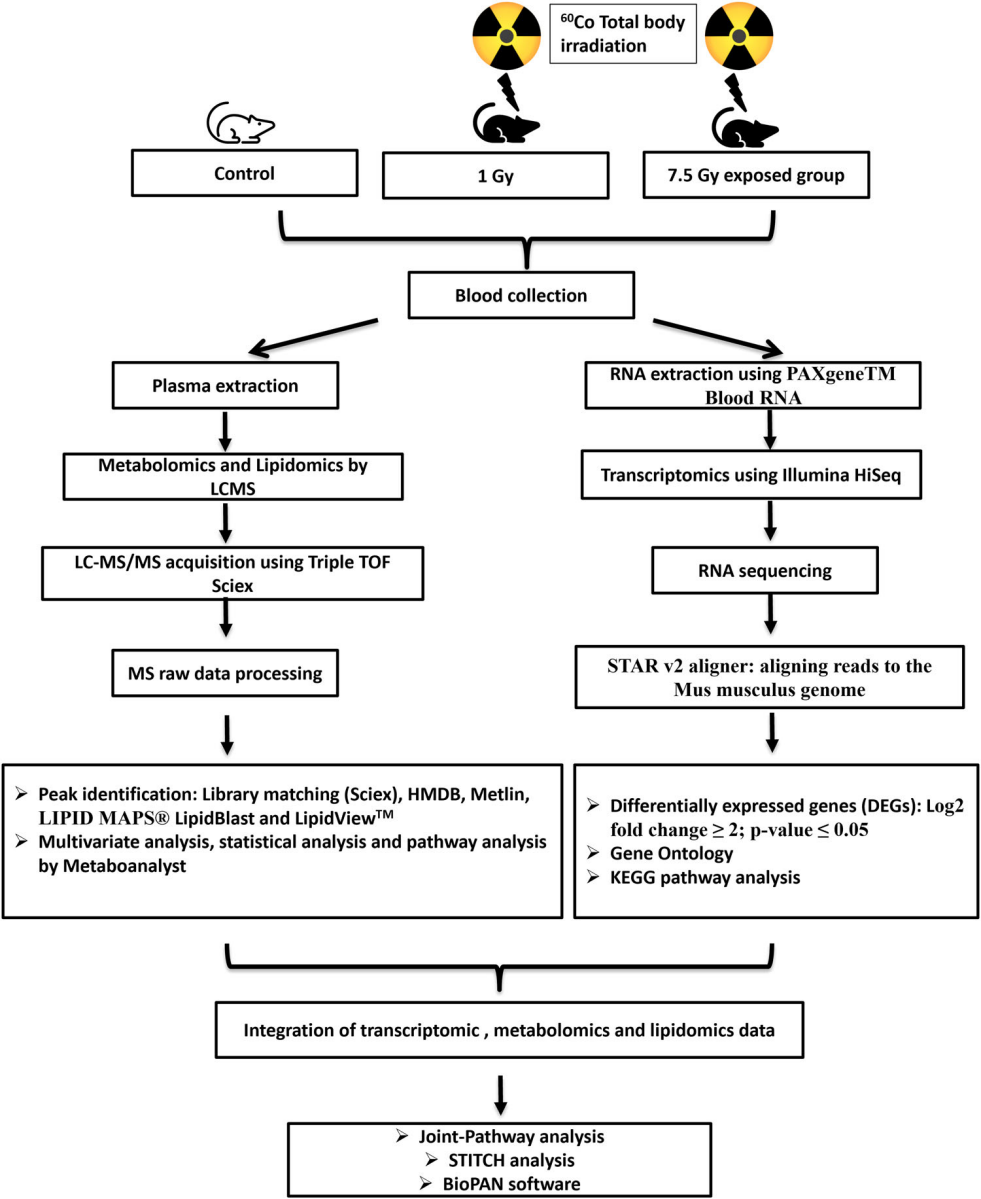
Workflow of metabolomics and transcriptomics in mice exposed to different radiation doses. Animals were exposed to 1 Gy (LD) and 7.5 Gy (HD) of total-body gamma radiation using ^60^Co source. Collected blood sample was subjected to metabolomics/lipidomics and transcriptomics analyses. Statistical analysis was performed followed by their pathway integration.

The present study was carried out for understanding the impact of ionizing radiation on gene expression (mRNA diversity) and metabolic profile in mice blood after 1 Gy and 7.5 Gy radiation exposure at 24 hours. The workflow used in the present study is represented in Fig. 1.

### Transcriptomic profiling

RNA sequencing was performed on RNA samples that passed the quality control (QC) indices. QC status and mean quality value across each base position can be observed from Supplementary Table 1 and Supplementary Fig. 1 respectively. The raw reads were mapped to 52636 mouse Ensembl genes IDs. However, ~23500 uniquely mapped genes were identified post-alignment to the mus musculus genome (9GRCm38.90). The principal component analysis (PCA) score plot showed a clear separation of High Dose (HD; 7.5 Gy) group from control, however, overlapping of Low dose (LD; 1 Gy) group with control was quite visible (Supplementary Fig. 2). After normalization, a total of 19668 genes were taken for differential gene expression analysis that resulted in the dysregulation of 2837 (1595 upregulated and 1242 downregulated) and 143 (67 upregulated and 76 downregulated) genes in HD and LD irradiated groups, respectively. Supplementary Table 2A and 2B lists the differentially expressed genes with log2 fold change ≥2 and adj. *p*-value ≤ 0.05 in HD and LD groups post irradiation compared to the control group.

#### Gene Ontology and KEGG pathway enrichment of transcriptomic data

Gene Ontology (GO)-based enrichment analysis mainly showed perturbation in pathways associated with immune response, cell adhesion, and receptor activity and were also found to be more prominent in HD group (41 in biological process (BP); 11 in cellular component (CC); 10 in molecular function (MF) pathways) than LD group (18 in BP; 4 in CC pathways) when compared to control (Supplementary Table 3A and Supplementary Table 3B).

Among BP in GO, “immunoglobulin production” showed the highest significance in HD group. Among the various differentially expressed genes (DEGs) involved in this pathway, H2-Ab1 (histocompatibility 2, class II antigen A, beta 1), Lax1 (lymphocyte transmembrane adaptor 1), Cd22 (CD22 antigen), Cd40 (CD40 antigen,) Cd28 (CD28 antigen) Vpreb3 (pre-B lymphocyte gene 3), Cd27 (CD27 antigen) Siglecg (sialic acid binding Ig-like lectin G), Clcf1 (cardiotrophin-like cytokine factor 1) and Swap70 (SWA-70 protein) are the top 10 DEGs based on adj. *p*-value. Detailed categories and sub-categories of dysregulated pathways are presented in Supplementary Table 3 and can be visualized in Supplementary Fig. 3. Visualization of the GO enrichment results were done by GOplot based on z-score (Supplementary Fig. 3). The z-score indicates whether BP, MF or CC are more likely to increase i.e., positive value or decrease i.e., negative value. In other words, it provides expression profile of genes within a GO term.

$$z-{score}=({up}-{down})/\surd {count}$$

Up and down means number of upregulated and downregulated genes in the GO term respectively.

For further analysis, main focus was imparted to BP from the “GO knowledgebase” which includes specifically signaling pathways, biological programs, and cellular functions which could be identified as therapeutic targets. The “Mammalian Metabolic Enzyme Database” (https://esbl.nhlbi.nih.gov/) was utilized to find DEGs (obtained from BP) which encode metabolic enzymes. In HD group, 16 DEGs were found to be encoding those metabolic enzymes that were involved in lipid, nucleotide, amino acid, and carbohydrate metabolism (Table 1). These 16 metabolic enzyme genes are arylacetamide deacetylase (Aadac), 4-aminobutyrate aminotransferase, mitochondrial (Abat), adenylate cyclase type 7 (Adcy7), retinal dehydrogenase 2 (Aldh1a2), plasma membrane calcium-transporting ATPase 3 (Atp2b3), ADP-ribosyl cyclase/cyclic ADP-ribose hydrolase 1 (Cd38), ectonucleotide pyrophosphatase/phosphodiesterase family member 4 isoform X6 (Enpp4), heme oxygenase 1 (Hmox1), inosine-5’-monophosphate dehydrogenase 2 (Impdh2), nitric oxide synthase, brain (Nos1), nitric oxide synthase, endothelial (Nos3), cGMP-specific 3’,5’-cyclic phosphodiesterase (Pde5a), group 10 secretory phospholipase A2 precursor (Pla2g10), calcium-dependent phospholipase A2 precursor (Pla2g5), prostaglandin G/H synthase 1 precursor (Ptgs1), and thymidine phosphorylase (Tymp) (Table 1). However, only one gene encoding to one enzyme cGMP-specific 3’,5’-cyclic phosphodiesterase (Pde5a) could be identified in LD group.

**Table 1:** Metabolic genes identified from “Mammalian Metabolic Enzyme Database” present in “Biological Process” of Gene Ontology in HD group.

Gene symbol	Annotation	Roche Chart Sector	Roche Chart Subsector	Sigma Chart Substrate	Sigma Chart General Term	Signma MiniMap Term
Aadac	Arylacetamide deacetylase	Lipid Metabolism: Carotenoids and Isoprenoids; Lipid Metabolism: Fatty Acids;	Carotenoids; Fatty Acid Oxidation;	Lipids; Vitamins, Co-Enzymes, and Hormones;	Isoprenoids; Lipid biosynthesis;	Lipid Metabolism;
						Products of Isoprene Metabolism;
Abat	4-aminobutyrate aminotransferase, mitochondrial	Amino Acid Metabolism: Glutamate, Proline, Hydroxyproline;	Asparate Group of Amino Acids;	Amino Acids;	Amino acids;	Pyrimidine Metabolism;
		Amino Acid Metabolism: Serine, Threonine, Cysteine, Methionine;	Glutamate Decarboxylation Pathway;			
Adcy7	Adenylate cyclase type 7	Carbohydrate Metabolism: Di-and Polysaccharides;	Polysaccharides;	Purines and Pyrimidines;	Purines;	
Aldh1a2	Retinal dehydrogenase 2	Amino Acid Metabolism: Glutamate, Proline, Hydroxyproline;	Anaplerotic Reactions; Carotenoids;	Amino Acids; Vitamins,	Amino acids; Isoprenoids;	Isoprene Metabolism;
		Amino Acid Metabolism: Histidine; Amino Acid Metabolism: Lysine; Carbohydrate Metabolism: Acidic Carbohydrate Derivatives;	Glutamate Decarboxylation Pathway;	Co-Enzymes, and Hormones;		Products of Isoprene Metabolism;
		Carbohydrate Metabolism: Glycolysis and Gluconeogenesis;	Histidine Metabolism; Lysine Metabolism;			Pyrimidine Metabolism;
		Carbohydrate Metabolism: Pyruvate Turnover; Lipid Metabolism: Carotenoids and Isoprenoids;	Mesaconate Pathway; Porphyrins; Zoosterols;			
		Lipid Metabolism: Glyco- and Phospholipids; Steroid Metabolism: Cholesterol Synthesis;				
		Tetrapyrrole Metabolism: Porphyrins, Cobalamin;				
Atp2b3	Plasma membrane calcium-transporting ATPase 3			Carbohydrates;	TCA Cycle in the Mitochondrial Matrix;	
Cd38	ADP-ribosyl cyclase/cyclic ADP-ribose hydrolase 1	Nucleotide Metabolism: NAD, NADP;				
Enpp4	Ectonucleotide pyrophosphatase/phosphodiesterase family member 4 isoform X6	Lipid Metabolism: Sphingolipids; Nucleotide Metabolism: Pyrimidines;	Pyrimidine Biosynthesis; Pyrimidines;	Lipids;	Phospholipids;	
Hmox1	Heme oxygenase 1	Tetrapyrrole Metabolism: Heme,	Bile Pigments;			
		Cytochromes, Cholorphyll;				
Impdh2	Inosine-5’-monophosphate dehydrogenase 2	Nucleotide Metabolism: Purines;		Purines and Pyrimidines;	Purines;	Purine Metabolism;
Nos1	Nitric oxide synthase, brain			Amino Acids;	Amino acids;	Deamination of Amino Acids:
						The Urea Cycle;
Nos3	Nitric oxide synthase, endothelial			Amino Acids;	Amino acids;	Deamination of Amino Acids:
						The Urea Cycle;
Pde5a	cGMP-specific 3’,5’-cyclic phosphodiesterase	Carbohydrate Metabolism: Di-and Polysaccharides;	Polysaccharides;			Prostaglandins, Thromboxanes,
						and Leucotrienes;
Pla2g10	Group 10 secretory phospholipase A2 precursor	Lipid Metabolism: Fatty Acids; Lipid Metabolism: Glyco- and Phospholipids;	Fatty Acid Oxidation; Phosphatides;	Lipids;	Phospholipids;	Prostaglandins, Thromboxanes,
						and Leucotrienes;
Pla2g5	Calcium-dependent phospholipase A2 precursor	Lipid Metabolism: Fatty Acids; Lipid Metabolism: Glyco- and Phospholipids;	Fatty Acid Oxidation; Phosphatides;	Lipids;	Phospholipids;	Prostaglandins, Thromboxanes,
						and Leucotrienes;
Ptgs1	Prostaglandin G/H synthase 1 precursor			Lipids;	Lipid biosynthesis;	
Tymp	Thymidine phosphorylase	Nucleotide Metabolism: Pyrimidines;	Pyrimidines;	Purines and Pyrimidines;	Pyrimidines;	Pyrimidine Metabolism;

Additionally, KEGG (Kyoto Encyclopedia of Genes and Genomes) pathway database was also utilized to analyze the biological pathways in which DEGs were involved. Based on DEGs data, 18 KEGG pathways in LD group and 12 pathways in HD group were found to be significantly enriched (adj. *p*-value < 0.05) (Supplementary Table 4). These enriched pathways includes hematopoietic cell lineage, primary immunodeficiency, cell adhesion molecules, and intestinal immune network for IgA production which were found common in both the dose groups. Hematopoietic cell lineage pathway showed highest significance in HD group. DEGs associated with hematopoietic cell lineage pathway can be visualized in Supplementary Fig. 4. Among the 33 DEGs involved, only 9 DEGs (Cd59b, Gp1bb, Itga3, Kit, Itga2b, Il1r2, Gp5, Cd9, Gp1ba) showed upregulation whereas 24 DEGs (Ms4a1, Cd19, H2-Ab1, H2-Eb2, H2-Ob, H2-Aa, H2-Eb1, Fcer2a, H2-DMb2, H2-DMb1, Cd22, H2-Oa, Cd8b1, H2-DMa, Cd38, Cd8a, Cr2, Cd55, Cd5, Cd2, Cd1d1, Cd3d, Cd3g, Cd4) showed downregulation in HD group. Whereas in LD group, only 18 genes were found significant in hematopoietic cell lineage pathway.

### Metabolomics/Lipidomics profiling and pathway analysis

**Fig. 2:**
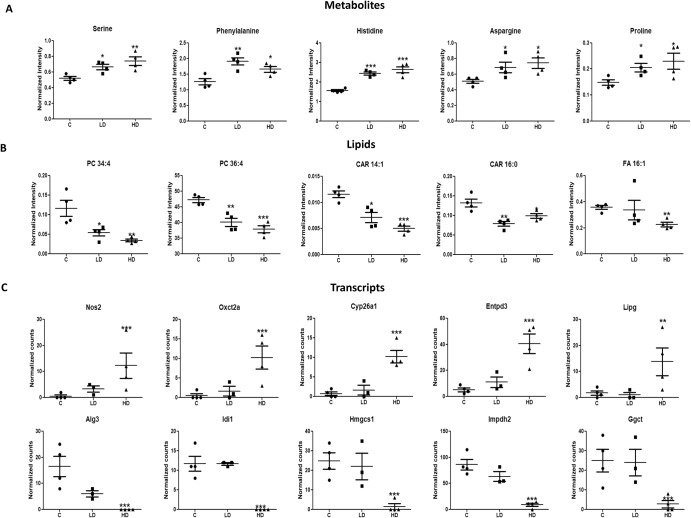
Differential changes observed using metabolomics and transcriptomics. Scatter plot showing significantly altered metabolites (**A**), lipids (**B**) and DEGs (**C**). Data is presented as mean ± standard error of means (number of biological replicates = 4). (For metabolites and lipids: *p-value < 0.05, **p-value < 0.01 and ***p-value < 0.001 using student’s t test (two-tailed); For transcripts * adj. *p*-value < 0.05, ** adj. *p*-value < 0.01 and *** adj. *p*-value < 0.001 using Wald statistics (two-tailed).

UPLC-MS-based untargeted metabolomics was conducted to identify changes in metabolites and lipids in plasma samples post-radiation exposure. During metabolic analysis of plasma, 3426 peaks in positive and 2548 peaks in the negative ionization mode were detected, whereas lipidomics analysis identified 2315 and 1056 peaks in positive and negative ionization mode respectively. Overall, the univariate analysis identified a total of 1491 significant m/z features (lipids and metabolites together; 696 downregulated and 795 upregulated) in LD group and 1221 features (424 features downregulated and 797 upregulated) in HD group (*p*-value < 0.05). Supplementary Table 5 lists identified metabolites and lipids with mass error in delta (Da). Significant identified metabolites and lipids can be visualized in Fig. 2 and Supplementary Table 6. PCA plot exhibits separation of HD group from controls and some partial overlap of LD with controls and HD can also be seen in both metabolic and lipid profiles (Supplementary Fig. 2). Serine, phenylalanine, histidine, aspargine and proline was upregulated irrespective of exposed dose (Fig. 2). Lipid categories common in both radiation group includes glycerophospholipids [glycerophosphocholines (PC), glycerophosphoethanolamines (PE), LysoPE, LysoPC], fatty acyls (fatty acids/esters (FA), acyl carnitines (CAR)], glycerolipids [tri(acyl|alkyl)glycerols (TG), di (acyl|alkyl)glycerols (DG)], cholesterol esters (CE).

**Fig. 3:**
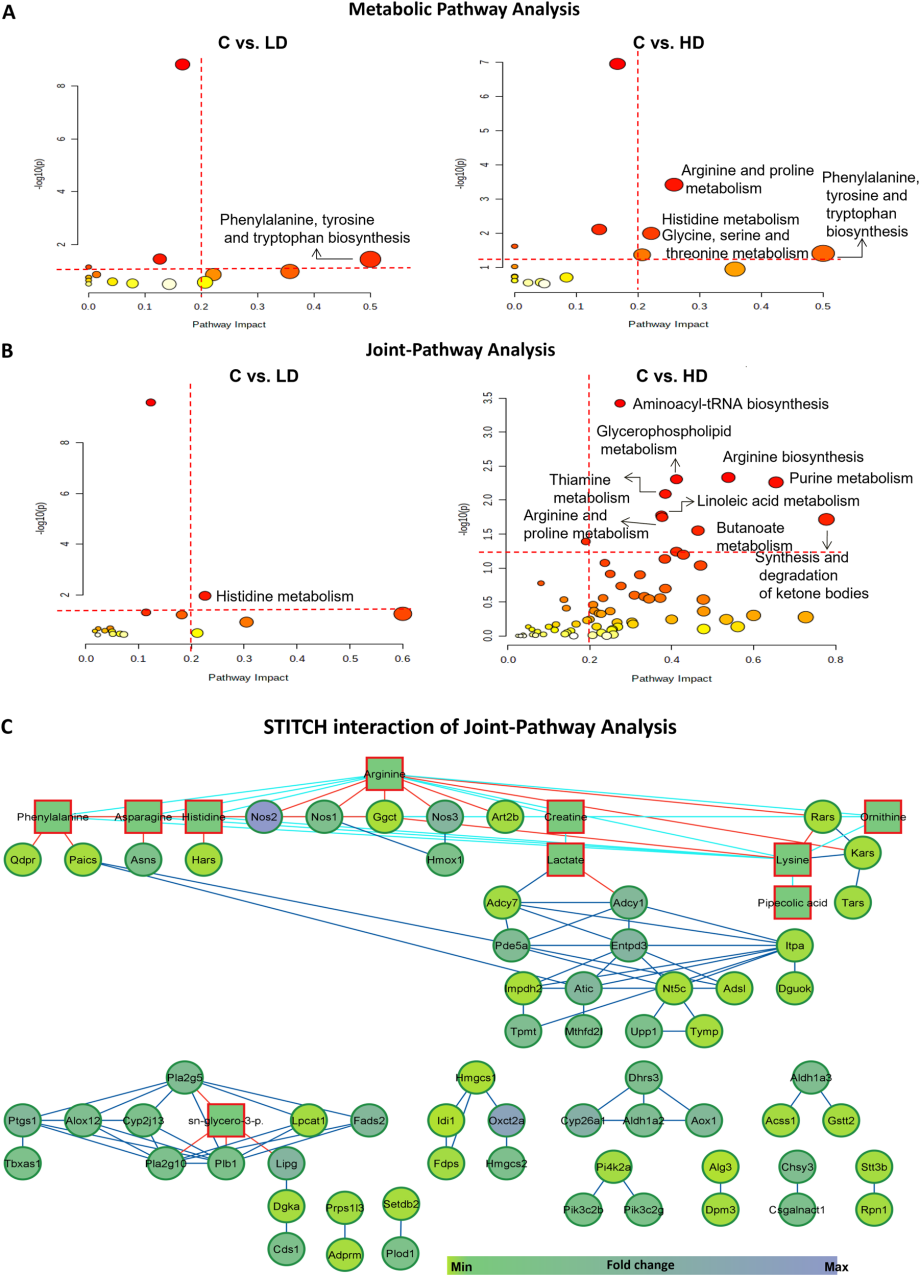
Overview of dysregulated metabolic pathways after radiation exposure. **A** KEGG pathway analysis of significantly changed metabolites using MetaboAnalyst. (*p* values shows pathway enrichment analysis using Global Test and pathway impact values shows the pathway topology analysis). **B** Joint-Pathway Analysis of significantly altered transcriptomic and metabolomic data after integration. Pathways having impact > 0.2 and –log 10(p) >1.3 (cutoff is highlighted in red dotted lines) were taken into consideration. Each circle represents a single metabolic pathway with area of circle proportional to pathway impact whereas color represents the pathway significance from highest (red) to lowest (yellow). (Enrichment analysis was performed based on hypergeometric test whereas topology measure was performed by degree centrality). **C** Network visualization of STITCH interactions by Cytoscape in HD group. Networking shows interactions between significant metabolites (*p*-value < 0.05; square) and DEGs (adj. *p*-value < 0.05; circle) with the thickness of edge proportional to the interaction score in the STITCH database. Node color shows minimum and maximum fold change. Red, blue and dark blue edge color represents the interaction between metabolite-gene, metabolite-metabolite and gene-gene respectively.

Further, pathway analysis demonstrated that a total of 18 and 15 pathways were altered in HD and LD groups respectively. Among them, 4 pathways namely, “arginine and proline metabolism”, “histidine metabolism”, “phenylalanine, tyrosine and tryptophan biosynthesis”, and “glycine, serine and threonine metabolism” showed a significant impact (*p*-value < 0.05; pathway impact >0.2) in HD group. Whereas, in LD group, only phenylalanine, tyrosine and tryptophan biosynthesis pathway had a significant impact >0.2 (Fig. 3A, Supplementary Table 7).

### Integration of metabolic/lipidomic and transcriptomic data

The involvement of GO enrichment in metabolic pathways was less evident along with the limited number of identified DEGs that function as metabolic enzymes. Therefore, in order to greatly improve the ability to comprehend transcriptomic data, we employed a multi-omics integrated approach utilizing Joint-Pathway Analysis (MetaboAnalyst), BioPAN software, and STITCH (Search Tool for Interactions of Chemicals) interaction to understand changes in metabolic pathways after IR exposure.

#### Differential networking by Joint-Pathway Analysis and STITCH interaction analysis

Joint-Pathway Analysis generated 16 and 71 altered pathways in LD and HD groups respectively (Tables 2 and 3). Pathways with high impact >0.2 in HD group included “aminoacyl-tRNA biosynthesis”, “arginine biosynthesis”, “glycerophospholipid metabolism”, “purine metabolism”, “thiamine metabolism”, “linoleic acid metabolism”, “arginine and proline metabolism”, “synthesis and degradation of ketone bodies”, “butanoate metabolism”, and “alpha-linolenic acid metabolism” (Table 3). In LD group, only histidine metabolism demonstrated a significant impact >0.2 (Table 2, Fig. 3B). It is worth noting that more number of metabolic pathways were significantly enriched under Joint-Pathway Analysis using both genes and metabolites as compared to the separate pathway analysis of transcriptomics and metabolomics. Furthermore, the transcript-metabolite interaction network for significant transcripts (DEGs) and the metabolites engaged in Joint-Pathway Analysis was also created using STITCH interaction analysis to attain more comprehensive information. This approach has thus helped in visualizing the relationship between functionally linked metabolites and genes.

**Table 2:** Pathway integration by Joint-Pathway Analysis of DEGs and metabolites of LD group with controls.

Pathways in LD group	Total	Raw *p*	Holm adjust	FDR	Impact	Matched features
Aminoacyl-tRNA biosynthesis	74	2.63 × 10^−10^	2.21 × 10^−08^	2.21 × 10^−08^	0.12329	cpd:C00152; cpd:C00135; cpd:C00079; cpd:C00065; cpd:C00073; cpd:C00123; cpd:C00078; cpd:C00148
Histidine metabolism	32	0.01059	0.87874	0.44466	0.22581	cpd:C00135; mmu:140483
Cysteine and methionine metabolism	71	0.04757	1	0.98896	0.11429	cpd:C00065; cpd:C00073

(“cpd” represents “KEGG ID” whereas “mmu” represents “Entrez ID” of DEGs).

**Table 3:** Pathway integration by Joint-Pathway Analysis of DEGs and metabolites of HD group with controls.

Pathways in HD group	Total	Raw p	Holm adjust	FDR	Impact	Matched features
Aminoacyl-tRNA biosynthesis	74	0.000376	0.0316	0.0316	0.27397	cpd:C00152; cpd:C00135; cpd:C00079; cpd:C00062; cpd:C00065; cpd:C00047; cpd:C00148; mmu:15115; mmu:104458; mmu:22321; mmu:85305; mmu:110960; mmu:67417
Arginine biosynthesis	27	0.004647	0.38569	0.11503	0.53846	cpd:C00062; cpd:C00077; mmu:18125; mmu:18126; mmu:18127; mmu:18416
Glycerophospholipid metabolism	86	0.004936	0.40475	0.11503	0.41176	cpd:C00670; mmu:210992; mmu:236899; mmu:18784; mmu:26565; mmu:665270; mmu:13139; mmu:331374; mmu:381925; mmu:14555; mmu:74596; mmu:66358
Purine metabolism	169	0.005478	0.44369	0.11503	0.65476	mmu:328099; mmu:108147; mmu:11564; mmu:67054; mmu:54611; mmu:11513; mmu:432530; mmu:215446; mmu:18103; mmu:11636; mmu:11639; mmu:229949; mmu:50773; mmu:23918; mmu:242202; mmu:16434; mmu:27369; mmu:66358; mmu:224794
Thiamine metabolism	14	0.008126	0.6501	0.13652	0.38462	mmu:18041; mmu:11636; mmu:11639; mmu:229949
Linoleic acid metabolism	17	0.016754	1	0.20215	0.375	mmu:230459; mmu:18784; mmu:26565; mmu:665270
Arginine and proline metabolism	78	0.017854	1	0.20215	0.37662	cpd:C00062; cpd:C00300; cpd:C00148; cpd:C00077; mmu:18125; mmu:18126; mmu:18127; mmu:20810; mmu:66194; mmu:69051
Synthesis and degradation of ketone bodies	10	0.019253	1	0.20215	0.77778	mmu:15360; mmu:208715; mmu:64059
Butanoate metabolism	29	0.028148	1	0.26272	0.46429	mmu:64059; mmu:15360; mmu:208715; mmu:268860; mmu:14415
alpha-Linolenic acid metabolism	22	0.040688	1	0.34178	0.19048	mmu:18784; mmu:26565; mmu:665270; mmu:56473
Glycerolipid metabolism	35	0.057552	1	0.43948	0.41176	mmu:16891; mmu:381925; mmu:225913; mmu:13139; mmu:331374

(“cpd” represents “KEGG ID” whereas “mmu” represents “Entrez ID” of DEGs).

The transcript-metabolite interaction in HD group consists of 114 nodes connected by 81 edges, with a clustering coefficient of 0.889. Whereas no significant interaction was observed in the case of LD group. Here, interactions mean associations between gene-gene, metabolite-gene, and metabolite-metabolite and are clearly shown using Cytoscape (Fig. 3C). Associations do not always imply that there is a physical binding present, but they might also contribute to a shared biological function. Network edge represents confidence that indicates the strength of data support. In other words, associations can be based on reactions from pathway databases, literature associations, similar structures or similar activities^13^. It is apparent that arginine and lysine have shown the maximum number of associations (*n* = 6) with other metabolites. Among various dysregulated genes present in the Cytoscape network, 3-oxoacid CoA transferase 2 A (Oxct2a) (upregulated), 3-hydroxy-3-methylglutaryl-Coenzyme A synthase 2 (Hmgcs2) (upregulated), and isopentenyl-diphosphate delta isomerase (Idi1) (downregulated) displayed high fold change (>10) and were present in the same cluster. Along with them, nitric oxide synthase 2 (Nos2) was also upregulated 17 times and exhibited an association with arginine and histidine. Altogether, from the above elucidation, it is evident that the integration analysis was successful in identifying differential pathway networks and their linked metabolites and DEGs associated with radiation-induced injury.

#### Lipid pathway analysis

**Fig. 4:**
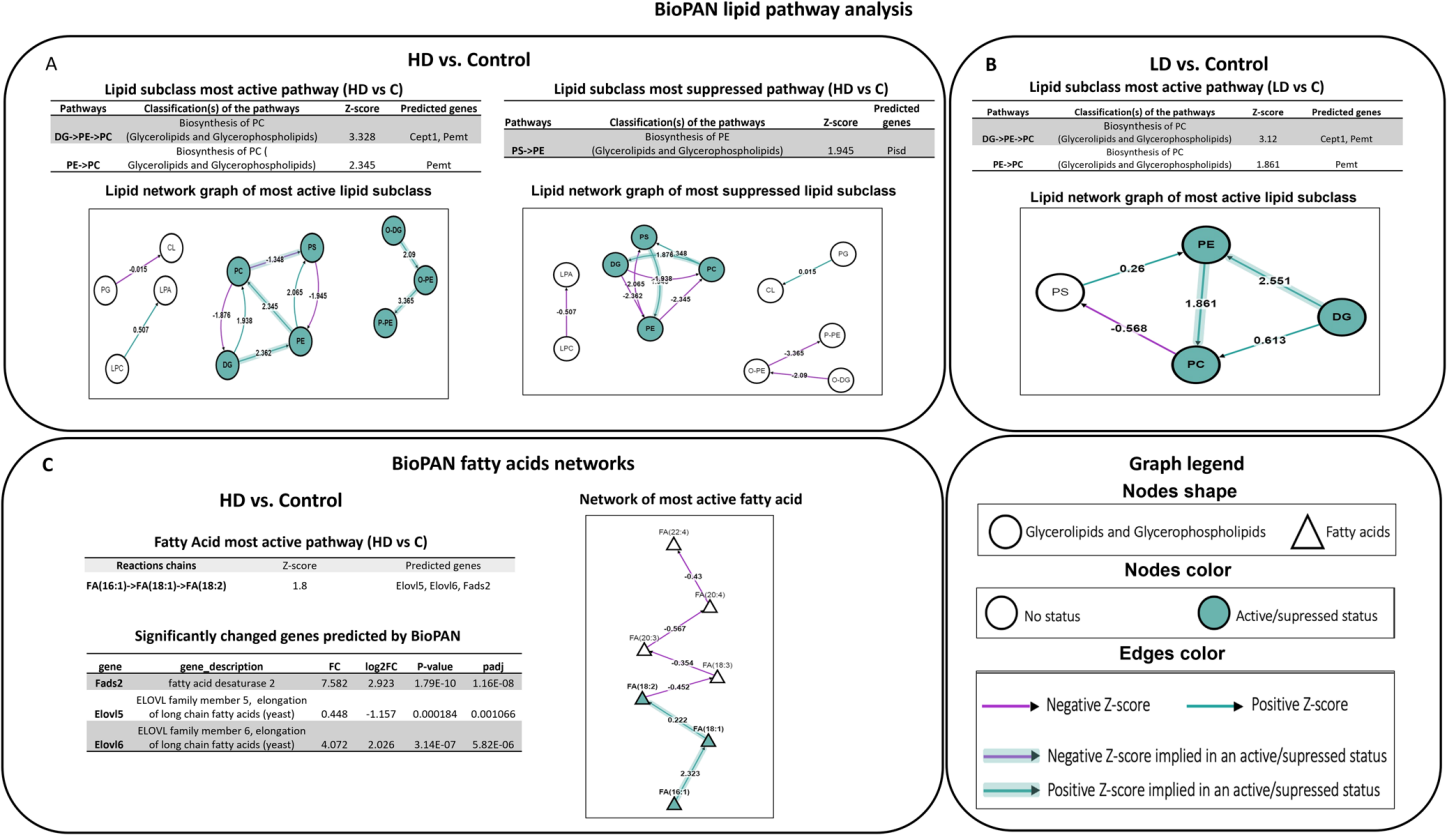
Lipid network generated using BioPAN software. Panels **A** and **B** represents active or suppressed lipid pathways in HD and LD groups respectively. Panel **C** represents the active fatty acid pathway in HD group compared to the control. Green nodes represent active lipids whereas green shaded arrows represent active pathways. (*p*-value < 0.05 equivalent to Z-score >1.645; represents significantly altered reaction between the control and irradiated group; Green arrow: positive Z-score; purple arrow: negative Z-score).

In the present study, lipid pathways based on lipidomic data were analyzed using BioPAN. The network of lipid subclasses and fatty acid (FA) pathways of LD and HD groups is presented in Fig. 4A, B. Pathway analysis revealed the biosynthesis of PC (glycerolipids and glycerophospholipids) as the most active pathway in both the irradiated groups whereas biosynthesis of PE (glycerolipids and glycerophospholipids) was observed as the most suppressed pathway only in HD group (Fig. 4). These findings clearly indicate common as well as distinct pathways following radiation exposure at different doses. On the other hand, the FA pathway was found to be hampered only in HD group with Elovl5, Elovl6 and Fads2 as the observed predicted genes. Fads2 (fatty acid desaturase 2), Elovl5 (ELOVL family member 5, elongation of long-chain fatty acids), and Elovl6 (ELOVL family member 6, elongation of long-chain fatty acids) were also amongst the DEGs identified in HD group (Fig. 4C). It noticeably displays the complementary information obtained from different omics.

## Discussion

In order to facilitate the clinical management of radiation-exposed people during a nuclear accident, several international health and research agencies are looking for high throughput biomarkers as diagnostic tools for immediate use in dose assessment. Multiple potential biomarkers based on genes, proteins and metabolites for radiation injury are being identified and validated across the globe^4^. In this study, we have performed an integrated metabolomic and transcriptomic analysis in the preclinical model exposed to low and high doses of radiation for biomarker identification.

A total of 2837 and 143 DEGs in HD and LD groups respectively, were identified between irradiated groups and controls, 93 of which were common to both the irradiated groups showing that they are sensitive to even 1 Gy of radiation dose [LD dose group] (Supplementary Table 8). However, dose-dependent changes were quite evident from the lesser number of DEGs observed in LD group as compared to HD group. A similar pattern was also observed in the metabolomics/lipidomics data, wherein 53 metabolites/lipids amongst the identified features were found common in both the irradiated groups. In addition, 59 and 57 metabolites/lipids were found unique to the high and low-dose exposed groups respectively (Supplementary Table 6). Numerous transcriptomic and metabolomic investigations conducted in recent years have shown variations in transcript and metabolite patterns associated with radiation exposure^12,14–17^. The complementing evidence given by transcriptomics and metabolomics on alterations at the genetic level, protein synthesis, metabolism along with cell function represents the changes in genotype and phenotype, respectively^7^. This study is unique as the integration of transcriptomic and metabolomic data has been used for the identification of blood-based biomarkers for radiation exposure.

The molecular mechanism and relationships between the identified genes were defined using GO analysis. DEGs were enriched in various cellular pathways in the radiation-exposed group when they were compared with controls. Radiation exposure is known to elicit an immune response and the same has been reflected in our transcriptomic data as many of the significantly identified DEGs were enriched in immunological functions in both the radiation groups. Fang et al. has also shown similar observations on exposure to low-dose radiation^13^. The group showed that ‘signal transduction,’ ‘immune system,’ ‘cancer: overview,’ ‘transport and catabolism’ and ‘signaling molecules and interaction’ were significantly enriched in GO. Radiation results in apoptosis of B and T lymphocytes which are mainly responsible for mediating adaptive immunity. This causes lethal damage to monocytes and granulocytes precursors which are involved in innate immunity in bone marrow stem cells. Hence, resulting in various immunological response changes after radiation exposure as observed in this study^18,19^. Supplementary Table 3 highlights the various genes which are significantly altered in various immunological pathways. It is evident from the results that a radiation dose as low as 1 Gy can elicit changes in immunological functions. Interestingly, we also observed hematopoietic cell lineage pathway in both radiation groups as well. Acute myelosuppression and long-term hematopoietic injury are caused by IR due to direct or indirect damage to hematopoietic stem cells. A review by Lu et al. has explained in detail the mechanisms regulating the hematopoietic stem cells after radiation exposure by affecting DNA damage response (DDR), including DNA-damage repair, cell-cycle arrest, apoptosis and senescence, or bone marrow microenvironment^20^.

Moreover, these transcriptional changes were dose-dependent as the number of DEGs, and fold change level was significantly higher in the high-dose group. In addition, transcriptomics study revealed DEGs associated with antigen processing, complement activation, calcium signaling, ion channel activity, and G protein-coupled receptor activity in animals exposed to 7.5 Gy (HD) radiation dose (Supplementary Table 3). These findings are also supported by KEGG pathway analysis that displayed “antigen processing and presentation” only in HD group along with other pathways associated with “primary immunodeficiency”, “cell adhesion molecules” and “hematopoietic cell lineage” in both the radiation groups (Supplementary Table 4). Based on the differential observations in two irradiated groups obtained from GO pathway analysis, our study substantiates that certain molecular signaling pathways may become activated only when radiation exposure reaches a certain dose level. Overall, these observations support changes in the immunological process post-radiation exposure.

On the other hand, untargeted metabolomics identified a total of 13 and 15 dysregulated circulating metabolites in LD and HD groups respectively when compared to controls that were involved in “lipid metabolism and amino acid metabolism” in metabolic pathway analysis. Moreover, KEGG pathway analysis of metabolites revealed “phenylalanine, tyrosine and tryptophan biosynthesis” as the common dysregulated pathway in both the radiation groups (Supplementary Table 7; impact >0.2; *p*-value < 0.05). We also observed a significant change in L-histidine in both the radiation groups, which is an important metabolite of “histidine metabolism” pathway and acts as a proton buffer, scavenger of nitric oxide (NO) and reactive oxygen species (ROS), and is also involved in anti-inflammatory function^21^. Increased histidine in both the radiation groups in the present study also aligns with an earlier study^22^.

We also performed differential networking using Joint-Pathway Analysis. The integrated analysis of transcriptomics and metabolomics provided visualization of the interplay between DEGs and dysregulated metabolites/lipids. Data integration revealed uniquely enriched pathway networks in the irradiated group in comparison with controls which were not significant in their individual analysis alone. Overall, when both transcriptomics and metabolomics data were merged for analysis, the number of hits within a particular pathway increased, affecting the pathway impact as well as its significance (Fig. 3B). Data integration illustrated 3 and 10 enriched pathways (*p*-value < 0.05) in LD and HD exposed groups respectively when compared with controls. Differential networking by Joint-Pathway Analysis and STITCH presented a good metabolite–gene interaction network in high dose group (Fig. 3C and Table 3). Furthermore, STITCH networking highlighted one distinct cluster for DEGs and metabolites involved in “glycerophospholipid metabolism” as well as “synthesis and degradation of ketones”. Whereas others are showing associations among themselves suggesting that they are involved directly or indirectly in corresponding pathways. However, networking could not be well appreciated in the low-dose group due to the limited number of significant DEGs and metabolites. An important metabolic pathway that observed a good number of DEGs hit was nucleotide metabolism. Though metabolite hits could not be observed in plasma owing to the limited sensitivity of the chromatographic method and column used in the present study. However, deaminated purines and pyrimidines in other biological matrices (urine) have been observed in earlier studies^23^. Also, altered amino acids and associated pathways identified in plasma in this study correlate well with our earlier study on urine samples^3,12^. Upregulated arginine and associated genes with “arginine biosynthesis” pathway have been observed in Joint-Pathway Analysis. Dysregulated “arginine and proline metabolism” has also been observed in mice exposed to 4 Gy X-ray radiation^24^. Another interesting finding by DEGs showed upregulation of Nos (nitric oxide synthase) in our study. Upregulation of Nos is reported in the inflammatory and hypoxic condition in an earlier study^25^. A dose-dependent increase of plasma Nos has also been observed at 24 h post irradiation in rats^26^. We also utilized Joint-Pathway Analysis for finding differential networking between LD and HD groups as well. Differential networking was able to differentiate these group based on “Arginine biosynthesis” pathway (Supplementary Fig. 5A). Furthermore, lipid pathway by BioPAN showed most active and suppressed lipid subclasses. However, no significant lipid as well as fatty acid pathway was observed in HD vs. LD comparison. Lipid pathway analysis suggests that although changes were observed among lipid subclasses, but they could not contribute significantly to lipid pathway. Overall, our observation demonstrated differential response in terms of both Joint Pathway Analysis and lipid pathway in LD and HD groups (Supplementary Fig. 5B).

In our earlier studies, we found changes in energy metabolism post radiation exposure in urine^2,3,12^. Similarly, some DEGs observed in present study are found to be linked indirectly with energy metabolism. Upregulation of Hmgcs2 (FC 12.6) and Oxct2a (FC 14) observed in our study can be linked to dysregulation of “synthesis and degradation of ketone bodies”. Interestingly, Hmgcs2 is also involved in the rate-limiting step of ketogenesis^27^. These two DEGs have also shown association in STITCH analysis predicting elevated energy demand at the systemic level after radiation exposure. Similarly perturbed ketogenesis after radiation exposure has also been shown in earlier studies^28^. Likewise, another pathway “butanoate metabolism” can also be linked to energy metabolism as it can get involved directly in the citric acid cycle via succinate or indirectly via pyruvate and acetyl-CoA^29^. The above observations, by and large, infer changes in energy metabolism post TBI.

Our findings have also been able to delineate different pathways associated with lipids based on lipidomic data. Pathway analysis using BioPAN showed “biosynthesis of PC” as the most active pathway in both radiation groups whereas “biosynthesis of PE” was the most suppressed pathway observed in HD group only (Fig. 4A, B). This has further been substantiated by our Joint-Pathway Analysis that showed dysregulated “glycerophospholipid metabolism” in HD group (Table 3). These findings support hampered lipid pathways after radiation exposure however changes are pertinent to dose level. Radiation exposure induces lipid peroxidation as well as dyslipidemia leading to cellular damage^30,31^. Lipids play different roles apart from cell membrane formation and storage of energy including cellular signaling and inflammatory response^32^. The influence of IR on apoptosis has been reported widely. Some of the lipids reported in this study like PCs, LysoPCs (LPC), and LysoPEs (LPE), have been linked to apoptosis in previous studies^33^. On a similar note, PCs play a major role in cell signaling through the generation of LPCs, phosphatidic acids (PA) and diacylglycerol (DG)^22^. Changes observed in LPCs, and PCs in this study indicates radiation-induced cell membrane damage as well as dysregulation at molecular level. Linoleic acid metabolism, alpha-Linolenic acid metabolism and glycerophospholipid metabolism have also been observed in both rats and mice after radiation exposure in a recent study^24,34^. It is known that linoleic acid is involved in the regulation of cholesterol synthesis and hence affect lipid metabolism as well. Dysregulated linoleic acid metabolism may also be involved in apoptosis and inflammation^34^. Lipid pathways identified through lipidomic data analysis have been correlated well with pathways (fatty acid, glycerophospholipid, and glycerophospholipid metabolism) identified through KEGG based on transcriptomic data (Supplementary Table 4B). Joint-Pathway Analysis has also reported some lipid-associated metabolism viz a viz glycerolipid metabolism, fatty acid elongation and arachidonic acid metabolism which are in agreement with previous reports^22^. Additionally, BioPAN has predicted three genes: Elovl5, Elovl6, and Fads2 for dysregulated FA(16:1) (Palmitoleic acid)->FA(18:1) (Oleic acid)->FA (18:2) (Linoleic acid)” pathway. Joint-Pathway Analysis has also shown significant involvement of these genes in “fatty acid elongation” denoting complementarity of lipidomics and transcriptomics data. Furthermore, these 3 DEGs have also shown confidence in STITCH interaction networking with glycerophosphocholine (Fig. 3C). Although fatty acids are important members of the membrane matrix, they also serve as secondary messengers as well as a source of energy production^35^. The study further highlighted the significance of integration of transcriptomics with metabolomics through CAR 18:1 (octadecanoylcarnitine), CAR 16:2 ((7Z,10Z)-hexadecadienoylcarnitine) or ST 23:1;O4, CAR 16:0 (palmitoylcarnitine) and CAR 14:1 (tetradecenoylcarnitine) which were significantly decreased in both radiation groups and seen to be linked with increased energy demand through fatty acid oxidation/degradation.

The findings are also in support of already identified metabolite markers available in the literature for radiation exposure. Earlier research suggested that radiation exposure causes alterations in the lipidome^36–38^. Decreased PC 30:0, PC 34:4, PC 36:2, PC 36:3, PC 36:6, PC 36:4 and PC 38:5 in our study is also concurrent with the study by Pannkuk et al.^39^. They showed that these polyunsaturated PCs were significantly decreased following different radiation dose exposure in non-human primate (NHP)^39^. Similarly, a recent study by Crook et al. group has illustrated the downregulation of six carnitine metabolites even at day 4 compared to baseline. CAR 18:1 and CAR 14:2 were also found to be downregulated in NHPs at 24 h post-radiation^40^. Changes in acylcarnitines along with phospholipids and amino acids indicate an alteration in the metabolism of proteins, beta-oxidation and changes in inflammatory pathways^39^. Increased LPCs at day 2 have also been observed in an earlier study in NHP by Pannkuk et al. indicating the potentialities of these compounds as possible markers for biodosimetry^36^. The plasma lipidome thus reported changes in specific lipid concentrations after radiation exposure.

In the present study, we have observed upregulation of amino acid at 24 h post radiation exposure which is in concurrence with the previous studies that had also shown changes in amino acids like valine, proline, glutamate, histidine, alanine, arginine, alanine, and asparagine, tyrosine in NHP serum after IR^39^. Recently, changes in histidine metabolism have also been observed by Sato et al., though the changes observed were in contrast to our findings^41^. Some of these changes complement pathways based on transcriptomic data for their validation. Largely, the integrated omics has helped us in understanding the dysregulated pathways and ameliorating inter-linking between them to gain a better insight into molecular mechanisms during radiation injury.

Overall, the combination of transcriptomics and metabolomics approaches have brought better insight into radiation-induced changes at the metabolic level. Primarily, the present study identified interactions among DEGs and dysregulated metabolites/lipids based on extensive pathway analysis. Overall, radiation exposure resulted in changes in immunological processes, carbohydrate metabolism (synthesis and degradation of ketone bodies, butanoate metabolism), lipid metabolism (glycerophospholipid metabolism, linoleic acid metabolism, alpha-linolenic acid metabolism), amino acid metabolism (arginine biosynthesis, arginine and proline metabolism, histidine metabolism,) and nucleotide metabolism (purine metabolism, thiamine metabolism). To the best of our knowledge, the approach used in the present study has not been applied so far after total body radiation exposure in mice. Biomolecules crosstalk synergistically for signal perception and derive biological functions. A single “omics” field cannot adequately capture biological complexity. The present study supports the drive for an integrated multi-omics approach that would not only help in looking for deeper insights into crosstalk between different biomolecules but also in substantiating the validation of the identified biomarkers mechanistically for radiation exposure.

## Materials and methods

### Chemicals

All solvents, isopropyl alcohol, acetonitrile, water, and methanol were of LC-MS grade (Fisher Scientific). Lipid internal standards including lysophosphatidylethanolamine 17:1, lysophosphatidylcholine 13:0, phosphatidylethanolamine 15:0, and 1-(1Z-octadecenyl)-2-oleoyl-sn-glycero-3-phosphocholine (C18(plasm)-18:1PC) were purchased from Avanti polar lipids, Inc. (Alabaster, Alabama, US). Taurine (1,2-^13^C_2_) from Cambridge isotope laboratories, Inc. whereas caffeine, nitrobenzoic acid, ammonium formate (AF), formic acid and tert-butyl methyl ether (MTBE) were purchased from Sigma-Aldrich (St. Louis, Missouri, US).

### Animals

C57BL6 male mice (8–10 weeks old) were obtained from the animal facility of the institute followed by one-week acclimatization before irradiation. Temperature and humidity conditions were properly maintained. Animals were randomly allocated to three groups (*n* = 8 each): control, low dose, and high-dose group. Animals were exposed to 1 Gy (Low dose, LD) and 7.5 Gy (High Dose, HD) of total-body gamma radiation through a ^60^Co source. Blood was collected through retro-orbital sampling at 24 hours post-radiation exposure.

#### Ethics

All the methods used in the study were approved by institutional animal ethics committee at the Institute of Nuclear Medicine and Allied Sciences (INMAS), DRDO, India with approval number 8/GO/RBI/S/99/CPCSEA/INM/IAEC/2017/09.

### Metabolomics and Lipidomics

#### Sample collection and processing

Whole blood collection was done using BD Vacutainer EDTA tubes. Plasma samples were processed for extraction of lipids and polar metabolites using MTBE and methanol. In brief, 50 μL of plasma was mixed with 370 μL cold MTBE and 125 μL methanol followed by vortexing and incubation for 15 minutes at 4^o^C. Water containing 0.1% formic acid (100 μL) was added for phase separation followed by vortexing and centrifugation at 13,000 *g* for 20 minutes at 4 °C. The upper organic layer containing lipids was collected in a separate tube. For polar metabolites, 400 μL of cold methanol was added to the remaining phase, followed by centrifugation at 13,000 *g*, 20 minutes at 4 °C. The supernatant containing polar metabolites was separated. Both organic and polar phases (supernatant) were evaporated in a nitrogen gas evaporator (Crescent scientific, INDIA).

For the metabolomics study, the evaporated extract was resuspended in 500 μL of mobile phase (1:1 v/v), containing internal standards (IS) caffeine (0.5 μg/ml), nitrobenzoic acid (0.5 μg/ml) and 1,2-^13^C_2_ labeled taurine (6 μg/ml; Cambridge isotope laboratories, Inc.). Prepared samples were then filtered through 3 kDa filters. The evaporated lipid samples were reconstituted in 500 μL of 1:2 v/v of mobile phase A: B containing all IS i.e., lysophosphatidylcholine 13:0 (0.5 μg/ml), C18(plasm) (2.5 μg /ml), phosphatidylethanolamine 15:0 (6 μg/ml) and lysophosphatidylethanolamine 17:1 (2 μg/ml). By combining equal aliquots from each of the prepared samples, a quality control (QC) sample was also prepared.

#### LC-MS Analysis

LC-MS/MS analysis was performed using a Sciex 5600 + UHPLC MS-Q-TOF system (AB Sciex, Singapore) using an acquity bridged ethylene hybrid (BEH) amide (100 mm × 2.1 mm,1.7μm) (Waters, Milford, Massachusetts, US) and charged surface hybrid (CSH) C18 (100 mm × 2.1 mm, 1.7 μm) (Waters, Milford, Massachusetts, US) column with their compatible vanguard pre-column for metabolomics and lipidomics respectively. A multistep gradient elution method was used for the metabolomics and lipidomics study.

For metabolomics analysis, mobile phase A (water + 0.1% formic acid) and mobile phase B (acetonitrile + 0.1% formic acid) were used as follows: the initial mobile phase was 95% B for 3 mins; followed by 70% B from 3–4 min, 45% B from 4–11 min, 45% B from 11–15 min, 95% B from 15–15.1 min followed by equilibration at 95% B for one minute.

For lipidomics, mobile phase A consisting of water:acetonitrile (40:60 v/v) having 10 mM AF and 0.1% formic acid and mobile phase B consisting of isopropanol:acetonitrile (80:20 v/v) having 10 mM AF and 0.1% formic acid was used as follows for chromatographic separation: the initial mobile phase was 15% B followed by 30% B from 0–2 min, 48% B from 2–2.1 min, 55% B from 2.1–2.5 min; 70% B from 2.5–5 min, 75% B from 5–6.5 min; 82% B from 6.5–8 min; 99% B from 8–14 min, 99% B for 3 min, 15% B from 17–17.1 followed by equilibration at 15% B from17.1–19 min. A flow rate of 0.2 mL/min was used for both metabolomics and lipidomics analysis, whereas injection volume of 10 μL (in both positive and negative mode of ionization) for metabolomics analysis through BEH amide column and injection volume of 2 μL in positive mode and 4 μL in negative ionization mode for lipidomics analysis through CSH column.

The mass spectrum was acquired in data-dependent acquisition (DDA) in both positive and negative ionization modes. The different MS parameters used in the study are outlined in our earlier study^3^. Mass spectrometry was performed with the following conditions: nebulizer gas (GS1) = 50 psi, desolvation gas (GS2) = 50 psi, curtain gas flow = 30 psi, capillary voltages (ISVF) = 5500 V for positive and 4500 V for negative ionization modes with source temperature = 400 °C for metabolomics and 500 °C for lipids with flow rate of 200 μL/min. Parameters used for MS/MS fragmentation include collision energy (CE) of 20 eV for metabolites and 35 eV for lipids with a CE spread of 15 eV. The mass range was set at 60–1000 m/z and 100–1600 m/z for metabolomics and lipidomics respectively.

#### Identification of metabolites

The pretreatment of the acquired raw data was performed which includes peak finding, alignment, filtering, and retention time correction by Markerview^TM^ software (Sciex Applied Biosystems) for the generation of the m/z features list with their intensities. Features having relative standard deviation (RSD) > 25% based on QC samples were excluded from the analysis. Metabolite identification was done based on m/z having mass accuracy (error<5 ppm), isotope pattern matching (error <10%) and MS/MS fragmentation matching (library score >70) by utilizing Accurate Mass Metabolite Spectral Library from Sciex which contains a highly curated collection of high-resolution accurate mass spectra. LIPIDMAPS® (www.lipidmaps.org), LipidBlast (www.fiehnlab.ucdavis.edu/projects/LipidBlast)^42^ and LipidView^TM^ (AB Sciex) software were used for the assignment of lipid class (delta < 0.002)^43^.

### Transcriptomics

#### Sample collection

For transcriptome sequencing, 1 mL of whole blood was drawn into PAXgenes^TM^ Blood RNA tubes (PreAnalyticX, Becton, Dickinson and Company), as per the manufacturer’s instruction. Briefly, blood samples were mixed instantly by inverting few times and were kept at room temperature for 4 hours and stored at −80 °C in PAXgenes^TM^ Blood RNA tubes until further analysis.

#### RNA extraction

Total RNA extraction from whole blood was done by using the PAXgene^TM^ Blood RNA Kit protocol followed by elution in nuclease-free water (20–30 μL). Assessment of RNA quality was performed by the RNA ScreenTape system (Agilent). The RNA integrity was ascertained by the RNA integrity number (RIN^e^). All the samples showed RIN^e^ > 7 (recommended minimum value for microarray analysis). Further, Qubit® 3.0 Fluorometer (ThermoFisher Scientific) was used to measure the RNA concentration.

#### RNA Library preparation

The RNA-seq experiment was conducted by utilizing the next-generation sequencing technologies to assess mRNA in blood 24 h after radiation exposure. The mRNA enrichment and library preparation were performed by NEBNext® Ultra™ II RNA Library Prep Kit for Illumina (New England BioLabs). Enriched mRNA was fragmented to achieve ~200 nucleotide-long inserts. Further, complementary DNA (cDNA) libraries were constructed, and the end was repaired following the manufacturer’s protocol. Amplification and purification were also performed for facilitating the multiplexing in sequencing. The library concentration was assayed by Qubit3 Fluorometer (Life Technologies) by utilizing the Qubit dsDNA High Sensitivity Assay Kit (Thermo Fisher Scientific) as per protocol whereas quality assessment of the cDNA library was measured by Agilent D1000 ScreenTape System in 4150 TapeStation System (Agilent).

#### Sequencing and processing

The library sequencing was performed using Illumina HiSeq. FastQC and MultiQC softwares were used for checking the data quality of sequencing. The processing of the raw sequence reads was performed using fastp to filter out adapter sequences along with low-quality bases. STAR v2 aligner was used for aligning the QC passed reads to the Mus musculus genome (GRCm38.90). On average, 99.91% of the reads were matched to the reference genome. Gene level expression value (read-count) of each gene was attained using feature-counts software.

### Statistical analysis

Normalization of the transcriptomic data was done using a trimmed mean of M (TMM) value. TMM is defined as the weighted trimmed mean of the log expression ratios. TMM are gene-wise log-fold change quantities originally defined by Robinson and Oshlack^44^. In order to compare differentially expressed genes (DEGs) among the samples, the TMM normalization approach is widely utilized to normalize total RNA output. Furthermore, PCA was performed on normalized read counts for visualizing differences between groups. Those features which did not have one count-per-million in at least three samples were removed. Differential gene expression analysis was performed using edgeR software (two-tailed *p*-value calculation using Wald statistics and Log2 fold change by maximum-likelihood estimate). DEGs were presented as genes having absolute log2 fold change ≥ 2 and adj. *p*-value ≤ 0.05. Normalization by the total area, log-transformation and pareto-scaling was done on the metabolomic and lipidomic dataset using MetaboAnalyst5.0 (www.metaboanalyst.ca). In addition to this, univariate analysis (two-tailed student’s *t* test and fold change) was also performed using MetaboAnalyst software. Identified metabolite/lipids having *p*-value < 0.05 were considered significant.

### Pathway analysis

#### Gene Ontology and pathway analysis for gene set

KEGG pathway, as well GO (http://geneontology.org/) focusing on BP, MF, and CC, were conducted using the clusterProfiler R package for determining functions of the DEGs. Pathways and GO having adj. *p*-value ≤ 0.05 were regarded as significant. GOplot R package and Pathview package were used for GO pathway visualization.

#### Pathway analysis for metabolomics

Metabolic pathway enrichment was done through KEGG (http://www.kegg.jp/). Pathway analysis for metabolites was performed using MetaboAnalyst whereas BioPAN software (www.lipidmaps.org/biopan) was used for analyzing lipid pathways. Metabolic pathways having a *p*-value < 0.05 and impact >0.2 were regarded as significant. For lipid pathways, we have taken the most active and most suppressed lipid reaction as significant (*p*-value < 0.05 equivalent to Z-score >1.645). The BioPAN software workflow utilizes Z-score, which takes into account both the mean as well as the standard deviation assuming a normally distributed data of lipid subclasses. Z-score was calculated as detailed in Gaud et al.^45^.

### Joint-Pathway enrichment for Integration of gene and metabolomics data set

For differential networking, integrated transcriptomic and metabolic analysis was carried out using the Joint-Pathway Analysis module of MetaboAnalyst software (www.metaboanalyst.ca). Both metabolic (*p*-value < 0.05) and transcriptomic (FDR < 0.05, fold change >2) datasets were used for Joint-Pathway Analysis. For that, official gene symbols of mRNA (or DEGs) and compound names of metabolites along with their corresponding log2Fold Change were uploaded for assessing the potential importance of individual molecules within a network. In the Joint Pathway Analysis module, various selected parameters were (i) in pathway database- metabolic pathways (integrated); and (ii) in algorithm selection: enrichment analysis- hypergeometric test, topology measure-degree centrality, integration method- combine queries^46^. Pathways having a *p*-value < 0.05 with impact >0.2 were considered significant. The pathway impact score summarizes the normalized topological measure of altered genes or metabolites present in each metabolic pathway whereas, the −log 10(*p*) value shows enrichment analysis results^47^.

Furthermore, the interaction network between the key metabolites and DEGs involved in the Joint-Pathway Analysis was also established using STITCH - an online webtool for visualizing biological relationships between metabolites-genes, metabolites-metabolites and genes-genes (www.stitch.embl.de)^48,49^. The subgraph generated from STITCH is bipartite due to the existence of edges only between proteins and chemicals. The obtained interactions were then constructed by Cytoscape software (3.9.1) to create the final differential network image.

### Reporting summary

Further information on research design is available in the Nature Research Reporting Summary linked to this article.

### Supplementary information

Supplementary Material

Reporting Summary

## Data Availability

The RNA-seq are deposited in a publicly accessible database (Sequence Read Archive (SRA)) with accession code PRJNA1005932. The other datasets generated during and/or analyzed during the current study are available from the corresponding author upon reasonable request.
