# Supplementary material for: Metabolomics and transcriptomics based multi-omics integration reveals radiation-induced altered pathway networking and underlying mechanism
Source: NPJ Syst Biol Appl. 2023 Sep 9;9:42. doi: 10.1038/s41540-023-00305-5 (PMC10492812; doi:10.1038/s41540-023-00305-5)
Supplement: Supplementary file 1 — Supplementary Material [file 41540_2023_305_MOESM1_ESM.pdf]

Metabolomics and Transcriptomics based Multi-Omics Integration Reveals Radiation-Induced altered pathway networking and underlying mechanism

Kiran Maan[1, 2], Ruchi Baghel[1], Seema Dhariwal[1], Apoorva Sharma[1], Radhika Bakhshi[2], Poonam Rana[1]*

[1]Metabolomics Research Facility, Institute of Nuclear Medicine and Allied Sciences (INMAS), DRDO, Delhi, India.

[2]Department of Biomedical Science, Shaheed Rajguru College of Applied Sciences for Women, University of Delhi, Delhi, India.

*Corresponding Author:

Dr. Poonam Rana, Ph.D. Metabolomics Research Facility

Institute of Nuclear Medicine and Allied Sciences (INMAS), DRDO

S. K Mazumdar Road, Timarpur, Delhi-54, India Tel: 91-11-23905319

Email address: poonam.inmas@gov.in

TABLE OF CONTENT

Supplementary Fig. 1: The mean quality value across each base position in the read generated by MultiQC.

Supplementary Fig. 2: Principal component analysis (PCA) of metabolomics (A), lipidomics (B) in both ionization modes using LC-MS and transcriptomics profiles (C) in mice blood at 24 hrs post irradiation.

Supplementary Fig. 3: GO enrichment of differentially expressed genes in LD (A) and HD (B) group.

Supplementary Fig. 4: Dysregulated "Hematopoietic cell lineage" by KEGG pathway analysis of DEGs.

Supplementary Fig. 5: Joint-Pathway Analysis of significantly altered transcriptomic and metabolomic data after integration when LD and HD groups were compared.

Pathways having pathway impact > 0.2 and –log 10(p) >1.3 (cutoff is highlighted in red dotted lines) were considered.

(Enrichment analysis by hypergeometric test and topology measure by degree centrality).

(B) Lipid network generated using BioPAN software representing active or suppressed lipid pathways in HD vs LD groups respectively.

Tables

Supplementary Table 1: Total RNA QC status of all the samples.

Supplementary Table 2: List of differentially expressed genes in LD group (A) and HD group (B) using edgeR software (two-tailed p-value calculation using Wald statistics and Log2 fold change by maximum-likelihood estimate).

Supplementary Table 3: GO analysis of differentially expressed genes (DEGs) in the biological process (BP), molecular function (MF) and cellular component (CC) in LD group (A) and HD group (B) using the clusterProfiler R package for determining functions of the DEGs based on BH adjusted p-values (adjusted p-value for multiple testing calculated using Benjamin-Hochberg correction method to examine the significance of pathway–term enrichment).

Supplementary Table 4: KEGG pathway analysis of differentially expressed genes in LD group (A) and HD group (B) using the clusterProfiler R package for determining functions of the DEGs based on BH adjusted p-values (adjusted p-value for multiple testing calculated using Benjamin-Hochberg correction method).

Supplementary Table 5: Identified metabolites/ lipids list with their error in Da.

Supplementary Table 6: List of significantly changed metabolites and lipids in LD group and HD group using univariate analysis (two tailed student's t test).

Supplementary Table 7: Pathway analysis of dysregulated metabolites in LD group (A) and HD group (B) compared to Control group (p values shows pathway enrichment analysis based on Global Test and pathway impact values shows the pathway topology analysis).

Supplementary Table 8: Differentially expressed genes common in both radiation groups. (Two-tailed p-value calculation using Wald statistics and Log2 fold change by maximum-likelihood estimate).

Supplementary Figures

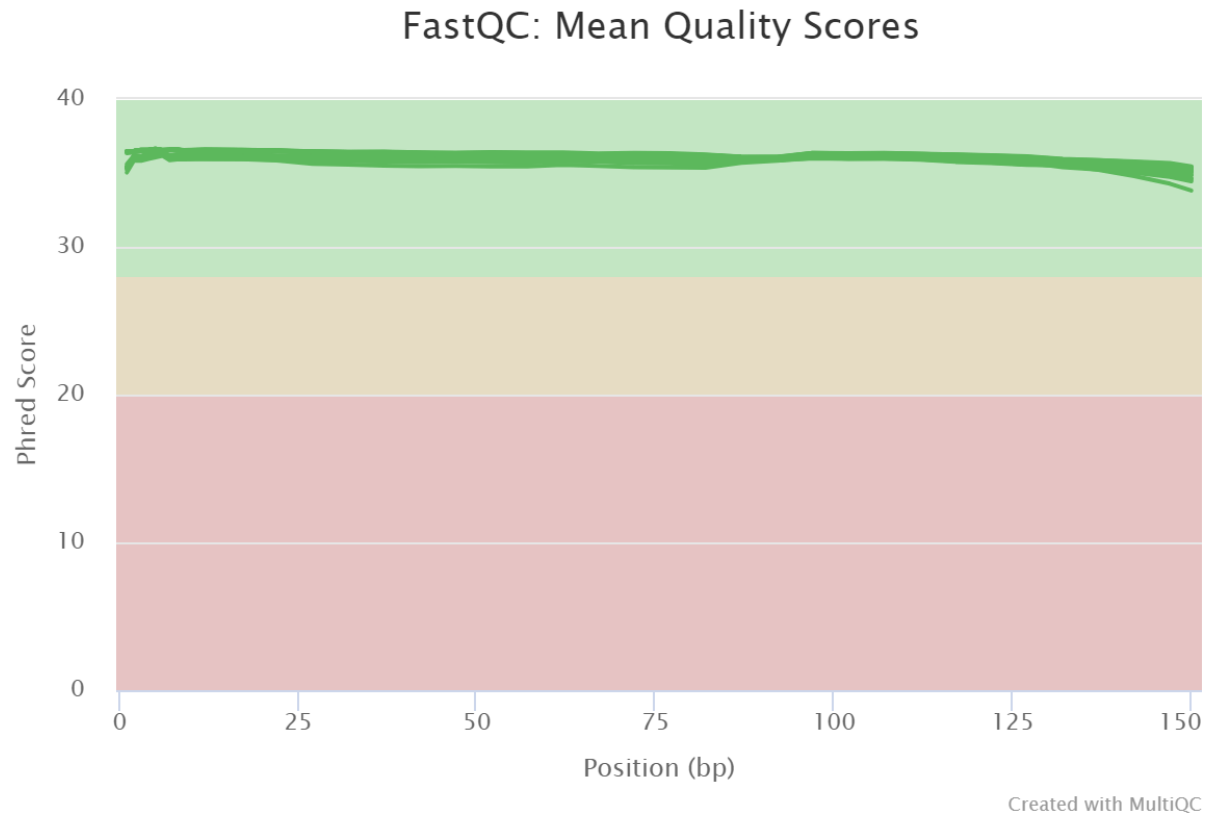

Supplementary Fig. 1: The mean quality value across each base position in the read generated by MultiQC.

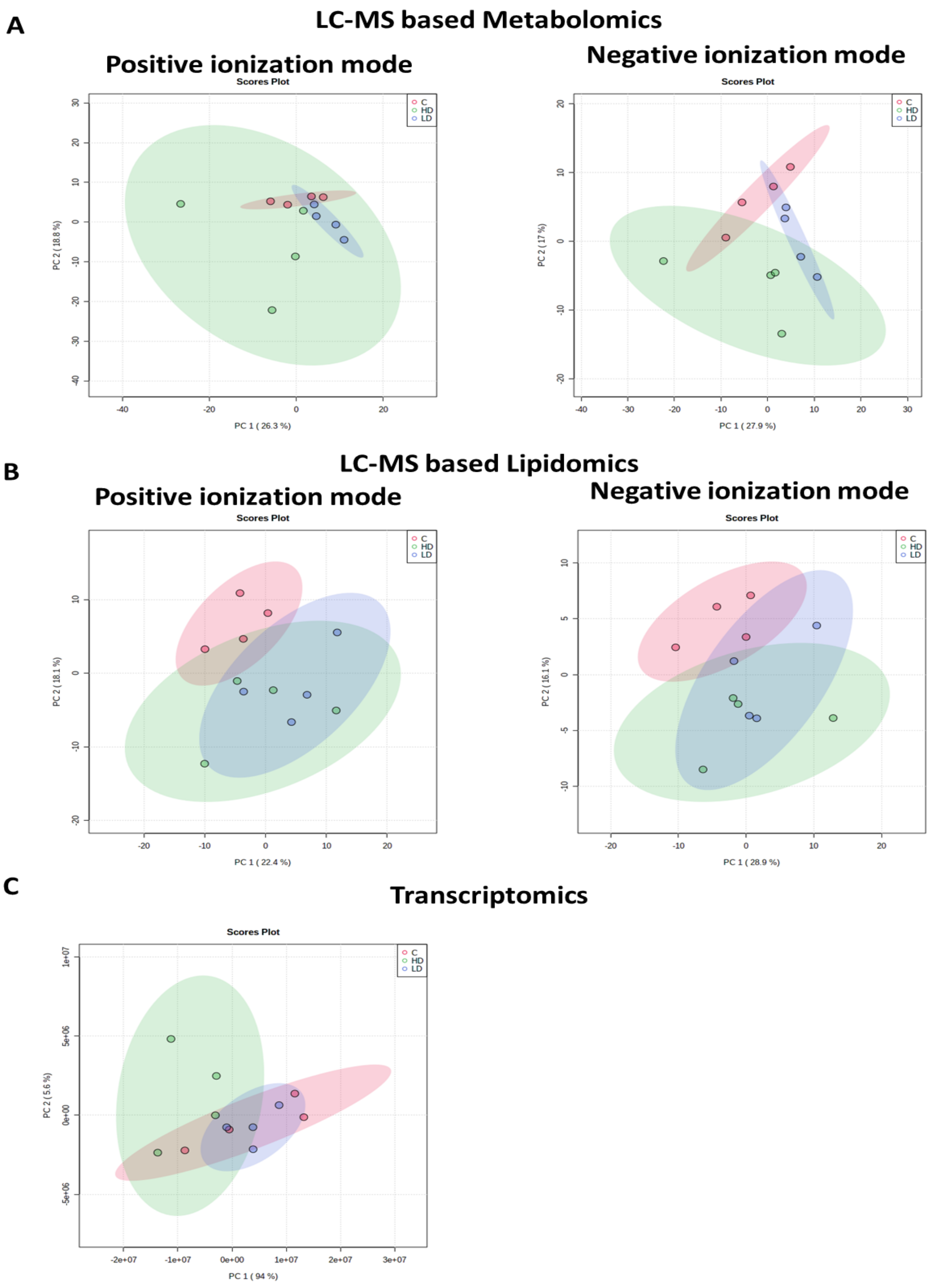

Supplementary Fig. 2: Principal component analysis (PCA) of metabolomics (A), lipidomics (B) in both ionization modes using LC-MS and transcriptomics profiles (C) in mice blood at 24 hrs post irradiation.

A

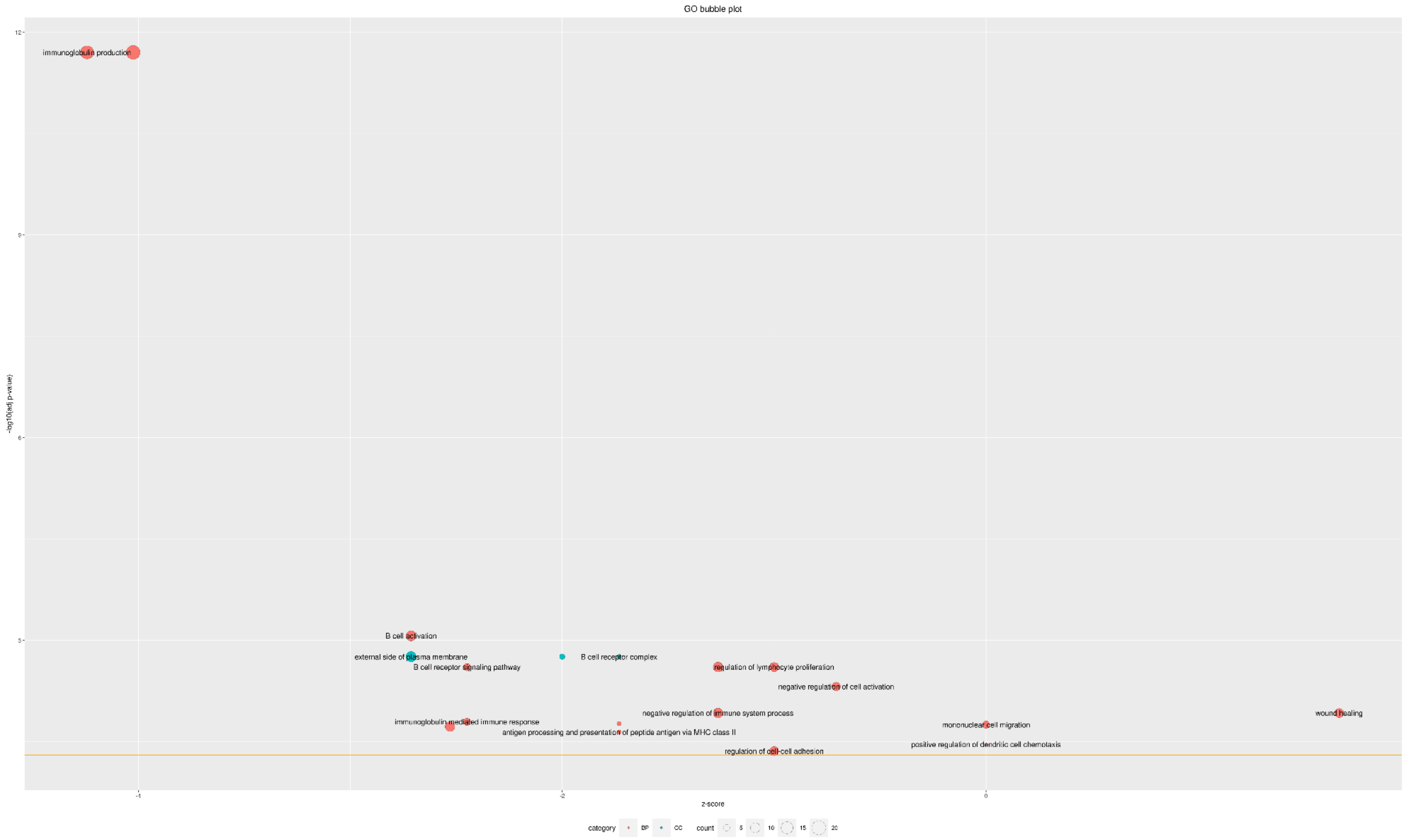
GO bubble plot
-log10(adj p-value)
12
9
6
3
immunoglobulin production
B cell activation
external side of plasma membrane
B cell receptor signaling pathway
B cell receptor complex
regulation of lymphocyte proliferation
negative regulation of cell activation
negative regulation of immune system process
immunoglobulin mediated immune response
antigen processing and presentation of peptide antigen via MHC class II
mononuclear cell migration
wound healing
positive regulation of dendritic cell chemotaxis
regulation of cell-cell adhesion
-4
-2
0
z-score
category
BP
CC
count
5
10
15
20

B:

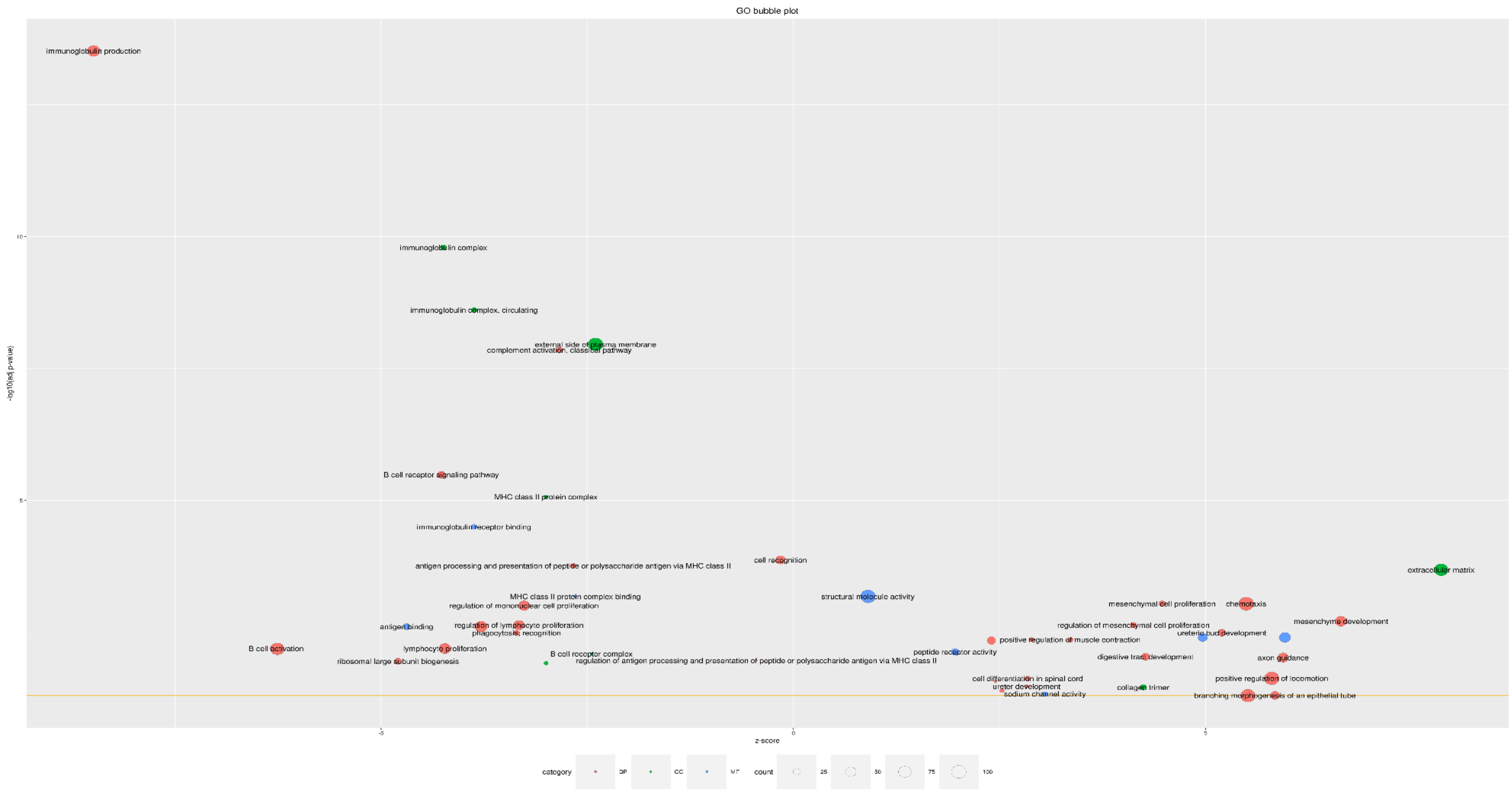

Supplementary Fig. 3: GO enrichment of differentially expressed genes in LD (A) and HD (B) group.

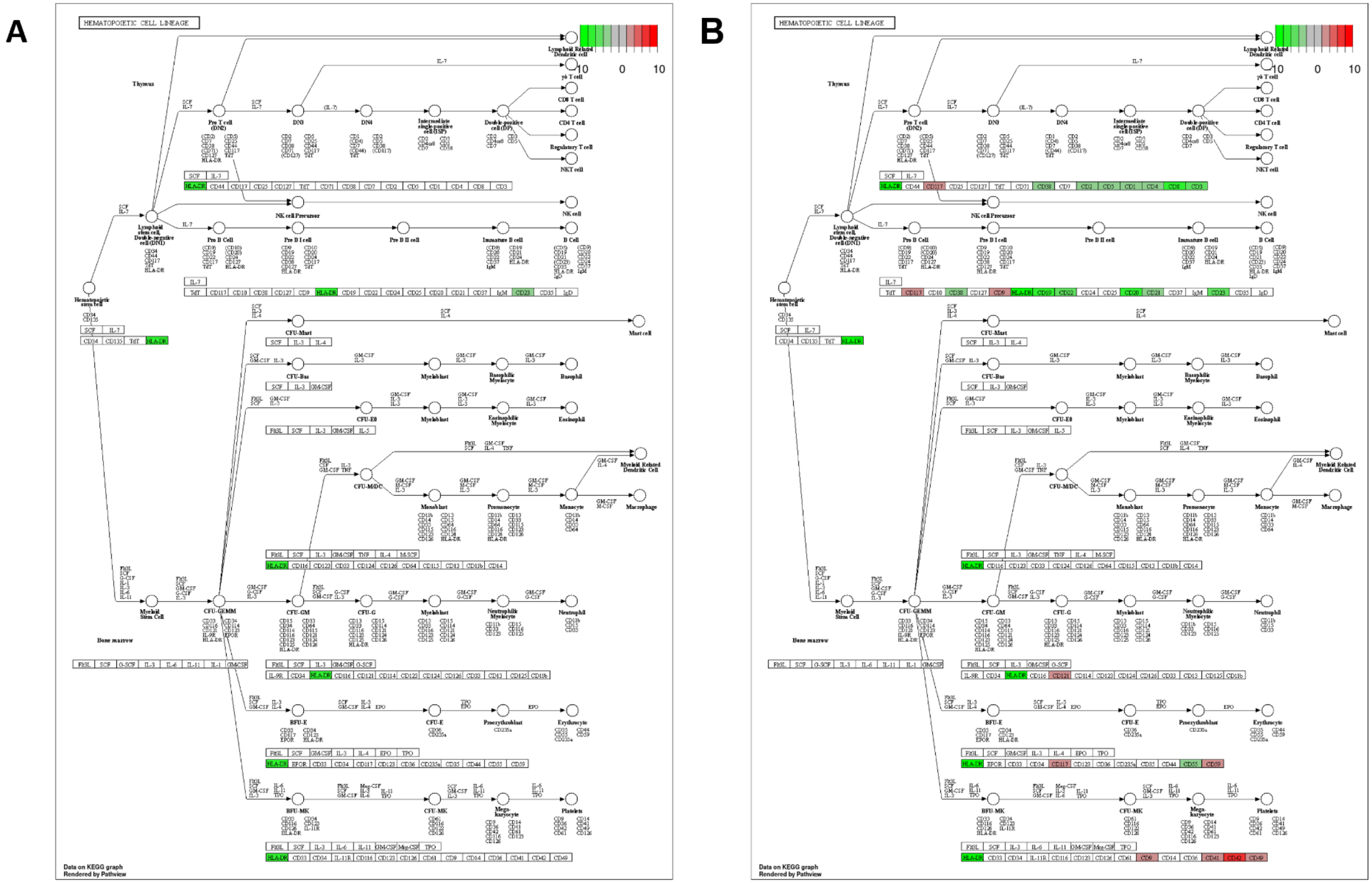

Supplementary Fig. 4: Dysregulated “Hematopoietic cell lineage” by KEGG pathway analysis of DEGs.

A

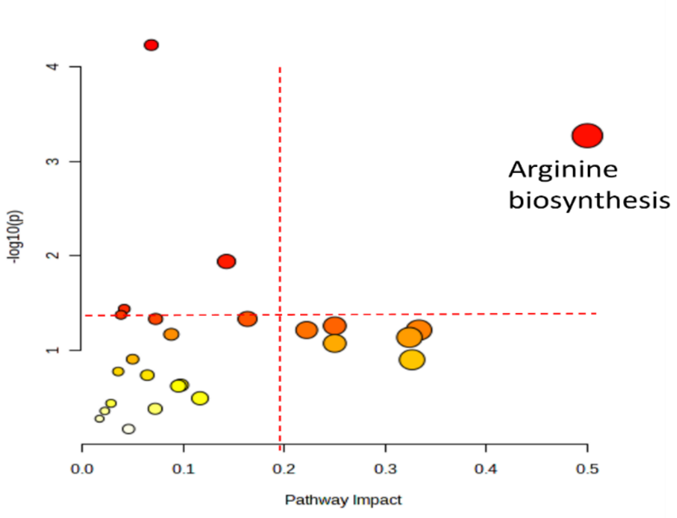

B

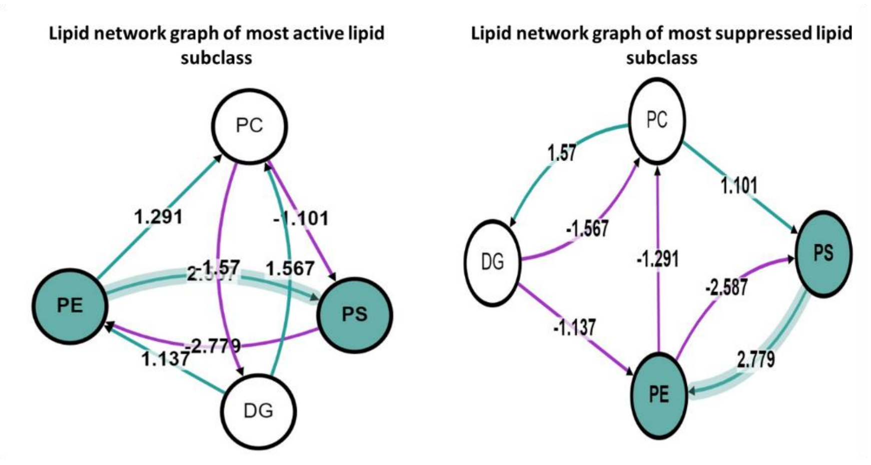

Supplementary Fig. 5: (A) Joint-Pathway Analysis of significantly altered transcriptomic and metabolomic data after integration when LD and HD groups were compared.

Pathways having pathway impact > 0.2 and –log 10(p) >1.3 (cutoff is highlighted in red dotted lines) were considered.
(Enrichment analysis by hypergeometric test and topology measure by degree centrality).

(B) Lipid network generated using BioPAN software representing active or suppressed lipid pathways in HD vs LD groups respectively.

Supplementary Table 1: Total RNA QC status of all the samples.

Sample Details	Tapestation QC		QUBIT Quantification			Results
Sample ID	RIN	28S/18S Ratio	Conc. (ng/ul)	Vol of RNA available (ul)	Total RNA mass (ng)	Test Result
C1	7.7	1.8	50.8	25	1270	Pass
C2	8.5	1.1	51.4	25	1285	Pass
C3	7.3	2.1	45	15	675	Pass
CT4	8.4	1	51.8	25	1295	Pass
HD1	7.9	2.1	18.7	25	467.5	Pass
HD2	8.3	1.2	30.6	25	765	Pass
HD3	8.4	1.1	37.8	25	945	Pass
HD4	7.8	1.5	22.2	15	333	Pass
LD1	7.6	1.2	20	15	300	Pass
LD2	9.7	1.5	35	25	875	Pass
LD3	9.2	1.9	32.8	25	820	Pass

Supplementary Table 2 (A): List of differentially expressed genes in LD group using edgeR software (two-tailed p-value calculation using Wald statistics and Log2 fold change by maximum-likelihood estimate).

**Column Names**	**Description**
**Id**	Gene Id
**Sample1**	Normalized expression value of Sample1
**Sample2**	Normalized expression value of Sample2
**baseMean**	The mean of normalized counts for all samples (normalized for sequencing depth)
**FoldChange**	fold change of expression, calculated as 2log2(FC)
**log2FoldChange**	Log2 fold change (MLE – maximum-likelihood estimate): the ratio between the mean of normalized counts of Control over Test.
**lfcSE**	Standard error estimate for the log2 fold change estimate.
**Stat**	Wald statistic (the log2FoldChange divided by lfcSE, which is compared to a standard Normal distribution to generate a two-tailed pvalue)
**pvalue**	Wald test p-value.
**Padj**	BH adjusted p-values (adjusted p-value for multiple testing calculated using Benjamin-Hochberg correction method)
**gene_name**	Gene symbol
**gene_description**	Gene Description
**gene_biotype**	Gene Biotype
**ENTREZID**	Entrez Gene Id
**TF.Family**	Transcription Factor Family

**Control vs LD**										
**Id**	**Base Mean**	**FC**	**log2Fold Change**	**pvalue**	**padj**	**gene_ name**	**gene_description**	**gene_biotype**	**Entrez_id**	**TF_ Family**
ENSMUSG00000000204	1611.69	5.529	2.467	3.01475E-05	0.004095172	Slfn4	schlafen 4	protein_coding	20558	-
ENSMUSG00000000903	36.36	0.198	-2.338	2.8913E-06	0.001138169	Vpreb3	pre-B lymphocyte gene 3	protein_coding	22364	-
ENSMUSG00000001027	35.44	0.188	-2.414	3.53847E-07	0.000310243	Scn4a	sodium channel, voltage-gated, type IV, alpha	protein_coding	110880	-
ENSMUSG00000002007	26.93	0.201	-2.313	1.21856E-05	0.002527398	Srpk3	serine/arginine-rich protein specific kinase 3	protein_coding	56504	-
ENSMUSG00000002249	3.62	21.556	4.43	0.000321116	0.020785271	Tead3	TEA domain family member 3	protein_coding	21678	TEA
ENSMUSG00000003379	838.5	0.247	-2.016	2.11019E-06	0.000969132	Cd79a	CD79A antigen (immunoglobulin-associated alpha)	protein_coding	12518	-
ENSMUSG00000003559	13.49	4.068	2.024	0.000341911	0.021763634	As3mt	arsenic (+3 oxidation state) methyltransferase	protein_coding	57344	-

ENSMUSG00000005540	148.38	0.199	-2.327	1.28322E-07	0.000165013	Fcer2a	Fc receptor, IgE, low affinity II, alpha polypeptide	protein_coding	14128	-
ENSMUSG00000014453	151.63	0.202	-2.307	3.60188E-11	2.31589E-07	Blk	B lymphoid kinase	protein_coding	12143	-
ENSMUSG00000015316	334.24	4.122	2.043	0.00011489	0.010355659	Slamf1	signaling lymphocytic activation molecule family member 1	protein_coding	27218	-
ENSMUSG00000017009	33.35	0.209	-2.261	9.49285E-06	0.002317817	Sdc4	syndecan 4	protein_coding	20971	-
ENSMUSG00000017400	21.82	0.155	-2.687	2.46073E-05	0.003618843	Stac2	SH3 and cysteine rich domain 2	protein_coding	217154	-
ENSMUSG00000017999	136.78	4.455	2.156	0.000526542	0.029439031	Ddx27	DEAD (Asp-Glu-Ala-Asp) box polypeptide 27	protein_coding	228889	-
ENSMUSG00000018199	77.96	4.621	2.208	1.93529E-05	0.003218094	Trove2	TROVE domain family, member 2	protein_coding	20822	-
ENSMUSG00000019851	4.66	15.215	3.927	0.000521937	0.029266402	Perp	PERP, TP53 apoptosis effector	protein_coding	64058	-
ENSMUSG00000022504	155.64	0.168	-2.569	2.47504E-11	2.31589E-07	Ciita	class II transactivator	protein_coding	12265	-
ENSMUSG00000022661	23.52	0.225	-2.153	0.000465378	0.026957003	Cd200	CD200 antigen	protein_coding	17470	-
ENSMUSG00000022803	35.05	4.777	2.256	6.06909E-06	0.001833855	Popdc2	popeye domain containing 2	protein_coding	64082	-
ENSMUSG00000023067	46.09	4.615	2.206	0.000682333	0.034274777	Cdkn1a	cyclin-dependent kinase inhibitor 1A (P21)	protein_coding	12575	-
ENSMUSG00000024076	142.45	0.018	-5.769	6.43616E-05	0.006974556	Vit	vitrin	protein_coding	74199	-
ENSMUSG00000024298	15.69	0.208	-2.264	0.001050439	0.044047636	Zfp871	zinc finger protein 871	protein_coding	208292	zf-C2H2
ENSMUSG00000024610	2580.77	0.239	-2.064	1.29498E-05	0.002619968	Cd74	CD74 antigen (invariant polypeptide of major histocompatibility complex, class II antigen-associated)	protein_coding	16149	-
ENSMUSG00000025196	6.02	10.171	3.346	0.000922113	0.040888836	Cpn1	carboxypeptidase N, polypeptide 1	protein_coding	93721	-
ENSMUSG00000025856	65.18	4.629	2.211	4.93308E-08	7.92952E-05	Pdgfa	platelet derived growth factor, alpha	protein_coding	18590	-
ENSMUSG00000026181	151.86	0.08	-3.647	0.000101166	0.00951894	Ppm1f	protein phosphatase 1F (PP2C domain containing)	protein_coding	68606	-
ENSMUSG00000026357	1234.82	5.105	2.352	9.10367E-05	0.008831609	Rgs18	regulator of G-protein signaling 18	protein_coding	64214	-
ENSMUSG00000026837	57.56	6.328	2.662	0.000619833	0.032666571	Col5a1	collagen, type V, alpha 1	protein_coding	12831	-
ENSMUSG00000026976	108.73	0.017	-5.903	0.00115039	0.0464057	Pax8	paired box 8	protein_coding	18510	PAX
ENSMUSG00000026986	10.89	5.128	2.358	0.000388878	0.02388873	Hnmt	histamine N-methyltransferase	protein_coding	140483	-
ENSMUSG00000027014	66.9	0.181	-2.468	0.001215641	0.047778322	Cwc22	CWC22 spliceosome-associated protein	protein_coding	80744	-
ENSMUSG00000027690	5.79	10.536	3.397	0.000130823	0.011366851	Slc2a2	solute carrier family 2 (facilitated glucose transporter), member 2	protein_coding	20526	-

ENSMUSG00000027855	7.32	5.855	2.55	0.000677731	0.034174367	Sycp1	synaptonemal complex protein 1	protein_coding	20957	-
ENSMUSG00000028524	78.93	4.674	2.225	3.97953E-06	0.001421501	Sgip1	SH3-domain GRB2-like (endophilin) interacting protein 1	protein_coding	73094	-
ENSMUSG00000030787	5.02	14.239	3.832	5.63824E-05	0.006431565	Lyve1	lymphatic vessel endothelial hyaluronan receptor 1	protein_coding	114332	-
ENSMUSG00000031425	116.98	4.853	2.279	2.47556E-06	0.001041053	Plp1	proteolipid protein (myelin) 1	protein_coding	18823	-
ENSMUSG00000031647	66.39	6.472	2.694	6.03898E-11	2.91215E-07	Mfap3l	microfibrillar-associated protein 3-like	protein_coding	71306	-
ENSMUSG00000031842	11.39	0.153	-2.706	0.000458006	0.026690282	Pde4c	phosphodiesterase 4C, cAMP specific	protein_coding	110385	-
ENSMUSG00000032261	602.31	4.126	2.045	2.43228E-05	0.003618843	Sh3bgrl2	SH3 domain binding glutamic acid-rich protein like 2	protein_coding	212531	-
ENSMUSG00000037071	144.77	0.228	-2.134	6.49026E-09	1.32396E-05	Scd1	stearoyl-Coenzyme A desaturase 1	protein_coding	20249	-
ENSMUSG00000038055	245.19	0.242	-2.047	2.66169E-07	0.000256706	Dexi	dexamethasone-induced transcript	protein_coding	58239	-
ENSMUSG00000038059	436.68	4.178	2.063	9.97354E-06	0.002346092	Smim3	small integral membrane protein 3	protein_coding	106878	-
ENSMUSG00000038235	47.41	4.042	2.015	2.55667E-05	0.003680267	F11r	F11 receptor	protein_coding	16456	-
ENSMUSG00000038331	37.16	5.352	2.42	0.001133072	0.046012283	Satb2	special AT-rich sequence binding protein 2	protein_coding	212712	CUT
ENSMUSG00000038421	131.28	0.25	-2.001	6.86383E-09	1.32396E-05	Fcrla	Fc receptor-like A	protein_coding	98752	-
ENSMUSG00000038775	7.36	6.529	2.707	0.000548102	0.03020159	Vill	villin-like	protein_coding	22351	-
ENSMUSG00000040447	38.13	0.2	-2.319	1.86816E-05	0.003133479	Spns2	spinster homolog 2	protein_coding	216892	-
ENSMUSG00000040592	467.69	0.245	-2.03	1.62155E-07	0.000195488	Cd79b	CD79B antigen	protein_coding	15985	-
ENSMUSG00000040899	24.02	0.178	-2.49	1.73005E-06	0.000813924	Ccr6	chemokine (C-C motif) receptor 6	protein_coding	12458	-
ENSMUSG00000041538	279.79	0.188	-2.412	1.171E-09	3.22677E-06	H2-Ob	histocompatibility 2, O region beta locus	protein_coding	15002	-
ENSMUSG00000041670	48.27	4.185	2.065	6.4545E-06	0.001834401	Rims1	regulating synaptic membrane exocytosis 1	protein_coding	116837	-
ENSMUSG00000042474	177.91	0.22	-2.185	4.37832E-08	7.67758E-05	Fcmr	Fc fragment of IgM receptor	protein_coding	69169	-
ENSMUSG00000042734	50.77	0.082	-3.605	2.19798E-05	0.003419104	Ttc9	tetratricopeptide repeat domain 9	protein_coding	69480	-
ENSMUSG00000045613	9.29	7.519	2.91	0.000435875	0.026191894	Chrm2	cholinergic receptor, muscarinic 2, cardiac	protein_coding	243764	-
ENSMUSG00000046580	6.88	0.074	-3.766	7.79457E-05	0.008120205	Gm7862	-	processed_pseudogene	-	-
ENSMUSG00000046637	4.68	19.503	4.286	6.3468E-05	0.006929899	Ttc34	tetratricopeptide repeat domain 34	protein_coding	242800	-

ENSMUSG00000047250	45.61	5.024	2.329	6.61418E-07	0.000472522	Ptgs1	prostaglandin-endoperoxide synthase 1	protein_coding	19224	-
ENSMUSG00000048965	10.62	0.109	-3.204	0.000320043	0.020785271	Mrgpre	MAS-related GPR, member E	protein_coding	244238	-
ENSMUSG00000049001	6.24	6.822	2.77	0.000980489	0.042310195	Ndnf	neuron-derived neurotrophic factor	protein_coding	68169	-
ENSMUSG00000050345	125.91	4.529	2.179	1.42978E-05	0.002730589	4930486L24Rik	RIKEN cDNA 4930486L24 gene	protein_coding	214639	-
ENSMUSG00000050394	13.68	4.062	2.022	0.001222308	0.047920946	Armcx6	armadillo repeat containing, X-linked 6	protein_coding	278097	-
ENSMUSG00000051855	310.9	4.619	2.208	1.12398E-05	0.002492009	Mest	mesoderm specific transcript	protein_coding	17294	-
ENSMUSG00000052188	4.11	0.018	-5.784	0.000733354	0.035631402	Gm14964	-	antisense_RNA	-	-
ENSMUSG00000052270	44.38	4.328	2.114	5.50539E-06	0.001712797	Fpr2	formyl peptide receptor 2	protein_coding	14289	-
ENSMUSG00000053684	14.77	0.146	-2.777	9.53957E-05	0.009065447	BC048403	cDNA sequence BC048403	protein_coding	270802	-
ENSMUSG00000053965	30.2	4.106	2.038	2.71875E-06	0.00109254	Pde5a	phosphodiesterase 5A, cGMP-specific	protein_coding	242202	-
ENSMUSG00000054976	18.51	4.032	2.011	0.000295464	0.019780643	Nyap2	neuronal tyrosine-phophorylated phosphoinositide 3-kinase adaptor 2	protein_coding	241134	-
ENSMUSG00000055745	13.96	0.172	-2.542	0.001197174	0.047778322	Ldoc1l	retrotransposon Gag like 6	protein_coding	223732	-
ENSMUSG00000055963	24.25	6.521	2.705	6.20648E-05	0.006880278	Triqk	triple QxxK/R motif containing	protein_coding	208820	-
ENSMUSG00000056004	22.21	4.072	2.026	0.001208279	0.047778322	9330182L06Rik	RIKEN cDNA 9330182L06 gene	protein_coding	231014	-
ENSMUSG00000056025	11.87	6.039	2.594	0.000170504	0.01345527	Clca3a1	chloride channel accessory 3A1	protein_coding	12722	-
ENSMUSG00000056492	30.07	14.338	3.842	4.11515E-05	0.005088095	Adgrf5	adhesion G protein-coupled receptor F5	protein_coding	224792	-
ENSMUSG00000057337	193.24	0.191	-2.387	9.4182E-10	3.0278E-06	Chst3	carbohydrate sulfotransferase 3	protein_coding	53374	-
ENSMUSG00000058626	79.62	5.182	2.374	4.49178E-06	0.001507439	Capn11	calpain 11	protein_coding	268958	-
ENSMUSG00000060594	26.63	6.233	2.64	1.06919E-05	0.002426299	Layn	layilin	protein_coding	244864	-
ENSMUSG00000061132	90.24	0.243	-2.044	1.13152E-07	0.000155899	Blnk	B cell linker	protein_coding	17060	-
ENSMUSG00000062345	19.01	4.665	2.222	9.5406E-05	0.009065447	Serpinb2	serine (or cysteine) peptidase inhibitor, clade B, member 2	protein_coding	18788	-
ENSMUSG00000064337	23511.22	4.903	2.294	0.000240095	0.016658932	mt-Rnr1	-	Mt_rRNA	-	-
ENSMUSG00000066838	6.01	0.056	-4.146	0.000375481	0.023288259	Zfp772	zinc finger protein 772	protein_coding	232855	zf-C2H2
ENSMUSG00000067341	52.43	0.214	-2.227	1.7475E-05	0.003036721	H2-Eb2	histocompatibility 2, class II antigen E beta2	protein_coding	381091	-

ENSMUSG00000068105	89.02	0.171	-2.551	3.40657E-11	2.31589E-07	Tnfrsf13c	tumor necrosis factor receptor superfamily, member 13c	protein_coding	72049	-
ENSMUSG00000069306	116.25	4.169	2.06	6.67319E-06	0.001865496	Hist1h4m	histone cluster 1, H4m	protein_coding	100041230	-
ENSMUSG00000071745	3.42	63.701	5.993	2.66102E-05	0.0037466	DXBay18	DNA segment, Chr X, Baylor 18	protein_coding	574405	-
ENSMUSG00000073421	1081.55	0.182	-2.459	4.90557E-07	0.000394265	H2-Ab1	histocompatibility 2, class II antigen A, beta 1	protein_coding	14961	-
ENSMUSG00000076501	12.26	0.174	-2.523	0.001071348	0.044496936	Igkv2-137	-	IG_V_gene	-	-
ENSMUSG00000076508	16.48	0.173	-2.534	8.30572E-05	0.008470694	Igkv17-127	-	IG_V_gene	-	-
ENSMUSG00000076538	12.27	0.081	-3.635	1.10848E-05	0.002486228	Igkv13-84	-	IG_V_gene	-	-
ENSMUSG00000076540	7.48	0.125	-3.001	0.001057987	0.04424113	Igkv4-80	-	IG_V_gene	-	-
ENSMUSG00000076545	17.89	0.164	-2.604	0.000476691	0.027529615	Igkv4-72	-	IG_V_gene	-	-
ENSMUSG00000076550	11.48	0.094	-3.407	4.31733E-05	0.005233308	Igkv4-63	-	IG_V_gene	-	-
ENSMUSG00000076618	27.26	0.23	-2.117	0.000347061	0.021949018	Ighj4	-	IG_J_gene	-	-
ENSMUSG00000076621	21.94	0.233	-2.104	9.11136E-05	0.008831609	Ighj1	-	IG_J_gene	-	-
ENSMUSG00000076646	3.7	0.02	-5.644	0.000762226	0.03621324	Ighv2-6-8	-	IG_V_gene	-	-
ENSMUSG00000076870	13.35	7.52	2.911	0.000984191	0.042375126	Trdj1	-	TR_J_gene	-	-
ENSMUSG00000076934	18.64	0.22	-2.184	0.00091051	0.040560809	Iglv1	-	IG_V_gene	-	-
ENSMUSG00000078810	144.85	4.546	2.185	9.48805E-07	0.000560002	Gp6	glycoprotein 6 (platelet)	protein_coding	243816	-
ENSMUSG00000079001	4.85	8.526	3.092	0.001076324	0.044511349	4930404H24Rik	-	protein_coding	-	-
ENSMUSG00000079543	12.12	0.122	-3.03	0.00021835	0.015833638	Igkv13-85	-	IG_V_gene	-	-
ENSMUSG00000080969	8.28	0.009	-6.852	9.04473E-07	0.000560002	Gm12372	-	processed_pseudogene	-	-
ENSMUSG00000082141	7.13	0.138	-2.856	0.000499055	0.028312556	Gm11212	-	unprocessed_pseudogene	-	-
ENSMUSG00000084893	85.91	5.007	2.324	5.44724E-05	0.006254278	Hba-ps4	-	unprocessed_pseudogene	-	-
ENSMUSG00000084970	21.49	6.054	2.598	2.26772E-05	0.003499361	1700060J05Rik	-	antisense_RNA	-	-
ENSMUSG00000085971	22.95	0.229	-2.124	0.000109663	0.010025102	Gm15411	-	processed_transcript	-	-
ENSMUSG00000086146	21.98	5.787	2.533	3.83052E-05	0.004860986	Gm15729	-	antisense_RNA	-	-

ENSMUSG00000086199	61.32	4.839	2.275	0.000688497	0.034366479	Bcas3os1	breast carcinoma amplified sequence 3, opposite strand 1	antisense_RNA	71489	-
ENSMUSG00000086245	52.64	0.22	-2.183	1.05879E-06	0.000600676	Gm16170	-	antisense_RNA	-	-
ENSMUSG00000086425	54.88	10.689	3.418	0.000750722	0.035843242	F730016J06Rik	-	lincRNA	-	-
ENSMUSG00000087626	24.2	4.869	2.284	0.000448221	0.026513092	Gm15050	predicted gene 15050	antisense_RNA	100504154	-
ENSMUSG00000087684	3.44	14.071	3.815	0.000643196	0.03343592	1200007C13Rik	-	lincRNA	-	-
ENSMUSG00000089656	8.37	7.19	2.846	0.000145013	0.012175024	Gm16271	-	antisense_RNA	-	-
ENSMUSG00000092035	44.31	4.042	2.015	0.000264626	0.018124109	Peg10	paternally expressed 10	protein_coding	170676	-
ENSMUSG00000093327	379.72	0.201	-2.317	6.30401E-09	1.32396E-05	Mir5107	microRNA 5107	miRNA	100628611	-
ENSMUSG00000093598	12.1	6.164	2.624	9.3933E-05	0.0090452	A730085K08Rik	-	antisense_RNA	-	-
ENSMUSG00000094117	15.41	0.219	-2.191	0.000799667	0.037621407	Igkv3-12	-	IG_V_gene	-	-
ENSMUSG00000094124	6.01	0.088	-3.506	0.001253274	0.049035279	Ighv1-74	-	IG_V_gene	-	-
ENSMUSG00000094157	28.75	0.031	-4.993	0.000973709	0.042206463	Gm7995	predicted gene 7995	protein_coding	666233	-
ENSMUSG00000094198	3.55	0.021	-5.586	0.000994313	0.042715578	Ighv1-50	-	IG_V_gene	-	-
ENSMUSG00000094344	60.84	0.14	-2.832	0.000705448	0.034890728	Gm11942	-	processed_pseudogene	-	-
ENSMUSG00000094420	35.97	0.248	-2.009	6.17972E-06	0.001833855	Igkv10-96	-	IG_V_gene	-	-
ENSMUSG00000094561	5.18	0.014	-6.121	0.000201804	0.015029367	Ighv1-22	-	IG_V_gene	-	-
ENSMUSG00000094985	4.7	9.798	3.293	0.000834702	0.038662322	Topaz1	testis and ovary specific PAZ domain containing 1	protein_coding	671232	-
ENSMUSG00000095197	5.05	0.06	-4.064	0.000626154	0.032731416	Ighv1-59	-	IG_V_gene	-	-
ENSMUSG00000095204	3.41	0.02	-5.615	0.000875969	0.039719154	Ighv1-52	-	IG_V_gene	-	-
ENSMUSG00000095280	7.28	18.548	4.213	8.34378E-05	0.008470694	Gm21738	-	protein_coding	-	-
ENSMUSG00000095612	8.47	0.063	-3.983	0.000448792	0.026513092	Ighv5-4	-	IG_V_gene	-	-
ENSMUSG00000095771	16.43	0.177	-2.497	0.00016689	0.013302233	Igkv14-111	-	IG_V_gene	-	-
ENSMUSG00000096490	14.07	0.086	-3.539	7.10059E-06	0.001929061	Igkv10-94	-	IG_V_gene	-	-
ENSMUSG00000096515	5.54	0.051	-4.288	0.000457904	0.026690282	Igkv14-100	-	IG_V_gene	-	-

ENSMUSG00000096638	7.43	0.01	-6.679	9.70657E-06	0.002340375	Ighv2-9	-	IG_V_gene	-	-
ENSMUSG00000096715	22.14	0.225	-2.149	0.001184003	0.047382216	Igkv3-4	-	IG_V_gene	-	-
ENSMUSG00000097133	5.99	0.012	-6.382	0.00026497	0.018124109	Gm26628	-	antisense_RNA	-	-
ENSMUSG00000097174	5.06	0.061	-4.033	0.000755043	0.035960546	Gm4890	-	bidirectional_promoter_lncRNA	-	-
ENSMUSG00000098537	20.02	4.438	2.15	0.00073107	0.03561012	Gm27867	-	misc_RNA	-	-
ENSMUSG00000101308	243.96	4.071	2.025	4.53271E-06	0.001507439	Gm28989	-	antisense_RNA	-	-
ENSMUSG00000102817	8.13	7.215	2.851	0.000630154	0.032851473	Gm6185	-	transcribed_unprocessed_pseudogene	-	-
ENSMUSG00000104213	716.04	0.145	-2.784	2.01602E-10	7.7774E-07	Ighd	-	IG_C_gene	-	-
ENSMUSG00000105703	39.19	0.092	-3.436	0.000262231	0.018064938	Gm43305	-	processed_transcript	-	-
ENSMUSG00000105901	12.7	0.153	-2.707	0.000492598	0.028028679	Gm7291	-	processed_pseudogene	-	-
ENSMUSG00000107528	43.86	4.044	2.016	1.14069E-06	0.000611188	Gm44442	-	lincRNA	-	-
ENSMUSG00000108854	12.66	4.463	2.158	0.00105964	0.04424113	D830036C21Rik	-	lincRNA	-	-
ENSMUSG00000109799	33.95	0.195	-2.36	3.59147E-06	0.001332229	Gm45515	-	antisense_RNA	-	-
ENSMUSG00000110575	30.34	0.25	-2.002	0.001033619	0.043531618	6330537M06Rik	-	TEC	-	-
ENSMUSG00000110631	23.53	8.069	3.012	3.48394E-05	0.004540652	Gm42047	-	lincRNA	-	-
ENSMUSG00000112063	17.81	0.146	-2.774	4.34097E-05	0.005233308	AC161114.1	-	processed_pseudogene	-	-

Supplementary Table 2 (B): List of differentially expressed genes in HD group using edgeR software (two-tailed p-value calculation using Wald statistics and Log2 fold change by maximum-likelihood estimate).

**Control vs HD**										
**Id**	**baseMean**	**FC**	**log2Fold Change**	**pvalue**	**padj**	**gene_name**	**gene_description**	**gene_biotype**	**Entrez_id**	**TF_Family**
ENSMUSG00000000028	14.03	5.641	2.496	0.003170837	0.010748305	Cdc45	cell division cycle 45	protein_coding	12544	-
ENSMUSG00000000049	24.18	4.879	2.287	6.64828E-06	7.14492E-05	Apoh	apolipoprotein H	protein_coding	11818	-
ENSMUSG00000000127	341.15	4.798	2.262	4.76205E-08	1.2069E-06	Fer	fer (fms/fps related) protein kinase	protein_coding	14158	-
ENSMUSG00000000157	5.78	8.752	3.13	0.004757099	0.014921833	Itgb2l	integrin beta 2-like	protein_coding	16415	-
ENSMUSG00000000159	18.24	4.979	2.316	1.27306E-05	0.000122146	Igsf5	immunoglobulin superfamily, member 5	protein_coding	72058	-
ENSMUSG00000000202	5.31	6.237	2.641	0.003565792	0.011819539	Btbd17	BTB (POZ) domain containing 17	protein_coding	72014	-
ENSMUSG00000000204	1196.78	4.686	2.228	0.000117552	0.000741934	Slfn4	schlafen 4	protein_coding	20558	-
ENSMUSG00000000244	123.59	0.172	-2.543	5.98052E-13	9.42666E-11	Tspan32	tetraspanin 32	protein_coding	27027	-
ENSMUSG00000000266	10.85	5.893	2.559	0.000398178	0.001992996	Mid2	midline 2	protein_coding	23947	-
ENSMUSG00000000295	11.88	0.153	-2.711	0.000747289	0.003315562	Hddc2	HD domain containing 2	protein_coding	69692	-
ENSMUSG00000000320	966.48	5.292	2.404	2.78415E-08	7.85376E-07	Alox12	arachidonate 12-lipoxygenase	protein_coding	11684	-
ENSMUSG00000000385	7.18	4.426	2.146	0.007129196	0.020672023	Tmprss2	transmembrane protease, serine 2	protein_coding	50528	-
ENSMUSG00000000409	227.85	0.183	-2.454	1.31697E-08	4.28708E-07	Lck	lymphocyte protein tyrosine kinase	protein_coding	16818	-
ENSMUSG00000000567	3.74	9.781	3.29	0.000690838	0.003114318	Sox9	SRY (sex determining region Y)-box 9	protein_coding	20682	HMG
ENSMUSG00000000581	19.23	0.205	-2.286	7.10428E-05	0.000495104	C1d	C1D nuclear receptor co-repressor	protein_coding	57316	-
ENSMUSG00000000782	302.38	0.175	-2.514	7.23095E-11	5.52906E-09	Tcf7	transcription factor 7, T cell specific	protein_coding	21414	HMG
ENSMUSG00000000823	17.8	0.247	-2.018	0.000148437	0.000899649	Zfp512b	zinc finger protein 512B	protein_coding	269401	zf-C2H2
ENSMUSG00000000902	24.17	0.197	-2.342	4.26313E-06	5.01336E-05	Smarcb1	SWI/SNF related, matrix associated, actin dependent regulator of chromatin, subfamily b, member 1	protein_coding	20587	-
ENSMUSG00000000903	22.67	0.088	-3.512	2.22001E-11	2.01472E-09	Vpreb3	pre-B lymphocyte gene 3	protein_coding	22364	-
ENSMUSG00000001014	10.17	8.837	3.144	0.000102781	0.000668427	Icam4	intercellular adhesion molecule 4, Landsteiner-Wiener blood group	protein_coding	78369	-

ENSMUSG00000 001016	17.55	0.244	-2.034	0.000239662	0.001319792	Ilf2	interleukin enhancer binding factor 2	protein_coding	67781	-
ENSMUSG00000 001020	57.23	0.164	-2.608	6.33897E-09	2.37797E-07	S100a4	S100 calcium binding protein A4	protein_coding	20198	-
ENSMUSG00000 001027	23.97	0.145	-2.786	5.20712E-07	8.91112E-06	Scn4a	sodium channel, voltage-gated, type IV, alpha	protein_coding	110880	-
ENSMUSG00000 001029	47.14	0.138	-2.856	5.38619E-12	6.01146E-10	Icam2	intercellular adhesion molecule 2	protein_coding	15896	-
ENSMUSG00000 001056	30.99	0.183	-2.446	1.34902E-08	4.36768E-07	Nhp2	NHP2 ribonucleoprotein	protein_coding	52530	-
ENSMUSG00000 001100	18.12	0.189	-2.406	9.76717E-05	0.000641002	Poldip2	polymerase (DNA-directed), delta interacting protein 2	protein_coding	67811	-
ENSMUSG00000 001227	28.07	4.063	2.023	6.84387E-05	0.000479445	Sema6b	sema domain, transmembrane domain I, and cytoplasmic domai19aladin19izaorin) 6B	protein_coding	20359	-
ENSMUSG00000 001348	159.23	0.128	-2.968	7.22913E-18	3.09286E-15	Acp5	acid phosphatase 5, tartrate resistant	protein_coding	11433	-
ENSMUSG00000 001380	22.44	0.177	-2.496	6.36929E-07	1.04808E-05	Hars	histidyl-tRNA synthetase	protein_coding	15115	-
ENSMUSG00000 001415	79.42	0.245	-2.03	1.02297E-07	2.29198E-06	Smg5	Smg-5 homolog, nonsense mediated mRNA decay factor (C. elegans)	protein_coding	229512	-
ENSMUSG00000 001435	22.3	5.153	2.365	0.000277623	0.001484263	Col18a1	collagen, type XVIII, alpha 1	protein_coding	12822	-
ENSMUSG00000 001496	7.68	8.264	3.047	0.000492637	0.002366897	Nkx2-1	NK2 homeobox 1	protein_coding	21869	Homeobox
ENSMUSG00000 001506	6.93	4.573	2.193	0.001726444	0.00658116	Col1a1	collagen, type I, alpha 1	protein_coding	12842	-
ENSMUSG00000 001507	6.4	4.595	2.2	0.013145137	0.033590012	Itga3	integrin alpha 3	protein_coding	16400	-
ENSMUSG00000 001518	8.56	0.034	-4.861	1.25573E-07	2.68302E-06	Itfg2	integrin alpha FG-GAP repeat containing 2	protein_coding	101142	-
ENSMUSG00000 001520	10.29	0.153	-2.707	2.07754E-05	0.000183627	Nrip2	nuclear receptor interacting protein 2	protein_coding	60345	-
ENSMUSG00000 001666	7.98	0.199	-2.326	0.004882481	0.015244709	Ddt	D-dopachrome tautomerase	protein_coding	13202	-
ENSMUSG00000 001700	82.23	0.17	-2.554	3.54675E-11	2.96425E-09	Gramd3	GRAM domain containing 3	protein_coding	107022	-
ENSMUSG00000 001707	10.8	0.248	-2.009	0.004620969	0.014541889	Eef1e1	eukaryotic translation elongation factor 1 epsilon 1	protein_coding	66143	-
ENSMUSG00000 001755	9.63	0.196	-2.354	0.000295473	0.001561572	Coasy	Coenzyme A synthase	protein_coding	71743	-
ENSMUSG00000 001783	90.82	0.143	-2.807	3.93629E-11	3.24455E-09	Rtcb	R’A ”,3'-cyclic phosphate a’d 5'-OH ligase	protein_coding	28088	-
ENSMUSG00000 001802	7.03	8.168	3.03	0.002185372	0.007981495	Lrp3	low density lipoprotein receptor-related protein 3	protein_coding	435965	-
ENSMUSG00000 001815	7.71	6.226	2.638	0.000186166	0.001076962	Evx2	even-skipped homeobox 2	protein_coding	14029	Homeobox
ENSMUSG00000 001870	58.93	5.099	2.35	6.87712E-10	3.71096E-08	Ltbp1	latent transforming growth factor beta binding protein 1	protein_coding	268977	-

ENSMUSG00000001943	67.17	6.485	2.697	1.87511E-10	1.19482E-08	Vsig2	V-set and immunoglobulin domain containing 2	protein_coding	57276	-
ENSMUSG00000001946	113.25	4.548	2.185	4.60104E-08	1.17438E-06	Esam	endothelial cell-specific adhesion molecule	protein_coding	69524	-
ENSMUSG00000001948	18.47	9.068	3.181	1.66312E-07	3.40372E-06	Spa17	sperm autoantigenic protein 17	protein_coding	20686	-
ENSMUSG00000001983	13.64	0.199	-2.327	4.24681E-05	0.000326953	Taco1	translational activator of mitochondrially encoded cytochrome c oxidase I	protein_coding	70207	-
ENSMUSG00000002007	16.81	0.062	-4.013	3.55138E-09	1.48062E-07	Srpk3	serine/arginine-rich protein specific kinase 3	protein_coding	56504	-
ENSMUSG00000002033	166.78	0.163	-2.613	3.16056E-12	3.73632E-10	Cd3g	CD3 antigen, gamma polypeptide	protein_coding	12502	-
ENSMUSG00000002068	17.82	0.2	-2.324	2.21947E-05	0.000193038	Ccne1	cyclin E1	protein_coding	12447	-
ENSMUSG00000002109	20.61	0.201	-2.314	2.72758E-05	0.00022874	Ddb2	damage specific DNA binding protein 2	protein_coding	107986	-
ENSMUSG00000002129	26.64	0.144	-2.796	1.75093E-06	2.41738E-05	Sf3a1	splicing factor 3a, subunit 1	protein_coding	67465	-
ENSMUSG00000002228	8.52	0.127	-2.973	0.000334433	0.001729331	Ppm1j	protein phosphatase 1J	protein_coding	71887	-
ENSMUSG00000002329	43.99	0.199	-2.333	6.90625E-10	3.71552E-08	Mdp1	magnesium-dependent phosphatase 1	protein_coding	67881	-
ENSMUSG00000002416	20.54	0.166	-2.594	3.63761E-06	4.40757E-05	Ndufb2	NADH:ubiquinone oxidoreductase subunit B2	protein_coding	68198	-
ENSMUSG00000002475	9.85	4.862	2.282	0.000785938	0.003458861	Abhd3	abhydrolase domain containing 3	protein_coding	106861	-
ENSMUSG00000002565	9.88	4.733	2.243	0.004488964	0.014228647	Scin	scinderin	protein_coding	20259	-
ENSMUSG00000002633	9.84	5.054	2.337	0.002530017	0.008959771	Shh	sonic hedgehog	protein_coding	20423	-
ENSMUSG00000002660	23.83	0.152	-2.72	3.72356E-05	0.000293588	Clpp	caseinolytic mitochondrial matrix peptidase proteolytic subunit	protein_coding	53895	-
ENSMUSG00000002718	26.94	0.245	-2.029	5.00574E-06	5.70375E-05	Cse1l	chromosome segregation 1-like (S. cerevisiae)	protein_coding	110750	-
ENSMUSG00000002767	17.69	0.089	-3.494	8.85953E-08	2.0126E-06	Mrpl2	mitochondrial ribosomal protein L2	protein_coding	27398	-
ENSMUSG00000002797	13.24	0.12	-3.06	3.0206E-05	0.000247389	Ggct	gamma-glutamyl cyclotransferase	protein_coding	110175	-
ENSMUSG00000002870	16.81	0.214	-2.221	0.000248042	0.001358033	Mcm2	minichromosome maintenance complex component 2	protein_coding	17216	-
ENSMUSG00000002871	21.75	0.234	-2.096	0.0001905	0.001097852	Tpra1	transmembrane protein, adipoc20aladin20izated 1	protein_coding	24100	-
ENSMUSG00000002949	26.97	0.169	-2.566	3.52255E-08	9.47555E-07	Timm44	translocase of inner mitochondrial membrane 44	protein_coding	21856	-
ENSMUSG00000003072	87.4	0.236	-2.08	1.06172E-07	2.356E-06	Atp5d	ATP synthase, H+ transporting, mitochondrial F1 complex, delta subunit	protein_coding	66043	-

ENSMUSG00000003099	38.1	0.194	-2.364	5.05808E-08	1.2694E-06	Ppp5c	protein phosphatase 5, catalytic subunit	protein_coding	19060	-
ENSMUSG00000003153	663.85	4.805	2.265	5.1496E-08	1.28876E-06	Slc2a3	solute carrier family 2 (facilitated glucose transporter), member 3	protein_coding	20527	-
ENSMUSG00000003184	39.16	0.159	-2.655	1.87382E-07	3.78323E-06	Irf3	interferon regulatory factor 3	protein_coding	54131	IRF
ENSMUSG00000003190	18.93	0.182	-2.458	1.34583E-06	1.92849E-05	Bcl2l12	BCL2-like 12 (proline rich)	protein_coding	75736	-
ENSMUSG00000003227	6.9	4.169	2.06	0.003333071	0.01118861	Edar	ectodysplasin-A receptor	protein_coding	13608	-
ENSMUSG00000003341	6.04	8.069	3.012	3.78645E-05	0.000297372	Atp8b3	ATPase, class I, type 8B, member 3	protein_coding	67331	-
ENSMUSG00000003379	485.66	0.014	-6.132	1.18412E-22	8.511E-20	Cd79a	CD79A antigen (immunoglobulin-associated alpha)	protein_coding	12518	-
ENSMUSG00000003411	8.51	4.215	2.076	0.010155103	0.027444283	Rab3b	RAB3B, member RAS oncogene family	protein_coding	69908	-
ENSMUSG00000003429	812.84	0.222	-2.169	4.24934E-09	1.72752E-07	Rps11	ribosomal protein S11	protein_coding	27207	-
ENSMUSG00000003528	14.59	0.237	-2.074	0.000807424	0.0035456	Slc25a1	solute carrier family 25 (mitochondrial carrier, citrate transporter), member 1	protein_coding	13358	-
ENSMUSG00000003848	30.77	0.236	-2.081	0.000117713	0.000742168	Nob1	NIN1/RPN12 binding protein 1 homolog	protein_coding	67619	-
ENSMUSG00000003923	36.41	0.121	-3.043	1.77403E-08	5.45849E-07	Tfam	transcription factor A, mitochondrial	protein_coding	21780	HMG
ENSMUSG00000003948	1672.27	5.258	2.395	8.70226E-08	1.98692E-06	Mmd	monocyte to macrophage differentiation-associated	protein_coding	67468	-
ENSMUSG00000003949	7.25	4.388	2.134	0.016836964	0.041009297	Hlf	hepatic leukemia factor	protein_coding	217082	TF_bZIP
ENSMUSG00000003955	14.1	0.214	-2.222	6.68776E-05	0.000469974	Fam162a	family with sequence similarity 162, member A	protein_coding	70186	-
ENSMUSG00000004031	12.05	4.099	2.035	0.003365729	0.011285462	Brinp2	bone morphogenic protein/retinoic acid inducible neural-specific 2	protein_coding	240843	-
ENSMUSG00000004044	18.18	9.133	3.191	5.96614E-07	9.88784E-06	Cavin1	caveolae associated 1	protein_coding	19285	-
ENSMUSG00000004085	23.27	4.575	2.194	1.63187E-07	3.35227E-06	Map3k20	mitogen-activated protein kinase kinase kinase 20	protein_coding	65964	-
ENSMUSG00000004100	15.6	0.19	-2.394	7.38686E-05	0.000510139	Ppan	peter pan homolog	protein_coding	235036	-
ENSMUSG00000004231	17.61	5.372	2.425	5.07592E-06	5.7618E-05	Pax2	paired box 2	protein_coding	18504	PAX
ENSMUSG00000004268	100.06	0.169	-2.563	7.69832E-13	1.19251E-10	Emg1	EMG1 N1-specific pseudouridine methyltransferase	protein_coding	14791	-
ENSMUSG00000004393	13.52	0.196	-2.352	0.000346051	0.001776119	Ddx56	DEAD (Asp-Glu-Ala-Asp) box polypeptide 56	protein_coding	52513	-
ENSMUSG00000004562	7.67	5.662	2.501	0.003928172	0.012784882	Arhgef40	Rho guanine nucleotide exchange factor (GEF) 40	protein_coding	268739	-
ENSMUSG00000004631	17.22	4.233	2.082	0.001608373	0.00621925	Sgce	sarcoglycan, epsilon	protein_coding	20392	-

ENSMUSG00000004788	24.7	0.249	-2.009	0.00014523	0.000885526	Eif2b2	eukaryotic translation initiation factor 2B, subunit 2 beta	protein_coding	217715	-
ENSMUSG00000004837	95.17	0.137	-2.865	4.05489E-13	6.62385E-11	Grap	GRB2-related adaptor protein	protein_coding	71520	-
ENSMUSG00000004872	15.75	6.454	2.69	3.33561E-06	4.08016E-05	Pax3	paired box 3	protein_coding	18505	PAX
ENSMUSG00000005089	17.06	6.347	2.666	1.80963E-06	2.47844E-05	Slc1a2	solute carrier family 1 (glial high affinity glutamate transporter), member 2	protein_coding	20511	-
ENSMUSG00000005204	35.86	0.158	-2.663	3.10165E-09	1.33334E-07	Senp3	SUMO/sentrin specific peptidase 3	protein_coding	80886	-
ENSMUSG00000005268	14.98	4.385	2.133	0.004345559	0.01388703	Prlr	prolactin receptor	protein_coding	19116	-
ENSMUSG00000005413	29.51	4.007	2.002	0.000391328	0.00196199	Hmox1	heme oxygenase 1	protein_coding	15368	-
ENSMUSG00000005465	57.14	0.188	-2.408	3.33945E-08	9.0632E-07	Il27ra	interleukin 27 receptor, alpha	protein_coding	50931	-
ENSMUSG00000005505	13.74	0.157	-2.669	0.000100419	0.000655916	Kbtbd4	kelch repeat and BTB (POZ) domain containing 4	protein_coding	67136	-
ENSMUSG00000005540	89.11	0.024	-5.381	1.56673E-24	1.48172E-21	Fcer2a	Fc receptor, IgE, low affinity II, alpha polypeptide	protein_coding	14128	-
ENSMUSG00000005611	94.89	5.986	2.582	1.67125E-14	4.05821E-12	Mrvi1	MRV integration site 1	protein_coding	17540	-
ENSMUSG00000005672	10	4.62	2.208	0.001160736	0.004766284	Kit	KIT proto-oncogene receptor tyrosine kinase	protein_coding	16590	-
ENSMUSG00000005674	17.79	0.247	-2.02	0.000333391	0.001725431	Tomm40l	translocase of outer mitochondrial membrane 40-like	protein_coding	641376	-
ENSMUSG00000005677	11.96	0.229	-2.13	0.000586645	0.002732352	Nr1i3	nuclear receptor subfamily 1, group I, member 3	protein_coding	12355	THR-like
ENSMUSG00000005696	22.9	0.158	-2.666	3.72706E-08	9.9567E-07	Sh2d1a	SH2 domain containing 1A	protein_coding	20400	-
ENSMUSG00000005718	9.56	0.237	-2.076	0.002041516	0.007540391	Tfap4	transcription factor AP4	protein_coding	83383	bHLH
ENSMUSG00000005732	32.76	0.225	-2.152	5.87544E-07	9.78126E-06	Ranbp1	RAN binding protein 1	protein_coding	19385	-
ENSMUSG00000005763	179.7	0.204	-2.296	1.13342E-11	1.13147E-09	Cd247	CD247 antigen	protein_coding	12503	-
ENSMUSG00000005846	32.9	0.216	-2.208	2.41539E-08	6.98909E-07	Rsl1d1	ribosomal L1 domain containing 1	protein_coding	66409	-
ENSMUSG00000005952	5.47	6.558	2.713	0.000711063	0.003190283	Trpv1	transient receptor potential cation channel, subfamily V, member 1	protein_coding	193034	-
ENSMUSG00000005958	4.73	9.684	3.276	0.000456195	0.002231789	Ephb3	Eph receptor B3	protein_coding	13845	-
ENSMUSG00000006204	10.27	7.312	2.87	0.00164741	0.006340979	5430419d17Rik	RIKEN cDNA 5430419D17 gene	protein_coding	71395	-
ENSMUSG00000006288	51.8	0.248	-2.01	3.8491E-07	6.91644E-06	Ttc5	tetratricopeptide repeat domain 5	protein_coding	219022	-
ENSMUSG00000006289	24.63	0.206	-2.282	2.99576E-05	0.000245803	Osgep	O-sialoglycoprotein endopeptidase	protein_coding	66246	-

ENSMUSG00000006315	19.39	0.234	-2.098	2.17061E-05	0.000189799	Tmem147	transmembrane protein 147	protein_coding	69804	-
ENSMUSG00000006356	33.11	4.081	2.029	0.002540961	0.0089879	Crip2	cysteine rich protein 2	protein_coding	68337	-
ENSMUSG00000006360	144.31	0.135	-2.89	1.74686E-16	6.15476E-14	Crip1	cysteine-rich protein 1 (intestinal)	protein_coding	12925	-
ENSMUSG00000006386	19.84	7.049	2.818	1.48666E-06	2.10179E-05	Tek	TEK receptor tyrosine kinase	protein_coding	21687	-
ENSMUSG00000006389	136.87	4.665	2.222	2.12924E-08	6.3345E-07	Mpl	myeloproliferative leukemia virus oncogene	protein_coding	17480	-
ENSMUSG00000006442	18.08	0.217	-2.203	0.000157673	0.000941894	Srm	spermidine synthase	protein_coding	20810	-
ENSMUSG00000006462	9.73	4.51	2.173	0.007912258	0.022467662	A530013C23Rik	RIKEN cDNA A530013C23 gene	lincRNA	329562	-
ENSMUSG00000006498	98.43	0.231	-2.112	2.77812E-09	1.22654E-07	Ptbp1	polypyrimidine tract binding protein 1	protein_coding	19205	-
ENSMUSG00000006567	21.08	5.072	2.343	2.91924E-06	3.66823E-05	Atp7b	ATPase, Cu++ transporting, beta polypeptide	protein_coding	11979	-
ENSMUSG00000006728	49.44	0.138	-2.86	1.84528E-11	1.72697E-09	Cdk4	cyclin-dependent kinase 4	protein_coding	12567	-
ENSMUSG00000006931	7.21	8.842	3.144	5.7026E-05	0.000414188	P3h4	prolyl 3-hydroxylase family member 4 (non-enzymatic)	protein_coding	66180	-
ENSMUSG00000007029	96.34	0.144	-2.8	3.92158E-11	3.24455E-09	Vars	valyl-tRNA synthetase	protein_coding	22321	-
ENSMUSG00000007338	14.01	0.19	-2.397	3.23441E-05	0.000262034	Mrpl49	mitochondrial ribosomal protein L49	protein_coding	18120	-
ENSMUSG00000007603	33.02	0.142	-2.817	9.7804E-10	5.02126E-08	Dus3l	dihydrouridine synthase 3-like (S. cerevisiae)	protein_coding	224907	-
ENSMUSG00000007653	18.32	4.754	2.249	3.11223E-05	0.000253246	Gabrb2	gamma-aminobutyric acid (GABA) A receptor, subunit beta 2	protein_coding	14401	-
ENSMUSG00000007739	93.02	0.211	-2.248	3.25781E-07	6.01022E-06	Cct4	chaperonin containing Tcp1, subunit 4 (delta)	protein_coding	12464	-
ENSMUSG00000007805	9.13	6.762	2.758	0.000545761	0.002573631	Twist2	twist basic helix-loop-helix transcription factor 2	protein_coding	13345	bHLH
ENSMUSG00000007872	29.42	0.05	-4.325	8.02323E-14	1.63829E-11	Id3	inhibitor of DNA binding 3	protein_coding	15903	bHLH
ENSMUSG00000007950	49.49	0.167	-2.581	2.72658E-11	2.37835E-09	Abhd8	abhydrolase domain containing 8	protein_coding	64296	-
ENSMUSG00000007989	11.19	4.615	2.206	6.65977E-05	0.000469108	Fzd3	frizzled class receptor 3	protein_coding	14365	-
ENSMUSG00000008167	4.5	0.014	-6.145	1.19717E-05	0.000115968	Fbxw9	F-box and WD-40 domain protein 9	protein_coding	68628	-
ENSMUSG00000008193	99.38	0.019	-5.717	1.26818E-28	2.07289E-25	Spib	Spi-B transcription factor (Spi-1/PU.1 related)	protein_coding	272382	ETS
ENSMUSG00000008333	30.17	0.237	-2.077	7.54392E-05	0.000518977	Snrpb2	U2 small nuclear ribonucleoprotein B	protein_coding	20639	-
ENSMUSG00000008373	15.98	0.218	-2.196	0.000325785	0.001692405	Prpf31	pre-mRNA processing factor 31	protein_coding	68988	-

ENSMUSG00000 008575	46.76	4.983	2.317	2.53471E-06	3.2533E-05	Nfib	nuclear factor I/B	protein_coding	18028	CTF/NFI
ENSMUSG00000 008668	957.99	0.12	-3.059	3.60146E-12	4.14838E-10	Rps18	ribosomal protein S18	protein_coding	20084	-
ENSMUSG00000 009070	60	5.259	2.395	1.82976E-10	1.17425E-08	Rsph14	radial spoke head homolog 14 (Chlamydomonas)	protein_coding	71236	-
ENSMUSG00000 009097	5.97	5.206	2.38	0.019549824	0.046210312	Tbx1	T-box 1	protein_coding	21380	T-box
ENSMUSG00000 009216	4.97	6.09	2.607	0.011458999	0.030209324	Fam163b	family with sequence similarity 163, member B	protein_coding	109349	-
ENSMUSG00000 009291	2072.27	5.204	2.38	3.30158E-07	6.07227E-06	Pttg1ip	pituitary tumor-transforming 1 interacting protein	protein_coding	108705	-
ENSMUSG00000 009376	14.33	5.367	2.424	3.68057E-05	0.000290839	Met	met proto-oncogene	protein_coding	17295	-
ENSMUSG00000 009487	7.23	4.265	2.092	0.018820287	0.044911253	Otog	otogelin	protein_coding	18419	-
ENSMUSG00000 009614	11.57	7.935	2.988	1.11959E-05	0.000109874	Sardh	sarcosine dehydrogenase	protein_coding	192166	-
ENSMUSG00000 009628	7.37	6.741	2.753	0.000238432	0.00131504	Tex15	testis expressed gene 15	protein_coding	104271	-
ENSMUSG00000 009739	33.8	0.171	-2.544	1.51775E-08	4.79304E-07	Pou6f1	POU domain, class 6, transcription factor 1	protein_coding	19009	Pou
ENSMUSG00000 009927	558.61	0.239	-2.064	2.77546E-09	1.22654E-07	Rps25	ribosomal protein S25	protein_coding	75617	-
ENSMUSG00000 010064	5.98	7.061	2.82	0.000317885	0.001656152	Slc38a3	solute carrier family 38, member 3	protein_coding	76257	-
ENSMUSG00000 010080	4.75	6.101	2.609	0.004673011	0.014682521	Epn3	epsin 3	protein_coding	71889	-
ENSMUSG00000 010142	66.9	0.101	-3.303	1.21655E-12	1.68456E-10	Tnfrsf13b	tumor necrosis factor receptor superfamily, member 13b	protein_coding	57916	-
ENSMUSG00000 010406	30.4	0.244	-2.034	0.000170814	0.001003848	Mrpl52	mitochondrial ribosomal protein L52	protein_coding	68836	-
ENSMUSG00000 010461	25.28	4.121	2.043	1.13349E-06	1.6736E-05	Eya4	EYA transcriptional coactivator and phosphatase 4	protein_coding	14051	-
ENSMUSG00000 010476	25.05	6.81	2.768	4.659E-08	1.1864E-06	Ebf3	early B cell factor 3	protein_coding	13593	COE
ENSMUSG00000 010607	8.63	0.123	-3.025	8.50752E-05	0.000571937	Pigyl	phosphatidylinositol glycan anchor biosynthesis, class Y-like	protein_coding	66268	-
ENSMUSG00000 010797	4.43	5.654	2.499	0.005562082	0.016953573	Wnt2	wingless-type MMTV integration site family, member 2	protein_coding	22413	-
ENSMUSG00000 010803	5.85	5.415	2.437	0.000398867	0.001995332	Gabra1	gamma-aminobutyric acid (GABA) A receptor, subunit alpha 1	protein_coding	14394	-
ENSMUSG00000 010825	13.67	4.374	2.129	9.07282E-05	0.000602426	Grid2ip	glutamate receptor, ionotropic, delta 2 (Grid2) interacting protein 1	protein_coding	170935	-
ENSMUSG00000 011589	4.93	6.288	2.653	0.011244744	0.029793541	Fsd1	fibronectin type 3 and SPRY domain-containing protein	protein_coding	240121	-
ENSMUSG00000 012017	11.51	4.872	2.284	0.000162485	0.000965507	Scarf2	scavenger receptor class F, member 2	protein_coding	224024	-

ENSMUSG00000 012483	12.54	0.173	-2.529	0.000101096	0.000659379	Rpa3	replication protein A3	protein_coding	68240	-
ENSMUSG00000 013419	5.61	10.651	3.413	0.000493106	0.002368127	Zfp651	zinc finger protein 651	protein_coding	270210	ZBTB
ENSMUSG00000 013584	12.37	6.351	2.667	4.00105E-06	4.7576E-05	Aldh1a2	aldehyde dehydrogenase family 1, subfamily A2	protein_coding	19378	-
ENSMUSG00000 013611	7.14	4.185	2.065	0.003408189	0.011387459	Snx31	sorting nexin 31	protein_coding	66696	-
ENSMUSG00000 014030	122.27	0.211	-2.248	3.45451E-10	1.99595E-08	Pax5	paired box 5	protein_coding	18507	PAX
ENSMUSG00000 014158	15.8	5.578	2.48	1.51754E-05	0.000141435	Trpv4	transient receptor potential cation channel, subfamily V, member 4	protein_coding	63873	-
ENSMUSG00000 014329	25.75	5.156	2.366	0.000400062	0.001999084	Bicc1	BicC family RNA binding protein 1	protein_coding	83675	-
ENSMUSG00000 014355	28.36	0.234	-2.098	0.000109797	0.000703868	Anapc1	anaphase promoting complex subunit 1	protein_coding	17222	-
ENSMUSG00000 014361	11.22	5.55	2.472	0.000357102	0.00181984	Mertk	c-mer proto-oncogene tyrosine kinase	protein_coding	17289	-
ENSMUSG00000 014453	96.52	0.081	-3.63	1.41895E-21	9.10614E-19	Blk	B lymphoid kinase	protein_coding	12143	-
ENSMUSG00000 014554	32.31	0.231	-2.117	3.67802E-07	6.64224E-06	Dguok	deoxyguanosine kinase	protein_coding	27369	-
ENSMUSG00000 014767	30.05	0.247	-2.017	0.000321586	0.001672043	Tbp	TATA box binding protein	protein_coding	21374	-
ENSMUSG00000 014771	14.29	0.143	-2.809	1.6708E-05	0.000153176	Pdcd2	programmed cell death 2	protein_coding	18567	-
ENSMUSG00000 014873	15.03	0.172	-2.539	0.000107154	0.000691118	Surf2	surfeit gene 2	protein_coding	20931	-
ENSMUSG00000 015134	3.94	5.433	2.442	0.002521615	0.008935299	Aldh1a3	aldehyde dehydrogenase family 1, subfamily A3	protein_coding	56847	-
ENSMUSG00000 015305	25.27	4.901	2.293	7.65575E-07	1.21097E-05	Sash1	SAM and SH3 domain containing 1	protein_coding	70097	-
ENSMUSG00000 015314	76.9	0.218	-2.199	5.75806E-08	1.4193E-06	Slamf6	SLAM family member 6	protein_coding	30925	-
ENSMUSG00000 015316	347.47	5.326	2.413	6.57757E-09	2.45212E-07	Slamf1	signaling lymphocytic activation molecule family member 1	protein_coding	27218	-
ENSMUSG00000 015337	8.32	0.201	-2.313	0.001503913	0.005885162	Endog	endonuclease G	protein_coding	13804	-
ENSMUSG00000 015355	85.38	0.23	-2.118	1.00885E-10	7.19364E-09	Cd48	CD48 antigen	protein_coding	12506	-
ENSMUSG00000 015396	35.44	0.151	-2.73	5.93253E-08	1.45432E-06	Cd83	CD83 antigen	protein_coding	12522	-
ENSMUSG00000 015461	43.15	0.227	-2.137	2.37048E-07	4.61609E-06	Atf6b	activating transcription factor 6 beta	protein_coding	12915	TF_bZIP
ENSMUSG00000 015484	11.65	10.974	3.456	1.2309E-06	1.7953E-05	Fam163a	family with sequence similarity 163, member A	protein_coding	329274	-
ENSMUSG00000 015597	56.04	0.21	-2.251	3.60631E-08	9.67191E-07	Zfp318	zinc finger protein 318	protein_coding	57908	-

ENSMUSG00000 015806	15.39	0.237	-2.075	0.000457671	0.002237183	Qdpr	quinoid dihydropteridine reductase	protein_coding	110391	-
ENSMUSG00000 015843	5.12	4.698	2.232	0.019478094	0.046105584	Rxrg	retinoid X receptor gamma	protein_coding	20183	RXR-like
ENSMUSG00000 015879	17.67	5.121	2.357	3.7902E-05	0.000297536	Fam184b	family with sequence similarity 184, member B	protein_coding	58227	-
ENSMUSG00000 015957	6.68	4.786	2.259	0.001088178	0.004521032	Wnt11	wingless-type MMTV integration site family, member 11	protein_coding	22411	-
ENSMUSG00000 016128	20.75	6.022	2.59	3.33273E-06	4.07942E-05	Stard13	StAR-related lipid transfer (START) domain containing 13	protein_coding	243362	-
ENSMUSG00000 016206	31.06	0.105	-3.255	2.8296E-10	1.67511E-08	H2-M3	histocompatibility 2, M region locus 3	protein_coding	14991	-
ENSMUSG00000 016255	577.14	5.251	2.393	7.74158E-08	1.80895E-06	Tubb1	tubulin, beta 1 class VI	protein_coding	545486	-
ENSMUSG00000 016346	10.67	8.085	3.015	8.82146E-06	9.03722E-05	Kcnq2	potassium voltage-gated channel, subfamily Q, member 2	protein_coding	16536	-
ENSMUSG00000 016382	8.45	4.078	2.028	0.006960242	0.020283585	Pls3	plastin 3 (T-isoform)	protein_coding	102866	-
ENSMUSG00000 016554	73.67	0.186	-2.43	5.5795E-11	4.41666E-09	Eif3d	eukaryotic translation initiation factor 3, subunit D	protein_coding	55944	-
ENSMUSG00000 016763	22.34	7.049	2.817	1.339E-06	1.92176E-05	Scube1	signal peptide, CUB domain, EGF-like 1	protein_coding	64706	-
ENSMUSG00000 016918	19.17	4.116	2.041	0.000383535	0.001931542	Sulf1	sulfatase 1	protein_coding	240725	-
ENSMUSG00000 016921	114.79	0.246	-2.023	3.71438E-10	2.11651E-08	Srsf6	serine/arginine-rich splicing factor 6	protein_coding	67996	-
ENSMUSG00000 016984	10.83	0.092	-3.44	9.46791E-06	9.53103E-05	Etaa1	Ewing tumor-associated antigen 1	protein_coding	68145	-
ENSMUSG00000 016995	6.47	4.484	2.165	0.000737366	0.003285328	Matn4	matrilin 4	protein_coding	17183	-
ENSMUSG00000 017009	22.91	0.171	-2.55	2.1579E-05	0.000188854	Sdc4	syndecan 4	protein_coding	20971	-
ENSMUSG00000 017221	39.76	0.225	-2.15	1.25097E-07	2.68219E-06	Psmd3	proteasome (prosome, macropain) 26S subunit, non-ATPase, 3	protein_coding	22123	-
ENSMUSG00000 017314	27.3	4.677	2.225	3.28458E-05	0.000265619	Mpp2	membrane protein, palmitoylated 2 (MAGUK p55 subfamily member 2)	protein_coding	50997	-
ENSMUSG00000 017615	13.44	0.222	-2.173	0.000483277	0.002333158	Tnfaip1	tumor necrosis factor, alpha-induced protein 1 (endothelial)	protein_coding	21927	-
ENSMUSG00000 017652	34.83	0.048	-4.384	5.61618E-15	1.48408E-12	Cd40	CD40 antigen	protein_coding	21939	-
ENSMUSG00000 017688	11.2	5.302	2.407	1.91991E-05	0.000171807	Hnf4g	hepatocyte nuclear factor 4, gamma	protein_coding	30942	RXR-like
ENSMUSG00000 017716	15.43	4.225	2.079	0.000295994	0.00156387	Birc5	baculoviral IAP repeat-containing 5	protein_coding	11799	-
ENSMUSG00000 017724	5.23	5.843	2.547	0.01341506	0.03414017	Etv4	ets variant 4	protein_coding	18612	ETS
ENSMUSG00000 017740	10.95	4.094	2.034	0.001325611	0.005309833	Slc12a5	solute carrier family 12, member 5	protein_coding	57138	-

ENSMUSG00000017801	28.35	0.194	-2.365	6.37565E-06	6.92649E-05	Mlx	MAX-like protein X	protein_coding	21428	bHLH
ENSMUSG00000017817	11.29	7.159	2.84	0.000524341	0.00249256	Jph2	junctophilin 2	protein_coding	59091	-
ENSMUSG00000017978	231.79	24.114	4.592	6.4624E-07	1.06117E-05	Cadps2	Ca2+-dependent activator protein for secretion 2	protein_coding	320405	-
ENSMUSG00000018126	7.52	7.219	2.852	0.012556876	0.032460726	Baiap2l2	BAI1-associated protein 2-like 2	protein_coding	207495	-
ENSMUSG00000018168	104.49	0.184	-2.444	4.97918E-09	1.96639E-07	Ikzf3	IKAROS family zinc finger 3	protein_coding	22780	zf-C2H2
ENSMUSG00000018169	80.39	0.139	-2.848	1.26317E-13	2.38925E-11	Mfng	MFNG O-fucosylpeptide 3-beta-N-acetylglucosaminyltransferase	protein_coding	17305	-
ENSMUSG00000018199	69.27	4.944	2.306	1.54406E-08	4.85907E-07	Trove2	TROVE domain family, member 2	protein_coding	20822	-
ENSMUSG00000018417	19.67	4.125	2.044	2.23146E-05	0.000193612	Myo1b	myosin IB	protein_coding	17912	-
ENSMUSG00000018446	25.44	0.147	-2.769	2.34446E-06	3.05494E-05	C1qbp	complement component 1, q subcomponent binding protein	protein_coding	12261	-
ENSMUSG00000018459	16.45	5.081	2.345	9.42448E-05	0.00062169	Slc13a3	solute carrier family 13 (sodium-dependent dicarboxylate transporter), member 3	protein_coding	114644	-
ENSMUSG00000018474	157.55	0.171	-2.547	3.54401E-12	4.10853E-10	Chd3	chromodomain helicase DNA binding protein 3	protein_coding	216848	-
ENSMUSG00000018593	5.31	4.264	2.092	0.012386895	0.032122978	Sparc	secreted acidic cysteine rich glycoprotein	protein_coding	20692	-
ENSMUSG00000018648	15.14	5.327	2.413	0.000224991	0.00125555	Dusp14	dual specificity phosphatase 14	protein_coding	56405	-
ENSMUSG00000018698	9.68	9.795	3.292	7.84925E-07	1.23722E-05	Lhx1	LIM homeobox protein 1	protein_coding	16869	Homeobox
ENSMUSG00000018750	42.63	0.159	-2.655	4.27761E-09	1.73509E-07	Zbtb4	zinc finger and BTB domain containing 4	protein_coding	75580	ZBTB
ENSMUSG00000018821	3.34	0.019	-5.713	0.000176169	0.001028121	Avpi1	arginine vasopressin-induced 1	protein_coding	69534	-
ENSMUSG00000018848	16.61	0.143	-2.809	9.04389E-06	9.20213E-05	Rars	arginyl-tRNA synthetase	protein_coding	104458	-
ENSMUSG00000018858	23.17	0.21	-2.25	1.25729E-06	1.82637E-05	Mrpl58	mitochondrial ribosomal protein L58	protein_coding	68572	-
ENSMUSG00000018882	10.36	0.209	-2.26	0.001702836	0.006504731	Mrpl45	mitochondrial ribosomal protein L45	protein_coding	67036	-
ENSMUSG00000018965	1265.66	4.347	2.12	2.33878E-06	3.04975E-05	Ywhah	tyrosine 3-monooxygenase/tryptophan 5-monooxygenase activation protein, eta polypeptide	protein_coding	22629	-
ENSMUSG00000019027	8.8	4.906	2.295	0.003049322	0.010411032	Dnah1	dynein, axonemal, heavy chain 1	protein_coding	110084	-
ENSMUSG00000019039	33.53	0.211	-2.243	5.14546E-07	8.814E-06	Dalrd3	DALR anticodon binding domain containing 3	protein_coding	67789	-
ENSMUSG00000019055	22.46	4.046	2.017	0.000860245	0.003731281	Plod1	procollagen-lysine, 2-oxoglutarate 5-dioxygenase 1	protein_coding	18822	-

ENSMUSG00000 019080	3.31	0.172	-2.541	0.013688244	0.034681902	Mfsd3	major facilitator superfamily domain containing 3	protein_coding	69572	-
ENSMUSG00000 019122	62.03	0.22	-2.183	4.06351E-06	4.81317E-05	Ccl9	chemokine (C-C motif) ligand 9	protein_coding	20308	-
ENSMUSG00000 019295	12.66	0.146	-2.774	1.69429E-05	0.000155014	Tmem129	transmembrane protein 129	protein_coding	68366	-
ENSMUSG00000 019297	13.8	0.22	-2.186	0.000191833	0.001103763	Nop9	NOP9 nucleolar protein	protein_coding	67842	-
ENSMUSG00000 019432	223.79	0.241	-2.051	8.33311E-10	4.34075E-08	Ddx39b	DEAD (Asp-Glu-Ala-Asp) box polypeptide 39B	protein_coding	53817	-
ENSMUSG00000 019518	14.59	0.18	-2.474	7.07278E-05	0.000493173	Ap4m1	adaptor-related protein complex AP-4, mu 1	protein_coding	11781	-
ENSMUSG00000 019579	25.87	0.096	-3.377	9.18267E-11	6.73483E-09	Mydgf	myeloid derived growth factor	protein_coding	28106	-
ENSMUSG00000 019647	16.36	4.634	2.212	2.01005E-05	0.000178805	Sema6a	sema domain, transmembrane domI(TM), and cytoplasmic d28aladin28izamaphorin) 6A	protein_coding	20358	-
ENSMUSG00000 019710	59.26	0.224	-2.16	1.11487E-08	3.74451E-07	Mrpl24	mitochondrial ribosomal protein L24	protein_coding	67707	-
ENSMUSG00000 019803	8.88	4.49	2.167	0.000347585	0.001781673	Nr2e1	nuclear receptor subfamily 2, group E, member 1	protein_coding	21907	RXR-like
ENSMUSG00000 019817	10.93	4.641	2.215	0.001214562	0.004945493	Plagl1	pleiomorphic adenoma gene-like 1	protein_coding	22634	zf-C2H2
ENSMUSG00000 019852	25.97	4.808	2.265	5.39025E-07	9.13749E-06	Arfgef3	ARFGEF family member 3	protein_coding	215821	-
ENSMUSG00000 019913	9.29	5.57	2.478	0.001190328	0.004860032	Sim1	single-minded family bHLH transcription factor 1	protein_coding	20464	bHLH
ENSMUSG00000 019917	10.25	4.535	2.181	0.002123028	0.007791809	Sept10	septin 10	protein_coding	103080	-
ENSMUSG00000 019927	26.12	0.247	-2.018	0.000157907	0.000942952	Ube2d1	ubiquitin-conjugating enzyme E2D 1	protein_coding	216080	-
ENSMUSG00000 019929	5.28	4.614	2.206	0.010517053	0.028273628	Dcn	decorin	protein_coding	13179	-
ENSMUSG00000 020000	6.31	5.012	2.325	0.007253957	0.020952637	Moxd1	monooxygenase, DBH-like 1	protein_coding	59012	-
ENSMUSG00000 020018	30.63	0.2	-2.323	2.9276E-06	3.67617E-05	Snrpf	small nuclear ribonucleoprotein polypeptide F	protein_coding	69878	-
ENSMUSG00000 020032	11.32	5.506	2.461	0.000869829	0.003758105	Nuak1	NUAK family, SNF1-like kinase, 1	protein_coding	77976	-
ENSMUSG00000 020048	117.62	0.249	-2.008	9.27346E-09	3.24192E-07	Hsp90b1	heat shock protein 90, beta (Grp94), member 1	protein_coding	22027	-
ENSMUSG00000 020067	10.7	5.933	2.569	0.000866391	0.003747755	Mypn	myopalladin	protein_coding	68802	-
ENSMUSG00000 020069	30.59	0.196	-2.348	1.49459E-07	3.13011E-06	Hnrnph3	heterogeneous nuclear ribonucleoprotein H3	protein_coding	432467	-
ENSMUSG00000 020075	55.13	0.202	-2.309	5.81949E-08	1.43072E-06	Ddx21	DEAD (Asp-Glu-Ala-Asp) box polypeptide 21	protein_coding	56200	-
ENSMUSG00000 020090	6.01	4.775	2.255	0.014707768	0.036726498	Npffr1	neuropeptide FF receptor 1	protein_coding	237362	-

ENSMUSG00000 020099	18.93	5.199	2.378	8.46011E-06	8.75186E-05	Unc5b	unc-5 netrin receptor B	protein_coding	107449	-
ENSMUSG00000 020120	1291.05	4.388	2.133	4.19026E-08	1.09281E-06	Plek	pleckstrin	protein_coding	56193	-
ENSMUSG00000 020135	10.2	4.269	2.094	0.002487358	0.008834817	Apc2	adenomatosis polyposis coli 2	protein_coding	23805	-
ENSMUSG00000 020142	8.07	6.116	2.613	0.004532129	0.014321248	Slc1a4	solute carrier family 1 (glutamate/neutral amino acid transporter), member 4	protein_coding	55963	-
ENSMUSG00000 020156	43.77	0.213	-2.234	7.03161E-09	2.58916E-07	Mum1	melanoma associated antigen (mutated) 1	protein_coding	68114	-
ENSMUSG00000 020167	111.57	0.243	-2.044	1.7594E-08	5.42276E-07	Tcf3	transcription factor 3	protein_coding	21423	bHLH
ENSMUSG00000 020169	6.79	4.531	2.18	0.004009746	0.013002563	Best3	bestrophin 3	protein_coding	382427	-
ENSMUSG00000 020171	37.87	0.24	-2.062	9.36151E-07	1.43634E-05	Yeats4	YEATS domain containing 4	protein_coding	64050	-
ENSMUSG00000 020181	55.57	4.351	2.121	8.71961E-08	1.98836E-06	Nav3	neuron navigator 3	protein_coding	260315	-
ENSMUSG00000 020185	8.88	4.457	2.156	0.008171932	0.023041182	E2f7	E2F transcription factor 7	protein_coding	52679	E2F
ENSMUSG00000 020205	12.14	4.185	2.065	0.001571658	0.00610487	Phlda1	pleckstrin homology like domain, family A, member 1	protein_coding	21664	-
ENSMUSG00000 020219	39.52	0.167	-2.581	1.88811E-09	9.0473E-08	Timm13	translocase of inner mitochondrial membrane 13	protein_coding	30055	-
ENSMUSG00000 020231	20.29	4.661	2.221	0.000155402	0.000931738	Dip2a	disco interacting protein 2 homolog A	protein_coding	64451	-
ENSMUSG00000 020268	6.25	0.134	-2.903	0.001433409	0.005663351	Lyrm7	LYR motif containing 7	protein_coding	75530	-
ENSMUSG00000 020312	5.81	5.081	2.345	0.001624872	0.006266864	Shc2	SHC (Src homology 2 domain containing) transforming protein 2	protein_coding	216148	-
ENSMUSG00000 020331	8.37	5.892	2.559	0.000472497	0.002290341	Hcn2	hyperpolarization-activated, cyclic nucleotide-gated K+ 2	protein_coding	15166	-
ENSMUSG00000 020358	123.49	0.247	-2.018	9.31888E-10	4.81181E-08	Hnrnpab	heterogeneous nuclear ribonucleoprotein A/B	protein_coding	15384	-
ENSMUSG00000 020363	6.5	5.304	2.407	0.020235722	0.047500416	Gfpt2	glutamine fructose-6-phosphate transaminase 2	protein_coding	14584	-
ENSMUSG00000 020374	8.21	7.79	2.962	6.75245E-05	0.000473964	Rasgef1c	RasGEF domain family, member 1C	protein_coding	74563	-
ENSMUSG00000 020401	7.24	0.109	-3.2	8.98719E-05	0.000599001	Fam71b	family with sequence similarity 71, member B	protein_coding	432552	-
ENSMUSG00000 020407	11.24	4.524	2.178	0.000385404	0.001938424	Upp1	uridine phosphorylase 1	protein_coding	22271	-
ENSMUSG00000 020422	46.86	5.079	2.345	1.44959E-07	3.05724E-06	Tns3	tensin 3	protein_coding	319939	-
ENSMUSG00000 020430	23.22	0.12	-3.06	2.83223E-09	1.24618E-07	Pes1	pescadillo ribosomal biogenesis factor 1	protein_coding	64934	-
ENSMUSG00000 020431	16.58	7.327	2.873	6.42938E-06	6.95962E-05	Adcy1	adenylate cyclase 1	protein_coding	432530	-

ENSMUSG00000 020435	16.62	4.738	2.244	0.00023188	0.001287991	Osbp2	oxysterol binding protein 2	protein_coding	74309	-
ENSMUSG00000 020436	6.52	8.857	3.147	0.000195182	0.001119448	Gabrg2	gamma-aminobutyric acid (GABA) A receptor, subunit gamma 2	protein_coding	14406	-
ENSMUSG00000 020439	62.78	5.003	2.323	1.10623E-08	3.72943E-07	Smtn	smoothelin	protein_coding	29856	-
ENSMUSG00000 020459	17.52	0.178	-2.489	3.07158E-06	3.80905E-05	Mtif2	mitochondrial translational initiation factor 2	protein_coding	76784	-
ENSMUSG00000 020460	737.55	0.248	-2.01	1.78568E-07	3.62563E-06	Rps27a	ribosomal protein S27A	protein_coding	78294	-
ENSMUSG00000 020471	14.8	0.124	-3.014	1.53959E-06	2.15627E-05	Pold2	polymerase (DNA directed), delta 2, regulatory subunit	protein_coding	18972	-
ENSMUSG00000 020473	5.9	10.165	3.346	0.000427231	0.00211293	Aebp1	AE binding protein 1	protein_coding	11568	-
ENSMUSG00000 020486	5.95	6.101	2.609	0.012015059	0.031377727	Sept4	septin 4	protein_coding	18952	-
ENSMUSG00000 020495	10.88	0.233	-2.103	0.00088771	0.00382616	Smg8	smg-8 homolog, nonsense mediated mRNA decay factor (C. elegans)	protein_coding	74133	-
ENSMUSG00000 020537	18.84	0.197	-2.341	4.77145E-06	5.47156E-05	Drg2	developmentally regulated GTP binding protein 2	protein_coding	13495	-
ENSMUSG00000 020542	7.65	4.704	2.234	0.020195658	0.047412565	Myocd	myocardin	protein_coding	214384	-
ENSMUSG00000 020549	13.7	0.072	-3.793	9.35862E-06	9.44748E-05	Elac2	elaC ribonuclease Z 2	protein_coding	68626	-
ENSMUSG00000 020553	60.12	5.819	2.541	1.60695E-08	5.01831E-07	Pctp	phosphatidylcholine transfer protein	protein_coding	18559	-
ENSMUSG00000 020614	8.63	4.169	2.06	0.008524405	0.023851342	Fam20a	family with sequence similarity 20, member A	protein_coding	208659	-
ENSMUSG00000 020627	40.34	5.843	2.547	2.91094E-11	2.50271E-09	Klhl29	kelch-like 29	protein_coding	208439	-
ENSMUSG00000 020633	9.57	4.016	2.006	0.002898267	0.00998138	Dcdc2c	doublecortin domain containing 2C	protein_coding	68511	-
ENSMUSG00000 020672	18.96	4.21	2.074	0.000847513	0.0036874	Sntg2	syntrophin, gamma 2	protein_coding	268534	-
ENSMUSG00000 020674	11.25	8.24	3.043	6.465E-06	6.99395E-05	Pxdn	peroxidasin	protein_coding	69675	-
ENSMUSG00000 020679	7.3	5.955	2.574	0.000280342	0.001494351	Hnf1b	HNF1 homeobox B	protein_coding	21410	Homeobox
ENSMUSG00000 020681	228.89	7.555	2.917	0.001294557	0.005208663	Ace	angiotensin I converting enzyme (peptidyl-dipeptidase A) 1	protein_coding	11421	-
ENSMUSG00000 020692	15.7	0.169	-2.564	3.7532E-05	0.000295255	Nle1	notchless homolog 1	protein_coding	217011	-
ENSMUSG00000 020701	10.1	6.415	2.682	0.000256169	0.001392767	Tmem132e	transmembrane protein 132E	protein_coding	270893	-
ENSMUSG00000 020702	12.03	7.303	2.869	0.000282555	0.001504363	Ccl1	chemokine (C-C motif) ligand 1	protein_coding	20290	-
ENSMUSG00000 020706	19.77	0.137	-2.87	2.99321E-05	0.000245803	Ftsj3	FtsJ RNA methyltransferase homolog 3 (E. coli)	protein_coding	56095	-

ENSMUSG00000 020736	52.24	0.135	-2.892	1.72847E-10	1.12941E-0’	N’5c	5',3'-nucleotidase, cytosolic	protein_coding	50773	-
ENSMUSG00000 020743	50.31	0.147	-2.764	1.28671E-10	8.79125E-09	Mif4gd	MIF4G domain containing	protein_coding	69674	-
ENSMUSG00000 020787	250.14	4.235	2.082	4.07186E-08	1.07126E-06	P2rx1	purinergic receptor P2X, ligand-gated ion channel, 1	protein_coding	18436	-
ENSMUSG00000 020826	6.67	17.866	4.159	4.82126E-05	0.000363242	Nos2	nitric oxide synthase 2, inducible	protein_coding	18126	-
ENSMUSG00000 020848	6.49	7.588	2.924	0.001684722	0.00645475	Doc2b	double C2, beta	protein_coding	13447	-
ENSMUSG00000 020857	142.16	0.153	-2.704	2.03505E-12	2.55719E-10	Nme2	NME/NM23 nucleoside diphosphate kinase 2	protein_coding	18103	-
ENSMUSG00000 020869	25.46	0.237	-2.078	1.54863E-05	0.000143885	Lrrc59	leucine rich repeat containing 59	protein_coding	98238	-
ENSMUSG00000 020870	6.14	5.635	2.494	0.018375722	0.04408075	Cdc34b	-	protein_coding	-	-
ENSMUSG00000 020904	5.93	6.721	2.749	0.000262441	0.001417862	Cfap52	cilia and flagella associated protein 52	protein_coding	71860	-
ENSMUSG00000 020908	6.69	6.689	2.742	0.001272478	0.005138237	Myh3	myosin, heavy polypeptide 3, skeletal muscle, embryonic	protein_coding	17883	-
ENSMUSG00000 020910	10.37	0.181	-2.462	0.000721341	0.003226732	Adprm	ADP-ribose/CDP-alcohol diphosphatase, manganese dependent	protein_coding	66358	-
ENSMUSG00000 020926	5.73	6.507	2.702	0.006853604	0.020024782	Adam11	a disintegrin and metallopeptidase domain 11	protein_coding	11488	-
ENSMUSG00000 020961	133.87	4.02	2.007	3.58263E-07	6.52242E-031aladin2	stonin 2	protein_coding	108800	-	
ENSMUSG00000 021010	69.73	4.665	2.222	7.65179E-10	4.0679E-08	Npas3	neuronal PAS domain protein 3	protein_coding	27386	-
ENSMUSG00000 021018	7.28	0.075	-3.732	4.34101E-05	0.00033278	Polr2h	polymerase (RNA) II (DNA directed) polypeptide H	protein_coding	245841	-
ENSMUSG00000 021047	9.82	5.184	2.374	0.000408725	0.002036559	Nova1	neuro-oncological ventral antigen 1	protein_coding	664883	-
ENSMUSG00000 021071	14.27	4.652	2.218	0.000726589	0.003243744	Trim9	tripartite motif-containing 9	protein_coding	94090	-
ENSMUSG00000 021098	9.61	5.285	2.402	0.003532743	0.011727297	4930447C04Rik	RIKEN cDNA 4930447C04 gene	protein_coding	75801	-
ENSMUSG00000 021118	8.33	15.005	3.907	2.13266E-05	0.00018738	Plek2	pleckstrin 2	protein_coding	27260	-
ENSMUSG00000 021127	93.2	0.243	-2.044	1.03602E-08	3.55919E-07	Zfp36l1	zinc finger protein 36, C3H type-like 1	protein_coding	12192	-
ENSMUSG00000 021130	16.11	5.136	2.361	8.21064E-05	0.000555695	Galnt16	polypeptide N-acetylgalactosaminyltransferase 16	protein_coding	108760	-
ENSMUSG00000 021131	44.69	0.183	-2.446	1.09637E-08	3.71712E-07	Erh	ERH mRNA splicing and mitosis factor	protein_coding	13877	-
ENSMUSG00000 021149	18.29	0.083	-3.588	2.18039E-09	1.00719E-07	Gtpbp4	GTP binding protein 4	protein_coding	69237	-
ENSMUSG00000 021194	5.44	23.836	4.575	2.45708E-07	4.75767E-06	Chga	chromogranin A	protein_coding	12652	-

ENSMUSG00000021203	22.89	4.542	2.183	0.000170216	0.001002166	Otub2	OTU domain, ubiquitin aldehyde binding 2	protein_coding	68149	-
ENSMUSG00000021252	12.13	0.106	-3.235	2.24179E-06	2.93869E-05	Erg28	ergosterol biosynthesis 28	protein_coding	58520	-
ENSMUSG00000021260	6.72	7.518	2.91	0.001088458	0.004521151	Hhipl1	hedgehog interacting protein-like 1	protein_coding	214305	-
ENSMUSG00000021262	238.6	0.234	-2.097	1.33936E-08	4.35207E-07	Evl	Ena-vasodilator stimulated phosphoprotein	protein_coding	14026	-
ENSMUSG00000021263	154.55	0.22	-2.187	8.7723E-09	3.08473E-07	Degs2	delta(4)-desaturase, sphingolipid 2	protein_coding	70059	-
ENSMUSG00000021356	22.21	0.14	-2.837	2.83364E-08	7.9311E-07	Irf4	interferon regulatory factor 4	protein_coding	16364	IRF
ENSMUSG00000021373	32.35	4.736	2.244	5.45724E-06	6.12499E-05	Cap2	CAP, adenylate cyclase-associated protein, 2 (yeast)	protein_coding	67252	-
ENSMUSG00000021376	5.96	5.608	2.488	0.002830243	0.009791773	Tpmt	thiopurine methyltransferase	protein_coding	22017	-
ENSMUSG00000021413	47.2	0.182	-2.46	3.08249E-10	1.80421E-08	Prpf4b	pre-mRNA processing factor 4B	protein_coding	19134	-
ENSMUSG00000021423	66.1	0.202	-2.306	7.93562E-09	2.85007E-07	Ly86	lymphocyte antigen 86	protein_coding	17084	-
ENSMUSG00000021476	23.88	0.232	-2.106	0.000356496	0.001817564	Habp4	hyaluronic acid binding protein 4	protein_coding	56541	-
ENSMUSG00000021493	240.08	4.226	2.079	1.82789E-05	0.000165052	Pdlim7	PDZ and LIM domain 7	protein_coding	67399	-
ENSMUSG00000021504	30.27	0.164	-2.612	4.0536E-10	2.29777E-08	B4galt7	xylosylprotein beta1,4-galactosyltransferase, polypeptide 7 (galactosyltransferase I)	protein_coding	218271	-
ENSMUSG00000021506	5.54	4.267	2.093	0.004014024	0.013010101	Pitx1	paired-like homeodomain transcription factor 1	protein_coding	18740	Homeobox
ENSMUSG00000021508	3.88	9.697	3.278	0.007021861	0.020426678	Cxcl14	chemokine (C-X-C motif) ligand 14	protein_coding	57266	-
ENSMUSG00000021514	14.88	0.184	-2.44	0.000199696	0.001142057	Zfp369	zinc finger protein 369	protein_coding	170936	zf-C2H2
ENSMUSG00000021537	25.19	0.157	-2.671	1.29404E-06	1.87069E-05	Cetn3	centrin 3	protein_coding	12626	-
ENSMUSG00000021567	6.74	13.928	3.8	0.000299594	0.001579639	Nkd2	naked cuticle 2	protein_coding	72293	-
ENSMUSG00000021575	8.2	4.096	2.034	0.002875427	0.009926713	Ahrr	aryl-hydrocarbon receptor repressor	protein_coding	11624	bHLH
ENSMUSG00000021583	40.22	0.241	-2.054	3.97671E-07	7.12438E-06	Erap1	endoplasmic reticulum aminopeptidase 1	protein_coding	80898	-
ENSMUSG00000021589	9.38	5.17	2.37	0.00057145	0.002669886	Rhobtb3	Rho-related BTB domain containing 3	protein_coding	73296	-
ENSMUSG00000021596	421.13	5.329	2.414	3.50029E-10	2.00308E-08	Mctp1	multiple C2 domains, transmembrane 1	protein_coding	78771	-
ENSMUSG00000021604	7.4	11.941	3.578	6.69312E-06	7.18451E-05	Irx4	Iroquois homeobox 4	protein_coding	50916	Homeobox
ENSMUSG00000021608	43.62	0.229	-2.129	5.87066E-09	2.26373E-07	Lpcat1	lysophosphatidylcholine acyltransferase 1	protein_coding	210992	-

ENSMUSG00000 021624	44.13	0.216	-2.211	4.97396E-07	8.57745E-06	Cd180	CD180 antigen	protein_coding	17079	-
ENSMUSG00000 021675	49.6	4.632	2.212	1.42366E-07	3.00739E-06	F2rl2	coagulation factor II (thrombin) receptor-like 2	protein_coding	14064	-
ENSMUSG00000 021678	11.41	0.228	-2.133	0.017469021	0.042253444	F2rl1	coagulation factor II (thrombin) receptor-like 1	protein_coding	14063	-
ENSMUSG00000 021685	7.83	4.974	2.314	0.000404857	0.002020089	Otp	orthopedia homeobox	protein_coding	18420	Homeobox
ENSMUSG00000 021687	67.59	4.044	2.016	7.99225E-07	1.25536E-05	Scamp1	secretory carrier membrane protein 1	protein_coding	107767	-
ENSMUSG00000 021696	24.48	4.402	2.138	2.57525E-06	3.29592E-05	Elovl7	ELOVL family member 7, elongation of long chain fatty acids (yeast)	protein_coding	74559	-
ENSMUSG00000 021704	5.53	0.097	-3.37	0.000221896	0.001241362	Mtx3	metaxin 3	protein_coding	382793	-
ENSMUSG00000 021730	33.93	4.321	2.111	3.90635E-08	1.03562E-06	Hcn1	hyperpolarization-activated, cyclic nucleotide-gated K+ 1	protein_coding	15165	-
ENSMUSG00000 021737	25.34	0.24	-2.056	8.42556E-05	0.00056801	Psmd6	proteasome (prosome, macropain) 26S subunit, non-ATPase, 6	protein_coding	66413	-
ENSMUSG00000 021748	33.73	0.163	-2.613	2.5448E-08	7.29306E-07	Pdhb	pyruvate dehydrogenase (lipoamide) beta	protein_coding	68263	-
ENSMUSG00000 021756	54.68	0.19	-2.396	4.67515E-10	2.63347E-08	Il6st	interleukin 6 signal transducer	protein_coding	16195	-
ENSMUSG00000 021758	7.85	5.203	2.379	0.001006956	0.004246418	Ddx4	DEAD (Asp-Glu-Ala-Asp) box polypeptide 4	protein_coding	13206	-
ENSMUSG00000 021798	8.85	5.985	2.581	0.000589895	0.002743934	Ldb3	LIM domain binding 3	protein_coding	24131	-
ENSMUSG00000 021807	42.09	0.226	-2.145	2.81209E-08	7.88305E-07	2700060E02R ik	RNA transcription, translation and transport factor	protein_coding	68045	-
ENSMUSG00000 021811	41.94	0.109	-3.201	5.03492E-11	4.03895E-09	Dnajc9	DnaJ heat shock protein family (Hsp40) member C9	protein_coding	108671	-
ENSMUSG00000 021832	23.07	0.235	-2.088	1.48496E-05	0.000138903	Psmc6	proteasome (prosome, macropain) 26S subunit, ATPase, 6	protein_coding	67089	-
ENSMUSG00000 021930	5.67	0.193	-2.376	0.007890024	0.022422244	Spryd7	SPRY domain containing 7	protein_coding	66674	-
ENSMUSG00000 021944	13.04	4.786	2.259	0.004041464	0.013081274	Gata4	GATA binding protein 4	protein_coding	14463	zf-GATA
ENSMUSG00000 021974	9.83	7.849	2.973	0.001228744	0.004995026	Fgf9	fibroblast growth factor 9	protein_coding	14180	-
ENSMUSG00000 021986	26.76	5.256	2.394	1.15033E-06	1.69567E-05	Amer2	APC membrane recruitment 2	protein_coding	72125	-
ENSMUSG00000 021996	870.82	4.211	2.074	6.77993E-06	7.26467E-05	Esd	esterase D/formylglutathione hydrolase	protein_coding	13885	-
ENSMUSG00000 021997	10.25	5.163	2.368	0.000194385	0.001115585	Lrrc63	leucine rich repeat containing 63	protein_coding	70859	-
ENSMUSG00000 022010	8465.79	6.745	2.754	7.49453E-08	1.75579E-06	Tsc22d1	TSC22 domain family, member 1	protein_coding	21807	TSC22
ENSMUSG00000 022037	1408.19	5.868	2.553	2.15242E-08	6.36133E-07	Clu	clusterin	protein_coding	12759	-

ENSMUSG00000022076	21.1	4.123	2.044	0.000408808	0.002036559	Klhl1	kelch-like 1	protein_coding	93688	-
ENSMUSG00000022103	9.58	5.09	2.348	5.29785E-05	0.000390145	Gfra2	glial cell line derived neurotrophic factor family receptor alpha 2	protein_coding	14586	-
ENSMUSG00000022109	18.85	0.155	-2.685	6.41067E-06	6.95192E-05	Med4	mediator complex subunit 4	protein_coding	67381	-
ENSMUSG00000022228	23.39	0.143	-2.807	2.44216E-09	1.11096E-07	Zscan26	zinc finger and SCAN domain containing 26	protein_coding	432731	zf-C2H2
ENSMUSG00000022234	121.25	0.176	-2.503	1.46837E-13	2.72012E-11	Cct5	chaperonin containing Tcp1, subunit 5 (epsilon)	protein_coding	12465	-
ENSMUSG00000022241	16.25	0.245	-2.031	0.00013518	0.000835009	Tars	threonyl-tRNA synthetase	protein_coding	110960	-
ENSMUSG00000022245	10.24	7.641	2.934	9.19247E-05	0.000608172	Skor1	SKI family transcriptional corepressor 1	protein_coding	207667	-
ENSMUSG00000022248	7.74	0.137	-2.868	0.000140568	0.000860893	Rad1	RAD1 checkpoint DNA exonuclease	protein_coding	19355	-
ENSMUSG00000022269	11.74	4.549	2.185	0.000106867	0.000690007	March11	membrane-associated ring finger (C3HC4) 11	protein_coding	211147	-
ENSMUSG00000022303	53.23	7.392	2.886	3.48532E-13	5.79886E-11	Dcstamp	dendrocyte expressed seven transmembrane protein	protein_coding	75766	-
ENSMUSG00000022309	85.82	4.81	2.266	3.94554E-09	1.61866E-07	Angpt1	angiopoietin 1	protein_coding	11600	-
ENSMUSG00000022322	5.81	6.641	2.731	0.003993512	0.012955303	Shcbp1	Shc SH2-domain binding protein 1	protein_coding	20419	-
ENSMUSG00000022323	134.12	4.583	2.196	2.8725E-08	8.00246E-07	Rida	reactive intermediate imine deaminase A homolog	protein_coding	15473	-
ENSMUSG00000022336	115.47	0.104	-3.272	8.19258E-13	1.24793E-10	Eif3e	eukaryotic translation initiation factor 3, subunit E	protein_coding	16341	-
ENSMUSG00000022338	14.79	0.166	-2.589	1.27319E-05	0.000122146	Eny2	ENY2 transcription and export complex 2 subunit	protein_coding	223527	-
ENSMUSG00000022357	4.45	11.86	3.568	0.001057802	0.004421439	Klhl38	kelch-like 38	protein_coding	268807	-
ENSMUSG00000022366	13.77	4.386	2.133	0.000336275	0.001737356	Slc22a22	solute carrier family 22 (organic cation transporter), member 22	protein_coding	210463	-
ENSMUSG00000022370	19.22	0.232	-2.109	2.43879E-05	0.000208283	Mrpl13	mitochondrial ribosomal protein L13	protein_coding	68537	-
ENSMUSG00000022371	18.35	4.075	2.027	9.05626E-05	0.000602042	Col14a1	collagen, type XIV, alpha 1	protein_coding	12818	-
ENSMUSG00000022407	23.94	0.146	-2.778	3.33956E-05	0.000269459	Adsl	adenylosuccinate lyase	protein_coding	11564	-
ENSMUSG00000022421	13.86	4.275	2.096	0.000292342	0.001548448	Nptxr	neuronal pentraxin receptor	protein_coding	73340	-
ENSMUSG00000022432	69.12	32.964	5.043	9.33945E-06	9.43342E-05	Smc1b	structural maintenance of chromosomes 1B	protein_coding	140557	-
ENSMUSG00000022434	12.04	0.21	-2.252	0.000100047	0.000654203	Fam118a	family with sequence similarity 118, member A	protein_coding	73225	-
ENSMUSG00000022438	824.47	4.187	2.066	9.79514E-07	1.48531E-05	Parvb	parvin, beta	protein_coding	170736	-

ENSMUSG00000 022449	18.47	5.807	2.538	0.001361893	0.005428669	Adamts20	a disintegrin-like and metallopeptidase (reprolysin type) with thrombospondin type 1 motif, 20	protein_coding	223838	-
ENSMUSG00000 022454	26.74	4.586	2.197	2.69593E-07	5.14071E-06	Nell2	NEL-like 2	protein_coding	54003	-
ENSMUSG00000 022463	88.19	0.182	-2.455	5.2207E-10	2.90436E-08	Srebf2	sterol regulatory element binding factor 2	protein_coding	20788	bHLH
ENSMUSG00000 022500	975.66	6.131	2.616	3.02757E-09	1.31965E-07	Litaf	LPS-induced TN factor	protein_coding	56722	zf-LITAF-like
ENSMUSG00000 022503	40.38	0.216	-2.21	1.44062E-08	4.61315E-07	Nubp1	nucleotide binding protein 1	protein_coding	26425	-
ENSMUSG00000 022504	97.54	0.041	-4.601	2.731E-25	2.72629E-22	Ciita	class II transactivator	protein_coding	12265	-
ENSMUSG00000 022510	17.36	4.217	2.076	9E-05	0.000599633	Trp63	transformation related protein 63	protein_coding	22061	P53
ENSMUSG00000 022516	22.83	0.182	-2.461	2.02706E-06	2.7041E-05	Nudt16l1	nudix (nucleoside diphosphate linked moiety X)-type motif 16-like 1	protein_coding	66911	-
ENSMUSG00000 022523	16.95	5.455	2.448	5.87899E-05	0.000423745	Fgf12	fibroblast growth factor 12	protein_coding	14167	-
ENSMUSG00000 022544	17.82	0.166	-2.589	4.56847E-07	8.00105E-06	Eef2kmt	eukaryotic elongation factor 2 lysine methyltransferase	protein_coding	70511	-
ENSMUSG00000 022545	18.73	0.199	-2.33	9.09092E-06	9.23952E-05	Ercc4	excision repair cross-complementing rodent repair deficiency, complementation group 4	protein_coding	50505	-
ENSMUSG00000 022551	46.08	0.236	-2.084	9.86303E-09	3.42802E-07	Cyc1	cytochrome c-1	protein_coding	66445	-
ENSMUSG00000 022560	9.98	0.205	-2.29	0.001092575	0.004535109	Slc52a2	solute carrier protein 52, member 2	protein_coding	52710	-
ENSMUSG00000 022571	13.35	0.101	-3.305	3.1334E-06	3.87769E-05	Pycrl	pyrroline-5-carboxylate reductase-like	protein_coding	66194	-
ENSMUSG00000 022594	16.68	0.209	-2.257	0.000112243	0.000716734	Lynx1	Ly6/neurotoxin 1	protein_coding	23936	-
ENSMUSG00000 022615	8.77	0.185	-2.434	0.002251966	0.008169913	Tymp	thymidine phosphorylase	protein_coding	72962	-
ENSMUSG00000 022621	4.92	4.939	2.304	0.008967392	0.024843519	Rabl2	RAB, member RAS oncogene family-like 2	protein_coding	68708	-
ENSMUSG00000 022634	126.65	4.549	2.185	2.80623E-10	1.66971E-08	Yaf2	YY1 associated factor 2	protein_coding	67057	-
ENSMUSG00000 022657	39.6	0.23	-2.121	4.93174E-06	5.63014E-05	Cd96	CD96 antigen	protein_coding	84544	-
ENSMUSG00000 022659	13.11	5.563	2.476	0.005292736	0.016279558	Gcsam	germinal center associated, signaling and motility	protein_coding	14525	-
ENSMUSG00000 022661	15.87	0.151	-2.724	5.48025E-05	0.000401773	Cd200	CD200 antigen	protein_coding	17470	-
ENSMUSG00000 022668	13.04	0.226	-2.147	0.000654597	0.002980855	Gtpbp8	GTP-binding protein 8 (putative)	protein_coding	66067	-

ENSMUSG00000022673	14.1	0.176	-2.505	5.52628E-05	0.000403994	Mcm4	minichromosome maintenance complex component 4	protein_coding	17217	-
ENSMUSG00000022674	14.05	0.171	-2.55	4.72922E-06	5.44042E-05	Ube2v2	ubiquitin-conjugating enzyme E2 variant 2	protein_coding	70620	-
ENSMUSG00000022682	19.28	0.231	-2.117	4.07322E-05	0.000316163	Rrn3	RRN3 RNA polymerase I transcription factor homolog (yeast)	protein_coding	106298	-
ENSMUSG00000022683	9.93	4.683	2.227	0.008119927	0.022952174	Pla2g10	phospholipase A2, group X	protein_coding	26565	-
ENSMUSG00000022686	54.31	0.022	-5.496	1.38431E-28	2.07289E-25	B3gnt5	UDP-GlcNAc:betaGal beta-1,3-N-acetylglucosaminyltransferase 5	protein_coding	108105	-
ENSMUSG00000022696	77.54	0.247	-2.019	4.35058E-07	7.69444E-06	Sidt1	SID1 transmembrane family, member 1	protein_coding	320007	-
ENSMUSG00000022721	43.73	0.204	-2.295	5.94747E-07	9.86796E-06	Trmt2a	TRM2 tRNA methyltransferase 2A	protein_coding	15547	-
ENSMUSG00000022754	4.54	8.048	3.009	0.011478742	0.030234759	Tmem45a	transmembrane protein 45a	protein_coding	56277	-
ENSMUSG00000022769	24.93	0.223	-2.164	7.3485E-07	1.17478E-05	Sdf2l1	stromal cell-derived factor 2-like 1	protein_coding	64136	-
ENSMUSG00000022774	26.05	0.205	-2.286	1.45908E-05	0.000136981	Ncbp2	nuclear cap binding protein subunit 2	protein_coding	68092	-
ENSMUSG00000022787	11.73	0.246	-2.025	0.005682084	0.017267859	Wdr53	WD repeat domain 53	protein_coding	68980	-
ENSMUSG00000022799	17.52	5.725	2.517	0.00016582	0.000981108	Arhgap31	Rho GTPase activating protein 31	protein_coding	12549	-
ENSMUSG00000022802	32.8	4.081	2.029	0.004511479	0.01427483	Lmln	leishmanolysin-like (metallopeptidase M8 family)	protein_coding	239833	-
ENSMUSG00000022803	36.23	6.272	2.649	2.26307E-10	1.40256E-08	Popdc2	popeye domain containing 2	protein_coding	64082	-
ENSMUSG00000022805	6.15	5.184	2.374	0.005186891	0.016022562	Maats1	MYCBP-associated, testis expressed 1	protein_coding	320214	-
ENSMUSG00000022816	6.9	4.547	2.185	0.010948987	0.029177272	Fstl1	follistatin-like 1	protein_coding	14314	-
ENSMUSG00000022817	555.79	4.199	2.07	4.81127E-07	8.36109E-06	Itgb5	integrin beta 5	protein_coding	16419	-
ENSMUSG00000022857	7.35	4.115	2.041	0.004335066	0.013863107	Tmprss15	transmembrane protease, serine 15	protein_coding	19146	-
ENSMUSG00000022885	100.87	0.189	-2.401	7.09758E-08	1.68032E-06	St6gal1	beta galactoside alpha 2,6 sialyltransferase 1	protein_coding	20440	-
ENSMUSG00000022887	7.99	5.103	2.351	0.0019521	0.007268396	Masp1	mannan-binding lectin serine peptidase 1	protein_coding	17174	-
ENSMUSG00000022949	6.97	4.691	2.23	0.002069521	0.007620332	Clic6	chloride intracellular channel 6	protein_coding	209195	-
ENSMUSG00000022992	46.48	0.147	-2.768	2.01003E-08	6.06009E-07	Kansl2	KAT8 regulatory NSL complex subunit 2	protein_coding	69612	-
ENSMUSG00000022997	6.7	4.721	2.239	0.011378617	0.030054736	Wnt1	wingless-type MMTV integration site family, member 1	protein_coding	22408	-
ENSMUSG00000023018	41.14	0.168	-2.574	1.55012E-10	1.0203E-08	Smarcd1	SWI/SNF related, matrix associated, actin dependent regulator of chromatin, subfamily d, member 1	protein_coding	83797	-

ENSMUSG00000023019	4.14	5.483	2.455	0.005647501	0.017173794	Gpd1	glycerol-3-phosphate dehydrogenase 1 (soluble)	protein_coding	14555	-
ENSMUSG00000023033	33.7	5.16	2.367	1.079E-08	3.67903E-07	Scn8a	sodium channel, voltage-gated, type VIII, alpha	protein_coding	20273	-
ENSMUSG00000023039	12.14	4.326	2.113	0.003277502	0.011045281	Krt7	keratin 7	protein_coding	110310	-
ENSMUSG00000023067	46.06	5.587	2.482	5.43727E-05	0.000399111	Cdkn1a	cyclin-dependent kinase inhibitor 1A (P21)	protein_coding	12575	-
ENSMUSG00000023092	479.08	5.317	2.411	9.42704E-08	2.12274E-06	Fhl1	four and a half LIM domains 1	protein_coding	14199	-
ENSMUSG00000023106	28.24	0.25	-2.003	2.69673E-06	3.41973E-05	Denr	density-regulated protein	protein_coding	68184	-
ENSMUSG00000023110	19	0.199	-2.328	0.000435405	0.002148805	Prmt5	protein arginine N-methyltransferase 5	protein_coding	27374	-
ENSMUSG00000023150	83.96	0.243	-2.038	1.00025E-08	3.4631E-07	Ivns1abp	influenza virus NS1A binding protein	protein_coding	117198	-
ENSMUSG00000023156	16.1	0.244	-2.035	0.000398801	0.001995332	Rpp14	ribonuclease P 14 subunit	protein_coding	67053	-
ENSMUSG00000023191	5.58	8.797	3.137	0.001649038	0.00634509	P3h3	prolyl 3-hydroxylase 3	protein_coding	14789	-
ENSMUSG00000023274	68.74	0.223	-2.165	9.82272E-09	3.42063E-07	Cd4	CD4 antigen	protein_coding	12504	-
ENSMUSG00000023403	6.72	4.22	2.077	0.015874891	0.0390708	Stk31	serine threonine kinase 31	protein_coding	77485	-
ENSMUSG00000023577	6.34	8.988	3.168	0.00026927	0.001448216	Iqcf3	IQ motif containing F3	protein_coding	68265	-
ENSMUSG00000023828	19.95	11.111	3.474	2.24377E-09	1.02331E-07	Slc22a3	solute carrier family 22 (organic cation transporter), member 3	protein_coding	20519	-
ENSMUSG00000023886	13.1	6.717	2.748	0.000772574	0.00340923	Smoc2	SPARC related modular calcium binding 2	protein_coding	64074	-
ENSMUSG00000023931	10.52	4.174	2.061	0.000530315	0.002514975	Efhb	EF hand domain family, member B	protein_coding	211482	-
ENSMUSG00000023947	27.56	0.164	-2.606	1.47858E-05	0.000138418	Nfkbie	nuclear factor of kappa light polypeptide gene enhancer in B cells inhibitor, epsilon	protein_coding	18037	-
ENSMUSG00000023951	17.33	5.075	2.344	0.00028232	0.001503559	Vegfa	vascular endothelial growth factor A	protein_coding	22339	-
ENSMUSG00000023959	21.7	5.492	2.457	0.001072563	0.004463383	Clic5	chloride intracellular channel 5	protein_coding	224796	-
ENSMUSG00000023960	8.86	4.905	2.294	0.006542919	0.019311012	Enpp5	ectonucleotide pyrophosphatase/phosphodiesterase 5	protein_coding	83965	-
ENSMUSG00000023961	8.41	0.246	-2.025	0.00748921	0.021522469	Enpp4	ectonucleotide pyrophosphatase/phosphodiesterase 4	protein_coding	224794	-
ENSMUSG00000023967	16.43	0.179	-2.482	6.60045E-06	7.102E-05	Mrps18a	mitochondrial ribosomal protein S18A	protein_coding	68565	-
ENSMUSG00000023972	10.89	7.855	2.974	4.96571E-05	0.000370552	Ptk7	PTK7 protein tyrosine kinase 7	protein_coding	71461	-

ENSMUSG00000023992	11.23	5.241	2.39	0.001657013	0.006370319	Trem2	triggering receptor expressed on myeloid cells 2	protein_coding	83433	-
ENSMUSG00000024013	73.06	0.149	-2.745	4.23055E-12	4.84196E-10	Fgd2	FYVE, RhoGEF and PH domain containing 2	protein_coding	26382	-
ENSMUSG00000024049	11.96	5.026	2.329	3.66614E-05	0.000290206	Myom1	myomesin 1	protein_coding	17929	-
ENSMUSG00000024069	9.04	0.203	-2.3	0.007611861	0.021795003	Slc30a6	solute carrier family 30 (zinc transporter), member 6	protein_coding	210148	-
ENSMUSG00000024078	300.64	4.037	2.013	3.88263E-08	1.03322E-06	Ttc27	tetratricopeptide repeat domain 27	protein_coding	74196	-
ENSMUSG00000024097	73.22	0.157	-2.671	8.00068E-15	2.05377E-12	Srsf7	serine/arginine-rich splicing factor 7	protein_coding	225027	-
ENSMUSG00000024134	6.54	7.068	2.821	0.000418569	0.002075979	Six2	sine oculis-related homeobox 2	protein_coding	20472	Homeobox
ENSMUSG00000024142	13.69	0.134	-2.904	9.21146E-06	9.33037E-05	Mlst8	MTOR associated protein, LST8 homolog (S. cerevisiae)	protein_coding	56716	-
ENSMUSG00000024181	19.25	0.184	-2.441	3.23127E-06	3.97689E-05	Mrpl28	mitochondrial ribosomal protein L28	protein_coding	68611	-
ENSMUSG00000024205	23.1	0.237	-2.078	9.69877E-05	0.000636745	Rpl36-ps2	-	processed_pseudogene	-	-
ENSMUSG00000024207	7.52	16.318	4.028	1.01145E-06	1.52473E-05	Acsbg2	acyl-CoA synthetase bubblegum family member 2	protein_coding	328845	-
ENSMUSG00000024211	74.62	4.172	2.061	4.65544E-07	8.12961E-06	Grm8	glutamate receptor, metabotropic 8	protein_coding	14823	-
ENSMUSG00000024215	4.82	5.03	2.331	0.017066409	0.041463804	Spdef	SAM pointed domain containing ets transcription factor	protein_coding	30051	ETS
ENSMUSG00000024245	6.29	4.906	2.294	0.002309251	0.008335663	Tmem178	transmembrane protein 178	protein_coding	68027	-
ENSMUSG00000024299	61.51	0.241	-2.053	7.78762E-09	2.81561E-07	Adamts10	a disintegrin-like and metallopeptidase (reprolysin type) with thrombospondin type 1 motif, 10	protein_coding	224697	-
ENSMUSG00000024325	19.5	0.23	-2.12	4.54039E-05	0.000345998	Ring1	ring finger protein 1	protein_coding	19763	-
ENSMUSG00000024334	59.72	0.057	-4.133	3.05284E-14	6.83099E-12	H2-Oa	histocompatibility 2, O region alpha locus	protein_coding	15001	-
ENSMUSG00000024347	7.92	14.081	3.816	9.79021E-07	1.48531E-05	Psd2	pleckstrin and Sec7 domain containing 2	protein_coding	74002	-
ENSMUSG00000024349	41.3	0.222	-2.17	2.4627E-07	4.76141E-06	Tmem173	transmembrane protein 173	protein_coding	72512	-
ENSMUSG00000024353	51.05	0.102	-3.298	2.69349E-08	7.64604E-07	Mzb1	marginal zone B and B1 cell-specific protein 1	protein_coding	69816	-
ENSMUSG00000024376	27.92	5.207	2.38	1.66256E-07	3.40372E-06	Epb41l4a	erythrocyte membrane protein band 4.1 like 4a	protein_coding	13824	-
ENSMUSG00000024436	8.58	0.118	-3.083	8.18551E-05	0.000554621	Mrps18b	mitochondrial ribosomal protein S18B	protein_coding	66973	-
ENSMUSG00000024442	17.14	0.158	-2.666	1.76572E-06	2.42942E-05	0610009O20Rik	DAP3 binding cell death enhancer 1	protein_coding	66839	-

ENSMUSG00000 024500	29.25	4.149	2.053	0.002508488	0.008895799	Ppp2r2b	protein phosphatase 2, regulatory subunit B, beta	protein_coding	72930	-
ENSMUSG00000 024501	15.37	6.383	2.674	2.0797E-05	0.000183727	Dpysl3	dihydropyrimidinase-like 3	protein_coding	22240	-
ENSMUSG00000 024511	124.89	5.664	2.502	9.65392E-13	1.44559E-10	Rab27b	RAB27B, member RAS oncogene family	protein_coding	80718	-
ENSMUSG00000 024534	19.26	6.921	2.791	5.89667E-07	9.80178E-06	Sncaip	synuclein, alpha interacting protein (synphilin)	protein_coding	67847	-
ENSMUSG00000 024535	21.08	4.49	2.167	3.4655E-05	0.000278371	Snx24	sorting nexing 24	protein_coding	69226	-
ENSMUSG00000 024556	32.88	0.234	-2.095	6.46658E-07	1.06117E-05	Me2	malic enzyme 2, NAD(+)-dependent, mitochondrial	protein_coding	107029	-
ENSMUSG00000 024610	1517.78	0.025	-5.317	6.63678E-27	7.45352E-24	Cd74	CD74 antigen (invariant polypeptide of major histocompatibility complex, class II antigen-associated)	protein_coding	16149	-
ENSMUSG00000 024613	63.46	0.236	-2.081	2.36213E-08	6.86815E-07	Tcof1	treacle ribosome biogenesis factor 1	protein_coding	21453	-
ENSMUSG00000 024622	22.91	0.21	-2.248	1.30702E-06	1.88187E-05	Hmgxb3	HMG box domain containing 3	protein_coding	106894	HMG
ENSMUSG00000 024645	12.32	0.082	-3.607	1.39453E-07	2.95152E-06	Timm21	translocase of inner mitochondrial membrane 21	protein_coding	67105	-
ENSMUSG00000 024665	27.59	7.582	2.923	1.79097E-10	1.1618E-08	Fads2	fatty acid desaturase 2	protein_coding	56473	-
ENSMUSG00000 024669	61.41	0.124	-3.008	1.70103E-14	4.07545E-12	Cd5	CD5 antigen	protein_coding	12507	-
ENSMUSG00000 024670	81.72	0.243	-2.038	2.0369E-09	9.53151E-08	Cd6	CD6 antigen	protein_coding	12511	-
ENSMUSG00000 024673	172.8	0.008	-6.925	1.33067E-39	5.97771E-36	Ms4a1	membrane-spanning 4-domains, subfamily A, member 1	protein_coding	12482	-
ENSMUSG00000 024675	108.26	0.192	-2.384	2.78788E-10	1.6643E-08	Ms4a4c	membrane-spanning 4-domains, subfamily A, member 4C	protein_coding	64380	-
ENSMUSG00000 024677	305.47	0.212	-2.236	5.57881E-11	4.41666E-09	Ms4a6b	membrane-spanning 4-domains, subfamily A, member 6B	protein_coding	69774	-
ENSMUSG00000 024680	8.31	0.123	-3.02	5.54155E-05	0.000404548	Ms4a2	membrane-spanning 4-domains, subfamily A, member 2	protein_coding	14126	-
ENSMUSG00000 024683	8.84	0.161	-2.637	0.001487638	0.005833858	Mrpl16	mitochondrial ribosomal protein L16	protein_coding	94063	-
ENSMUSG00000 024696	32.36	0.142	-2.812	2.30118E-10	1.42096E-08	Lpxn	leupaxin	protein_coding	107321	-
ENSMUSG00000 024713	75.65	5.212	2.382	8.28913E-06	8.59878E-05	Pcsk5	proprotein convertase subtilisin/kexin type 5	protein_coding	18552	-
ENSMUSG00000 024735	31.75	0.093	-3.422	6.05223E-11	4.68761E-09	Prpf19	pre-mRNA processing factor 19	protein_coding	28000	-
ENSMUSG00000 024742	13.45	0.164	-2.612	0.000106296	0.000687307	Fen1	flap structure specific endonuclease 1	protein_coding	14156	-
ENSMUSG00000 024793	15.04	0.165	-2.601	0.000174653	0.001021263	Tnfrsf25	tumor necrosis factor receptor superfamily, member 25	protein_coding	85030	-
ENSMUSG00000 024798	10.79	4.102	2.036	0.002722046	0.009492034	Htr7	5-hydroxytryptamine (serotonin) receptor 7	protein_coding	15566	-

ENSMUSG00000024831	12.76	0.188	-2.409	7.94684E-05	0.000541307	Ighmbp2	immunoglobulin mu binding protein 2	protein_coding	20589	-
ENSMUSG00000024854	85.43	0.181	-2.469	7.18461E-12	7.59413E-10	Pold4	polymerase (DNA-directed), delta 4	protein_coding	69745	-
ENSMUSG00000024869	13.52	0.211	-2.247	0.001328991	0.005320998	Nudt8	-	protein_coding	-	-
ENSMUSG00000024875	13.51	0.184	-2.439	0.000664301	0.003016635	Yif1a	Yip1 interacting factor homolog A (S. cerevisiae)	protein_coding	68090	-
ENSMUSG00000024889	12.32	0.082	-3.61	6.60092E-07	1.07927E-05	Rce1	Ras converting CAAX endopeptidase 1	protein_coding	19671	-
ENSMUSG00000024897	39.64	4.181	2.064	4.7395E-05	0.000358284	Apba1	amyloid beta (A4) precursor protein binding, family A, member 1	protein_coding	319924	-
ENSMUSG00000024906	13.03	0.231	-2.116	0.000555263	0.002605776	Mus81	MUS81 structure-specific endonuclease subunit	protein_coding	71711	-
ENSMUSG00000024909	7.06	4.949	2.307	0.00898927	0.024892616	Efemp2	epidermal growth factor-containing fibulin-like extracellular matrix protein 2	protein_coding	58859	-
ENSMUSG00000024910	32.95	0.121	-3.049	1.32169E-10	8.92835E-09	Ctsw	cathepsin W	protein_coding	13041	-
ENSMUSG00000024911	21.24	0.246	-2.023	0.00011369	0.000721869	Fibp	fibroblast growth factor (acidic) intracellular binding protein	protein_coding	58249	-
ENSMUSG00000024924	5.01	5.627	2.492	0.004909596	0.015316065	Vldlr	very low density lipoprotein receptor	protein_coding	22359	-
ENSMUSG00000024987	5.85	10.174	3.347	1.90251E-05	0.00017076	Cyp26a1	cytochrome P450, family 26, subfamily a, polypeptide 1	protein_coding	13082	-
ENSMUSG00000025020	22.88	4.584	2.197	4.40913E-06	5.13798E-05	Slit1	slit guidance ligand 1	protein_coding	20562	-
ENSMUSG00000025038	13.04	4.906	2.294	0.000978549	0.004147062	Efhc2	EF-hand domain (C-terminal) containing 2	protein_coding	74405	-
ENSMUSG00000025064	11.29	5.811	2.539	9.57277E-06	9.62581E-05	Col17a1	collagen, type XVII, alpha 1	protein_coding	12821	-
ENSMUSG00000025083	12.92	4.41	2.141	0.001022269	0.004297091	Afap1l2	actin filament associated protein 1-like 2	protein_coding	226250	-
ENSMUSG00000025085	275.76	0.249	-2.005	4.68342E-09	1.87849E-07	Ablim1	actin-binding LIM protein 1	protein_coding	226251	-
ENSMUSG00000025145	12.27	0.222	-2.173	0.00162361	0.006264688	Lrrc45	leucine rich repeat containing 45	protein_coding	217366	-
ENSMUSG00000025155	22.56	0.234	-2.093	1.08919E-05	0.000107242	Dus1l	dihydrouridine synthase 1-like (S. cerevisiae)	protein_coding	68730	-
ENSMUSG00000025170	4.27	4.794	2.261	0.011340639	0.029994105	Rab40b	Rab40B, member RAS oncogene family	protein_coding	217371	-
ENSMUSG00000025178	35.28	0.236	-2.085	3.90755E-08	1.03562E-06	Pi4k2a	phosphatidylinositol 4-kinase type 2 alpha	protein_coding	84095	-
ENSMUSG00000025212	29.75	0.225	-2.149	3.22161E-05	0.000261587	Sfxn3	sideroflexin 3	protein_coding	94280	-
ENSMUSG00000025216	3.95	4.973	2.314	0.019533756	0.046182516	Lbx1	ladybird homeobox 1	protein_coding	16814	Homeobox
ENSMUSG00000025260	31.6	0.196	-2.352	2.10156E-08	6.2729E-07	Hsd17b10	hydroxysteroid (17-beta) dehydrogenase 10	protein_coding	15108	-

ENSMUSG00000025264	6.66	0.17	-2.553	0.00202479	0.007490932	Tsr2	TSR2 20S rRNA accumulation	protein_coding	69499	-
ENSMUSG00000025265	7.44	4.164	2.058	0.011344612	0.029995781	Fgd1	FYVE, RhoGEF and PH domain containing 1	protein_coding	14163	-
ENSMUSG00000025357	229.72	0.199	-2.33	1.0672E-12	1.58484E-10	Dgka	diacylglycerol kinase, alpha	protein_coding	13139	-
ENSMUSG00000025366	136.19	0.171	-2.547	4.1789E-13	6.76493E-11	Esyt1	extended synaptotagmin-like protein 1	protein_coding	23943	-
ENSMUSG00000025381	16	0.2	-2.318	0.000164593	0.000975129	Cnpy2	canopy FGF signaling regulator 2	protein_coding	56530	-
ENSMUSG00000025420	7.82	4.819	2.269	0.000821091	0.0035942	Katnal2	katanin p60 subunit A-like 2	protein_coding	71206	-
ENSMUSG00000025491	489.57	7.061	2.82	1.09482E-07	2.39913E-06	Ifitm1	interferon induced transmembrane protein 1	protein_coding	68713	-
ENSMUSG00000025555	40.87	4.117	2.042	2.16786E-07	4.28254E-06	Farp1	FERM, RhoGEF (Arhgef) and pleckstrin domain protein 1 (chondrocyte-derived)	protein_coding	223254	-
ENSMUSG00000025576	65.56	4.287	2.1	1.25236E-07	2.68219E-06	Rbfox3	RNA binding protein, fox-1 homolog (C. elegans) 3	protein_coding	52897	-
ENSMUSG00000025607	220.65	5.69	2.508	1.04358E-14	2.64113E-12	Copg2	coatomer protein complex, subunit gamma 2	protein_coding	54160	-
ENSMUSG00000025626	22.68	0.234	-2.098	0.000138581	0.000851157	Phf6	PHD finger protein 6	protein_coding	70998	-
ENSMUSG00000025645	8.7	0.204	-2.29	0.008808034	0.02449645	Ccdc51	coiled-coil domain containing 51	protein_coding	66658	-
ENSMUSG00000025650	6.06	4.137	2.049	0.005809979	0.017587518	Col7a1	collagen, type VII, alpha 1	protein_coding	12836	-
ENSMUSG00000025658	14.81	4.621	2.208	0.000523344	0.002488481	Cnksr2	connector enhancer of kinase suppressor of Ras 2	protein_coding	245684	-
ENSMUSG00000025722	21.18	0.21	-2.251	6.98034E-05	0.000488243	Wdr73	WD repeat domain 73	protein_coding	71968	-
ENSMUSG00000025738	5.37	4.524	2.178	0.011747778	0.030809284	Fbxl16	F-box and leucine-rich repeat protein 16	protein_coding	214931	-
ENSMUSG00000025779	5.53	5.794	2.535	0.001578443	0.006125926	Ly96	lymphocyte antigen 96	protein_coding	17087	-
ENSMUSG00000025794	518.38	0.181	-2.465	2.60402E-09	1.16688E-07	Rpl14	ribosomal protein L14	protein_coding	67115	-
ENSMUSG00000025813	14.29	4.158	2.056	0.000136635	0.000841974	Homer2	homer scaffolding protein 2	protein_coding	26557	-
ENSMUSG00000025856	57	4.801	2.263	3.08682E-08	8.50722E-07	Pdgfa	platelet derived growth factor, alpha	protein_coding	18590	-
ENSMUSG00000025869	8.69	0.116	-3.114	0.000200782	0.001144987	Nop16	NOP16 nucleolar protein	protein_coding	28126	-
ENSMUSG00000025872	19.37	0.208	-2.263	9.81709E-05	0.000643808	Thoc3	THO complex 3	protein_coding	73666	-
ENSMUSG00000025876	15.4	6.08	2.604	0.000378443	0.001910179	Unc5a	unc-5 netrin receptor A	protein_coding	107448	-
ENSMUSG00000025892	27.16	5.11	2.353	2.58031E-08	7.38304E-07	Gria4	glutamate receptor, ionotropic, AMPA4 (alpha 4)	protein_coding	14802	-

ENSMUSG00000 025899	9.29	0.149	-2.749	0.000364783	0.001850065	Alkbh8	alkB homolog 8, tRNA methyltransferase	protein_coding	67667	-
ENSMUSG00000 025900	27.37	6.386	2.675	6.30056E-08	1.52375E-06	Rp1	retinitis pigmentosa 1 (human)	protein_coding	19888	-
ENSMUSG00000 025902	3.32	7.191	2.846	0.006541262	0.019309994	Sox17	SRY (sex determining region Y)-box 17	protein_coding	20671	HMG
ENSMUSG00000 025946	9.95	8.763	3.131	2.40906E-05	0.00020643	Pth2r	parathyroid hormone 2 receptor	protein_coding	213527	-
ENSMUSG00000 025962	8.57	0.199	-2.326	0.003255463	0.010983367	Fastkd2	FAST kinase domains 2	protein_coding	75619	-
ENSMUSG00000 025968	29.31	0.214	-2.224	3.94749E-06	4.70999E-05	Ndufs1	NADH:ubiquinone oxidoreductase core subunit S1	protein_coding	227197	-
ENSMUSG00000 026012	50.16	0.177	-2.502	1.74818E-11	1.65201E-09	Cd28	CD28 antigen	protein_coding	12487	-
ENSMUSG00000 026023	10.18	4.433	2.148	0.005427129	0.016601987	Cdk15	cyclin-dependent kinase 15	protein_coding	271697	-
ENSMUSG00000 026062	14.48	4.057	2.02	0.000606748	0.00280418	Slc9a2	solute carrier family 9 (sodium/hydrogen exchanger), member 2	protein_coding	226999	-
ENSMUSG00000 026073	88.19	4.855	2.28	2.42301E-06	3.13908E-05	Il1r2	interleukin 1 receptor, type II	protein_coding	16178	-
ENSMUSG00000 026077	18.42	6.772	2.76	1.4502E-08	4.62596E-07	Npas2	neuronal PAS domain protein 2	protein_coding	18143	bHLH
ENSMUSG00000 026078	17.3	0.192	-2.379	1.03504E-05	0.000102529	Pdcl3	phosducin-like 3	protein_coding	68833	-
ENSMUSG00000 026090	13.61	4.555	2.187	0.001688693	0.00646739	2010300C02R ik	RIKEN cDNA 2010300C02 gene	protein_coding	72097	-
ENSMUSG00000 026147	7.47	4.014	2.005	0.009559713	0.026153851	Col9a1	collagen, type IX, alpha 1	protein_coding	12839	-
ENSMUSG00000 026149	6.66	8.058	3.01	3.75457E-05	0.000295255	Tm4sf20	transmembrane 4 L six family member 20	protein_coding	66261	-
ENSMUSG00000 026180	623.59	4.252	2.088	7.15588E-09	2.62416E-07	Cxcr2	chemokine (C-X-C motif) receptor 2	protein_coding	12765	-
ENSMUSG00000 026181	101.62	0.056	-4.163	1.53553E-06	2.15394E-05	Ppm1f	protein phosphatase 1F (PP2C domain containing)	protein_coding	68606	-
ENSMUSG00000 026192	162.15	8.056	3.01	0.006372942	0.018928165	Atic	5-aminoimidazole-4-carboxamide ribonucleotide formyltransferase/IMP cyclohydrolase	protein_coding	108147	-
ENSMUSG00000 026204	51.13	5.905	2.562	1.38661E-07	2.93821E-06	Ptprn	protein tyrosine phosphatase, receptor type, N	protein_coding	19275	-
ENSMUSG00000 026207	18.54	5.407	2.435	2.52595E-06	3.24437E-05	Speg	SPEG complex locus	protein_coding	11790	-
ENSMUSG00000 026227	5.91	15.507	3.955	9.7655E-06	9.78674E-05	2810459M11 Rik	RIKEN cDNA 2810459M11 gene	protein_coding	72792	-
ENSMUSG00000 026234	133.56	0.22	-2.185	5.32765E-09	2.07663E-07	Ncl	nucleolin	protein_coding	17975	-
ENSMUSG00000 026238	548.77	0.214	-2.221	6.78667E-10	3.70214E-08	Ptma	prothymosin alpha	protein_coding	19231	-

ENSMUSG00000 026247	6.34	9.732	3.283	3.61378E-05	0.000287328	Ecel1	endothelin converting enzyme-like 1	protein_coding	13599	-
ENSMUSG00000 026248	10.04	0.006	-7.315	1.30172E-08	4.26057E-07	Mrpl44	mitochondrial ribosomal protein L44	protein_coding	69163	-
ENSMUSG00000 026255	6.43	14.004	3.808	5.92926E-06	6.51538E-05	Efhd1	EF hand domain containing 1	protein_coding	98363	-
ENSMUSG00000 026270	11.49	0.089	-3.497	1.61939E-06	2.25397E-05	Capn10	calpain 10	protein_coding	23830	-
ENSMUSG00000 026303	5.18	4.047	2.017	0.009289165	0.025557648	Mlph	melanophilin	protein_coding	171531	-
ENSMUSG00000 026321	7.34	5.277	2.4	0.015372229	0.038057809	Tnfrsf11a	tumor necrosis factor receptor superfamily, member 11a, NFKB activator	protein_coding	21934	-
ENSMUSG00000 026344	7.02	5.144	2.363	0.001280301	0.005165186	Lypd1	Ly6/Plaur domain containing 1	protein_coding	72585	-
ENSMUSG00000 026357	1532.44	7.869	2.976	6.64E-11	5.12078E-09	Rgs18	regulator of G-protein signaling 18	protein_coding	64214	-
ENSMUSG00000 026377	19.2	0.121	-3.045	5.20208E-09	2.04436E-07	Nifk	nucleolar protein interacting with the FHA domain of MKI67	protein_coding	67949	-
ENSMUSG00000 026387	7.2	4.367	2.127	0.006834944	0.019976759	Sctr	secretin receptor	protein_coding	319229	-
ENSMUSG00000 026399	90.35	0.124	-3.011	5.32927E-16	1.65106E-13	Cd55	CD55 molecule, decay accelerating factor for complement	protein_coding	13136	-
ENSMUSG00000 026407	10.15	4.47	2.16	0.004016965	0.013014938	Cacna1s	calcium channel, voltage-dependent, L type, alpha 1S subunit	protein_coding	12292	-
ENSMUSG00000 026442	26.71	4.822	2.27	6.90148E-07	1.11522E-05	Nfasc	neurofascin	protein_coding	269116	-
ENSMUSG00000 026447	284.34	4.141	2.05	8.2455E-08	1.89467E-06	Pik3c2b	phosphatidylinositol-4-phosphate 3-kinase catalytic subunit type 2 beta	protein_coding	240752	-
ENSMUSG00000 026468	11.52	4.059	2.021	0.006381121	0.018933681	Lhx4	LIM homeobox protein 4	protein_coding	16872	Homeobox
ENSMUSG00000 026479	6.05	5.539	2.47	0.001256159	0.005086058	Lamc2	laminin, gamma 2	protein_coding	16782	-
ENSMUSG00000 026494	41.03	4.62	2.208	3.65836E-07	6.61338E-06	Kif26b	kinesin family member 26B	protein_coding	269152	-
ENSMUSG00000 026496	55.51	0.169	-2.568	2.17369E-08	6.41364E-07	Parp1	poly (ADP-ribose) polymerase family, member 1	protein_coding	11545	-
ENSMUSG00000 026504	764.86	4.894	2.291	1.05856E-06	1.58248E-05	Sdccag8	serologically defined colon cancer antigen 8	protein_coding	76816	-
ENSMUSG00000 026520	18.28	0.172	-2.537	3.59601E-07	6.52693E-06	Pycr2	pyrroline-5-carboxylate reductase family, member 2	protein_coding	69051	-
ENSMUSG00000 026523	10.84	4.18	2.063	0.001587744	0.006154051	Wdr64	WD repeat domain 64	protein_coding	75820	-
ENSMUSG00000 026527	26.68	5.187	2.375	8.62049E-08	1.97076E-06	Rgs7	regulator of G protein signaling 7	protein_coding	24012	-
ENSMUSG00000 026535	28.87	0.166	-2.592	0.001375584	0.005472187	Ifi202b	interferon activated gene 202B	protein_coding	26388	-
ENSMUSG00000 026536	35.98	0.236	-2.08	1.31582E-06	1.89151E-05	Ifi211	interferon activated gene 211	protein_coding	381308	-

ENSMUSG00000026579	116.72	4.337	2.117	1.57695E-09	7.70005E-08	F5	coagulation factor V	protein_coding	14067	-
ENSMUSG00000026586	5.03	4.004	2.002	0.007312874	0.021099073	Prrx1	paired related homeobox 1	protein_coding	18933	Homeobox
ENSMUSG00000026608	15.81	0.183	-2.45	8.25888E-05	0.000558539	Kctd3	potassium 44aladin44izationrisation domain containing 3	protein_coding	226823	-
ENSMUSG00000026609	63.15	4.333	2.116	2.91502E-10	1.71229E-08	Ush2a	usherin	protein_coding	22283	-
ENSMUSG00000026610	61.79	4.402	2.138	4.35977E-07	7.70312E-06	Esrrg	estrogen-related receptor gamma	protein_coding	26381	ESR-like
ENSMUSG00000026616	31.23	0.115	-3.116	4.92447E-12	5.53049E-10	Cr2	complement receptor 2	protein_coding	12902	-
ENSMUSG00000026620	11.92	4.541	2.183	0.014957415	0.037225732	Mark1	MAP/microtubule affinity regulating kinase 1	protein_coding	226778	-
ENSMUSG00000026640	20.63	5.577	2.48	8.98869E-06	9.16153E-05	Plxna2	plexin A2	protein_coding	18845	-
ENSMUSG00000026676	10.57	4.485	2.165	0.000178747	0.001041474	Ccdc3	coiled-coil domain containing 3	protein_coding	74186	-
ENSMUSG00000026686	14.26	5.251	2.393	0.001615909	0.006238991	Lmx1a	LIM homeobox transcription factor 1 alpha	protein_coding	110648	Homeobox
ENSMUSG00000026696	31.67	0.204	-2.29	6.1282E-08	1.4882E-06	Vamp4	vesicle-associated membrane protein 4	protein_coding	53330	-
ENSMUSG00000026701	603.92	4.162	2.057	4.57308E-07	8.00133E-06	Prdx6	peroxiredoxin 6	protein_coding	11758	-
ENSMUSG00000026712	10.09	4.53	2.179	0.000136251	0.000840473	Mrc1	mannose receptor, C type 1	protein_coding	17533	-
ENSMUSG00000026726	16.02	5.508	2.462	0.00014339	0.000875792	Cubn	cubilin (intrinsic factor-cobalamin receptor)	protein_coding	65969	-
ENSMUSG00000026755	97.48	0.208	-2.263	7.54771E-08	1.76595E-06	Arpc5l	actin related protein 2/3 complex, subunit 5-like	protein_coding	74192	-
ENSMUSG00000026764	26.78	4.46	2.157	1.86545E-05	0.000167937	Kif5c	kinesin family member 5C	protein_coding	16574	-
ENSMUSG00000026805	8.4	6.188	2.629	0.002108052	0.007749506	Barhl1	BarH like homeobox 1	protein_coding	54422	Homeobox
ENSMUSG00000026815	59.1	5.045	2.335	3.606E-05	0.000286963	Gfi1b	growth factor independent 1B	protein_coding	14582	zf-C2H2
ENSMUSG00000026817	6.46	7.401	2.888	0.001496417	0.005859474	Ak1	adenylate kinase 1	protein_coding	11636	-
ENSMUSG00000026824	7.28	5.374	2.426	0.000132698	0.00082194	Kcnj3	potassium inwardly-rectifying channel, subfamily J, member 3	protein_coding	16519	-
ENSMUSG00000026836	19.09	6.115	2.612	8.5568E-06	8.8417E-05	Acvr1	activin A receptor, type 1	protein_coding	11477	-
ENSMUSG00000026840	9.22	7.398	2.887	0.00066322	0.003014773	Lamc3	laminin gamma 3	protein_coding	23928	-
ENSMUSG00000026848	31.14	0.198	-2.337	2.51876E-07	4.85098E-06	Tor1b	torsin family 1, member B	protein_coding	30934	-
ENSMUSG00000026874	9.34	7.322	2.872	0.003316204	0.011158964	Hc	hemolytic complement	protein_coding	15139	-

ENSMUSG00000026883	36.76	4.019	2.007	1.79825E-07	3.64293E-06	Dab2ip	disabled 2 interacting protein	protein_coding	69601	-
ENSMUSG00000026904	27.41	4.382	2.132	3.81195E-06	4.57561E-05	Slc4a10	solute carrier family 4, sodium bicarbonate cotransporter-like, member 10	protein_coding	94229	-
ENSMUSG00000026919	3.51	0.163	-2.619	0.006429817	0.019037302	Lcn4	lipocalin 4	protein_coding	16821	-
ENSMUSG00000026941	10.66	0.206	-2.278	0.000802183	0.00352431	Mamdc4	MAM domain containing 4	protein_coding	381352	-
ENSMUSG00000026975	12.25	0.103	-3.286	4.01078E-07	7.16398E-06	Dph7	diphthamine biosynethesis 7	protein_coding	67228	-
ENSMUSG00000026986	15.06	8.923	3.158	5.57954E-07	9.39632E-06	Hnmt	histamine N-methyltransferase	protein_coding	140483	-
ENSMUSG00000026989	30.02	0.121	-3.052	3.65039E-07	6.60562E-06	Dapl1	death associated protein-like 1	protein_coding	76747	-
ENSMUSG00000027002	81.26	5.498	2.459	4.79484E-10	2.69246E-08	Nckap1	NCK-associated protein 1	protein_coding	50884	-
ENSMUSG00000027048	10.12	6.156	2.622	6.26949E-05	0.000445811	Abcb11	ATP-binding cassette, sub-family B (MDR/TAP), member 11	protein_coding	27413	-
ENSMUSG00000027067	45.45	0.092	-3.443	1.02049E-11	1.036E-09	Ssrp1	structure specific recognition protein 1	protein_coding	20833	HMG
ENSMUSG00000027102	7.75	13.106	3.712	0.00032886	0.001707059	Hoxd8	homeobox D8	protein_coding	15437	Homeobox
ENSMUSG00000027164	25.8	0.18	-2.476	2.57043E-05	0.000217765	Traf6	TNF receptor-associated factor 6	protein_coding	22034	-
ENSMUSG00000027165	12.91	4.039	2.014	0.000665276	0.003018816	B230118H07Rik	RIKEN cDNA B230118H07 gene	protein_coding	68170	-
ENSMUSG00000027168	6.12	4.102	2.036	0.002449707	0.008725739	Pax6	paired box 6	protein_coding	18508	PAX
ENSMUSG00000027170	73.63	0.091	-3.465	9.82666E-18	4.01307E-15	Eif3m	eukaryotic translation initiation factor 3, subunit M	protein_coding	98221	-
ENSMUSG00000027173	7.29	4.041	2.015	0.002742474	0.009548443	Depdc7	DEP domain containing 7	protein_coding	211896	-
ENSMUSG00000027210	24.41	4.509	2.173	1.94714E-06	2.61908E-05	Meis2	Meis homeobox 2	protein_coding	17536	Homeobox
ENSMUSG00000027227	481.78	10.495	3.392	1.78623E-13	3.24209E-11	Sord	sorbitol dehydrogenase	protein_coding	20322	-
ENSMUSG00000027238	36.95	4.048	2.017	4.06785E-08	1.07126E-06	Frmd5	FERM domain containing 5	protein_coding	228564	-
ENSMUSG00000027239	9.7	6.135	2.617	0.005562955	0.016953573	Mdk	midkine	protein_coding	17242	-
ENSMUSG00000027286	7.04	0.199	-2.332	0.002615579	0.009190328	Lrrc57	leucine rich repeat containing 57	protein_coding	66606	-
ENSMUSG00000027287	1085.94	4.764	2.252	1.08665E-06	1.61916E-05	Snap23	synaptosomal-associated protein 23	protein_coding	20619	-
ENSMUSG00000027356	5.19	4.308	2.107	0.006294999	0.018743138	Fermt1	fermitin family member 1	protein_coding	241639	-
ENSMUSG00000027358	9.42	5.137	2.361	0.000337576	0.001742072	Bmp2	bone morphogenetic protein 2	protein_coding	12156	-

ENSMUSG00000027359	18.53	7.329	2.874	3.88641E-06	4.64945E-05	Slc27a2	solute carrier family 27 (fatty acid transporter), member 2	protein_coding	26458	-
ENSMUSG00000027368	42.98	0.105	-3.256	3.47397E-14	7.61265E-12	Dusp2	dual specificity phosphatase 2	protein_coding	13537	-
ENSMUSG00000027378	6.58	7.619	2.93	4.87941E-05	0.000366088	Nphp1	nephronophthisis 1 (juvenile) homolog (human)	protein_coding	53885	-
ENSMUSG00000027386	5.46	6.004	2.586	0.006399426	0.018969198	Fbln7	fibulin 7	protein_coding	70370	-
ENSMUSG00000027406	42.97	0.095	-3.395	4.47361E-11	3.65392E-09	Idh3b	isocitrate dehydrogenase 3 (NAD+) beta	protein_coding	170718	-
ENSMUSG00000027411	41.59	0.217	-2.202	4.62969E-07	8.09251E-06	Vps16	VSP16 CORVET/HOPS core subunit	protein_coding	80743	-
ENSMUSG00000027420	9.37	4.826	2.271	0.000943642	0.004020122	Bfsp1	beaded filament structural protein 1, in lens-CP94	protein_coding	12075	-
ENSMUSG00000027434	5.68	4.441	2.151	0.014907961	0.03713866	Nkx2-2	NK2 homeobox 2	protein_coding	18088	Homeobox
ENSMUSG00000027452	52.6	0.213	-2.234	5.8747E-10	3.21837E-08	Acss1	acyl-CoA synthetase short-chain family member 1	protein_coding	68738	-
ENSMUSG00000027488	10.24	4.577	2.194	0.00573294	0.017389465	Snta1	syntrophin, acidic 1	protein_coding	20648	-
ENSMUSG00000027509	23.79	0.209	-2.259	1.86573E-06	2.53213E-05	Rae1	ribonucleic acid export 1	protein_coding	66679	-
ENSMUSG00000027520	9.39	5.234	2.388	0.002450253	0.008725739	Zdbf2	zinc finger, DBF-type containing 2	protein_coding	73884	-
ENSMUSG00000027582	93.33	0.248	-2.014	6.5328E-07	1.07106E-05	Zgpat	zinc finger, CCCH-type with G patch domain	protein_coding	229007	-
ENSMUSG00000027583	10.77	4.327	2.113	0.004579182	0.014429639	Zbtb46	zinc finger and BTB domain containing 46	protein_coding	72147	ZBTB
ENSMUSG00000027618	22.41	0.189	-2.407	4.186E-06	4.93558E-05	Nfs1	nitrogen fixation gene 1 (S. cerevisiae)	protein_coding	18041	-
ENSMUSG00000027624	24.69	5.749	2.523	2.18188E-06	2.8763E-05	Epb41l1	erythrocyte membrane protein band 4.1 like 1	protein_coding	13821	-
ENSMUSG00000027667	16.18	0.146	-2.776	5.78063E-05	0.000418502	Zfp639	zinc finger protein 639	protein_coding	67778	zf-C2H2
ENSMUSG00000027673	29.31	0.244	-2.035	9.64589E-06	9.68848E-05	Ndufb5	NADH:ubiquinone oxidoreductase subunit B5	protein_coding	66046	-
ENSMUSG00000027674	18.21	4.244	2.085	0.000460745	0.002247321	Pex5l	peroxisomal biogenesis factor 5-like	protein_coding	58869	-
ENSMUSG00000027716	7.95	4.14	2.05	0.013516098	0.034348852	Trpc3	transient receptor potential cation channel, subfamily C, member 3	protein_coding	22065	-
ENSMUSG00000027737	44.46	4.475	2.162	1.09893E-06	1.63061E-05	Slc7a11	solute carrier family 7 (cationic amino acid transporter, y+ system), member 11	protein_coding	26570	-
ENSMUSG00000027752	28.9	0.244	-2.037	7.13253E-06	7.58369E-05	Exosc8	exosome component 8	protein_coding	69639	-
ENSMUSG00000027765	74.58	6.188	2.629	3.48824E-10	2.00256E-08	P2ry1	purinergic receptor P2Y, G-protein coupled 1	protein_coding	18441	-
ENSMUSG00000027787	14.96	0.197	-2.347	2.83388E-05	0.00023575	Nmd3	NMD3 ribosome export adaptor	protein_coding	97112	-

ENSMUSG00000027796	7.87	4.505	2.172	0.000711054	0.003190283	Smad9	SMAD family member 9	protein_coding	55994	MH1
ENSMUSG00000027797	27.62	4.197	2.069	1.46892E-05	0.000137833	Dclk1	doublecortin-like kinase 1	protein_coding	13175	-
ENSMUSG00000027803	21.13	6.465	2.693	2.80349E-08	7.88305E-07	Wwtr1	WW domain containing transcription regulator 1	protein_coding	97064	-
ENSMUSG00000027809	16.76	0.193	-2.374	4.17906E-06	4.93063E-05	Etfdh	electron transferring flavoprotein, dehydrogenase	protein_coding	66841	-
ENSMUSG00000027855	6.26	5.564	2.476	0.000416186	0.002065869	Sycp1	synaptonemal complex protein 1	protein_coding	20957	-
ENSMUSG00000027863	157.9	0.148	-2.754	5.4452E-15	1.46037E-12	Cd2	CD2 antigen	protein_coding	12481	-
ENSMUSG00000027868	9.89	5.349	2.419	0.000421792	0.002090234	Tbx15	T-box 15	protein_coding	21384	T-box
ENSMUSG00000027875	5.13	4.761	2.251	0.017684589	0.0426991	Hmgcs2	3-hydroxy-3-methylglutaryl-Coenzyme A synthase 2	protein_coding	15360	-
ENSMUSG00000027894	10.18	9.34	3.223	0.00084744	0.0036874	Slc6a17	solute carrier family 6 (neurotransmitter transporter), member 17	protein_coding	229706	-
ENSMUSG00000027953	34.45	0.117	-3.094	4.31536E-12	4.90776E-10	Slc50a1	solute carrier family 50 (sugar transporter), member 1	protein_coding	19729	-
ENSMUSG00000027966	15.14	4.718	2.238	0.000105772	0.000684685	Col11a1	collagen, type XI, alpha 1	protein_coding	12814	-
ENSMUSG00000027977	22.2	4.277	2.096	1.01229E-05	0.000100545	Ndst3	N-deacetylase/N-sulfotransferase (heparan glucosaminyl) 3	protein_coding	83398	-
ENSMUSG00000028015	36.01	0.209	-2.256	1.69197E-08	5.24665E-07	Ctso	cathepsin O	protein_coding	229445	-
ENSMUSG00000028017	11.04	4.17	2.06	0.000759023	0.003360986	Egf	epidermal growth factor	protein_coding	13645	-
ENSMUSG00000028020	8.57	4.063	2.023	0.013655998	0.034629499	Glrb	glycine receptor, beta subunit	protein_coding	14658	-
ENSMUSG00000028023	5.38	4.381	2.131	0.001893538	0.007092971	Pitx2	paired-like homeodomain transcription factor 2	protein_coding	18741	Homeobox
ENSMUSG00000028034	36.46	0.196	-2.351	8.94295E-08	2.02899E-06	Fubp1	far upstream element (FUSE) binding protein 1	protein_coding	51886	-
ENSMUSG00000028063	130.74	4.418	2.143	1.92876E-06	2.60194E-05	Lmna	lamin A	protein_coding	16905	-
ENSMUSG00000028071	36.6	0.209	-2.256	5.87136E-07	9.78126E-06	Sh2d2a	SH2 domain containing 2A	protein_coding	27371	-
ENSMUSG00000028076	11.1	0.159	-2.652	0.000332358	0.001721572	Cd1d1	CD1d1 antigen	protein_coding	12479	-
ENSMUSG00000028096	11.93	0.22	-2.182	0.002862534	0.009892016	Gpr89	G protein-coupled receptor 89	protein_coding	67549	-
ENSMUSG00000028108	459.31	6.327	2.661	5.3134E-09	2.07558E-07	Ecm1	extracellular matrix protein 1	protein_coding	13601	-
ENSMUSG00000028114	9.81	0.135	-2.891	7.06961E-05	0.000493144	Mettl14	methyltransferase like 14	protein_coding	210529	-
ENSMUSG00000028132	17.36	5.428	2.44	0.000177827	0.001037123	Tmem56	transmembrane protein 56	protein_coding	99887	-

ENSMUSG00000028140	28.99	0.122	-3.031	2.93376E-11	2.51032E-09	Mrpl9	mitochondrial ribosomal protein L9	protein_coding	78523	-
ENSMUSG00000028182	7.91	5.159	2.367	0.001665358	0.006396926	Lrriq3	leucine-rich repeats and IQ motif containing 3	protein_coding	74435	-
ENSMUSG00000028184	22.24	4.286	2.1	7.98637E-05	0.000543382	Adgrl2	adhesion G protein-coupled receptor L2	protein_coding	99633	-
ENSMUSG00000028185	4.53	4.121	2.043	0.011670485	0.03065891	Dnase2b	deoxyribonuclease II beta	protein_coding	56629	-
ENSMUSG00000028214	3.76	0.149	-2.746	0.003534138	0.011729762	Gem	GTP binding protein (gene overexpressed in skeletal muscle)	protein_coding	14579	-
ENSMUSG00000028217	5.89	5.108	2.353	0.003138	0.010659827	Cdh17	cadherin 17	protein_coding	12557	-
ENSMUSG00000028234	1057.94	0.228	-2.131	1.60853E-08	5.01831E-07	Rps20	ribosomal protein S20	protein_coding	67427	-
ENSMUSG00000028246	9.45	4.497	2.169	0.000451086	0.002211612	Faxc	failed axon connections homolog	protein_coding	76132	-
ENSMUSG00000028261	5.99	0.242	-2.047	0.005276876	0.016241895	Ndufaf4	NADH:ubiquinone oxidoreductase complex assembly factor 4	protein_coding	68493	-
ENSMUSG00000028327	4.24	4.342	2.118	0.019423698	0.046002957	Stra6l	STRA6-like	protein_coding	74152	-
ENSMUSG00000028358	19.17	4.548	2.185	0.000933064	0.003982477	Zfp618	zinc finger protein 618	protein_coding	72701	zf-C2H2
ENSMUSG00000028364	9.94	4.069	2.025	0.007863489	0.022360979	Tnc	tenascin C	protein_coding	21923	-
ENSMUSG00000028389	6.79	4.678	2.226	0.018559852	0.044425467	Zfp37	zinc finger protein 37	protein_coding	22696	zf-C2H2
ENSMUSG00000028412	348.13	5.316	2.41	4.73449E-09	1.88634E-07	Slc44a1	solute carrier family 44, member 1	protein_coding	100434	-
ENSMUSG00000028423	50.16	0.203	-2.301	1.01051E-09	5.17318E-08	Nfx1	nuclear transcription factor, X-box binding 1	protein_coding	74164	zf-NF-X1
ENSMUSG00000028434	17.05	4.026	2.009	0.000255885	0.001391644	Epb41l4b	erythrocyte membrane protein band 4.1 like 4b	protein_coding	54357	-
ENSMUSG00000028444	14.62	6.749	2.755	7.7405E-06	8.11488E-05	Cntfr	ciliary neurotrophic factor receptor	protein_coding	12804	-
ENSMUSG00000028454	18.47	0.234	-2.094	2.25788E-05	0.000195527	Pigo	phosphatidylinositol glycan anchor biosynthesis, class O	protein_coding	56703	-
ENSMUSG00000028459	77.93	0.13	-2.938	9.95419E-11	7.15467E-09	Cd72	CD72 antigen	protein_coding	12517	-
ENSMUSG00000028460	15.26	0.248	-2.011	0.000908054	0.003895158	Sit1	suppression inducing transmembrane adaptor 1	protein_coding	54390	-
ENSMUSG00000028461	13.24	0.185	-2.437	1.9775E-05	0.000176259	Ccdc107	coiled-coil domain containing 107	protein_coding	622404	-
ENSMUSG00000028495	1093.68	0.244	-2.036	7.35365E-08	1.73103E-06	Rps6	ribosomal protein S6	protein_coding	20104	-
ENSMUSG00000028497	701	5.015	2.326	1.08712E-07	2.39486E-06	Hacd4	3-hydroxyacyl-CoA dehydratase 4	protein_coding	66775	-
ENSMUSG00000028518	19.49	7.366	2.881	2.38905E-08	6.93518E-07	Prkaa2	protein kinase, AMP-activated, alpha 2 catalytic subunit	protein_coding	108079	-

ENSMUSG00000028527	9.46	5.632	2.494	0.000116971	0.000739048	Ak4	adenylate kinase 4	protein_coding	11639	-
ENSMUSG00000028546	18.35	4.831	2.272	0.000203365	0.00115678	Elavl4	ELAV like RNA binding protein 4	protein_coding	15572	-
ENSMUSG00000028560	28.67	0.176	-2.505	4.48464E-07	7.89578E-06	Usp1	ubiquitin specific peptidase 1	protein_coding	230484	-
ENSMUSG00000028571	6.68	4.473	2.161	0.011618377	0.030557759	Cyp2j13	cytochrome P450, family 2, subfamily j, polypeptide 13	protein_coding	230459	-
ENSMUSG00000028573	29.85	4.246	2.086	1.73878E-07	3.54242E-06	Fggy	FGGY carbohydrate kinase domain containing	protein_coding	75578	-
ENSMUSG00000028609	27.85	0.206	-2.282	1.86485E-07	3.76935E-06	Magoh	mago homolog, exon junction complex core component	protein_coding	17149	-
ENSMUSG00000028629	14.3	0.145	-2.791	6.02838E-05	0.000432259	Exo5	exonuclease 5	protein_coding	73172	-
ENSMUSG00000028649	427.48	0.219	-2.193	5.09678E-09	2.00842E-07	Macf1	microtubule-actin crosslinking factor 1	protein_coding	11426	-
ENSMUSG00000028651	21.32	0.235	-2.089	7.41448E-05	0.000511836	Ppie	peptidylprolyl isomerase E (cyclophilin E)	protein_coding	56031	-
ENSMUSG00000028661	11.13	5.58	2.48	0.000161664	0.000961584	Epha8	Eph receptor A8	protein_coding	13842	-
ENSMUSG00000028664	92.65	4.267	2.093	9.20772E-06	9.33037E-05	Ephb2	Eph receptor B2	protein_coding	13844	-
ENSMUSG00000028707	6.55	9.411	3.234	0.000591414	0.002750288	Dmbx1	diencephalon/mesencephalon homeobox 1	protein_coding	140477	Homeobox
ENSMUSG00000028716	201.45	4.727	2.241	9.23852E-07	1.42101E-05	Pdzk1ip1	PDZK1 interacting protein 1	protein_coding	67182	-
ENSMUSG00000028717	163.02	5.369	2.425	1.4908E-10	9.88496E-09	Tal1	T cell acute lymphocytic leukemia 1	protein_coding	21349	bHLH
ENSMUSG00000028736	12.4	4	2	0.003637457	0.012028241	Pax7	paired box 7	protein_coding	18509	PAX
ENSMUSG00000028780	15.04	7.356	2.879	8.3492E-07	1.30345E-05	Sema3c	sema domain, immunoglobulin domain (Ig), short basic domain, sec49aladin49izamaphorin) 3C	protein_coding	20348	-
ENSMUSG00000028790	72.95	0.249	-2.003	5.7984E-10	3.18629E-08	Khdrbs1	KH domain containing, RNA binding, signal transduction associated 1	protein_coding	20218	-
ENSMUSG00000028798	125.03	0.249	-2.008	7.21145E-09	2.63635E-07	Eif3i	eukaryotic translation initiation factor 3, subunit I	protein_coding	54709	-
ENSMUSG00000028801	15	5.345	2.418	0.000244127	0.001341324	Stpg1	sperm tail PG rich repeat containing 1	protein_coding	78806	-
ENSMUSG00000028833	20.53	0.239	-2.064	3.06061E-05	0.000249983	Ncdn	neurochondrin	protein_coding	26562	-
ENSMUSG00000028843	1441.92	3.999	2	8.82313E-07	1.36557E-05	Sh3bgrl3	SH3 domain binding glutamic acid-rich protein-like 3	protein_coding	73723	-
ENSMUSG00000028847	24.31	0.218	-2.197	6.20872E-06	6.78204E-05	Trappc3	trafficking protein particle complex 3	protein_coding	27096	-
ENSMUSG00000028871	4.43	4.649	2.217	0.003337974	0.011202848	Rspo1	R-spondin 1	protein_coding	192199	-

ENSMUSG00000 028884	11.81	0.129	-2.951	1.59497E-05	0.000147353	Rpa2	replication protein A2	protein_coding	19891	-
ENSMUSG00000 028893	49.06	4.21	2.074	1.20602E-08	4.01314E-07	Sesn2	sestrin 2	protein_coding	230784	-
ENSMUSG00000 028936	355.72	0.157	-2.675	3.4738E-10	2.00066E-08	Rpl22	ribosomal protein L22	protein_coding	19934	-
ENSMUSG00000 028958	14.41	0.141	-2.829	2.67147E-06	3.39729E-05	Tmub1	transmembrane and ubiquitin-like domain containing 1	protein_coding	64295	-
ENSMUSG00000 028978	3.86	8.401	3.071	0.003720846	0.012231716	Nos3	nitric oxide synthase 3, endothelial cell	protein_coding	18127	-
ENSMUSG00000 029005	85.16	4.875	2.285	1.55099E-05	0.000143955	Draxin	dorsal inhibitory axon guidance protein	protein_coding	70433	-
ENSMUSG00000 029022	19.88	0.224	-2.158	6.28652E-05	0.000446668	Miip	migration and invasion inhibitory protein	protein_coding	28010	-
ENSMUSG00000 029027	16.27	0.196	-2.348	4.40026E-05	0.000336704	Dffb	DNA fragmentation factor, beta subunit	protein_coding	13368	-
ENSMUSG00000 029084	37.03	0.093	-3.432	6.90765E-11	5.30443E-09	Cd38	CD38 antigen	protein_coding	12494	-
ENSMUSG00000 029093	49.74	4.344	2.119	2.69155E-06	3.41556E-05	Sorcs2	sortilin-related VPS10 domain containing receptor 2	protein_coding	81840	-
ENSMUSG00000 029095	18.17	4.168	2.059	0.003427187	0.011433926	Ablim2	actin-binding LIM protein 2	protein_coding	231148	-
ENSMUSG00000 029096	6.52	9.28	3.214	0.000975984	0.004138146	Htra3	HtrA serine peptidase 3	protein_coding	78558	-
ENSMUSG00000 029097	46.93	0.07	-3.841	0.000842587	0.003671302	Trmt44	tRNA methyltransferase 44	protein_coding	78890	-
ENSMUSG00000 029134	10.82	4.377	2.13	0.002213626	0.008065015	Plb1	phospholipase B1	protein_coding	665270	-
ENSMUSG00000 029189	12.06	4.112	2.04	0.010370633	0.027942705	Sel1l3	sel-1 suppressor of lin-12-like 3 (C. elegans)	protein_coding	231238	-
ENSMUSG00000 029204	86.46	0.209	-2.262	2.49432E-08	7.16147E-07	Rhoh	ras homolog family member H	protein_coding	74734	-
ENSMUSG00000 029207	35.35	4.473	2.161	1.95969E-06	2.62985E-05	Apbb2	amyloid beta (A4) precursor protein-binding, family B, member 2	protein_coding	11787	-
ENSMUSG00000 029211	6.27	5.184	2.374	0.001635142	0.006301065	Gabra4	gamma-aminobutyric acid (GABA) A receptor, subunit alpha 4	protein_coding	14397	-
ENSMUSG00000 029247	26.67	0.179	-2.483	4.75006E-08	1.20556E-06	Paics	phosphoribosylaminoimidazole carboxylase, phosphoribosylaminoribosylaminoimidazole, succinocarboxamide synthetase	protein_coding	67054	-
ENSMUSG00000 029267	24.09	0.208	-2.269	1.6268E-06	2.26157E-05	Mtf2	metal response element binding transcription factor 2	protein_coding	17765	-
ENSMUSG00000 029309	7.69	5.567	2.477	0.000481823	0.002328011	Sparcl1	SPARC-like 1	protein_coding	13602	-
ENSMUSG00000 029330	23.06	4.584	2.197	3.84764E-06	4.611E-05	Cds1	CDP-diacylglycerol synthase 1	protein_coding	74596	-
ENSMUSG00000 029334	15.7	5.011	2.325	0.000183174	0.001063474	Prkg2	protein kinase, cGMP-dependent, type II	protein_coding	19092	-

ENSMUSG00000029360	7.99	0.218	-2.194	0.002946069	0.01012197	Gm9754	-	antisense_RNA	-	-
ENSMUSG00000029361	35.94	4.736	2.244	2.12589E-06	2.8109E-05	Nos1	nitric oxide synthase 1, neuronal	protein_coding	18125	-
ENSMUSG00000029372	3832.88	4.516	2.175	9.88537E-06	9.86814E-05	Ppbp	pro-platelet basic protein	protein_coding	57349	-
ENSMUSG00000029373	2716.89	4.927	2.301	2.99801E-06	3.73847E-05	Pf4	platelet factor 4	protein_coding	56744	-
ENSMUSG00000029376	17.63	4.897	2.292	0.000113157	0.000719959	Mthfd2l	methylenetetrahydrofolate dehydrogenase (NADP+ dependent) 2-like	protein_coding	665563	-
ENSMUSG00000029420	27.89	5.982	2.581	2.8861E-05	0.000239098	Rimbp2	RIMS binding protein 2	protein_coding	231760	-
ENSMUSG00000029447	40.93	0.229	-2.126	1.45809E-07	3.06796E-06	Cct6a	chaperonin containing Tcp1, subunit 6a (zeta)	processed_transcript	12466	-
ENSMUSG00000029469	12.1	7.083	2.824	4.76319E-06	5.469E-05	Ift81	intraflagellar transport 81	protein_coding	12589	-
ENSMUSG00000029484	8.15	5.614	2.489	0.001067858	0.004449987	Anxa3	annexin A3	protein_coding	11745	-
ENSMUSG00000029521	18.55	4.343	2.119	0.000146021	0.000888239	Chek2	checkpoint kinase 2	protein_coding	50883	-
ENSMUSG00000029534	112.98	4.01	2.004	1.25138E-09	6.24612E-08	St7	suppression of tumorigenicity 7	protein_coding	64213	-
ENSMUSG00000029535	117.48	4.676	2.225	3.59647E-09	1.49595E-07	Triap1	TP53 regulated inhibitor of apoptosis 1	protein_coding	69076	-
ENSMUSG00000029559	11.4	0.136	-2.883	1.67239E-05	0.000153244	2210016L21Rik	RIKEN cDNA 2210016L21 gene	protein_coding	72357	-
ENSMUSG00000029560	72.04	0.23	-2.122	2.55065E-11	2.23574E-09	Snx8	sorting nexin 8	protein_coding	231834	-
ENSMUSG00000029564	39.16	4.432	2.148	6.96567E-08	1.66003E-06	4930519G04Rik	RIKEN cDNA 4930519G04 gene	protein_coding	67593	-
ENSMUSG00000029581	8.98	7.119	2.832	0.000267667	0.001442193	Fscn1	fascin actin-bundling protein 1	protein_coding	14086	-
ENSMUSG00000029587	23.86	0.192	-2.378	1.2553E-07	2.68302E-06	Zfp12	zinc finger protein 12	protein_coding	231866	zf-C2H2
ENSMUSG00000029608	11.29	6.2	2.632	7.2576E-05	0.000502573	Rph3a	rabphilin 3A	protein_coding	19894	-
ENSMUSG00000029614	919.74	0.228	-2.13	1.4712E-08	4.67068E-07	Rpl6	ribosomal protein L6	protein_coding	19988	-
ENSMUSG00000029648	25.52	5.083	2.346	0.000106472	0.000688198	Flt1	FMS-like tyrosine kinase 1	protein_coding	14254	-
ENSMUSG00000029710	5.29	4.763	2.252	0.011808057	0.03093439	Ephb4	Eph receptor B4	protein_coding	13846	-
ENSMUSG00000029752	10.79	4.219	2.077	0.010407019	0.028011809	Asns	asparagine synthetase	protein_coding	27053	-
ENSMUSG00000029761	168.61	4.571	2.193	3.05894E-09	1.3213E-07	Cald1	caldesmon 1	protein_coding	109624	-
ENSMUSG00000029778	9.81	5.313	2.41	0.001391586	0.005519789	Adcyap1r1	adenylate cyclase activating polypeptide 1 receptor 1	protein_coding	11517	-

ENSMUSG00000029782	13.54	0.193	-2.371	0.000135089	0.000834734	Tmem209	transmembrane protein 209	protein_coding	72649	-
ENSMUSG00000029797	9.17	6.802	2.766	0.00016481	0.000976095	Sspo	SCO-spondin	protein_coding	243369	-
ENSMUSG00000029802	28.69	4.046	2.016	1.11291E-06	1.64727E-05	Abcg2	ATP binding cassette subfamily G member 2 (Junior blood group)	protein_coding	26357	-
ENSMUSG00000029810	10.68	0.192	-2.378	0.008499147	0.023799464	Tmem176b	transmembrane protein 176B	protein_coding	65963	-
ENSMUSG00000029836	40.76	0.218	-2.196	5.78086E-07	9.6752E-06	Cbx3	chromobox 3	protein_coding	12417	-
ENSMUSG00000029838	9.43	7.846	2.972	0.00029951	0.001579639	Ptn	pleiotrophin	protein_coding	19242	-
ENSMUSG00000029843	6.49	6.221	2.637	0.001079126	0.004486537	Slc13a4	solute carrier family 13 (sodium/sulfate symporters), member 4	protein_coding	243755	-
ENSMUSG00000029847	7.11	5.936	2.569	0.003528361	0.01171708	Slc23a4	solute carrier family 23 member 4	protein_coding	243753	-
ENSMUSG00000029860	1984.09	4.028	2.01	1.74036E-05	0.000158583	Zyx	zyxin	protein_coding	22793	-
ENSMUSG00000029869	9.58	0.241	-2.05	0.002253903	0.008172619	Ephb6	Eph receptor B6	protein_coding	13848	-
ENSMUSG00000029911	20.64	0.18	-2.476	1.87437E-06	2.54002E-05	Ssbp1	single-stranded DNA binding protein 1	protein_coding	381760	-
ENSMUSG00000029923	14.85	0.223	-2.167	0.000113355	0.000720303	Rab19	RAB19, member RAS oncogene family	protein_coding	19331	-
ENSMUSG00000029925	240	4.32	2.111	1.69003E-08	5.24665E-07	Tbxas1	thromboxane A synthase 1, platelet	protein_coding	21391	-
ENSMUSG00000030022	18.83	7.576	2.922	1.3442E-06	1.92769E-05	Adamts9	a disintegrin-like and metallopeptidase (reprolysin type) with thrombospondin type 1 motif, 9	protein_coding	101401	-
ENSMUSG00000030043	14.88	6.987	2.805	2.19574E-05	0.000191539	Tacr1	tachykinin receptor 1	protein_coding	21336	-
ENSMUSG00000030056	35.19	0.197	-2.344	4.88508E-08	1.23287E-06	Isy1	ISY1 splicing factor homolog	protein_coding	57905	-
ENSMUSG00000030062	52.98	0.227	-2.142	1.93399E-08	5.85048E-07	Rpn1	ribophorin I	protein_coding	103963	-
ENSMUSG00000030079	16.75	0.195	-2.358	5.86431E-06	6.46477E-05	Ruvbl1	RuvB-like protein 1	protein_coding	56505	-
ENSMUSG00000030098	12.37	4.426	2.146	0.000220995	0.001237474	Grip2	glutamate receptor interacting protein 2	protein_coding	243547	-
ENSMUSG00000030116	4.64	4.953	2.308	0.013072078	0.033441369	Mfap5	microfibrillar associated protein 5	protein_coding	50530	-
ENSMUSG00000030131	8.63	8.374	3.066	0.000181268	0.001054452	Mug2	murinoglobulin 2	protein_coding	17837	-
ENSMUSG00000030134	13.76	4.217	2.076	0.00258853	0.009114892	Rasgef1a	RasGEF domain family, member 1A	protein_coding	70727	-
ENSMUSG00000030159	1180.56	4.204	2.072	2.49529E-06	3.20958E-05	Clec1b	C-type lectin domain family 1, member b	protein_coding	56760	-

ENSMUSG00000030161	206.46	4.369	2.127	1.77533E-09	8.55253E-08	Gabarapl1	gamma-aminobutyric acid (GABA) A receptor-associated protein-like 1	protein_coding	57436	-
ENSMUSG00000030178	9.68	0.13	-2.944	9.04474E-05	0.000601721	Klra13-ps	killer cell lectin-like receptor subfamily A, member 13, pseudogene	transcribed_unprocessed_pseudogene	16631	-
ENSMUSG00000030200	6.27	8.509	3.089	0.000169607	0.000999566	Bcl2l14	BCL2-like 14 (apoptosis facilitator)	protein_coding	66813	-
ENSMUSG00000030228	32.56	4.269	2.094	0.000220791	0.001237104	Pik3c2g	phosphatidylinositol-4-phosphate 3-kinase catalytic subunit type 2 gamma	protein_coding	18705	-
ENSMUSG00000030243	33.25	0.243	-2.039	0.000113199	0.000719959	Recql	RecQ protein-like	protein_coding	19691	-
ENSMUSG00000030268	8.51	4.609	2.204	0.000829751	0.003621519	Bcat1	branched chain aminotransferase 1, cytosolic	protein_coding	12035	-
ENSMUSG00000030270	6.28	6.315	2.659	0.001886893	0.007073979	Cpne9	copine family member IX	protein_coding	211232	-
ENSMUSG00000030336	66.22	0.143	-2.809	1.01462E-10	7.20621E-09	Cd27	CD27 antigen	protein_coding	21940	-
ENSMUSG00000030340	45.65	4.266	2.093	7.48438E-09	2.72241E-07	Scnn1a	sodium channel, nonvoltage-gated 1 alpha	protein_coding	20276	-
ENSMUSG00000030342	3926.14	5.447	2.445	2.83947E-07	5.38779E-06	Cd9	CD9 antigen	protein_coding	12527	-
ENSMUSG00000030352	175.08	4.37	2.128	2.33562E-08	6.80208E-07	Tspan9	tetraspanin 9	protein_coding	109246	-
ENSMUSG00000030365	39.09	0.192	-2.384	5.87594E-07	9.78126E-06	Clec2i	C-type lectin domain family 2, member i	protein_coding	93675	-
ENSMUSG00000030376	9.46	4.653	2.218	0.00089892	0.003866132	Slc8a2	solute carrier family 8 (sodium/calcium exchanger), member 2	protein_coding	110891	-
ENSMUSG00000030401	7.67	4.401	2.138	0.011269667	0.029850332	Rtn2	reticulon 2 (Z-band associated protein)	protein_coding	20167	-
ENSMUSG00000030411	6.28	4.03	2.011	0.013051468	0.033407668	Nova2	neuro-oncological ventral antigen 2	protein_coding	384569	-
ENSMUSG00000030468	79.76	0.15	-2.741	1.24056E-10	8.50826E-09	Siglecg	sialic acid binding Ig-like lectin G	protein_coding	243958	-
ENSMUSG00000030491	9.82	5.297	2.405	0.000551057	0.002591455	Tdrd12	tudor domain containing 12	protein_coding	71981	-
ENSMUSG00000030500	4.78	7.939	2.989	0.005872286	0.017734303	Slc17a6	solute carrier family 17 (sodium-dependent inorganic phosphate cotransporter), member 6	protein_coding	140919	-
ENSMUSG00000030549	5.11	7.204	2.849	0.000752474	0.003335275	Rhcg	Rhesus blood group-associated C glycoprotein	protein_coding	56315	-
ENSMUSG00000030553	9.12	0.219	-2.192	0.01125687	0.029820831	Pgpep1l	pyroglutamyl-peptidase I-like	protein_coding	78444	-
ENSMUSG00000030560	68.4	0.133	-2.911	1.30808E-11	1.27054E-09	Ctsc	cathepsin C	protein_coding	13032	-
ENSMUSG00000030577	138.83	0.047	-4.419	1.70256E-15	4.93439E-13	Cd22	CD22 antigen	protein_coding	12483	-

ENSMUSG00000 030609	96.22	4.831	2.272	2.19398E-09	1.01086E-07	Aen	apoptosis enhancing nuclease	protein_coding	68048	-
ENSMUSG00000 030647	18.97	0.123	-3.018	1.03018E-07	2.29669E-06	Ndufc2	NADH:ubiquinone oxidoreductase subunit C2	protein_coding	68197	-
ENSMUSG00000 030650	11.98	4.366	2.126	0.000132281	0.000819924	Tmc5	transmembrane channel-like gene family 5	protein_coding	74424	-
ENSMUSG00000 030651	15.6	0.24	-2.058	0.000592912	0.002754406	Art2b	ADP-ribosyltransferase 2b	protein_coding	11872	-
ENSMUSG00000 030724	128.63	0.009	-6.751	4.672E-41	2.79837E-37	Cd19	CD19 antigen	protein_coding	12478	-
ENSMUSG00000 030727	37.5	0.17	-2.558	2.62236E-10	1.58125E-08	Rabep2	rabaptin, RAB GTPase binding effector protein 2	protein_coding	70314	-
ENSMUSG00000 030729	162.4	4.225	2.079	1.04063E-09	5.31225E-08	Pgm2l1	phosphoglucomutase 2-like 1	protein_coding	70974	-
ENSMUSG00000 030738	66	0.205	-2.285	3.27053E-08	8.93132E-07	Eif3c	eukaryotic translation initiation factor 3, subunit C	protein_coding	56347	-
ENSMUSG00000 030744	1900.88	0.205	-2.288	1.0989E-08	3.71866E-07	Rps3	ribosomal protein S3	protein_coding	27050	-
ENSMUSG00000 030769	8.8	4.713	2.237	0.002504071	0.008885227	Slc5a11	solute carrier family 5 (sodium/glucose cotransporter), member 11	protein_coding	233836	-
ENSMUSG00000 030793	60.95	0.2	-2.321	3.25746E-11	2.74804E-09	Pycard	PYD and CARD domain containing	protein_coding	66824	-
ENSMUSG00000 030823	9.61	0.237	-2.079	0.001953993	0.007270925	9130019O22R ik	RIKEN cDNA 9130019O22 gene	protein_coding	78921	zf-C2H2
ENSMUSG00000 030825	30.4	7.93	2.987	5.22006E-07	8.92476E-06	Hsd17b14	hydroxysteroid (17-beta) dehydrogenase 14	protein_coding	66065	-
ENSMUSG00000 030838	7.51	4.327	2.113	0.007738861	0.022065945	Ush1c	USH1 protein network component harmonin	protein_coding	72088	-
ENSMUSG00000 030870	22.95	0.219	-2.19	5.97048E-05	0.000428791	Ubfd1	ubiquitin family domain containing 1	protein_coding	28018	-
ENSMUSG00000 030871	11.69	0.18	-2.47	0.00027497	0.00147271	Ears2	glutamyl-tRNA synthetase 2, mitochondrial	protein_coding	67417	-
ENSMUSG00000 030872	43.63	0.115	-3.119	1.14693E-12	1.64873E-10	Gga2	golgi associated, gamma adaptin ear containing, ARF binding protein 2	protein_coding	74105	-
ENSMUSG00000 030880	16.09	0.146	-2.773	6.72382E-07	1.09044E-05	Polr3e	polymerase (RNA) III (DNA directed) polypeptide E	protein_coding	26939	-
ENSMUSG00000 030882	48.28	5.757	2.525	8.69509E-06	8.93833E-05	Dnhd1	dynein heavy chain domain 1	protein_coding	77505	-
ENSMUSG00000 030983	36.57	0.122	-3.04	2.13136E-11	1.94408E-09	Bccip	BRCA2 and CDKN1A interacting protein	protein_coding	66165	-
ENSMUSG00000 030994	12.44	6.124	2.614	7.43276E-05	0.000512703	D7Ertd443e	DNA segment, Chr 7, ERATO Doi 443, expressed	protein_coding	71007	-
ENSMUSG00000 031015	91.3	0.238	-2.069	1.30555E-08	4.26534E-07	Swap70	SWA-70 protein	protein_coding	20947	-
ENSMUSG00000 031029	246.81	0.205	-2.285	1.21872E-12	1.68456E-10	Eif3f	eukaryotic translation initiation factor 3, subunit F	protein_coding	66085	-
ENSMUSG00000 031078	34.87	5.786	2.533	3.33432E-08	9.0632E-07	Cttn	cortactin	protein_coding	13043	-

ENSMUSG00000 031099	7.55	7.853	2.973	0.000242025	0.001331582	Smarca1	SWI/SNF related, matrix associated, actin dependent regulator of chromatin, subfamily a, member 1	protein_coding	93761	-
ENSMUSG00000 031132	11.02	0.121	-3.041	7.93269E-05	0.000540753	Cd40lg	CD40 ligand	protein_coding	21947	-
ENSMUSG00000 031155	32.34	0.155	-2.691	2.35678E-10	1.45031E-08	Pim2	proviral integration site 2	protein_coding	18715	-
ENSMUSG00000 031173	6.56	7.101	2.828	0.010034587	0.027181707	Otc	ornithine transcarbamylase	protein_coding	18416	-
ENSMUSG00000 031256	13.48	0.115	-3.114	7.44601E-05	0.000513223	Cstf2	cleavage stimulation f’ctor, 3' pre-RNA subunit 2	protein_coding	108062	-
ENSMUSG00000 031258	11.95	0.133	-2.91	0.000170703	0.001003834	Xkrx	X-linked Kx blood group related, X-linked	protein_coding	331524	-
ENSMUSG00000 031283	8.87	4.818	2.269	0.000280537	0.001494948	Chrdl1	chordin-like 1	protein_coding	83453	-
ENSMUSG00000 031284	20.86	5.426	2.44	4.2766E-06	5.01651E-05	Pak3	p21 (RAC1) activated kinase 3	protein_coding	18481	-
ENSMUSG00000 031285	6.86	4.534	2.181	0.016352867	0.040006081	Dcx	doublecortin	protein_coding	13193	-
ENSMUSG00000 031298	9.47	7.274	2.863	5.74771E-05	0.000416994	Adgrg2	adhesion G protein-coupled receptor G2	protein_coding	237175	-
ENSMUSG00000 031302	5.54	4.271	2.094	0.01725784	0.041844032	Nlgn3	neuroligin 3	protein_coding	245537	-
ENSMUSG00000 031304	117.62	0.134	-2.898	2.28781E-13	3.95286E-11	Il2rg	interleukin 2 receptor, gamma chain	protein_coding	16186	-
ENSMUSG00000 031320	898.91	0.169	-2.562	2.23792E-11	2.02076E-09	Rps4x	ribosomal protein S4, X-linked	protein_coding	20102	-
ENSMUSG00000 031325	20.96	4.197	2.069	0.000386215	0.00194025	4930519F16Rik	RIKEN cDNA 4930519F16 gene	transcribed_unitary_pseudogene	75106	-
ENSMUSG00000 031342	24.55	4.646	2.216	2.02094E-05	0.000179596	Gpm6b	glycoprotein m6b	protein_coding	14758	-
ENSMUSG00000 031355	45.39	4.533	2.18	3.34404E-08	9.0632E-07	Arhgap6	Rho GTPase activating protein 6	protein_coding	11856	-
ENSMUSG00000 031362	10.36	0.126	-2.984	1.73889E-05	0.000158529	Xlr4c	X-linked lymphocyte-regulated 4C	protein_coding	72891	-
ENSMUSG00000 031376	14.92	6.599	2.722	2.86583E-05	0.000237967	Atp2b3	ATPase, Ca++ transporting, plasma membrane 3	protein_coding	320707	-
ENSMUSG00000 031377	24.31	4.538	2.182	5.67252E-06	6.31142E-05	Bmx	BMX non-receptor tyrosine kinase	protein_coding	12169	-
ENSMUSG00000 031379	17.74	4.553	2.187	0.000380598	0.0019189	Pir	pirin	protein_coding	69656	-
ENSMUSG00000 031384	5.31	13.227	3.725	0.000263581	0.001423591	Asb9	ankyrin repeat and SOCS box-containing 9	protein_coding	69299	-
ENSMUSG00000 031403	25.06	0.133	-2.906	1.08666E-08	3.69114E-07	Dkc1	dyskeratosis congenita 1, dyskerin	protein_coding	245474	-
ENSMUSG00000 031425	104.84	5.208	2.381	5.89203E-09	2.26711E-07	Plp1	proteolipid protein (myelin) 1	protein_coding	18823	-
ENSMUSG00000 031428	8.63	0.193	-2.371	0.001321208	0.005299281	Zcchc18	zinc finger, CCHC domain containing 18	protein_coding	66995	-

ENSMUSG00000 031448	5.28	5.553	2.473	0.020336202	0.047655348	Adprhl1	ADP-ribosylhydrolase like 1	protein_coding	234072	-
ENSMUSG00000 031461	10.36	4.155	2.055	0.003287237	0.011071858	Myom2	myomesin 2	protein_coding	17930	-
ENSMUSG00000 031481	11.12	5.761	2.526	0.002505782	0.008887957	Tpte	transmembrane phosphatase with tensin homology	protein_coding	234129	-
ENSMUSG00000 031493	7.18	4.681	2.227	0.007444023	0.021415571	Ggn	gametogenetin	protein_coding	243897	-
ENSMUSG00000 031494	6.65	0.124	-3.017	0.00017217	0.00101069	Cd209a	CD209a antigen	protein_coding	170786	-
ENSMUSG00000 031502	11.58	5.018	2.327	0.000160926	0.000957508	Col4a1	collagen, type IV, alpha 1	protein_coding	12826	-
ENSMUSG00000 031508	93.28	0.195	-2.357	1.20023E-11	1.17212E-09	Ankrd10	ankyrin repeat domain 10	protein_coding	102334	-
ENSMUSG00000 031548	8.1	5.359	2.422	0.000172474	0.001011507	Sfrp1	secreted frizzled-related protein 1	protein_coding	20377	-
ENSMUSG00000 031549	8.41	5.623	2.491	0.000520423	0.002477868	Ido2	indoleamine 2,3-dioxygenase 2	protein_coding	209176	-
ENSMUSG00000 031552	7.04	8.566	3.099	0.000957567	0.004071584	Adam18	a disintegrin and metallopeptidase domain 18	protein_coding	13524	-
ENSMUSG00000 031554	5.7	4.153	2.054	0.017447264	0.042223555	Adam5	a disintegrin and metallopeptidase domain 5	protein_coding	11499	-
ENSMUSG00000 031558	27.92	5.264	2.396	1.64188E-05	0.000150833	Slit2	slit guidance ligand 2	protein_coding	20563	-
ENSMUSG00000 031559	11.95	7.926	2.987	1.0868E-05	0.000107124	4930555F03R ik	-	lincRNA	-	-
ENSMUSG00000 031561	82.54	4.451	2.154	1.08169E-08	3.68124E-07	Tenm3	teneurin transmembrane protein 3	protein_coding	23965	-
ENSMUSG00000 031576	9.56	10.92	3.449	7.26602E-06	7.69829E-05	Kcnu1	potassium channel, subfamily U, member 1	protein_coding	16532	-
ENSMUSG00000 031577	26.52	0.22	-2.187	0.000640867	0.002929467	Tti2	TELO2 interacting protein 2	protein_coding	234138	-
ENSMUSG00000 031617	14.84	0.181	-2.468	7.76141E-06	8.12731E-05	Tmem184c	transmembrane protein 184C	protein_coding	234463	-
ENSMUSG00000 031626	26.42	4.236	2.083	9.96584E-06	9.92111E-05	Sorbs2	sorbin and SH3 domain containing 2	protein_coding	234214	-
ENSMUSG00000 031635	7.34	6.251	2.644	0.000813235	0.003565977	Anxa10	annexin A10	protein_coding	26359	-
ENSMUSG00000 031647	80.18	9.543	3.254	3.78728E-18	1.70134E-15	Mfap3l	microfibrillar-associated protein 3-like	protein_coding	71306	-
ENSMUSG00000 031659	135.05	0.243	-2.04	1.09809E-09	5.55818E-08	Adcy7	adenylate cyclase 7	protein_coding	11513	-
ENSMUSG00000 031661	16.06	6.023	2.591	0.000234988	0.001300029	Nkd1	naked cuticle 1	protein_coding	93960	-
ENSMUSG00000 031669	9.52	0.197	-2.347	0.000603379	0.0027922	Gins3	GINS complex subunit 3 (Psf3 homolog)	protein_coding	78833	-
ENSMUSG00000 031736	4.69	4.413	2.142	0.017292607	0.041905711	Crnde	colorectal neoplasia differentially expressed (non-protein coding)	processed_transc ript	71296	-

ENSMUSG00000 031748	20.77	6.094	2.607	3.07657E-07	5.73628E-06	Gnao1	guanine nucleotide binding protein, alpha O	protein_coding	14681	-
ENSMUSG00000 031750	6.36	4.254	2.089	0.018340608	0.044012072	Il34	interleukin 34	protein_coding	76527	-
ENSMUSG00000 031754	42.63	0.21	-2.252	1.8574E-09	8.92399E-08	Nudt21	nudix (nucleoside diphosphate linked moiety X)-type motif 21	protein_coding	68219	-
ENSMUSG00000 031756	4.25	0.201	-2.313	0.010135462	0.027403569	Cenpn	centromere protein N	protein_coding	72155	-
ENSMUSG00000 031766	8.38	4.221	2.077	0.005185812	0.016021984	Slc12a3	solute carrier family 12, member 3	protein_coding	20497	-
ENSMUSG00000 031785	195.49	5.806	2.538	1.37329E-10	9.214E-09	Adgrg1	adhesion G protein-coupled receptor G1	protein_coding	14766	-
ENSMUSG00000 031824	162.61	4.446	2.152	1.00539E-10	7.19364E-09	6430548M08 Rik	RIKEN cDNA 6430548M08 gene	protein_coding	234797	-
ENSMUSG00000 031825	43.67	11.786	3.559	1.37296E-09	6.79635E-08	Crispld2	cysteine-rich secretory protein LCCL domain containing 2	protein_coding	78892	-
ENSMUSG00000 031838	102.42	0.092	-3.44	1.50207E-15	4.42471E-13	Ifi30	interferon gamma inducible protein 30	processed_transc ript	65972	-
ENSMUSG00000 031840	5.52	0.15	-2.736	0.001034772	0.004341306	Rab3a	RAB3A, member RAS oncogene family	protein_coding	19339	-
ENSMUSG00000 031848	58.94	0.122	-3.033	2.83188E-12	3.40773E-10	Lsm4	LSM4 homolog, U6 small nuclear RNA and mRNA degradation associated	protein_coding	50783	-
ENSMUSG00000 031870	8.53	4.073	2.026	0.010450024	0.02811867	Pgr	progesterone receptor	protein_coding	18667	ESR-like
ENSMUSG00000 031907	8.76	0.12	-3.064	2.48058E-05	0.000211149	Zfp90	zinc finger protein 90	protein_coding	22751	zf-C2H2
ENSMUSG00000 031917	16.32	0.142	-2.815	9.8512E-07	1.49129E-05	Nip7	NIP7, nucleolar pre-rRNA processing protein	protein_coding	66164	-
ENSMUSG00000 031922	23.55	0.13	-2.945	1.05345E-09	5.3473E-08	Cep57	centrosomal protein 57	protein_coding	74360	-
ENSMUSG00000 031924	51.87	0.16	-2.64	4.70154E-09	1.88156E-07	Cyb5b	cytochrome b5 type B	protein_coding	66427	-
ENSMUSG00000 031931	6.64	0.24	-2.058	0.018639087	0.044567632	Ankrd49	ankyrin repeat domain 49	protein_coding	56503	-
ENSMUSG00000 031948	34.87	0.172	-2.543	1.1128E-07	2.43259E-06	Kars	lysyl-tRNA synthetase	protein_coding	85305	-
ENSMUSG00000 031955	8.27	5.769	2.528	0.000648933	0.002959562	Bcar1	breast cancer anti-estrogen resistance 1	protein_coding	12927	-
ENSMUSG00000 031963	21.77	4.977	2.315	8.14413E-07	1.27475E-05	Bmper	BMP-binding endothelial regulator	protein_coding	73230	-
ENSMUSG00000 031965	8.58	5.276	2.399	0.001424844	0.005639061	Tbx20	T-box 20	protein_coding	57246	T-box
ENSMUSG00000 031967	21.93	0.194	-2.363	6.65604E-07	1.08237E-05	Afg3l1	AFG3-like AAA ATPase 1	protein_coding	114896	-
ENSMUSG00000 031997	68.47	4.539	2.183	4.37551E-07	7.72334E-06	Trpc6	transient receptor potential cation channel, subfamily C, member 6	protein_coding	22068	-
ENSMUSG00000 032011	197.73	0.097	-3.365	1.7917E-15	5.11033E-13	Thy1	thymus cell antigen 1, theta	protein_coding	21838	-

ENSMUSG00000032017	68.04	4.175	2.062	1.64873E-10	1.08124E-08	Grik4	glutamate receptor, iono58aladin kainate 4	protein_coding	110637	-
ENSMUSG00000032033	9.18	4.922	2.299	0.001147164	0.004719286	Barx2	BarH-like homeobox 2	protein_coding	12023	Homeobox
ENSMUSG00000032035	310.02	0.163	-2.618	6.87317E-08	1.64016E-06	Ets1	E26 avian leukemia oncog'ne 1, 5' domain	protein_coding	23871	ETS
ENSMUSG00000032036	54.05	4.082	2.029	1.97838E-09	9.40463E-08	Kirrel3	kirre like nephrin family adhesion molecule 3	protein_coding	67703	-
ENSMUSG00000032044	16.32	0.242	-2.045	0.000121014	0.000760582	Rpusd4	RNA pseudouridylate synthase domain containing 4	protein_coding	71989	-
ENSMUSG00000032053	86.54	0.047	-4.4	1.30595E-21	8.69132E-19	Pou2af1	POU domain, class 2, associating factor 1	protein_coding	18985	-
ENSMUSG00000032076	30.9	4.02	2.007	0.000545124	0.002572302	Cadm1	cell adhesion molecule 1	protein_coding	54725	-
ENSMUSG00000032094	133.74	0.218	-2.195	8.76772E-09	3.08473E-07	Cd3d	CD3 antigen, delta polypeptide	protein_coding	12500	-
ENSMUSG00000032103	17.45	0.187	-2.419	1.56872E-06	2.19364E-05	Pus3	pseudouridine synthase 3	protein_coding	67049	-
ENSMUSG00000032105	6.92	5.165	2.369	0.000299937	0.00158098	Pdzd3	PDZ domain containing 3	protein_coding	170761	-
ENSMUSG00000032109	92.34	4.186	2.065	1.44281E-08	4.61315E-07	Nlrx1	NLR family member X1	protein_coding	270151	-
ENSMUSG00000032178	36.41	0.243	-2.043	1.16084E-07	2.52226E-06	Ilf3	interleukin enhancer binding factor 3	protein_coding	16201	-
ENSMUSG00000032192	10.47	7.879	2.978	2.84242E-05	0.000236242	Gnb5	guanine nucleotide binding protein (G protein), beta 5	protein_coding	14697	-
ENSMUSG00000032215	19.1	0.18	-2.47	5.78266E-06	6.39831E-05	Rsl24d1	ribosomal L24 domain containing 1	protein_coding	225215	-
ENSMUSG00000032261	791.7	6.973	2.802	1.15716E-11	1.14879E-09	Sh3bgrl2	SH3 domain binding glutamic acid-rich protein like 2	protein_coding	212531	-
ENSMUSG00000032262	5.59	6.127	2.615	0.005995615	0.018034015	Elovl4	elongation of very long chain fatty acids (FEN1/Elo2, SUR4/Elo3, yeast)-like 4	protein_coding	83603	-
ENSMUSG00000032288	33.3	0.182	-2.462	1.65611E-09	8.04287E-08	Imp3	IMP3, U3 small nucleolar ribonucleoprotein	protein_coding	102462	-
ENSMUSG00000032298	7.52	0.072	-3.791	7.70078E-06	8.08652E-05	Neil1	nei endonuclease VIII-like 1 (E. coli)	protein_coding	72774	-
ENSMUSG00000032299	26.67	0.076	-3.712	1.82392E-10	1.17425E-08	Commd4	COMM domain containing 4	protein_coding	66199	-
ENSMUSG00000032301	44.43	0.248	-2.012	1.15749E-06	1.70484E-05	Psma4	proteasome (prosome, macropain) subunit, alpha type 4	protein_coding	26441	-
ENSMUSG00000032309	32.33	0.206	-2.278	9.31905E-08	2.10554E-06	Fbxo22	F-box protein 22	protein_coding	71999	-
ENSMUSG00000032313	10.21	5.108	2.353	0.001641409	0.00632115	Tmem266	transmembrane protein 266	protein_coding	244886	-
ENSMUSG00000032323	9.84	0.13	-2.941	3.51459E-05	0.000281559	Cyp11a1	cytochrome P450, family 11, subfamily a, polypeptide 1	protein_coding	13070	-
ENSMUSG00000032333	26.84	0.167	-2.585	8.28142E-06	8.59669E-05	Stoml1	stomatin-like 1	protein_coding	69106	-

ENSMUSG00000032338	10.48	4.059	2.021	0.00584167	0.017665595	Hcn4	hyperpolarization-activated, cyclic nucleotide-gated K+ 4	protein_coding	330953	-
ENSMUSG00000032360	6.98	7.013	2.81	0.00090087	0.00387359	Hcrtr2	hypocretin (orexin) receptor 2	protein_coding	387285	-
ENSMUSG00000032383	157.39	0.205	-2.289	2.8829E-11	2.49052E-09	Ppib	peptidylprolyl isomerase B	protein_coding	19035	-
ENSMUSG00000032394	7.61	4.145	2.051	0.00350145	0.01164924	Igdcc3	immunoglobulin superfamily, DCC subclass, member 3	protein_coding	19289	-
ENSMUSG00000032397	9.15	0.185	-2.436	0.000611509	0.002824004	Tipin	timeless interacting protein	protein_coding	66131	-
ENSMUSG00000032403	9.43	0.166	-2.59	0.000186342	0.001076996	2300009A05Rik	RIKEN cDNA 2300009A05 gene	protein_coding	69478	-
ENSMUSG00000032437	73.52	0.208	-2.268	1.45937E-08	4.64131E-07	Stt3b	STT3, subunit of the oligosaccharyltransferase complex, homolog B (S. cerevisiae)	protein_coding	68292	-
ENSMUSG00000032443	13.58	4.287	2.1	0.000467214	0.002270248	Zcwpw2	-	transcribed_unprocessed_pseudogene	-	-
ENSMUSG00000032459	9.49	0.249	-2.007	0.007745737	0.022071543	Mrps22	mitochondrial ribosomal protein S22	protein_coding	64655	-
ENSMUSG00000032492	7.88	4.544	2.184	0.012960266	0.033250002	Pth1r	parathyroid hormone 1 receptor	protein_coding	19228	-
ENSMUSG00000032502	20.28	4.698	2.232	0.000317589	0.001655092	Stac	src homology three (SH3) and cysteine rich domain	protein_coding	20840	-
ENSMUSG00000032503	27.26	5.161	2.368	1.18452E-05	0.000115114	Arpp21	cyclic AMP-regulated phosphoprotein, 21	protein_coding	74100	-
ENSMUSG00000032511	20.19	6.629	2.729	0.00025013	0.001367382	Scn5a	sodium channel, voltage-gated, type V, alpha	protein_coding	20271	-
ENSMUSG00000032518	2339.78	0.158	-2.659	2.84522E-11	2.46985E-09	Rpsa	ribosomal protein SA	protein_coding	16785	-
ENSMUSG00000032528	39.49	0.184	-2.442	6.04181E-08	1.47507E-06	Vipr1	vasoactive intestinal peptide receptor 1	protein_coding	22354	-
ENSMUSG00000032531	15.52	5.609	2.488	0.000183016	0.001062898	Amotl2	angiomotin-like 2	protein_coding	56332	-
ENSMUSG00000032595	12.5	4.543	2.184	0.005394301	0.016521254	Cdhr4	cadherin-related family member 4	protein_coding	69398	-
ENSMUSG00000032657	57.42	0.135	-2.886	2.39313E-14	5.5847E-12	Fam189b	family with sequence similarity 189, member B	protein_coding	68521	-
ENSMUSG00000032714	5.07	4.915	2.297	0.011312799	0.029938099	Syde1	synapse defective 1, Rho GTPase, homolog 1 (C. elegans)	protein_coding	71709	-
ENSMUSG00000032726	12.06	10.213	3.352	3.70222E-05	0.000292161	Bmp8a	bone morphogenetic protein 8a	protein_coding	12163	-
ENSMUSG00000032739	79.37	4.677	2.226	8.38693E-07	1.3082E-05	Pram1	PML-RAR alpha-regulated adaptor molecule 1	protein_coding	378460	-
ENSMUSG00000032763	18.31	0.25	-2	0.000554942	0.002605134	Ilvbl	ilvB (bacterial acetolactate synthase)-like	protein_coding	216136	-
ENSMUSG00000032766	5366.19	4.504	2.171	1.37222E-05	0.000129973	Gng11	guanine nucleotide binding protein (G protein), gamma 11	protein_coding	66066	-

ENSMUSG00000 032826	61.35	4.167	2.059	4.21363E-08	1.09658E-06	Ank2	ankyrin 2, brain	protein_coding	109676	-
ENSMUSG00000 032834	12.91	0.239	-2.064	0.003802942	0.012456266	Pwp2	PWP2 periodic tryptophan protein homolog (yeast)	protein_coding	110816	-
ENSMUSG00000 032839	9.74	4.471	2.161	0.003418784	0.011413481	Trpc1	transient receptor potential cation channel, subfamily C, member 1	protein_coding	22063	-
ENSMUSG00000 032852	5.51	11.236	3.49	0.000100047	0.000654203	Rspo4	R-spondin 4	protein_coding	228770	-
ENSMUSG00000 032878	8.83	6.262	2.647	0.000486533	0.002343843	Ccdc85a	coiled-coil domain containing 85A	protein_coding	216613	-
ENSMUSG00000 032936	9.32	4.18	2.064	0.001293933	0.005207318	Camkv	CaM kinase-like vesicle-associated	protein_coding	235604	-
ENSMUSG00000 032959	55.51	0.158	-2.658	6.07911E-12	6.58045E-10	Pebp1	phosphatidylethanolamine binding protein 1	protein_coding	23980	-
ENSMUSG00000 033047	57.01	0.247	-2.016	1.26212E-05	0.000121213	Eif3l	eukaryotic translation initiation factor 3, subunit L	protein_coding	223691	-
ENSMUSG00000 033082	5.76	6.262	2.647	0.001404292	0.005561824	Clec1a	C-type lectin domain family 1, member a	protein_coding	243653	-
ENSMUSG00000 033105	5.15	0.168	-2.57	0.00629999	0.018754891	Lss	lanosterol synthase	protein_coding	16987	-
ENSMUSG00000 033147	43.5	4.063	2.023	8.24346E-09	2.92741E-07	Slc22a15	solute carrier family 22 (organic anion/cation transporter), member 15	protein_coding	242126	-
ENSMUSG00000 033170	44.28	4.822	2.27	1.60345E-08	5.01831E-07	Card10	caspase recruitment domain family, member 10	protein_coding	105844	-
ENSMUSG00000 033177	10.33	4.658	2.22	0.012237052	0.03179864	Tmprss7	transmembrane serine protease 7	protein_coding	208171	-
ENSMUSG00000 033182	10.71	4.536	2.181	0.000947031	0.004031557	Kbtbd12	kelch repeat and BTB (POZ) domain containing 12	protein_coding	74589	-
ENSMUSG00000 033196	9.16	4.41	2.141	0.002116676	0.007773256	Myh2	myosin, heavy polypeptide 2, skeletal muscle, adult	protein_coding	17882	-
ENSMUSG00000 033213	9.44	4.967	2.312	0.000307066	0.001614161	AA467197	expressed sequence AA467197	protein_coding	433470	-
ENSMUSG00000 033318	11.16	0.194	-2.368	0.000119616	0.000752581	Gstt2	glutathione S-transferase, theta 2	protein_coding	14872	-
ENSMUSG00000 033327	11.34	4.03	2.011	0.000531433	0.002518278	Tnxb	tenascin XB	protein_coding	81877	-
ENSMUSG00000 033389	22.42	6.17	2.625	0.000109524	0.000702622	Arhgap44	Rho GTPase activating protein 44	protein_coding	216831	-
ENSMUSG00000 033419	14.77	4.336	2.116	0.001425358	0.005639061	Snap91	synaptosomal-associated protein 91	protein_coding	20616	-
ENSMUSG00000 033450	93.77	0.144	-2.793	8.70086E-11	6.43504E-09	Tagap	T cell activation Rho GTPase activating protein	protein_coding	72536	-
ENSMUSG00000 033460	9.06	5.562	2.476	0.007884847	0.022411074	Armcx1	armadillo repeat containing, X-linked 1	protein_coding	78248	-
ENSMUSG00000 033470	45.05	4.685	2.228	2.03922E-07	4.0669E-06	Cysltr2	cysteinyl leukotriene receptor 2	protein_coding	70086	-
ENSMUSG00000 033597	7.71	7.046	2.817	0.000137069	0.000844358	Caskin1	CASK interacting protein 1	protein_coding	268932	-

ENSMUSG00000033676	34.16	4.008	2.003	1.84205E-06	2.51137E-05	Gabrb3	gamma-aminobutyric acid (GABA) A receptor, subunit beta 3	protein_coding	14402	-
ENSMUSG00000033706	8.6	0.122	-3.04	0.000199865	0.001142097	Smyd5	SET and MYND domain containing 5	protein_coding	232187	-
ENSMUSG00000033717	5.47	5.141	2.362	0.001304241	0.005242932	Adra2a	adrenergic receptor, alpha 2a	protein_coding	11551	-
ENSMUSG00000033781	29.08	0.2	-2.323	5.16197E-06	5.84839E-05	Asb13	ankyrin repeat and SOCS box-containing 13	protein_coding	142688	-
ENSMUSG00000033809	7.91	0.008	-6.954	2.56617E-07	4.92119E-06	Alg3	asparagine-linked glycosylation 3 (alpha-1,3-mannosyltransferase)	protein_coding	208624	-
ENSMUSG00000033813	79.63	0.211	-2.242	7.83208E-09	2.82033E-07	Tcea1	transcription elongation factor A (SII) 1	protein_coding	21399	-
ENSMUSG00000033845	12.75	0.041	-4.615	6.27471E-09	2.36583E-07	Mrpl15	mitochondrial ribosomal protein L15	protein_coding	27395	-
ENSMUSG00000033952	6.44	8.52	3.091	0.000848404	0.003689489	Aspm	abnormal spindle microtubule assembly	protein_coding	12316	-
ENSMUSG00000033960	12.15	15.256	3.931	1.22771E-09	6.14504E-08	Jcad	junctional cadherin 5 associated	protein_coding	240185	-
ENSMUSG00000033983	8.67	0.223	-2.165	0.000959445	0.004078604	Coil	coilin	protein_coding	12812	-
ENSMUSG00000033998	8.88	4.777	2.256	0.00217005	0.007933599	Kcnk1	potassium channel, subfamily K, member 1	protein_coding	16525	-
ENSMUSG00000034024	90.64	0.164	-2.612	5.47673E-14	1.15778E-11	Cct2	chaperonin containing Tcp1, subunit 2 (beta)	protein_coding	12461	-
ENSMUSG00000034028	389.84	4.552	2.187	4.54216E-09	1.83E-07	Cd226	CD226 antigen	protein_coding	225825	-
ENSMUSG00000034032	16.21	0.242	-2.047	0.000123814	0.000774924	Rpap1	RNA polymerase II associated protein 1	protein_coding	68925	-
ENSMUSG00000034059	19.45	4.306	2.107	0.000235845	0.00130437	Ypel4	yippee like 4	protein_coding	241525	-
ENSMUSG00000034063	10	4.354	2.122	0.001522545	0.00594623	4930590J08Rik	RIKEN cDNA 4930590J08 gene	protein_coding	381798	-
ENSMUSG00000034171	40.08	0.242	-2.047	4.84813E-06	5.54526E-05	Faah	fatty acid amide hydrolase	protein_coding	14073	-
ENSMUSG00000034201	527.66	5.012	2.325	8.77875E-08	1.99931E-06	Gas2l1	growth arrest-specific 2 like 1	protein_coding	78926	-
ENSMUSG00000034205	12.31	4.488	2.166	5.36385E-05	0.000394204	Loxl2	lysyl oxidase-like 2	protein_coding	94352	-
ENSMUSG00000034209	22.08	5.669	2.503	9.39514E-06	9.47901E-05	Rasl10a	RAS-like, family 10, member A	protein_coding	75668	-
ENSMUSG00000034239	6.09	4.909	2.296	0.001375157	0.0054717	Gm884	predicted gene 884	protein_coding	380730	-
ENSMUSG00000034263	16.48	0.175	-2.515	4.99256E-05	0.000372092	Ints14	integrator complex subunit 14	protein_coding	69882	-
ENSMUSG00000034275	8.87	5.68	2.506	2.93337E-05	0.000242232	Igsf9b	immunoglobulin superfamily, member 9B	protein_coding	235086	-
ENSMUSG00000034285	15.6	0.184	-2.441	5.7557E-05	0.00041737	Nipsnap1	nipsnap homolog 1	protein_coding	18082	-

ENSMUSG00000 034371	12.45	0.122	-3.037	4.56729E-06	5.2868E-05	Tkfc	triokinase, FMN cyclase	protein_coding	225913	-
ENSMUSG00000 034379	6.3	0.086	-3.545	4.73613E-05	0.000358255	Wdr5b	WD repeat domain 5B	protein_coding	69544	-
ENSMUSG00000 034402	24.52	4.773	2.255	4.54039E-06	5.26363E-05	Kcnh5	potassium voltage-gated channel, subfamily H (eag-related), member 5	protein_coding	238271	-
ENSMUSG00000 034429	10.79	0.05	-4.332	3.32749E-07	6.11367E-06	Zfp707	zinc finger protein 707	protein_coding	69020	zf-C2H2
ENSMUSG00000 034460	5.24	7.865	2.976	0.002982906	0.010220426	Six4	sine oculis-related homeobox 4	protein_coding	20474	Homeobox
ENSMUSG00000 034484	126.9	0.166	-2.591	5.70384E-12	6.26427E-10	Snx2	sorting nexin 2	protein_coding	67804	-
ENSMUSG00000 034486	7.52	7.294	2.867	6.63797E-05	0.000467939	Gbx2	gastrulation brain homeobox 2	protein_coding	14472	Homeobox
ENSMUSG00000 034533	17.95	5.172	2.371	0.000211599	0.001195291	Scn10a	sodium channel, voltage-gated, type X, alpha	protein_coding	20264	-
ENSMUSG00000 034634	209.17	0.022	-5.501	1.40075E-36	5.03403E-33	Ly6d	lymphocyte antigen 6 complex, locus D	protein_coding	17068	-
ENSMUSG00000 034645	6.28	6.945	2.796	0.000123256	0.000772241	Zyg11a	zyg-11 family member A, cell cycle regulator	protein_coding	230590	-
ENSMUSG00000 034659	24.2	0.223	-2.162	1.62659E-05	0.000149811	Tmem109	transmembrane protein 109	protein_coding	68539	-
ENSMUSG00000 034664	1392.1	4.634	2.212	1.27555E-05	0.000122242	Itga2b	integrin alpha 2b	protein_coding	16399	-
ENSMUSG00000 034684	13.3	6.425	2.684	1.23968E-05	0.000119186	Sema3f	sema domain, immunoglobulin domain (Ig), short basic domain, sec62aladin62izamaphorin) 3F	protein_coding	20350	-
ENSMUSG00000 034687	38.98	4.177	2.063	2.19583E-05	0.000191539	Fras1	Fraser extracellular matrix complex subunit 1	protein_coding	231470	-
ENSMUSG00000 034738	6.97	4.277	2.097	0.010709872	0.028701817	Nostrin	nitric oxide synthase trafficker	protein_coding	329416	-
ENSMUSG00000 034818	7.09	4.532	2.18	0.009313012	0.025595826	Celf5	CUGBP, Elav-like family member 5	protein_coding	319586	-
ENSMUSG00000 034826	14.96	0.213	-2.232	0.000484829	0.00233939	Nup54	nucleoporin 54	protein_coding	269113	-
ENSMUSG00000 034833	28.81	0.164	-2.612	1.22192E-07	2.6327E-06	Tespa1	thymocyte expressed, positive selection associated 1	protein_coding	67596	-
ENSMUSG00000 034842	15.69	6.691	2.742	1.31226E-06	1.8879E-05	Art3	ADP-ribosyltransferase 3	protein_coding	109979	-
ENSMUSG00000 034855	8.46	11.677	3.546	2.48357E-06	3.19909E-05	Cxcl10	chemokine (C-X-C motif) ligand 10	protein_coding	15945	-
ENSMUSG00000 034919	7.2	9.034	3.175	4.95482E-05	0.000370197	Ttc22	tetratricopeptide repeat domain 22	protein_coding	230576	-
ENSMUSG00000 034959	32.44	0.221	-2.177	1.21448E-06	1.77566E-05	Rubcnl	RUN and cysteine rich domain containing beclin 1 interacting protein like	protein_coding	271221	-
ENSMUSG00000 035032	19.08	4.071	2.025	0.000403678	0.002015476	Nek11	NIMA (never in mitosis gene a)-related expressed kinase 11	protein_coding	208583	-

ENSMUSG00000035067	56.02	5.005	2.323	1.33947E-09	6.64887E-08	Xkr6	X-linked Kx blood group related 6	protein_coding	219149	-
ENSMUSG00000035177	4.29	4.85	2.278	0.019851919	0.046764439	Nlrp2	NLR family, pyrin domain containing 2	protein_coding	232827	-
ENSMUSG00000035184	7.04	4.744	2.246	0.005128197	0.015863068	Fam124a	family with sequence similarity 124, member A	protein_coding	629059	-
ENSMUSG00000035227	75.03	0.157	-2.674	5.87248E-11	4.60797E-09	Spcs2	signal peptidase complex subunit 2 homolog (S. cerevisiae)	protein_coding	66624	-
ENSMUSG00000035246	27.32	4.178	2.063	4.06879E-06	4.81317E-05	Pcyt1b	phosphate cytidylyltransferase 1, choline, beta isoform	protein_coding	236899	-
ENSMUSG00000035273	152.29	4.19	2.067	1.57391E-07	3.26576E-06	Hpse	heparanase	protein_coding	15442	-
ENSMUSG00000035274	8.37	5.37	2.425	0.000261146	0.001413412	Tpbg	trophoblast glycoprotein	protein_coding	21983	-
ENSMUSG00000035283	9.97	6.853	2.777	0.002632016	0.009237246	Adrb1	adrenergic receptor, beta 1	protein_coding	11554	-
ENSMUSG00000035342	8.08	5.277	2.4	0.001771257	0.006716124	Lzts2	leucine zipper, putative tumor suppressor 2	protein_coding	226154	-
ENSMUSG00000035357	37.02	4.582	2.196	3.34011E-08	9.0632E-07	Pdzrn3	PDZ domain containing RING finger 3	protein_coding	55983	-
ENSMUSG00000035370	43.98	0.19	-2.397	1.28682E-08	4.23497E-07	Adat3	-	protein_coding	-	-
ENSMUSG00000035390	8.41	6.115	2.612	0.000499314	0.002389395	Brsk1	BR serine/threonine kinase 1	protein_coding	381979	-
ENSMUSG00000035403	5.48	5.2	2.379	0.003820533	0.012504766	Crb2	crumbs family member 2	protein_coding	241324	-
ENSMUSG00000035407	8.36	12.671	3.663	8.92107E-05	0.000595257	Kank4	KN motif and ankyrin repeat domains 4	protein_coding	242553	-
ENSMUSG00000035429	11.53	5.977	2.58	0.004471928	0.01419217	Ptprh	protein tyrosine phosphatase, receptor type, H	protein_coding	545902	-
ENSMUSG00000035456	9.3	10.132	3.341	4.7473E-06	5.45773E-05	Prdm8	PR domain containing 8	protein_coding	77630	zf-C2H2
ENSMUSG00000035459	20	5.696	2.51	5.59921E-06	6.25698E-05	Stab2	stabilin 2	protein_coding	192188	-
ENSMUSG00000035478	58.91	0.25	-2.001	3.01214E-08	8.32694E-07	Mbd3	methyl-CpG binding domain protein 3	protein_coding	17192	MBD
ENSMUSG00000035486	6.95	11.869	3.569	8.47555E-05	0.000570188	Plk5	polo like kinase 5	protein_coding	216166	-
ENSMUSG00000035498	8.11	7.055	2.819	0.000726058	0.00324218	Cdcp1	CUB domain containing protein 1	protein_coding	109332	-
ENSMUSG00000035606	8.33	8.5	3.087	0.00043326	0.00213939	Ky	kyphoscoliosis peptidase	protein_coding	16716	-
ENSMUSG00000035615	21.11	4.433	2.148	0.000244169	0.001341324	Frmpd1	FERM and PDZ domain containing 1	protein_coding	666060	-
ENSMUSG00000035683	7.05	4.173	2.061	0.00435378	0.013910574	Melk	maternal embryonic leucine zipper kinase	protein_coding	17279	-
ENSMUSG00000035772	8.59	0.062	-4.005	1.7723E-06	2.43661E-05	Mrps2	mitochondrial ribosomal protein S2	protein_coding	118451	-

ENSMUSG00000035829	5.52	8.678	3.117	0.008293074	0.023331493	Ppp1r26	protein phosphatase 1, regulatory subunit 26	protein_coding	241289	-
ENSMUSG00000035873	14.31	4.586	2.197	0.000410004	0.002040819	Pawr	PRKC, apoptosis, WT1, regulator	protein_coding	114774	-
ENSMUSG00000035934	28.57	5.361	2.423	2.93549E-05	0.000242296	Pknox2	Pbx/knotted 1 homeobox 2	protein_coding	208076	Homeobox
ENSMUSG00000035958	20.61	0.221	-2.181	0.000370057	0.001873127	Tdp2	tyrosyl-DNA phosphodiesterase 2	protein_coding	56196	-
ENSMUSG00000036030	12.65	4.293	2.102	8.11092E-05	0.000550397	Prtg	protogenin	protein_coding	235472	-
ENSMUSG00000036052	8.77	0.221	-2.177	0.000929306	0.003968322	Dnajb5	DnaJ heat shock protein family (Hsp40) member B5	protein_coding	56323	-
ENSMUSG00000036061	11.18	0.249	-2.005	0.00633188	0.018824411	Smug1	single-strand selective monofunctional uracil DNA glycosylase	protein_coding	71726	-
ENSMUSG00000036078	21.68	0.189	-2.4	1.50912E-06	2.12685E-05	Sigmar1	sigma non-opioid intracellular receptor 1	protein_coding	18391	-
ENSMUSG00000036098	6	4.232	2.081	0.005373715	0.016472243	Myrf	myelin regulatory factor	protein_coding	225908	NDT80/PhoG
ENSMUSG00000036158	19.1	4.304	2.106	5.90402E-05	0.000425208	Prickle1	prickle planar cell polarity protein 1	protein_coding	106042	-
ENSMUSG00000036160	27.55	0.163	-2.619	4.08891E-09	1.67366E-07	Surf6	surfeit gene 6	protein_coding	20935	-
ENSMUSG00000036192	19.51	4.705	2.234	0.000214229	0.001206355	Rorb	RAR-related orphan receptor beta	protein_coding	225998	THR-like
ENSMUSG00000036198	6.37	5.586	2.482	0.007360437	0.021222671	Arhgap36	Rho GTPase activating protein 36	protein_coding	75404	-
ENSMUSG00000036218	36.05	4.155	2.055	4.53065E-05	0.000345402	Pdzrn4	PDZ domain containing RING finger 4	protein_coding	239618	-
ENSMUSG00000036251	10.85	9.2	3.202	2.42766E-06	3.14058E-05	Trpm8	transient receptor potential cation channel, subfamily M, member 8	protein_coding	171382	-
ENSMUSG00000036264	45.89	4.144	2.051	1.60991E-07	3.31369E-06	Fstl4	follistatin-like 4	protein_coding	320027	-
ENSMUSG00000036295	259.16	4.826	2.271	2.05495E-07	4.09373E-06	Lrrn3	leucine rich repeat protein 3, neuronal	protein_coding	16981	-
ENSMUSG00000036298	30.53	4.527	2.179	2.20268E-08	6.48258E-07	Slc2a13	solute carrier family 2 (facilitated glucose transporter), member 13	protein_coding	239606	-
ENSMUSG00000036353	898.48	6.671	2.738	1.6384E-11	1.57435E-09	P2ry12	purinergic receptor P2Y, G-protein coupled 12	protein_coding	70839	-
ENSMUSG00000036356	90.57	4.705	2.234	6.31645E-09	2.37448E-07	Csgalnact1	chondroitin sulfate N-acetylgalactosaminyltransferase 1	protein_coding	234356	-
ENSMUSG00000036377	101.67	4.064	2.023	2.28803E-08	6.7179E-07	C530008M17Rik	RIKEN cDNA C530008M17 gene	protein_coding	320827	-
ENSMUSG00000036459	6.41	7.043	2.816	0.002484111	0.008826772	Wtip	WT1-interacting protein	protein_coding	101543	-
ENSMUSG00000036504	8.05	0.128	-2.97	0.000506473	0.002420428	Phpt1	phosphohistidine phosphatase 1	protein_coding	75454	-
ENSMUSG00000036510	32.98	4.104	2.037	1.52329E-05	0.000141824	Cdh8	cadherin 8	protein_coding	12564	-

ENSMUSG00000036523	14.36	6.777	2.761	8.8809E-06	9.08257E-05	Greb1	gene regulated by estrogen in breast cancer protein	protein_coding	268527	-
ENSMUSG00000036545	28.72	8.73	3.126	1.77638E-14	4.19998E-12	Adamts2	a disintegrin-like and metallopeptidase (reprolysin type) with thrombospondin type 1 motif, 2	protein_coding	216725	-
ENSMUSG00000036564	8.7	7.836	2.97	0.000166077	0.000981659	Ndrg4	N-myc downstream regulated gene 4	protein_coding	234593	-
ENSMUSG00000036594	688.79	0.02	-5.66	1.08897E-23	9.31795E-21	H2-Aa	histocompatibility 2, class II antigen A, alpha	protein_coding	14960	-
ENSMUSG00000036596	6.67	5.707	2.513	0.005234728	0.016145354	Cpz	carboxypeptidase Z	protein_coding	242939	-
ENSMUSG00000036693	21.81	0.228	-2.132	1.28491E-05	0.000123007	Nop14	NOP14 nucleolar protein	protein_coding	75416	-
ENSMUSG00000036718	8.65	5.687	2.508	0.000257413	0.00139784	Micall2	MICAL-like 2	protein_coding	231830	-
ENSMUSG00000036760	6.24	4.778	2.256	0.001952944	0.007268529	Kcnk9	potassium channel, subfamily K, member 9	protein_coding	223604	-
ENSMUSG00000036775	5.22	0.223	-2.166	0.010723814	0.028734895	Decr2	2-4-dienoyl-Coenzyme A reductase 2, peroxisomal	protein_coding	26378	-
ENSMUSG00000036800	27.58	4.75	2.248	0.000221642	0.001240325	Fam135b	family with sequence similarity 135, member B	protein_coding	70363	-
ENSMUSG00000036814	6.87	4.998	2.321	0.00155597	0.006055714	Slc6a20a	solute carrier family 6 (neurotransmitter transporter), member 20A	protein_coding	102680	-
ENSMUSG00000036819	10.51	0.146	-2.775	5.66171E-05	0.000412051	Jmjd4	jumonji domain containing 4	protein_coding	194952	-
ENSMUSG00000036825	28.47	4.416	2.143	3.5062E-05	0.000281263	Ssx2ip	synovial sarcoma, X 2 interacting protein	protein_coding	99167	-
ENSMUSG00000036867	17.02	4.075	2.027	0.000192616	0.001107555	Smad6	SMAD family member 6	protein_coding	17130	MH1
ENSMUSG00000036962	11.36	6.212	2.635	0.000961435	0.004086101	Cfap221	cilia and flagella associated protein 221	protein_coding	226356	-
ENSMUSG00000036980	20.49	0.158	-2.665	7.35518E-06	7.76533E-05	Taf6	TATA-box binding protein associated factor 6	protein_coding	21343	-
ENSMUSG00000036986	108.91	0.123	-3.026	2.85635E-13	4.84205E-11	Pml	promyelocytic leukemia	protein_coding	18854	-
ENSMUSG00000037029	25.1	0.231	-2.111	8.99998E-07	1.39055E-05	Zfp146	zinc finger protein 146	protein_coding	26465	zf-C2H2
ENSMUSG00000037032	11.73	6.622	2.727	2.95445E-05	0.000243302	Apbb1	amyloid beta (A4) precursor protein-binding, family B, member 1	protein_coding	11785	-
ENSMUSG00000037035	10.2	7.825	2.968	1.29592E-05	0.000123864	Inhbb	inhibin beta-B	protein_coding	16324	-
ENSMUSG00000037071	86.41	0.035	-4.841	2.81957E-20	1.5353E-17	Scd1	stearoyl-Coenzyme A desaturase 1	protein_coding	20249	-
ENSMUSG00000037149	14.63	0.158	-2.662	0.000199821	0.001142097	Ddx1	DEAD (Asp-Glu-Ala-Asp) box polypeptide 1	protein_coding	104721	-
ENSMUSG00000037211	8.72	4.37	2.127	0.00531439	0.016318265a ladi	sprouty RTK signaling antagonist 1	protein_coding	24063	-	

ENSMUSG00000 037236	93.05	0.211	-2.244	4.86841E-11	3.94056E-09	Matr3	matrin 3	protein_coding	17184	-
ENSMUSG00000 037242	678.78	4.808	2.265	2.76899E-08	7.8356E-07	Clic4	chloride intracellular channel 4 (mitochondrial)	protein_coding	29876	-
ENSMUSG00000 037262	12.13	0.171	-2.548	0.000135323	0.000835609	Kin	Kin17 DNA and RNA binding protein	protein_coding	16588	-
ENSMUSG00000 037278	6.36	4.187	2.066	0.013702814	0.034709031	Tmem97	transmembrane protein 97	protein_coding	69071	-
ENSMUSG00000 037279	7.74	25.053	4.647	4.38729E-08	1.12587E-06	Ovol2	ovo like zinc finger 2	protein_coding	107586	zf-C2H2
ENSMUSG00000 037316	10.82	0.096	-3.375	1.53381E-06	2.15394E-05	Bag4	BCL2-associated athanogene 4	protein_coding	67384	-
ENSMUSG00000 037336	129.37	4.072	2.026	2.53986E-07	4.88116E-06	Mfsd2b	major facilitator superfamily domain containing 2B	protein_coding	432628	-
ENSMUSG00000 037337	63.34	0.242	-2.048	6.12321E-08	1.4882E-06	Map4k1	mitogen-activated protein kinase kinase kinase kinase 1	protein_coding	26411	-
ENSMUSG00000 037361	57.84	0.199	-2.328	2.02381E-09	9.49499E-08	Sf3b6	splicing factor 3B, subunit 6	protein_coding	66055	-
ENSMUSG00000 037386	41.16	6.505	2.702	4.6105E-09	1.85338E-07	Rims2	regulating synaptic membrane exocytosis 2	protein_coding	116838	-
ENSMUSG00000 037406	4.75	5.638	2.495	0.015069686	0.037458458	Htra4	HtrA serine peptidase 4	protein_coding	330723	-
ENSMUSG00000 037455	26.38	6.217	2.636	0.000684956	0.003091677	Slc18b1	solute carrier family 18, subfamily B, member 1	protein_coding	76306	-
ENSMUSG00000 037461	20.77	0.17	-2.557	7.66019E-05	0.000525567	Ints7	integrator complex subunit 7	protein_coding	77065	-
ENSMUSG00000 037531	15.26	0.136	-2.883	5.27748E-06	5.96046E-05	Mrpl47	mitochondrial ribosomal protein L47	protein_coding	74600	-
ENSMUSG00000 037541	64.84	5.51	2.462	3.55796E-06	4.32272E-05	Shank2	SH3 and multiple ankyrin repeat domains 2	protein_coding	210274	-
ENSMUSG00000 037544	9	4.055	2.02	0.010541166	0.028329975	Dlgap5	DLG associated protein 5	protein_coding	218977	-
ENSMUSG00000 037548	212.94	0.024	-5.364	2.81982E-24	2.53347E-21	H2-DMb2	histocompatibility 2, class II, locus Mb2	protein_coding	15000	-
ENSMUSG00000 037594	6.37	4.816	2.268	0.009615246	0.026277773	BC022687	clathrin binding box of aftiphilin containing 1	protein_coding	217887	-
ENSMUSG00000 037649	89.37	0.092	-3.441	3.41712E-20	1.80595E-17	H2-DMa	histocompatibility 2, class II, locus DMa	protein_coding	14998	-
ENSMUSG00000 037664	8.62	6.541	2.709	0.000254377	0.001386801	Cdkn1c	cyclin-dependent kinase inhibitor 1C (P57)	protein_coding	12577	-
ENSMUSG00000 037686	4.48	7.66	2.937	0.000707754	0.003179408	Aspg	asparaginase	protein_coding	104816	-
ENSMUSG00000 037705	14.84	5.753	2.524	0.000710382	0.003188823	Tecta	tectorin alpha	protein_coding	21683	-
ENSMUSG00000 037708	5.64	5.109	2.353	0.005682279	0.017267859	Spag6	sperm associated antigen 6	protein_coding	381350	-
ENSMUSG00000 037712	7.02	7.244	2.857	0.000187444	0.001082323	Fermt2	fermitin family member 2	protein_coding	218952	-

ENSMUSG00000037722	11.74	0.21	-2.254	0.000166542	0.00098408	Gnpnat1	glucosamine-phosphate N-acetyltransferase 1	protein_coding	54342	-
ENSMUSG00000037740	33.3	0.164	-2.608	6.85123E-06	7.32796E-05	Mrps26	mitochondrial ribosomal protein S26	protein_coding	99045	-
ENSMUSG00000037771	5.8	7.403	2.888	0.00291334	0.010022937	Slc32a1	solute carrier family 32 (GABA vesicular transporter), member 1	protein_coding	22348	-
ENSMUSG00000037772	17.87	0.114	-3.134	1.53655E-07	3.19563E-06	Mrpl23	mitochondrial ribosomal protein L23	protein_coding	19935	-
ENSMUSG00000037805	809.79	0.228	-2.131	3.6102E-09	1.49819E-07	Rpl10a	ribosomal protein L10A	protein_coding	19896	-
ENSMUSG00000037813	7.69	5.119	2.356	0.000310753	0.00163034	D630003M21Rik	RIKEN cDNA D630003M21 gene	protein_coding	228846	-
ENSMUSG00000037849	23.99	0.149	-2.745	3.409E-06	4.15861E-05	Ifi206	interferon activated gene 206	protein_coding	102639543	-
ENSMUSG00000037852	18.1	7.877	2.978	1.09447E-07	2.39913E-06	Cpe	carboxypeptidase E	protein_coding	12876	-
ENSMUSG00000037884	7.72	48.616	5.603	2.00906E-09	9.47529E-08	1700017G19Rik	RIKEN cDNA 1700017G19 gene	lincRNA	100040882	-
ENSMUSG00000037942	7.91	5.359	2.422	0.00091652	0.003924917	Crp	C-reactive protein, pentraxin-related	protein_coding	12944	-
ENSMUSG00000037944	240.72	0.135	-2.888	8.19496E-13	1.24793E-10	Ccr7	chemokine (C-C motif) receptor 7	protein_coding	12775	-
ENSMUSG00000037966	488.2	4.309	2.107	1.27806E-07	2.72425E-06	Ninj1	ninjurin 1	protein_coding	18081	-
ENSMUSG00000037971	214.36	5.165	2.369	1.14751E-10	8.05451E-09	1110032A03Rik	RIKEN cDNA 1110032A03 gene	protein_coding	68721	-
ENSMUSG00000037979	282.55	4.363	2.125	2.83742E-07	5.38779E-06	Ccdc92	coiled-coil domain containing 92	protein_coding	215707	-
ENSMUSG00000038000	32.41	0.226	-2.146	4.62466E-06	5.34342E-05	Acd	adrenocortical dysplasia	protein_coding	497652	-
ENSMUSG00000038007	204.09	4.263	2.092	4.19085E-07	7.4339E-06	Acer2	alkaline ceramidase 2	protein_coding	230379	-
ENSMUSG00000038011	25.68	10.348	3.371	1.4345E-12	1.93809E-10	Dnah10	dynein, axonemal, heavy chain 10	protein_coding	56087	-
ENSMUSG00000038022	20.99	5.597	2.485	4.17611E-05	0.000322894	Mindy4	MINDY lysine 48 deubiquitinase 4	protein_coding	330323	-
ENSMUSG00000038055	155.81	0.107	-3.229	9.6863E-20	4.83481E-17	Dexi	dexamethasone-induced transcript	protein_coding	58239	-
ENSMUSG00000038059	568.19	6.993	2.806	5.0458E-10	2.81577E-08	Smim3	small integral membrane protein 3	protein_coding	106878	-
ENSMUSG00000038060	12.96	7.255	2.859	0.000107759	0.00069402	Dlec1	deleted in lung and esophageal cancer 1	protein_coding	320256	-
ENSMUSG00000038115	110.92	4.718	2.238	7.09177E-10	3.79262E-08	Ano2	anoctamin 2	protein_coding	243634	-
ENSMUSG00000038143	45.1	4.981	2.316	1.93146E-06	2.60363E-05	Stox2	storkhead box 2	protein_coding	71069	-
ENSMUSG00000038168	14.04	4.093	2.033	9.54353E-05	0.000628621	P3h2	prolyl 3-hydroxylase 2	protein_coding	210530	-

ENSMUSG00000038233	4.71	8.627	3.109	0.00017614	0.001028121	Fam198a	family with sequence similarity 198, member A	protein_coding	245050	-
ENSMUSG00000038235	44.09	4.608	2.204	2.39678E-06	3.11409E-05	F11r	F11 receptor	protein_coding	16456	-
ENSMUSG00000038241	36.08	0.245	-2.026	3.79687E-07	6.82942E-06	Cep250	centrosomal protein 250	protein_coding	16328	-
ENSMUSG00000038248	32.19	5.15	2.365	2.79994E-08	7.88305E-07	Sobp	sine oculis binding protein	protein_coding	109205	-
ENSMUSG00000038255	7.28	5.237	2.389	0.005194606	0.01604088	Neurod2	neurogenic differentiation 2	protein_coding	18013	bHLH
ENSMUSG00000038267	46.94	4.74	2.245	9.81194E-09	3.42063E-07	Slc22a23	solute carrier family 22, member 23	protein_coding	73102	-
ENSMUSG00000038268	12.07	0.132	-2.923	2.94927E-06	3.69564E-05	Ovca2	candidate tumor suppressor in ovarian cancer 2	protein_coding	246257	-
ENSMUSG00000038279	17.33	0.201	-2.316	3.17601E-06	3.92231E-05	Nop2	NOP2 nucleolar protein	protein_coding	110109	-
ENSMUSG00000038296	22.18	4.267	2.093	1.96522E-05	0.000175425	Galnt18	polypeptide N-acetylgalactosaminyltransferase 18	protein_coding	233733	-
ENSMUSG00000038299	14.7	0.127	-2.978	3.41302E-06	4.16069E-05	Wdr36	WD repeat domain 36	protein_coding	225348	-
ENSMUSG00000038331	26.88	4.489	2.167	6.99377E-07	1.12811E-05	Satb2	special AT-rich sequence binding protein 2	protein_coding	212712	CUT
ENSMUSG00000038352	62.48	0.129	-2.953	2.0124E-13	3.54518E-11	Arl5c	ADP-ribosylation factor-like 5C	protein_coding	217151	-
ENSMUSG00000038370	626.88	4.473	2.161	1.75159E-06	2.41738E-05	Pcp4l1	Purkinje cell protein 4-like 1	protein_coding	66425	-
ENSMUSG00000038379	7.1	7.473	2.902	6.4037E-05	0.000453918	Ttk	Ttk protein kinase	protein_coding	22137	-
ENSMUSG00000038416	22.02	0.214	-2.227	1.57768E-06	2.20259E-05	Cdc16	CDC16 cell division cycle 16	protein_coding	69957	-
ENSMUSG00000038421	77.58	0.036	-4.782	2.64393E-27	3.16726E-24	Fcrla	Fc receptor-like A	protein_coding	98752	-
ENSMUSG00000038437	134.57	0.218	-2.2	1.04935E-09	5.3416E-08	Mllt6	myeloid/lymphoid or mixed-lineage leukemia; translocated to, 6	protein_coding	246198	-
ENSMUSG00000038453	20.03	4.708	2.235	0.000188597	0.00108828	Srcin1	SRC kinase signaling inhibitor 1	protein_coding	56013	-
ENSMUSG00000038457	6.43	4.675	2.225	0.00931078	0.025595826	Tmem255b	transmembrane protein 255B	protein_coding	272465	-
ENSMUSG00000038462	49.85	0.232	-2.106	8.49138E-07	1.3222E-05	Uqcrfs1	ubiquinol-cytochrome c reductase, Rieske iron-sulfur polypeptide 1	protein_coding	66694	-
ENSMUSG00000038463	4.98	4.249	2.087	0.00720185	0.020832266	Olfml2b	olfactomedin-like 2B	protein_coding	320078	-
ENSMUSG00000038520	64.57	0.248	-2.01	5.86166E-08	1.43891E-06	Tbc1d17	TBC1 domain family, member 17	protein_coding	233204	-
ENSMUSG00000038527	9.05	4.719	2.238	0.000315534	0.001649168	C1rl	complement component 1, r subcomponent-like	protein_coding	232371	-
ENSMUSG00000038591	6.37	7.631	2.932	0.000147496	0.00089539	Colec10	collectin sub-family member 10	protein_coding	239447	-

ENSMUSG00000038593	173.05	0.129	-2.953	1.18038E-13	2.26046E-11	Tctn1	tectonic family member 1	protein_coding	654470	-
ENSMUSG00000038598	9.51	5.924	2.567	0.000692109	0.0031177	AI481877	expressed sequence AI481877	protein_coding	100155	-
ENSMUSG00000038762	44.41	0.249	-2.003	1.49105E-07	3.12633E-06	Abcf1	ATP-binding cassette, sub-family F (GCN20), member 1	protein_coding	224742	-
ENSMUSG00000038765	17.12	4.79	2.26	0.000154979	0.000929822	Lmx1b	LIM homeobox transcription factor 1 beta	protein_coding	16917	Homeobox
ENSMUSG00000038775	8.1	8.068	3.012	0.001551297	0.006042759	Vill	villin-like	protein_coding	22351	-
ENSMUSG00000038777	5.67	8.606	3.105	0.002350451	0.008467373	Sema6c	sema domain, transmembraneIain (TM), and cytoplasm69aladin69iza (semaphorin) 6C	protein_coding	20360	-
ENSMUSG00000038801	6	5.155	2.366	0.001191455	0.004863529	Scgb1c1	secretoglobin, family 1C, member 1	protein_coding	338417	-
ENSMUSG00000038805	11.58	6.216	2.636	0.001060672	0.004430076	Six3	sine oculis-related homeobox 3	protein_coding	20473	Homeobox
ENSMUSG00000038812	67.58	0.247	-2.018	1.34258E-08	4.35465E-07	Trmt112	tRNA methyltransferase 11-2	protein_coding	67674	-
ENSMUSG00000038816	15.45	4.437	2.149	0.005974565	0.017976719	Ctnnal1	catenin (cadherin associated protein), alpha-like 1	protein_coding	54366	-
ENSMUSG00000038845	57.37	0.199	-2.327	2.13621E-08	6.34472E-07	Phb	prohibitin	protein_coding	18673	-
ENSMUSG00000038884	10.27	0.098	-3.351	5.18751E-06	5.86623E-05	A230050P20Rik	RIKEN cDNA A230050P20 gene	protein_coding	319278	-
ENSMUSG00000038886	62.55	0.233	-2.104	1.37263E-08	4.42313E-07	Man2a2	mannosidase 2, alpha 2	protein_coding	140481	-
ENSMUSG00000038888	9.18	0.181	-2.463	0.000662722	0.003013271	Ctu1	cytosolic thiouridylase subunit 1	protein_coding	233189	-
ENSMUSG00000038902	18.34	0.192	-2.384	6.12042E-06	6.69372E-05	Pogz	pogo transposable element with ZNF domain	protein_coding	229584	-
ENSMUSG00000038903	8.63	4.031	2.011	0.012161367	0.031666077	Ccdc68	coiled-coil domain containing 68	protein_coding	381175	-
ENSMUSG00000038916	7.81	4.68	2.227	0.007462573	0.021462063	Soga3	SOGA family member 3	protein_coding	67412	-
ENSMUSG00000038932	6.48	5.19	2.376	0.003650178	0.012054778	Tcfl5	transcription factor-like 5 (basic helix-loop-helix)	protein_coding	277353	bHLH
ENSMUSG00000038991	73.78	0.126	-2.994	7.15713E-14	1.49542E-11	Txndc5	thioredoxin domain containing 5	protein_coding	105245	-
ENSMUSG00000039018	10.05	0.186	-2.424	0.000258731	0.001403725	Mtg1	mitochondrial ribosome-associated GTPase 1	protein_coding	212508	-
ENSMUSG00000039055	3.27	0.018	-5.764	9.41826E-05	0.000621508	Eme1	essential meiotic structure-specific endonuclease 1	protein_coding	268465	-
ENSMUSG00000039057	47.33	4.261	2.091	6.2677E-06	6.83815E-05	Myo16	myosin XVI	protein_coding	244281	-
ENSMUSG00000039058	24.47	6.319	2.66	3.75218E-06	4.51499E-05	Ak5	adenylate kinase 5	protein_coding	229949	-
ENSMUSG00000039099	6.13	9.34	3.223	0.000137381	0.000845583	Wdr93	WD repeat domain 93	protein_coding	626359	-

ENSMUSG00000039168	1384.43	5.743	2.522	1.03791E-08	3.55919E-07	Dap	death-associated protein	protein_coding	223453	-
ENSMUSG00000039178	10.08	4.169	2.06	0.005440884	0.01663557	Tbc1d19	TBC1 domain family, member 19	protein_coding	67249	-
ENSMUSG00000039179	8.42	10.774	3.429	2.56578E-06	3.28613E-05	Tekt5	tektin 5	protein_coding	70426	-
ENSMUSG00000039221	65.08	0.226	-2.147	1.0814E-05	0.000106741	Rpl22l1	ribosomal protein L22 like 1	protein_coding	68028	-
ENSMUSG00000039232	806.51	4.758	2.25	4.14441E-08	1.08558E-06	Stx11	syntaxin 11	protein_coding	74732	-
ENSMUSG00000039238	6.83	30.254	4.919	2.18987E-07	4.3194E-06	Zfp750	zinc finger protein 750	protein_coding	319530	-
ENSMUSG00000039264	356.66	0.178	-2.492	9.46591E-14	1.88992E-11	Gimap3	GTPase, IMAP family member 3	protein_coding	83408	-
ENSMUSG00000039323	7.69	11.997	3.585	0.00067914	0.003070819	Igfbp2	insulin-like growth factor binding protein 2	protein_coding	16008	-
ENSMUSG00000039356	13.63	0.152	-2.716	5.81624E-06	6.41863E-05	Exosc2	exosome component 2	protein_coding	227715	-
ENSMUSG00000039357	15.61	0.205	-2.283	3.87226E-05	0.000302656	Fut11	fucosyltransferase 11	protein_coding	73068	-
ENSMUSG00000039410	59.09	6.21	2.635	1.89648E-11	1.76569E-09	Prdm16	PR domain containing 16	protein_coding	70673	zf-C2H2
ENSMUSG00000039476	6.42	4.046	2.016	0.01374919	0.034801971	Prrx2	paired related homeobox 2	protein_coding	20204	Homeobox
ENSMUSG00000039509	12.88	0.193	-2.375	0.000619207	0.002852223	Nup133	nucleoporin 133	protein_coding	234865	-
ENSMUSG00000039599	86.71	0.224	-2.16	8.54898E-11	6.4007E-09	Fam149b	family with sequence similarity 149, member B	protein_coding	105428	-
ENSMUSG00000039601	21.02	4.636	2.213	3.04327E-05	0.000248792	Rcan2	regulator of calcineurin 2	protein_coding	53901	-
ENSMUSG00000039611	44.64	4.17	2.06	2.21041E-06	2.90555E-05	Tmem246	transmembrane protein 246	protein_coding	67063	-
ENSMUSG00000039617	12.69	0.081	-3.63	3.02443E-07	5.6754E-06	Gm7488	-	processed_pseudogene	-	-
ENSMUSG00000039640	38.63	0.24	-2.061	1.10997E-07	2.42937E-06	Mrpl12	mitochondrial ribosomal protein L12	protein_coding	56282	-
ENSMUSG00000039660	10.94	0.123	-3.026	2.05865E-05	0.000182316	Spout1	SPOUT domain containing methyltransferase 1	protein_coding	227695	-
ENSMUSG00000039678	37.32	0.243	-2.041	4.28024E-07	7.5775E-06	Tbc1d13	TBC1 domain family, member 13	protein_coding	70296	-
ENSMUSG00000039706	29.06	5.449	2.446	1.91378E-07	3.85957E-06	Ldb2	LIM domain binding 2	protein_coding	16826	-
ENSMUSG00000039716	26.61	4.112	2.04	8.0961E-06	8.42379E-05	Dock3	dedicator of cyto-kinesis 3	protein_coding	208869	-
ENSMUSG00000039735	11.29	4.624	2.209	0.003241754	0.010951509	Fnbp1l	formin binding protein 1-like	protein_coding	214459	-
ENSMUSG00000039738	26.68	0.183	-2.453	1.10138E-08	3.72004E-07	Slx4	SLX4 structure-specific endonuclease subunit homolog (S. cerevisiae)	protein_coding	52864	-

ENSMUSG00000 039741	23.32	6.718	2.748	1.75014E-06	2.41738E-05	Bahcc1	BAH domain and coiled-coil containing 1	protein_coding	268515	-
ENSMUSG00000 039756	27.03	0.195	-2.36	1.18062E-06	1.73463E-05	Dnttip2	deoxynucleotidyltransferase, terminal, interacting protein 2	protein_coding	99480	-
ENSMUSG00000 039763	9.94	4.216	2.076	0.005949273	0.017927635	Dnajc28	DnaJ heat shock protein family (Hsp40) member C28	protein_coding	246738	-
ENSMUSG00000 039783	29.5	0.171	-2.552	5.18041E-06	5.86189E-05	Kmo	kynurenine 3-monooxygenase (kynurenine 3-hydroxylase)	protein_coding	98256	-
ENSMUSG00000 039830	6.17	6.116	2.613	0.002407146	0.008612906	Olig2	oligodendrocyte transcription factor 2	protein_coding	50913	bHLH
ENSMUSG00000 039860	13.63	4.793	2.261	0.003870468	0.012635982	Srrm3	serine/arginine repetitive matrix 3	protein_coding	58212	-
ENSMUSG00000 039913	22.81	4.011	2.004	0.000484982	0.0023395	Pak7	p21 (RAC1) activated kinase 7	protein_coding	241656	-
ENSMUSG00000 039914	17.3	0.203	-2.301	3.24676E-06	3.99119E-05	Coq10a	coenzyme Q10A	protein_coding	210582	-
ENSMUSG00000 039954	13.79	8.091	3.016	4.79633E-07	8.3432E-06	Stk32a	serine/threonine kinase 32A	protein_coding	269019	-
ENSMUSG00000 039977	6.1	5.103	2.351	0.003064232	0.010453994	Deup1	deuterosome assembly protein 1	protein_coding	234964	-
ENSMUSG00000 040009	55.6	5.162	2.368	1.95088E-09	9.32323E-08	Gnaz	guanine nucleotide binding protein, alpha z subunit	protein_coding	14687	-
ENSMUSG00000 040048	36.08	0.221	-2.18	3.51441E-07	6.42426E-06	Ndufb10	NADH:ubiquinone oxidoreductase subunit B10	protein_coding	68342	-
ENSMUSG00000 040078	8.6	0.195	-2.356	0.000563043	0.00263678	Ptges3-ps	-	processed_pseud ogene	-	-
ENSMUSG00000 040136	14.92	6.14	2.618	7.56633E-07	1.20028E-05	Abcc8	ATP-binding cassette, sub-family C (CFTR/MRP), member 8	protein_coding	20927	-
ENSMUSG00000 040152	204.25	4.995	2.32	4.18082E-07	7.42343E-06	Thbs1	thrombospondin 1	protein_coding	21825	-
ENSMUSG00000 040219	4.16	4.816	2.268	0.016181661	0.039662839	Ttc12	tetratricopeptide repeat domain 12	protein_coding	235330	-
ENSMUSG00000 040265	50.32	6.287	2.652	1.66388E-13	3.05084E-11	Dnm3	dynamin 3	protein_coding	103967	-
ENSMUSG00000 040272	9.6	0.208	-2.264	0.004276304	0.013716692	Accs	1-aminocyclopropane-1-carboxylate synthase (non-functional)	protein_coding	329470	-
ENSMUSG00000 040310	6.05	8.233	3.041	0.000138599	0.000851157	Alx4	aristaless-like homeobox 4	protein_coding	11695	Homeobox
ENSMUSG00000 040350	56.85	0.239	-2.067	3.69289E-09	1.52196E-07	Trim7	tripartite motif-containing 7	protein_coding	94089	-
ENSMUSG00000 040415	14.28	0.107	-3.229	4.1445E-05	0.000320588	Dtx3	deltex 3, E3 ubiquitin ligase	protein_coding	80904	-
ENSMUSG00000 040429	6.41	0.169	-2.567	0.0019321	0.007208867	Mterf1a	mitochondrial transcription termination factor 1a	protein_coding	545725	-
ENSMUSG00000 040452	31.77	4.175	2.062	1.43798E-06	2.04585E-05	Cdh12	cadherin 12	protein_coding	215654	-
ENSMUSG00000 040463	30.82	0.063	-3.987	7.68664E-14	1.5876E-11	Mybbp1a	MYB binding protein (P160) 1a	protein_coding	18432	-

ENSMUSG00000 040464	6.59	0.172	-2.538	0.001422322	0.005630691	Gtpbp10	GTP-binding protein 10 (putative)	protein_coding	207704	-
ENSMUSG00000 040488	13.41	4.736	2.244	0.000561033	0.002628736	Ltbp4	latent transforming growth factor beta binding protein 4	protein_coding	108075	-
ENSMUSG00000 040490	20.85	4.625	2.209	2.69972E-05	0.000227325	Lrfn2	leucine rich repeat and fibronectin type III domain containing 2	protein_coding	70530	-
ENSMUSG00000 040532	16.43	0.241	-2.055	0.002511707	0.008901944	Abhd11	abhydrolase domain containing 11	protein_coding	68758	-
ENSMUSG00000 040592	269.27	0.007	-7.173	7.78183E-46	1.39832E-41	Cd79b	CD79B antigen	protein_coding	15985	-
ENSMUSG00000 040612	9.04	4.639	2.214	0.003731493	0.012253507	Ildr2	immunoglobulin-like domain containing receptor 2	protein_coding	100039 795	-
ENSMUSG00000 040614	5.49	6.071	2.602	0.000723409	0.003234372	Nlrp9c	NLR family, pyrin domain containing 9C	protein_coding	330490	-
ENSMUSG00000 040620	11.01	0.07	-3.829	1.82868E-07	3.70039E-06	Dhx33	DEAH (Asp-Glu-Ala-His) box polypeptide 33	protein_coding	216877	-
ENSMUSG00000 040663	76.71	0.215	-2.218	3.04792E-09	1.31971E-07	Clcf1	cardiotrophin-like cytokine factor 1	protein_coding	56708	-
ENSMUSG00000 040681	52.74	0.151	-2.73	1.16227E-08	3.88195E-07	Hmgn1	high mobility group nucleosomal binding domain 1	protein_coding	15312	-
ENSMUSG00000 040693	16.12	4.076	2.027	0.000386399	0.001940526	Slco4c1	solute carrier organic anion transporter family, member 4C1	protein_coding	227394	-
ENSMUSG00000 040722	17.03	4.883	2.288	0.000925303	0.003953408	Scamp5	secretory carrier membrane protein 5	protein_coding	56807	-
ENSMUSG00000 040732	17.16	4.114	2.041	7.19054E-05	0.000499447	Erg	ETS transcription factor	protein_coding	13876	ETS
ENSMUSG00000 040751	62.74	0.134	-2.904	2.88539E-12	3.43362E-10	Lat2	linker for activation of T cells family, member 2	protein_coding	56743	-
ENSMUSG00000 040752	11.31	6.519	2.705	3.65318E-05	0.000289435	Myh6	myosin, heavy polypeptide 6, cardiac muscle, alpha	protein_coding	17888	-
ENSMUSG00000 040794	10.95	6.895	2.786	6.1974E-05	0.000441557	C1qtnf4	C1q and tumor necrosis factor related protein 4	protein_coding	67445	-
ENSMUSG00000 040809	68.62	0.092	-3.435	2.10095E-05	0.000185149	Chil3	chitinase-like 3	protein_coding	12655	-
ENSMUSG00000 040828	7.21	7.24	2.856	0.001829522	0.006902094	Catsperd	cation channel sperm associated auxiliary subunit delta	protein_coding	106757	-
ENSMUSG00000 040852	92.05	5.04	2.333	0.002569627	0.009064315	Plekhh2	pleckstrin homology domain containing, family H (with MyTH4 domain) member 2	protein_coding	213556	-
ENSMUSG00000 040856	4.45	4.504	2.171	0.012045116	0.031434869	Dlk1	delta like non-canonical Notch ligand 1	protein_coding	13386	-
ENSMUSG00000 040899	16.85	0.172	-2.536	3.98216E-06	4.74191E-05	Ccr6	chemokine (C-C motif) receptor 6	protein_coding	12458	-
ENSMUSG00000 040957	12.93	4.301	2.105	0.001050712	0.004396112	Cables1	CDK5 and Abl enzyme substrate 1	protein_coding	63955	-
ENSMUSG00000 040969	9.01	5.229	2.387	0.003370372	0.011296495	Arhgef38	Rho guanine nucleotide exchange factor (GEF) 38	protein_coding	77669	-
ENSMUSG00000 040972	35.28	5.329	2.414	6.1287E-08	1.4882E-06	Igsf21	immunoglobulin superfamily, member 21	protein_coding	230868	-

ENSMUSG00000 040998	6.12	4.033	2.012	0.006214478	0.018558743	Npnt	nephronectin	protein_coding	114249	-
ENSMUSG00000 041037	19.63	0.212	-2.24	6.17177E-05	0.000440082	Irgq	immunity-related GTPase family, Q	protein_coding	210146	-
ENSMUSG00000 041078	69.09	5.394	2.431	2.06333E-08	6.18964E-07	Grid1	glutamate receptor, ionotropic, delta 1	protein_coding	14803	-
ENSMUSG00000 041130	36.11	0.234	-2.095	1.51693E-06	2.13618E-05	Zfp598	zinc finger protein 598	protein_coding	213753	-
ENSMUSG00000 041193	8.04	5.93	2.568	3.85245E-05	0.000301632	Pla2g5	phospholipase A2, group V	protein_coding	18784	-
ENSMUSG00000 041216	17.64	4.664	2.222	4.202E-05	0.000323781	Clvs1	clavesin 1	protein_coding	74438	-
ENSMUSG00000 041220	39.31	4.072	2.026	3.14062E-07	5.82397E-06	Elovl6	ELOVL family member 6, elongation of long chain fatty acids (yeast)	protein_coding	170439	-
ENSMUSG00000 041309	11.41	4.268	2.094	0.00431001	0.013810016	Nkx6-2	NK6 homeobox 2	protein_coding	14912	Homeobox
ENSMUSG00000 041360	20.97	0.21	-2.255	2.90192E-05	0.000240298	Pum3	pumilio RNA-binding family member 3	protein_coding	52874	-
ENSMUSG00000 041372	12	9.027	3.174	2.41117E-06	3.13052E-05	B4galnt3	beta-1,4-N-acetyl-galactosaminyl transferase 3	protein_coding	330406	-
ENSMUSG00000 041377	13.05	4.302	2.105	0.000814377	0.003569158	Ninj2	ninjurin 2	protein_coding	29862	-
ENSMUSG00000 041380	20.4	4.08	2.029	7.25373E-05	0.000502573	Htr2c	5-hydroxytryptamine (serotonin) receptor 2C	protein_coding	15560	-
ENSMUSG00000 041396	5.7	0.093	-3.433	8.21937E-05	0.000556076	Mettl18	methyltransferase like 18	protein_coding	69962	-
ENSMUSG00000 041444	16.39	5.532	2.468	7.23513E-06	7.67009E-05	Arhgap32	Rho GTPase activating protein 32	protein_coding	330914	-
ENSMUSG00000 041481	12.85	0.115	-3.123	0.000214175	0.001206355	Serpina3g	serine (or cysteine) peptidase inhibitor, clade A, member 3G	protein_coding	20715	-
ENSMUSG00000 041498	17.42	5.187	2.375	1.55312E-05	0.000144078	Kif14	kinesin family member 14	protein_coding	381293	-
ENSMUSG00000 041506	22.41	0.137	-2.865	6.60138E-08	1.58583E-06	Rrp9	RRP9, small subunit (SSU) processome component, homolog (yeast)	protein_coding	27966	-
ENSMUSG00000 041515	120.6	0.08	-3.636	5.68406E-21	3.40456E-18	Irf8	interferon regulatory factor 8	protein_coding	15900	IRF
ENSMUSG00000 041538	168.61	0.017	-5.902	1.53508E-27	1.97027E-24	H2-Ob	histocompatibility 2, O region beta locus	protein_coding	15002	-
ENSMUSG00000 041544	15.57	4.894	2.291	3.48291E-05	0.000279645	Disp3	dispatched RND transporter family member 3	protein_coding	242748	-
ENSMUSG00000 041560	375.51	0.247	-2.02	2.23643E-09	1.02256E-07	Nop53	NOP53 ribosome biogenesis factor	protein_coding	68077	-
ENSMUSG00000 041598	26.8	4.435	2.149	1.9979E-05	0.000177901	Cdc42ep4	CDC42 effector protein (Rho GTPase binding) 4	protein_coding	56699	-
ENSMUSG00000 041608	23.72	8.823	3.141	1.96335E-09	9.35793E-08	Entpd3	ectonucleoside triphosphate diphosphohydrolase 3	protein_coding	215446	-

ENSMUSG00000041617	7.11	4.453	2.155	0.012878863	0.033116813	Ccdc74a	coiled-coil domain containing 74A	protein_coding	72315	-
ENSMUSG00000041644	4.57	5.675	2.505	0.005112143	0.015833372	Slc5a12	solute carrier family 5 (sodium/glucose cotransporter), member 12	protein_coding	241612	-
ENSMUSG00000041671	21.7	0.217	-2.206	5.29992E-05	0.000390145	Pyroxd1	pyridine nucleotide-disulphide oxidoreductase domain 1	protein_coding	232491	-
ENSMUSG00000041674	10.52	4.242	2.085	0.015749792	0.038845213	BC006965	cDNA sequence BC006965	processed_transcript	217294	-
ENSMUSG00000041695	43.59	4.198	2.07	9.73876E-08	2.18535E-06	Kcnj2	potassium inwardly-rectifying channel, subfamily J, member 2	protein_coding	16518	-
ENSMUSG00000041708	15.88	6.28	2.651	3.36118E-07	6.16925E-06	Mpped1	metallophosphoesterase domain containing 1	protein_coding	223726	-
ENSMUSG00000041731	21.32	6.179	2.627	4.93237E-08	1.24131E-06	Pgm5	phosphoglucomutase 5	protein_coding	226041	-
ENSMUSG00000041733	11.67	0.227	-2.139	0.001379682	0.005481208	Coq5	coenzyme Q5 methyltransferase	protein_coding	52064	-
ENSMUSG00000041734	24.71	10.735	3.424	1.67592E-07	3.42211E-06	Kirrel	kirre like nephrin family adhesion molecule 1	protein_coding	170643	-
ENSMUSG00000041737	3.65	7.499	2.907	0.000758511	0.003359546	Tmem45b	transmembrane protein 45b	protein_coding	235135	-
ENSMUSG00000041741	18.85	4.487	2.166	5.87704E-05	0.000423745	Pde3a	phosphodiesterase 3A, cGMP inhibited	protein_coding	54611	-
ENSMUSG00000041765	204.66	0.247	-2.018	3.24732E-06	3.99119E-05	Ubac2	ubiquitin associated domain containing 2	protein_coding	68889	-
ENSMUSG00000041782	3.94	4.761	2.251	0.013665764	0.034649373	Lad1	ladinin	protein_coding	16763	-
ENSMUSG00000041801	72.62	9.077	3.182	2.19677E-10	1.37062E-08	Phlda3	pleckstrin homology like domain, family A, member 3	protein_coding	27280	-
ENSMUSG00000041886	13.41	5.064	2.34	0.00020413	0.001160396	Macc1	metastasis associated in colon cancer 1	protein_coding	238455	-
ENSMUSG00000041907	8.84	6.602	2.723	0.001869295	0.007015323	Gpr45	G protein-coupled receptor 45	protein_coding	93690	-
ENSMUSG00000041957	6.78	5.047	2.335	0.006163971	0.018444696	Pkp2	plakophilin 2	protein_coding	67451	-
ENSMUSG00000041986	7.09	6.645	2.732	0.016133533	0.03956646	Elmod1	ELMO/CED-12 domain containing 1	protein_coding	270162	-
ENSMUSG00000042063	19.87	0.182	-2.46	0.00011272	0.000719016	Zfp386	zinc finger protein 386 (Kruppel-like)	protein_coding	56220	zf-C2H2
ENSMUSG00000042104	17.45	5.931	2.568	0.000103313	0.000671403	Uggt2	UDP-glucose glycoprotein glucosyltransferase 2	protein_coding	66435	-
ENSMUSG00000042111	21.26	0.185	-2.433	6.3159E-06	6.87404E-05	Ccdc115	coiled-coil domain containing 115	protein_coding	69668	-
ENSMUSG00000042213	47.71	6.093	2.607	8.11734E-08	1.86909E-06	Zfand4	zinc finger, AN1-type domain 4	protein_coding	67492	-
ENSMUSG00000042216	10.98	4.306	2.106	0.00023171	0.001287445	Sgsm1	small G protein signaling modulator 1	protein_coding	52850	-
ENSMUSG00000042265	104.01	4.33	2.114	5.9929E-11	4.66175E-09	Trem1	triggering receptor expressed on myeloid cells 1	protein_coding	58217	-

ENSMUSG00000042286	11.63	7.397	2.887	0.000238497	0.00131504	Stab1	stabilin 1	protein_coding	192187	-
ENSMUSG00000042401	14.17	4.488	2.166	0.000725385	0.003239982	Crtac1	cartilage acidic protein 1	protein_coding	72832	-
ENSMUSG00000042474	105.28	0.018	-5.804	1.27412E-28	2.07289E-25	Fcmr	Fc fragment of IgM receptor	protein_coding	69169	-
ENSMUSG00000042477	6.29	5.263	2.396	0.004776181	0.014972159	Tfap2e	transcription factor AP-2, epsilon	protein_coding	332937	AP-2
ENSMUSG00000042529	8.12	6.544	2.71	0.003934478	0.012800767	Kcnj12	potassium inwardly-rectifying channel, subfamily J, member 12	protein_coding	16515	-
ENSMUSG00000042540	9.14	4.491	2.167	0.002607354	0.009168601	Acot5	acyl-CoA thioesterase 5	protein_coding	217698	-
ENSMUSG00000042567	24.48	4.355	2.123	0.000113204	0.000719959	Nek10	NIMA (never in mitosis gene a)-related kinase 10	protein_coding	674895	-
ENSMUSG00000042579	7.36	0.141	-2.825	0.000889786	0.00383327	4632404H12Rik	RIKEN cDNA 4632404H12 gene	lincRNA	74034	-
ENSMUSG00000042590	397.89	4.996	2.321	8.33412E-10	4.34075E-08	Ipo11	importin 11	protein_coding	76582	-
ENSMUSG00000042686	21.4	4.29	2.101	0.001865639	0.007006091	Jph1	junctophilin 1	protein_coding	57339	-
ENSMUSG00000042699	43.76	0.174	-2.524	2.2577E-07	4.43373E-06	Dhx9	DEAH (Asp-Glu-Ala-His) box polypeptide 9	protein_coding	13211	-
ENSMUSG00000042729	14.97	0.139	-2.852	2.1064E-06	2.79748E-05	Wdr74	WD repeat domain 74	protein_coding	107071	-
ENSMUSG00000042734	36.9	0.139	-2.852	0.000252113	0.001376546	Ttc9	tetratricopeptide repeat domain 9	protein_coding	69480	-
ENSMUSG00000042737	23.87	0.202	-2.311	2.82594E-05	0.000235416	Dpm3	dolichyl-phosphate mannosyltransferase polypeptide 3	protein_coding	68563	-
ENSMUSG00000042747	30.18	0.164	-2.612	4.22303E-08	1.09658E-06	Krtcap2	keratinocyte associated protein 2	protein_coding	66059	-
ENSMUSG00000042831	12.52	0.122	-3.031	4.29618E-06	5.03247E-05	Alkbh6	alkB homolog 6	protein_coding	233065	-
ENSMUSG00000042846	14.6	6.502	2.701	8.08847E-05	0.000549288	Lrrtm3	leucine rich repeat transmembrane neuronal 3	protein_coding	216028	-
ENSMUSG00000042961	15.64	4.448	2.153	0.001288767	0.005191178	Egflam	EGF-like, fibronectin type III and laminin G domains	protein_coding	268780	-
ENSMUSG00000042978	152.85	0.115	-3.115	7.95841E-12	8.31423E-10	Sbk1	SH3-binding kinase 1	protein_coding	104175	-
ENSMUSG00000043029	5.52	7.327	2.873	0.000450094	0.002207355	Trpv3	transient receptor potential cation channel, subfamily V, member 3	protein_coding	246788	-
ENSMUSG00000043061	16.55	0.089	-3.493	1.29035E-08	4.23882E-07	Tmem18	transmembrane protein 18	protein_coding	211986	-
ENSMUSG00000043090	29.04	0.216	-2.208	1.60354E-07	3.30437E-06	Zfp866	zinc finger protein 866	protein_coding	330788	zf-C2H2
ENSMUSG00000043140	15.44	0.034	-4.876	9.09973E-11	6.70135E-09	Tmem186	transmembrane protein 186	protein_coding	66690	-
ENSMUSG00000043243	32.51	0.116	-3.111	2.26015E-11	2.03063E-09	Fam129c	family with sequence similarity 129, member C	protein_coding	100037278	-

ENSMUSG00000 043252	35.79	0.231	-2.111	5.55745E-06	6.21804E-05	Tmem64	transmembrane protein 64	protein_coding	100201	-
ENSMUSG00000 043259	18.24	6.279	2.65	1.35482E-05	0.000128876	Fam13c	family with sequence similarity 13, member C	protein_coding	71721	-
ENSMUSG00000 043263	90.81	0.09	-3.471	1.75599E-11	1.65201E-09	Ifi209	interferon activated gene 209	protein_coding	236312	-
ENSMUSG00000 043290	8.18	0.204	-2.294	0.000717507	0.00321198	Zfp784	zinc finger protein 784	protein_coding	654801	zf-C2H2
ENSMUSG00000 043300	6.76	4.278	2.097	0.013042948	0.033395373	B3galnt1	UDP-GalNAc:betaGlcNAc beta 1,3-galactosaminyltransferase, polypeptide 1	protein_coding	26879	-
ENSMUSG00000 043391	22.26	4.163	2.058	0.000207751	0.001177917	2510009E07R ik	RIKEN cDNA 2510009E07 gene	protein_coding	72190	-
ENSMUSG00000 043456	43.83	5.375	2.426	4.55105E-05	0.000346663	Zfp536	zinc finger protein 536	protein_coding	243937	zf-C2H2
ENSMUSG00000 043461	4.27	4.071	2.026	0.007898913	0.022436858	Sptssb	serine palmitoyltransferase, small subunit B	protein_coding	66183	-
ENSMUSG00000 043483	28.48	0.052	-4.259	2.48654E-15	6.76981E-13	Gm6863	-	processed_pseud ogene	-	-
ENSMUSG00000 043518	12.8	4.959	2.31	0.001688741	0.00646739	Rai2	retinoic acid induced 2	protein_coding	24004	-
ENSMUSG00000 043639	27.01	4.052	2.019	5.54286E-05	0.000404548	Rbm20	RNA binding motif protein 20	protein_coding	73713	-
ENSMUSG00000 043659	17.12	5.023	2.329	0.000136202	0.000840459	Npsr1	neuropeptide S receptor 1	protein_coding	319239	-
ENSMUSG00000 043668	18.39	4.634	2.212	0.000234553	0.001298806	Tox3	TOX high mobility group box family member 3	protein_coding	244579	HMG
ENSMUSG00000 043683	44.07	0.22	-2.187	5.6566E-09	2.19059E-07	Fem1a	feminization 1 homolog a (C. elegans)	protein_coding	14154	-
ENSMUSG00000 043705	15.79	4.693	2.231	0.00017381	0.001017659	Capn13	calpain 13	protein_coding	381122	-
ENSMUSG00000 043719	14.96	4.291	2.101	3.56406E-05	0.000284634	Col6a6	collagen, type VI, alpha 6	protein_coding	245026	-
ENSMUSG00000 043789	4.69	5.278	2.4	0.010043025	0.027194561	Vwce	von Willebrand factor C and EGF domains	protein_coding	71768	-
ENSMUSG00000 043843	3.65	5.892	2.559	0.006543756	0.019311012	Tmem145	transmembrane protein 145	protein_coding	330485	-
ENSMUSG00000 043913	27.35	5.765	2.527	6.06363E-08	1.47839E-06	Ccdc60	coiled-coil domain containing 60	protein_coding	269693	-
ENSMUSG00000 043924	14.53	0.214	-2.221	0.007520868	0.021595156	Ncmap	noncompact myelin associated protein	protein_coding	230822	-
ENSMUSG00000 043931	30.95	0.09	-3.469	3.21294E-09	1.36809E-07	Gimap7	GTPase, IMAP family member 7	protein_coding	231932	-
ENSMUSG00000 043940	53.47	4.46	2.157	3.10956E-07	5.78449E-06	Wdfy3	WD repeat and FYVE domain containing 3	protein_coding	72145	-
ENSMUSG00000 043953	24.33	4.433	2.148	1.00146E-06	1.51348E-05	Ccrl2	chemokine (C-C motif) receptor-like 2	protein_coding	54199	-
ENSMUSG00000 044037	79.72	0.152	-2.72	1.15485E-13	2.26046E-11	Als2cl	ALS2 C-terminal like	protein_coding	235633	-

ENSMUSG00000044042	26.02	4.06	2.021	2.14863E-05	0.000188152	Fmn1	formin 1	protein_coding	14260	-
ENSMUSG00000044052	3.49	0.018	-5.805	7.17203E-05	0.000498422	Ccr10	chemokine (C-C motif) receptor 10	protein_coding	12777	-
ENSMUSG00000044080	645.95	5.644	2.497	7.89414E-08	1.83505E-06	S100a1	S100 calcium binding protein A1	protein_coding	20193	-
ENSMUSG00000044122	7.53	5.921	2.566	0.00388413	0.012671374	Proca1	protein interacting with cyclin A1	protein_coding	216974	-
ENSMUSG00000044155	13.41	0.114	-3.134	1.96434E-07	3.93942E-06	Lsm8	LSM8 homolog, U6 small nuclear RNA associated	protein_coding	76522	-
ENSMUSG00000044199	155.54	0.188	-2.412	1.18486E-12	1.67643E-10	S1pr4	sphingosine-1-phosphate receptor 4	protein_coding	13611	-
ENSMUSG00000044216	5.02	7.44	2.895	0.000287713	0.00152775	Kcnj4	potassium inwardly-rectifying channel, subfamily J, member 4	protein_coding	16520	-
ENSMUSG00000044252	23.96	4.477	2.163	6.13682E-05	0.000438633	Osbpl1a	oxysterol binding protein-like 1A	protein_coding	64291	-
ENSMUSG00000044258	13194.53	4.652	2.218	5.53761E-05	0.000404548	Ctla2a	cytotoxic T lymphocyte-associated protein 2 alpha	protein_coding	13024	-
ENSMUSG00000044288	5.86	10.594	3.405	0.001030232	0.004325288	Cnr1	cannabinoid receptor 1 (brain)	protein_coding	12801	-
ENSMUSG00000044365	6.47	4.609	2.204	0.002245375	0.008154232	Cxxc4	CXXC finger 4	protein_coding	319478	-
ENSMUSG00000044393	4.71	4.066	2.024	0.006553236	0.019332638	Dsg2	desmoglein 2	protein_coding	13511	-
ENSMUSG00000044576	6.97	9.025	3.174	0.000250513	0.001369061	Garem2	GRB2 associated regulator of MAPK1 subtype 2	protein_coding	242915	-
ENSMUSG00000044609	4.01	0.139	-2.844	0.005101109	0.015817218	Gm9294	-	processed_pseudogene	-	-
ENSMUSG00000044730	9.39	0.113	-3.148	1.45838E-05	0.000136981	9930104L06Rik	RIKEN cDNA 9930104L06 gene	processed_transcript	194268	-
ENSMUSG00000044734	64.01	0.084	-3.568	9.56289E-16	2.86393E-13	Serpinb1a	serine (or cysteine) peptidase inhibitor, clade B, member 1a	protein_coding	66222	-
ENSMUSG00000044763	18.31	0.084	-3.575	3.639E-09	1.5032E-07	Trmt10c	tRNA methyltransferase 10C	protein_coding	52575	-
ENSMUSG00000044867	46.92	0.2	-2.319	4.08337E-08	1.07272E-06	Gimap1os	-	processed_transcript	-	-
ENSMUSG00000044997	8.3	5.666	2.502	0.004359722	0.013927085	E130304I02Rik	RIKEN cDNA E130304I02 gene	antisense_RNA	78547	-
ENSMUSG00000045004	8.2	8.703	3.122	0.000104159	0.000676414	Spata21	spermatogenesis associated 21	protein_coding	329972	-
ENSMUSG00000045039	10.11	4.212	2.074	0.002129497	0.007812359	Megf8	multiple EGF-like-domains 8	protein_coding	269878	-
ENSMUSG00000045055	100.17	0.147	-2.763	7.76341E-15	2.02175E-12	Rpsa-ps2	-	processed_pseudogene	-	-
ENSMUSG00000045075	5.99	13.077	3.709	7.80361E-06	8.16675E-05	Gm9796	-	lincRNA	-	-
ENSMUSG00000045092	169.77	0.15	-2.735	1.32133E-13	2.47323E-11	S1pr1	sphingosine-1-phosphate receptor 1	protein_coding	13609	-

ENSMUSG00000045095	65.11	4.512	2.174	1.5015E-07	3.14092E-06	Magi1	membrane associated guanylate kinase, WW and PDZ domain containing 1	protein_coding	14924	-
ENSMUSG00000045106	84.73	0.197	-2.341	5.8131E-12	6.33065E-10	Ccdc73	coiled-coil domain containing 73	protein_coding	211936	-
ENSMUSG00000045132	3050.51	9.696	3.277	0.01015754	0.027446742	Olfr620	olfactory receptor 620	protein_coding	258808	-
ENSMUSG00000045180	14.41	4.514	2.175	0.00019146	0.001102677	Shroom2	shroom family member 2	protein_coding	110380	-
ENSMUSG00000045201	12.48	8.461	3.081	3.89511E-05	0.00030431	Lrrc3b	leucine rich repeat containing 3B	protein_coding	218763	-
ENSMUSG00000045215	14.09	5.93	2.568	0.00038695	0.001942751	Asxl3	additional sex combs like 3, transcriptional regulator	protein_coding	211961	-
ENSMUSG00000045238	5.57	24.928	4.64	2.41306E-06	3.13071E-05	A730035I17Rik	-	antisense_RNA	-	-
ENSMUSG00000045294	14.46	0.072	-3.801	2.30142E-08	6.73521E-07	Insig1	insulin induced gene 1	protein_coding	231070	-
ENSMUSG00000045319	65.91	5.458	2.448	8.58999E-07	1.33408E-05	Proser2	proline and serine rich 2	protein_coding	227545	-
ENSMUSG00000045328	5.98	5.134	2.36	0.002828508	0.009791073	Cenpe	centromere protein E	protein_coding	229841	-
ENSMUSG00000045411	31.4	0.137	-2.869	2.47687E-10	1.51384E-08	2410002F23Rik	RIKEN cDNA 2410002F23 gene	protein_coding	668661	-
ENSMUSG00000045463	6.74	5.77	2.528	0.004345638	0.01388703	Slco6b1	solute carrier organic anion transporter family, member 6b1	transcribed_unprocessed_pseudogene	67854	-
ENSMUSG00000045466	15.61	0.247	-2.019	0.003143515	0.010671797	Zfp956	zinc finger protein 956	protein_coding	101197	zf-C2H2
ENSMUSG00000045551	42.98	5.932	2.568	2.56399E-09	1.1547E-07	Fpr1	formyl peptide receptor 1	protein_coding	14293	-
ENSMUSG00000045744	7.17	0.076	-3.726	5.46667E-05	0.000401105	Bricd5	BRICHOS domain containing 5	protein_coding	319259	-
ENSMUSG00000045757	10.81	0.05	-4.317	5.38921E-07	9.13749E-06	Zfp764	zinc finger protein 764	protein_coding	233893	zf-C2H2
ENSMUSG00000045761	6.22	4.045	2.016	0.004384052	0.013990325	Togaram2	TOG array regulator of axonemal microtubules 2	protein_coding	320159	-
ENSMUSG00000045775	9.73	0.109	-3.198	1.06698E-05	0.000105402	Slc16a5	solute carrier family 16 (monocarboxylic acid transporters), member 5	protein_coding	217316	-
ENSMUSG00000045780	3036.69	9.947	3.314	0.019466558	0.046090753	Olfr624	olfactory receptor 624	protein_coding	258189	-
ENSMUSG00000045799	50.5	0.154	-2.694	2.61739E-12	3.22137E-10	Gm9800	-	processed_pseudogene	-	-
ENSMUSG00000045802	11.75	5.678	2.505	0.002181431	0.007968722	Hsf3	heat shock transcription factor 3	protein_coding	245525	HSF
ENSMUSG00000045826	210.12	0.107	-3.23	2.30728E-20	1.29561E-17	Ptprcap	protein tyrosine phosphatase, receptor type, C polypeptide-associated protein	protein_coding	19265	-

ENSMUSG00000045838	4.6	9.314	3.219	0.003027535	0.010350413	A430105I19Rik	coiled-coil domain containing 9B	protein_coding	214239	-
ENSMUSG00000045875	12.15	4.233	2.082	0.000598813	0.002776077	Adra1a	adrenergic receptor, alpha 1a	protein_coding	11549	-
ENSMUSG00000046031	15.72	0.102	-3.294	1.92032E-06	2.59337E-05	Fam26f	calcium homeostasis modulator family member 6	protein_coding	215900	-
ENSMUSG00000046159	56.17	4.257	2.09	2.86209E-08	7.99518E-07	Chrm3	cholinergic receptor, muscarinic 3, cardiac	protein_coding	12671	-
ENSMUSG00000046321	10.39	4.037	2.013	0.002249483	0.008165369	Hs3st2	heparan sulfate (glucosamine) 3-O-sulfotransferase 2	protein_coding	195646	-
ENSMUSG00000046337	19.77	4.821	2.269	4.85211E-05	0.000364801	Fam178b	family with sequence similarity 178, member B	protein_coding	381337	-
ENSMUSG00000046351	9.68	0.239	-2.064	0.013876519	0.035035432	Zfp322a	zinc finger protein 322A	protein_coding	218100	zf-C2H2
ENSMUSG00000046434	141.85	0.177	-2.5	4.31989E-14	9.3523E-12	Hnrnpa1	heterogeneous nuclear ribonucleoprotein A1	protein_coding	15382	-
ENSMUSG00000046463	7.7	4.216	2.076	0.013048077	0.033403746	5930403N24Rik	-	processed_transcript	-	-
ENSMUSG00000046561	7.43	6.91	2.789	0.00039017	0.001957824	Arsj	arylsulfatase J	protein_coding	271970	-
ENSMUSG00000046572	7.77	6.918	2.79	0.000119424	0.00075164	Zfp518b	zinc finger protein 518B	protein_coding	100515	-
ENSMUSG00000046585	17.48	6.955	2.798	2.76409E-08	7.83407E-07	Cfap58	cilia and flagella associated protein 58	protein_coding	381229	-
ENSMUSG00000046591	5.92	6.835	2.773	0.002564275	0.009052547	Ticrr	TOPBP1-interacting checkpoint and replication regulator	protein_coding	77011	-
ENSMUSG00000046607	7.14	4.719	2.239	0.006369789	0.0179aladin 79iHrk	harakiri, BCL2 interacting protein (contains only BH3 domain)	protein_coding	12123	-	
ENSMUSG00000046613	5.85	4.896	2.292	0.009096094	0.025139159	Vwa5b2	von Willebrand factor A domain containing 5B2	protein_coding	328643	-
ENSMUSG00000046637	6.42	28.213	4.818	1.45695E-07	3.06796E-06	Ttc34	tetratricopeptide repeat domain 34	protein_coding	242800	-
ENSMUSG00000046675	10.18	0.102	-3.298	2.03401E-06	2.71136E-05	Tmem251	transmembrane protein 251	protein_coding	320351	-
ENSMUSG00000046688	42.36	0.22	-2.182	3.13955E-07	5.82397E-06	Tifa	TRAF-interacting protein with forkhead-associated domain	protein_coding	211550	-
ENSMUSG00000046721	24.5	0.164	-2.608	1.9407E-06	2.61412E-05	Rpl14-ps1	ribosomal protein L14, pseudogene 1	processed_pseudogene	100040970	-
ENSMUSG00000046743	15.2	4.617	2.207	9.89582E-06	9.86814E-05	Fat4	FAT atypical cadherin 4	protein_coding	329628	-
ENSMUSG00000046761	9.13	4.075	2.027	0.007964087	0.022579155	Fam83h	family with sequence similarity 83, member H	protein_coding	105732	-
ENSMUSG00000046768	69.96	4.11	2.039	1.62891E-05	0.000149948	Rhoj	ras homolog family member J	protein_coding	80837	-
ENSMUSG00000046794	36.35	5.235	2.388	3.72205E-10	2.11651E-08	Ppp1r3b	protein phosphatase 1, regulatory subunit 3B	protein_coding	244416	-
ENSMUSG00000046806	458.38	4.177	2.063	1.08819E-06	1.62003E-05	3110062M04Rik	cell cycle regulator of NHEJ	protein_coding	78412	-

ENSMUSG00000 046814	621.32	5.938	2.57	1.99849E-10	1.26447E-08	Gchfr	GTP cyclohydrolase I feedback regulator	protein_coding	320415	-
ENSMUSG00000 046865	46.45	0.231	-2.113	9.71315E-07	1.47911E-05	Fbl	fibrillarin	protein_coding	14113	-
ENSMUSG00000 046916	210.07	5.739	2.521	7.45101E-11	5.67318E-09	Myct1	myc target 1	protein_coding	68632	-
ENSMUSG00000 046959	8.09	6.74	2.753	1.81818E-05	0.000164589	Slc26a1	solute carrier family 26 (sulfate transporter), member 1	protein_coding	231583	-
ENSMUSG00000 047013	13.18	7.427	2.893	9.56194E-05	0.000629372	Fbxo41	F-box protein 41	protein_coding	330369	-
ENSMUSG00000 047036	49.55	0.119	-3.073	1.10302E-11	1.1135E-09	Zfp445	zinc finger protein 445	protein_coding	235682	zf-C2H2
ENSMUSG00000 047123	12.49	0.15	-2.738	6.42697E-05	0.000455387	Ticam1	toll-like receptor adaptor molecule 1	protein_coding	106759	-
ENSMUSG00000 047129	11.15	4.097	2.035	0.008164559	0.02303485	1700113H08R ik	RIKEN cDNA 1700113H08 gene	protein_coding	76640	-
ENSMUSG00000 047250	53.72	7.357	2.879	1.16939E-11	1.15454E-09	Ptgs1	prostaglandin-endoperoxide synthase 1	protein_coding	19224	-
ENSMUSG00000 047324	6.39	6.457	2.691	0.006558962	0.019346353	4931429P17R ik	RIKEN cDNA 4931429P17 gene	lincRNA	70971	-
ENSMUSG00000 047409	55.15	4.888	2.289	8.03805E-09	2.87721E-07	Ctdspl	CTD (carboxy-terminal domain, RNA polymerase II, polypeptide A) small phosphatase-like	protein_coding	69274	-
ENSMUSG00000 047420	6.16	7.301	2.868	0.000427313	0.00211293	Fam180a	family with sequence similarity 180, member A	protein_coding	208164	-
ENSMUSG00000 047441	4.37	5.211	2.382	0.013074335	0.033442381	Antxrl	anthrax toxin receptor-like	protein_coding	239029	-
ENSMUSG00000 047591	9.36	4.962	2.311	0.004163113	0.013418292	Mafa	v-maf musculoaponeurotic fibrosarcoma oncogene family, protein A (avian)	protein_coding	378435	TF_bZIP
ENSMUSG00000 047613	12.81	0.161	-2.632	2.11428E-05	0.000186142	A430005L14 Rik	RIKEN cDNA A430005L14 gene	protein_coding	97159	-
ENSMUSG00000 047636	8.42	5.61	2.488	0.000924826	0.003952949	Cdcp2	CUB domain containing protein 2	protein_coding	242603	-
ENSMUSG00000 047676	91.96	0.164	-2.613	3.03419E-13	5.09545E-11	Rpsa-ps10	-	processed_pseud ogene	-	-
ENSMUSG00000 047694	11.47	0.165	-2.598	0.000151911	0.000916616	Yipf6	Yip1 domain family, member 6	protein_coding	77929	-
ENSMUSG00000 047721	64.01	0.244	-2.034	1.60863E-08	5.018380ala7	Bola2	bolA-like 2 (E. coli)	protein_coding	66162	-
ENSMUSG00000 047747	20.64	4.484	2.165	0.00011373	0.000721869	Rnf150	ring finger protein 150	protein_coding	330812	-
ENSMUSG00000 047766	9.84	5.262	2.396	0.000191802	0.001103763	Lrrc49	leucine rich repeat containing 49	protein_coding	102747	-
ENSMUSG00000 047767	68.18	0.229	-2.129	1.50869E-08	4.78126E-07	Atg16l2	autophagy related 16-like 2 (S. cerevisiae)	protein_coding	73683	-
ENSMUSG00000 047867	314.14	0.197	-2.341	9.28355E-12	9.53234E-10	Gimap6	GTPase, IMAP family member 6	protein_coding	231931	-

ENSMUSG00000047880	75.66	0.196	-2.353	7.74203E-10	4.09166E-08	Cxcr5	chemokine (C-X-C motif) receptor 5	protein_coding	12145	-
ENSMUSG00000047953	224.38	5.219	2.384	9.22421E-07	1.42031E-05	Gp5	glycoprotein 5 (platelet)	protein_coding	14729	-
ENSMUSG00000047986	7.52	0.178	-2.493	0.004741577	0.01487974	Palm3	paralemmin 3	protein_coding	74337	-
ENSMUSG00000048078	81.22	4.018	2.007	2.77825E-08	7.84942E-07	Tenm4	teneurin transmembrane protein 4	protein_coding	23966	-
ENSMUSG00000048096	8.17	10.107	3.337	6.9453E-06	7.40758E-05	Lmod1	leiomodin 1 (smooth muscle)	protein_coding	93689	-
ENSMUSG00000048126	8.77	4.001	2	0.01058826	0.028426781	Col6a3	collagen, type VI, alpha 3	protein_coding	12835	-
ENSMUSG00000048186	14.4	7.161	2.84	9.19749E-07	1.41741E-05	Bend7	BEN domain containing 7	protein_coding	209645	-
ENSMUSG00000048215	77.03	0.11	-3.185	1.37423E-10	9.214E-09	A630023P12Rik	RIKEN cDNA A630023P12 gene	lincRNA	231603	-
ENSMUSG00000048316	8.88	0.114	-3.137	0.000105438	0.000683479	Gm9823	-	pseudogene	-	-
ENSMUSG00000048332	35.45	4.152	2.054	1.03487E-06	1.55482E-05	Lhfp	lipoma HMGIC fusion partner	protein_coding	108927	-
ENSMUSG00000048349	9.59	4.228	2.08	0.01123886	0.029786294	Pou4f1	POU domain, class 4, transcription factor 1	protein_coding	18996	Pou
ENSMUSG00000048537	17.62	4.067	2.024	4.52223E-05	0.000344906	Phldb1	pleckstrin homology like domain, family B, member 1	protein_coding	102693	-
ENSMUSG00000048583	7.56	7.759	2.956	4.8091E-05	0.000362558	Igf2	insulin-like growth factor 2	protein_coding	16002	-
ENSMUSG00000048644	19.06	6.091	2.607	1.1265E-06	1.66464E-05	Ctxn1	cortexin 1	protein_coding	330695	-
ENSMUSG00000048782	13.78	7.803	2.964	1.99595E-07	3.99835E-06	Insc	INSC spindle orientation adaptor protein	protein_coding	233752	-
ENSMUSG00000048834	12.39	4.904	2.294	0.003384045	0.011326371	Vstm2a	V-set and transmembrane domain containing 2A	protein_coding	211739	-
ENSMUSG00000048899	10.08	9.828	3.297	9.77278E-06	9.78858E-05	Rimkla	ribosomal modification protein rimK-like family member A	protein_coding	194237	-
ENSMUSG00000048915	31.6	4.417	2.143	7.54908E-06	7.95131E-05	Efna5	ephrin A5	protein_coding	13640	-
ENSMUSG00000048939	13.76	5.608	2.488	0.001456156	0.005740603	Atp13a5	ATPase type 13A5	protein_coding	268878	-
ENSMUSG00000048960	24.92	5.772	2.529	5.96576E-08	1.45849E-06	Prex2	phosphatidylinositol-3,4,5-trisphosphate-dependent Rac exchange factor 2	protein_coding	109294	-
ENSMUSG00000049086	18.57	0.054	-4.22	2.64895E-09	1.18406E-07	Bmyc	brain expressed myelocytomatosis oncogene	protein_coding	107771	-
ENSMUSG00000049090	44	0.15	-2.734	2.16467E-10	1.35529E-08	Zadh2	zinc binding alcohol dehydrogenase, domain containing 2	protein_coding	225791	-
ENSMUSG00000049093	5.24	4.07	2.025	0.012331522	0.031997852	Il23r	interleukin 23 receptor	protein_coding	209590	-
ENSMUSG00000049100	8	5.451	2.446	0.000507312	0.002423496	Pcdh10	protocadherin 10	protein_coding	18526	-

ENSMUSG00000049103	58.36	0.208	-2.268	6.85944E-06	7.32801E-05	Ccr2	chemokine (C-C motif) receptor 2	protein_coding	12772	-
ENSMUSG00000049107	7.18	4.709	2.235	0.002200576	0.008027234	Ntf3	neurotrophin 3	protein_coding	18205	-
ENSMUSG00000049119	69.84	5.072	2.343	1.77611E-07	3.61029E-06	Fam110b	family with sequence similarity 110, member B	protein_coding	242297	-
ENSMUSG00000049124	29.47	0.102	-3.3	5.42798E-11	4.3349E-09	Gm8186	-	protein_coding	-	-
ENSMUSG00000049130	80.59	4.304	2.106	2.90742E-08	8.08721E-07	C5ar1	complement component 5a receptor 1	protein_coding	12273	-
ENSMUSG00000049265	11.4	4.776	2.256	0.000151089	0.000912274	Kcnk3	potassium channel, subfamily K, member 3	protein_coding	16527	-
ENSMUSG00000049300	14.48	0.213	-2.231	0.000277091	0.001482263	Prmt6	protein arginine N-methyltransferase 6	protein_coding	99890	-
ENSMUSG00000049404	6.47	4.893	2.291	0.001485401	0.005826494	Rarres1	retinoic acid receptor responder (tazarotene induced) 1	protein_coding	109222	-
ENSMUSG00000049421	40.6	0.173	-2.531	1.03215E-08	3.55301E-07	Zfp260	zinc finger protein 260	protein_coding	26466	zf-C2H2
ENSMUSG00000049482	33.1	0.168	-2.576	3.13208E-10	1.82729E-08	Ctu2	cytosolic thiouridylase subunit 2	protein_coding	66965	-
ENSMUSG00000049493	44.44	4.402	2.138	3.63708E-07	6.59482E-06	Pls1	plastin 1 (I-isoform)	protein_coding	102502	-
ENSMUSG00000049538	10.34	6.528	2.707	0.000275304	0.001474059	Adamts16	a disintegrin-like and metallopeptidase (reprolysin type) with thrombospondin type 1 motif, 16	protein_coding	271127	-
ENSMUSG00000049551	6.18	6.285	2.652	0.002197395	0.008018886	Fzd9	frizzled class receptor 9	protein_coding	14371	-
ENSMUSG00000049556	31.11	4.405	2.139	3.86239E-05	0.000302016	Lingo1	leucine rich repeat and Ig domain containing 1	protein_coding	235402	-
ENSMUSG00000049600	11.67	0.244	-2.035	0.003911486	0.012745823	Zbtb45	zinc finger and BTB domain containing 45	protein_coding	232879	ZBTB
ENSMUSG00000049691	8.08	4.047	2.017	0.012218189	0.031763402	Nkx3-2	NK3 homeobox 2	protein_coding	12020	Homeobox
ENSMUSG00000049751	170.1	0.182	-2.461	1.61297E-12	2.16295E-10	Rpl36al	ribosomal protein L36A-like	protein_coding	66483	-
ENSMUSG00000049775	19942.16	4.504	2.171	4.32961E-05	0.000332189	Tmsb4x	thymosin, beta 4, X chromosome	protein_coding	19241	-
ENSMUSG00000049858	8.36	0.168	-2.575	0.002590276	0.009119253	Suox	sulfite oxidase	protein_coding	211389	-
ENSMUSG00000049957	13.01	0.159	-2.651	8.98387E-06	9.16153E-05	Ccdc137	coiled-coil domain containing 137	protein_coding	67291	-
ENSMUSG00000049960	32.54	0.24	-2.058	0.000109646	0.000703153	Mrps16	mitochondrial ribosomal protein S16	protein_coding	66242	-
ENSMUSG00000049999	40.72	5.373	2.426	7.63377E-11	5.78781E-09	Ppp1r3d	protein phosphatase 1, regulatory subunit 3D	protein_coding	228966	-
ENSMUSG00000050052	35.68	0.182	-2.459	2.80914E-08	7.88305E-07	Tdrp	testis development related protein	protein_coding	72148	-

ENSMUSG00000050075	24.3	0.221	-2.177	5.58874E-05	0.00040707	Gpr171	G protein-coupled receptor 171	protein_coding	229323	-
ENSMUSG00000050174	12.86	0.21	-2.25	0.000377151	0.001904196	Nudt6	nudix (nucleoside diphosphate linked moiety X)-type motif 6	protein_coding	229228	-
ENSMUSG00000050197	5.02	21.952	4.456	5.74669E-07	9.63268E-06	Rhox13	reproductive homeobox 13	protein_coding	73614	Homeobox
ENSMUSG00000050222	7.66	5.129	2.359	0.006118043	0.018325573	Il17d	interleukin 17D	protein_coding	239114	-
ENSMUSG00000050248	11.82	5.952	2.573	6.26846E-05	0.000445811	Evc2	EvC ciliary complex subunit 2	protein_coding	68525	-
ENSMUSG00000050272	46.46	4.686	2.228	3.13718E-08	8.60642E-07	Dscam	DS cell adhesion molecule	protein_coding	13508	-
ENSMUSG00000050295	8.49	6.783	2.762	2.71604E-05	0.000227952	Foxc1	forkhead box C1	protein_coding	17300	Fork
ENSMUSG00000050296	17.01	5.08	2.345	0.000112854	0.000719358	Abca12	ATP-binding cassette, sub-family A (ABC1), member 12	protein_coding	74591	-
ENSMUSG00000050315	17.27	4.83	2.272	0.000103503	0.000672396	Synpo2	synaptopodin 2	protein_coding	118449	-
ENSMUSG00000050321	24.34	6.885	2.783	1.6935E-08	5.24665E-07	Neto1	neuropilin (NRP) and tolloid (TLL)-like 1	protein_coding	246317	-
ENSMUSG00000050334	19.9	4.423	2.145	1.08977E-06	1.62103E-05	C130071C03Rik	RIKEN cDNA C130071C03 gene	lincRNA	320203	-
ENSMUSG00000050345	152.99	6.983	2.804	2.07943E-11	1.90757E-09	4930486L24Rik	RIKEN cDNA 4930486L24 gene	protein_coding	214639	-
ENSMUSG00000050350	46.38	0.101	-3.31	4.57953E-13	7.34728E-11	Gpr18	G protein-coupled receptor 18	protein_coding	110168	-
ENSMUSG00000050357	58.19	0.147	-2.765	4.86754E-11	3.94056E-09	Carmil2	capping protein regulator and myosin 1 linker 2	protein_coding	234695	-
ENSMUSG00000050390	15.84	4.12	2.043	0.000111191	0.000711597	C77080	expressed sequence C77080	protein_coding	97130	-
ENSMUSG00000050394	16.94	6.413	2.681	0.00031322	0.001640411	Armcx6	armadillo repeat containing, X-linked 6	protein_coding	278097	-
ENSMUSG00000050439	16.7	4.495	2.168	0.000339939	0.001752255	Enthd1	ENTH domain containing 1	protein_coding	383075	-
ENSMUSG00000050447	12.38	5.668	2.503	0.000767108	0.00338692	Lypd6	LY6/PLAUR domain containing 6	protein_coding	320343	-
ENSMUSG00000050511	6.86	5.709	2.513	0.004865815	0.015200597	Oprd1	opioid receptor, delta 1	protein_coding	18386	-
ENSMUSG00000050530	12.77	4.179	2.063	0.00035637	0.001817564	Fam171a1	family with sequence similarity 171, member A1	protein_coding	269233	-
ENSMUSG00000050541	13.27	4.161	2.057	0.002481699	0.008821691	Adra1b	adrenergic receptor, alpha 1b	protein_coding	11548	-
ENSMUSG00000050545	5.77	4.165	2.058	0.006666841	0.019600207	Fam228b	family with sequence similarity 228, member B	protein_coding	207921	-
ENSMUSG00000050555	18.11	0.196	-2.352	5.61681E-06	6.26495E-05	Hyls1	HYLS1, centriolar and ciliogenesis associated	protein_coding	76832	-
ENSMUSG00000050558	4.42	4.623	2.209	0.005776956	0.01750525	Prokr2	prokineticin receptor 2	protein_coding	246313	-

ENSMUSG00000050605	6.11	0.2	-2.323	0.015487603	0.038306502	Zfp61	zinc finger protein 61	protein_coding	22719	zf-C2H2
ENSMUSG00000050621	46.64	0.217	-2.206	9.74157E-08	2.18535E-06	Rps27rt	ribosomal protein S27, retrogene	protein_coding	100043813	-
ENSMUSG00000050663	28.04	4.816	2.268	7.35491E-06	7.76533E-05	Trhde	TRH-degrading enzyme	protein_coding	237553	-
ENSMUSG00000050666	9.64	4.036	2.013	0.000999643	0.004219108	Vstm4	V-set and transmembrane domain containing 4	protein_coding	320736	-
ENSMUSG00000050675	106.71	6.267	2.648	3.24038E-09	1.37078E-07	Gp1ba	glycoprotein 1b, alpha polypeptide	protein_coding	14723	-
ENSMUSG00000050751	12.2	5.956	2.574	0.00015341	0.000923492	Pgbd5	piggyBac transposable element derived 5	protein_coding	209966	-
ENSMUSG00000050761	723.41	4.544	2.184	1.23327E-05	0.000118632	Gp1bb	glycoprotein Ib, beta polypeptide	protein_coding	14724	-
ENSMUSG00000050783	31.91	4.384	2.132	3.6806E-05	0.000290839	Htr1f	5-hydroxytryptamine (serotonin) receptor 1F	protein_coding	15557	-
ENSMUSG00000050908	32	0.202	-2.311	6.99745E-08	1.66539E-06	Tvp23a	trans-golgi network vesicle protein 23A	protein_coding	383103	-
ENSMUSG00000050921	69.58	0.106	-3.239	2.88098E-16	9.58672E-14	P2ry10	purinergic receptor P2Y, G-protein coupled 10	protein_coding	78826	-
ENSMUSG00000050966	9.92	4.237	2.083	0.000902488	0.003876836	Lin28a	lin-28 homolog A (C. elegans)	protein_coding	83557	CSD
ENSMUSG00000050973	7.75	0.248	-2.014	0.004329622	0.013859201	Gdpgp1	GDP-D-glucose phosphorylase 1	protein_coding	269952	-
ENSMUSG00000050989	38.49	4.827	2.271	1.99812E-08	6.03434E-07	Selenon	selenoprotein N	protein_coding	74777	-
ENSMUSG00000050994	8.38	4.916	2.297	0.000629706	0.002890214	Adgb	androglobin	protein_coding	215772	-
ENSMUSG00000051076	4.76	4.085	2.03	0.012785751	0.032912979	Vtcn1	V-set domain containing T cell activation inhibitor 1	protein_coding	242122	-
ENSMUSG00000051079	4.62	6.096	2.608	0.002834586	0.009802669	Rgs13	regulator of G-protein signaling 13	protein_coding	246709	-
ENSMUSG00000051098	56.78	4.477	2.163	8.03829E-08	1.85894E-06	Mblac2	metallo-beta-lactamase domain containing 2	protein_coding	72852	-
ENSMUSG00000051124	52.11	0.158	-2.658	1.16571E-08	3.88621E-07	Gimap9	GTPase, IMAP family member 9	protein_coding	317758	-
ENSMUSG00000051212	55.85	0.126	-2.989	9.41028E-12	9.60757E-10	Gpr183	G protein-coupled receptor 183	protein_coding	321019	-
ENSMUSG00000051228	16.78	5.962	2.576	1.28117E-07	2.72764E-06	Nyx	nyctalopin	protein_coding	236690	-
ENSMUSG00000051232	7.97	0.134	-2.903	7.76471E-05	0.000531116	Tmem199	transmembrane protein 199	protein_coding	195040	-
ENSMUSG00000051255	11.3	0.194	-2.368	0.000260756	0.00141258	Gm6563	-	protein_coding	-	-
ENSMUSG00000051316	9.96	0.153	-2.704	0.000530172	0.002514959	Taf7	TATA-box binding protein associated factor 7	protein_coding	24074	-
ENSMUSG00000051319	11.52	0.142	-2.813	4.20075E-05	0.000323781	1500011K16Rik	mitoregulin	protein_coding	67885	-

ENSMUSG00000 051323	13.13	4.712	2.236	0.001500233	0.005873135	Pcdh19	protocadherin 19	protein_coding	279653	-
ENSMUSG00000 051331	77.53	5.329	2.414	1.44146E-10	9.62887E-09	Cacna1c	calcium channel, voltage-dependent, L type, alpha 1C subunit	protein_coding	12288	-
ENSMUSG00000 051346	4.66	0.117	-3.092	0.001627353	0.006275085	Spryd4	SPRY domain containing 4	protein_coding	66701	-
ENSMUSG00000 051367	5.99	10.259	3.359	7.53074E-05	0.000518467	Six1	sine oculis-related homeobox 1	protein_coding	20471	Homeobox
ENSMUSG00000 051375	6.94	5.607	2.487	0.00351562	0.011685569	Pcdh1	protocadherin 1	protein_coding	75599	-
ENSMUSG00000 051378	7.39	5.904	2.562	0.006712269	0.01970153	Kif18b	kinesin family member 18B	protein_coding	70218	-
ENSMUSG00000 051401	21.94	4.3	2.104	0.0004703	0.002281537	Kctd16	potass85aladin85izationramerisation domain containing 16	protein_coding	383348	-
ENSMUSG00000 051457	168.51	0.228	-2.136	1.03588E-10	7.32825E-09	Spn	sialophorin	protein_coding	20737	-
ENSMUSG00000 051490	4.79	9.024	3.174	0.002526321	0.008948446	Foxd4	forkhead box D4	protein_coding	14237	Fork
ENSMUSG00000 051497	6.25	4.811	2.266	0.013482955	0.034282611	Kcnj16	potassium inwardly-rectifying channel, subfamily J, member 16	protein_coding	16517	-
ENSMUSG00000 051557	14.31	0.237	-2.075	0.000388221	0.001948589	Pusl1	pseudouridylate synthase-like 1	protein_coding	433813	-
ENSMUSG00000 051606	7.51	7.424	2.892	0.000106689	0.000689332	2010001K21R ik	-	processed_transc ript	-	-
ENSMUSG00000 051615	105.59	4.456	2.156	5.54518E-12	6.1507E-10	Rap2a	RAS related protein 2a	protein_coding	76108	-
ENSMUSG00000 051616	5.48	4.69	2.23	0.005241211	0.01615703	C230029F24R ik	RIKEN cDNA C230029F24 gene	lincRNA	442837	-
ENSMUSG00000 051639	13.07	0.202	-2.308	0.000740609	0.003294875	Gm5812	-	processed_pseud ogene	-	-
ENSMUSG00000 051727	14.77	0.07	-3.836	3.90048E-07	7.00176E-06	Kctd14	potass85aladin85izationramerisation domain containing 14	protein_coding	233529	-
ENSMUSG00000 051777	12.63	4.398	2.137	0.000984938	0.004168916	Iqcj	IQ motif containing J	protein_coding	208426	-
ENSMUSG00000 051811	137.11	4.343	2.119	2.05774E-09	9.60405E-08	Cox6b2	cytochrome c oxidase subunit 6B2	protein_coding	333182	-
ENSMUSG00000 051851	116.87	6.571	2.716	1.6793E-12	2.23521E-10	Cxx1c	retrotransposon Gag like 8C	protein_coding	72865	-
ENSMUSG00000 051855	439.13	8.345	3.061	8.42037E-16	2.5645E-13	Mest	mesoderm specific transcript	protein_coding	17294	-
ENSMUSG00000 051985	9.78	8.222	3.039	4.29953E-05	0.000330164	Igfn1	immunoglobulin-like and fibronectin type III domain containing 1	protein_coding	226438	-
ENSMUSG00000 051998	51.94	0.079	-3.663	1.06831E-16	3.99926E-14	Lax1	lymphocyte transmembrane adaptor 1	protein_coding	240754	-
ENSMUSG00000 052013	125.17	0.119	-3.069	3.65217E-11	3.03823E-09	Btla	B and T lymphocyte associated	protein_coding	208154	-
ENSMUSG00000 052031	58.49	0.176	-2.507	2.65805E-06	3.38261E-05	Tagap1	T cell activation GTPase activating protein 1	protein_coding	380608	-

ENSMUSG00000 052135	15.1	4.514	2.174	0.000569761	0.002664072	Foxo6	forkhead box O6	protein_coding	329934	Fork
ENSMUSG00000 052142	116.65	0.169	-2.563	4.14745E-11	3.40299E-09	Rasal3	RAS protein activator like 3	protein_coding	320484	-
ENSMUSG00000 052192	3.36	0.019	-5.719	0.000107041	0.000690632	Gm5963	-	processed_pseud ogene	-	-
ENSMUSG00000 052217	503.64	4.57	2.192	0.000316458	0.001651269	Hbb-bh1	hemoglobin Z, beta-like embryonic chain	protein_coding	15132	-
ENSMUSG00000 052241	5.93	4.739	2.245	0.003327326	0.011178431	A930035D04 Rik	-	pseudogene	-	-
ENSMUSG00000 052248	115.89	4.804	2.264	1.35852E-08	4.39053E-07	Zeb2os	zinc finger E-box binding homeobox 2, opposite strand	antisense_RNA	433424	-
ENSMUSG00000 052270	54.45	6.802	2.766	1.2774E-08	4.21174E-07	Fpr2	formyl peptide receptor 2	protein_coding	14289	-
ENSMUSG00000 052281	27.62	0.175	-2.515	1.81806E-08	5.54865E-07	Tspan32os	-	antisense_RNA	-	-
ENSMUSG00000 052293	26.61	0.169	-2.563	3.99588E-07	7.14448E-06	Taf9	TATA-box binding protein associated factor 9	protein_coding	108143	-
ENSMUSG00000 052353	14.07	4.094	2.034	0.000469423	0.002278997	Cemip	cell migration inducing protein, hyaluronan binding	protein_coding	80982	-
ENSMUSG00000 052371	14.04	5.789	2.533	0.000747247	0.003315562	Hoxd3os1	-	antisense_RNA	-	-
ENSMUSG00000 052373	6.72	4.622	2.208	0.002392092	0.008572698	Mpp3	membrane protein, palmitoylated 3 (MAGUK p55 subfamily member 3)	protein_coding	13384	-
ENSMUSG00000 052415	7.27	5.319	2.411	0.006185682	0.018491186	Tchh	trichohyalin	protein_coding	99681	-
ENSMUSG00000 052419	16.75	0.174	-2.521	1.14254E-05	0.000111699	2610001J05Ri k	RIKEN cDNA 2610001J05 gene	protein_coding	66520	-
ENSMUSG00000 052479	17.07	4.128	2.045	0.000341407	0.001756877	A330008L17 Rik	RIKEN cDNA A330008L17 gene	lincRNA	234624	-
ENSMUSG00000 052504	21.69	4.377	2.13	1.82542E-06	2.49611E-05	Epha3	Eph receptor A3	protein_coding	13837	-
ENSMUSG00000 052516	41.17	4.173	2.061	4.12675E-07	7.34195E-06	Robo2	roundabout guidance receptor 2	protein_coding	268902	-
ENSMUSG00000 052688	5.15	4.447	2.153	0.014084657	0.03542654	Rab7b	RAB7B, member RAS oncogene family	protein_coding	226421	-
ENSMUSG00000 052727	16.96	4.358	2.124	0.002016907	0.007466379	Map1b	microtubule-associated protein 1B	protein_coding	17755	-
ENSMUSG00000 052760	71.13	0.222	-2.171	2.4949E-08	7.16147E-07	A630001G21 Rik	RIKEN cDNA A630001G21 gene	protein_coding	319997	-
ENSMUSG00000 052949	169.79	5.021	2.328	0.000835502	0.00364308	Rnf157	ring finger protein 157	protein_coding	217340	-
ENSMUSG00000 052955	8.28	6.162	2.623	0.000529249	0.002512652	Cpvl	carboxypeptidase, vitellogenic-like	protein_coding	71287	-
ENSMUSG00000 053007	280.75	6.881	2.783	1.1696E-12	1.66798E-10	Creb5	cAMP responsive element binding protein 5	protein_coding	231991	TF_bZIP
ENSMUSG00000 053024	7.16	7.419	2.891	0.002878017	0.009931838	Cntn2	contactin 2	protein_coding	21367	-

ENSMUSG00000053025	18.49	5.084	2.346	2.28056E-05	0.000197111	Sv2b	synaptic vesicle glycoprotein 2 b	protein_coding	64176	-
ENSMUSG00000053040	6.41	5.446	2.445	0.004822485	0.015086216	Aph1c	aph1 homolog C, gamma secretase subunit	protein_coding	68318	-
ENSMUSG00000053044	87.21	0.074	-3.753	1.26046E-16	4.6223E-14	Cd8b1	CD8 antigen, beta chain 1	protein_coding	12526	-
ENSMUSG00000053046	13.71	5.016	2.327	3.05237E-05	0.000249423	Brsk2	BR serine/threonine kinase 2	protein_coding	75770	-
ENSMUSG00000053093	7.52	6.988	2.805	0.001641107	0.00632115	Myh7	myosin, heavy polypeptide 7, cardiac muscle, beta	protein_coding	140781	-
ENSMUSG00000053111	9.19	5.542	2.47	0.001604731	0.006207839	Fank1	fibronectin type 3 and ankyrin repeat domains 1	protein_coding	66930	-
ENSMUSG00000053137	21.41	0.218	-2.194	1.79658E-05	0.000162798	Mapk11	mitogen-activated protein kinase 11	protein_coding	19094	-
ENSMUSG00000053161	6.38	4.039	2.014	0.003419678	0.011413481	Daw1	dynein assembly factor with WDR repeat domains 1	protein_coding	71227	-
ENSMUSG00000053173	30.75	0.226	-2.146	1.69507E-06	2.35021E-05	Rpl18-ps2	-	processed_pseudogene	-	-
ENSMUSG00000053182	6.72	10.472	3.389	1.21706E-05	0.000117475	Gm609	predicted gene 609	protein_coding	208166	-
ENSMUSG00000053310	8442.69	5.911	2.563	8.54866E-07	1.32881E-05	Nrgn	neurogranin	protein_coding	64011	-
ENSMUSG00000053329	20.88	0.199	-2.326	0.000108292	0.000696704	D10Jhu81e	DNA segment, Chr 10, Johns Hopkins University 81 expressed	protein_coding	28295	-
ENSMUSG00000053414	23.44	4.325	2.113	4.273E-06	5.01651E-05	Hunk	hormonally upregulated Neu-associated kinase	protein_coding	26559	-
ENSMUSG00000053550	8.18	8.355	3.063	0.000309862	0.001626141	Shisa7	shisa family member 7	protein_coding	232813	-
ENSMUSG00000053580	153.37	12.021	3.588	6.49856E-05	0.000459554	Tanc2	tetratricopeptide repeat, ankyrin repeat and coiled-coil containing 2	protein_coding	77097	-
ENSMUSG00000053801	11.95	0.044	-4.491	1.80312E-08	5.52905E-07	Grwd1	glutamate-rich WD repeat containing 1	protein_coding	101612	-
ENSMUSG00000053841	34.23	0.114	-3.139	2.07669E-10	1.30476E-08	Txlna	taxilin alpha	protein_coding	109658	-
ENSMUSG00000053846	7.82	8.645	3.112	0.000522003	0.002483418	Lipg	lipase, endothelial	protein_coding	16891	-
ENSMUSG00000053852	8.05	4.651	2.218	0.006612897	0.019479861	Adgrg4	adhesion G protein-coupled receptor G4	protein_coding	236798	-
ENSMUSG00000053963	7.25	4.001	2	0.014179462	0.035595243	Stum	mechanosensory transduction mediator	protein_coding	381310	-
ENSMUSG00000053965	37.07	6.334	2.663	8.64538E-11	6.43504E-09	Pde5a	phosphodiesterase 5A, cGMP-specific	protein_coding	242202	-
ENSMUSG00000053977	80.42	0.112	-3.153	9.20148E-14	1.85777E-11	Cd8a	CD8 antigen, alpha chain	protein_coding	12525	-
ENSMUSG00000054013	7.13	5.451	2.447	0.015530016	0.038395549	Tmem179	transmembrane protein 179	protein_coding	104885	-
ENSMUSG00000054061	5.21	5.147	2.364	0.005175715	0.015993536	Gm9934	-	antisense_RNA	-	-

ENSMUSG00000 054065	37.07	0.173	-2.528	2.99856E-08	8.30219E-07	Pkp3	plakophilin 3	protein_coding	56460	-
ENSMUSG00000 054146	4.93	6.517	2.704	0.007037485	0.020456324	Krt15	keratin 15	protein_coding	16665	-
ENSMUSG00000 054252	3.21	5.736	2.52	0.012159976	0.031666077	Fgfr3	fibroblast growth factor receptor 3	protein_coding	14184	-
ENSMUSG00000 054256	7.73	4.256	2.09	0.001360688	0.005427349	Msi1	musashi RNA-binding protein 1	protein_coding	17690	-
ENSMUSG00000 054293	16.1	0.145	-2.787	2.37111E-07	4.61609E-06	A630033H20 Rik	purinergic receptor P2Y, G-protein coupled 10B	protein_coding	213438	-
ENSMUSG00000 054310	8.97	6.715	2.747	0.000145674	0.000886727	Obox1	oocyte specific homeobox 1	protein_coding	71468	Homeobox
ENSMUSG00000 054320	7.71	4.432	2.148	0.005729576	0.017382933	Lrrc36	leucine rich repeat containing 36	protein_coding	270091	-
ENSMUSG00000 054435	217.5	0.187	-2.423	3.46282E-11	2.90763E-09	Gimap4	GTPase, IMAP family member 4	protein_coding	107526	-
ENSMUSG00000 054514	6.01	5.27	2.398	0.000763143	0.003375027	Atad3aos	ATPase family, AAA domain containing 3A, opposite strand	antisense_RNA	70448	-
ENSMUSG00000 054519	4.43	0.122	-3.04	0.001112103	0.004604464	Zfp867	zinc finger protein 867	protein_coding	237775	zf-C2H2
ENSMUSG00000 054555	29.67	4.738	2.244	2.6267E-06	3.34983E-05	Adam12	a disintegrin and metallopeptidase domain 12 (meltrin alpha)	protein_coding	11489	-
ENSMUSG00000 054641	127.96	5.17	2.37	9.55411E-11	6.92249E-09	Mmrn1	multimerin 1	protein_coding	70945	-
ENSMUSG00000 054659	14.3	4.03	2.011	0.007597152	0.021768971	Pm20d2	peptidase M20 domain containing 2	protein_coding	242377	-
ENSMUSG00000 054715	8.75	0.233	-2.102	0.00725202	0.020950411	Zscan22	zinc finger and SCAN domain containing 22	protein_coding	232878	zf-C2H2
ENSMUSG00000 054746	8.91	8.983	3.167	0.000174424	0.00102059	Abca15	ATP-binding cassette, sub-family A (ABC1), member 15	protein_coding	320631	-
ENSMUSG00000 054752	9.78	7.471	2.901	0.001839516	0.006926711	Fsd1l	fibronectin type III and SPRY domain containing 1-like	protein_coding	319636	-
ENSMUSG00000 054793	9.05	15.583	3.962	1.06334E-07	2.356E-06	Cadm4	cell adhesion molecule 4	protein_coding	260299	-
ENSMUSG00000 054863	16.04	5.45	2.446	3.52809E-06	4.28932E-05	Fam19a5	family with sequence similarity 19, member A5	protein_coding	106014	-
ENSMUSG00000 054871	74.66	7.569	2.92	2.7487E-09	1.21954E-07	Tmem158	transmembrane protein 158	protein_coding	72309	-
ENSMUSG00000 054976	19	5.06	2.339	7.62502E-06	8.0219E-05	Nyap2	neuronal tyrosine-phophorylated phosphoinositide 3-kinase adaptor 2	protein_coding	241134	-
ENSMUSG00000 055015	6.69	9.322	3.221	5.36102E-05	0.000394158	Gm9961	predicted gene 9961	protein_coding	791359	-
ENSMUSG00000 055041	8.5	0.198	-2.336	0.00172844	0.006585739	Commd5	COMM domain containing 5	protein_coding	66398	-
ENSMUSG00000 055044	320.71	4.1	2.035	4.77649E-08	1.20886E-06	Pdlim1	PDZ and LIM domain 1 (elfin)	protein_coding	54132	-
ENSMUSG00000 055078	9.72	4.512	2.174	0.000558231	0.002617657	Gabra5	gamma-aminobutyric acid (GABA) A receptor, subunit alpha 5	protein_coding	110886	-

ENSMUSG00000 055093	78.83	0.234	-2.093	2.98995E-08	8.29112E-07	Gm8430	-	processed_pseud ogene	-	-
ENSMUSG00000 055134	6.66	6.607	2.724	0.000780679	0.003439929	9130017K11R ik	-	processed_transc ript	-	-
ENSMUSG00000 055320	32.55	4.196	2.069	5.7194E-06	6.33959E-05	Tead1	TEA domain family member 1	protein_coding	21676	TEA
ENSMUSG00000 055333	11.81	5.885	2.557	0.004868505	0.015206356	Fat2	FAT atypical cadherin 2	protein_coding	245827	-
ENSMUSG00000 055373	17.61	5.271	2.398	1.88305E-05	0.000169267	Fut9	fucosyltransferase 9	protein_coding	14348	-
ENSMUSG00000 055403	15.38	4.181	2.064	4.42916E-05	0.000338526	4933427D06R ik	RIKEN cDNA 4933427D06 gene	lincRNA	232217	-
ENSMUSG00000 055415	9.77	5.393	2.431	0.000145074	0.000884873	Atp10b	ATPase, class V, type 10B	protein_coding	319767	-
ENSMUSG00000 055471	69.21	4.652	2.218	3.88699E-08	1.03322E-06	Alk	anaplastic lymphoma kinase	protein_coding	11682	-
ENSMUSG00000 055485	18.08	4.739	2.244	7.05301E-05	0.000492751	Soga1	suppressor of glucose, autophagy associated 1	protein_coding	320706	-
ENSMUSG00000 055489	7.35	5.808	2.538	0.000265086	0.001430375	Ano5	anoctamin 5	protein_coding	233246	-
ENSMUSG00000 055540	75.88	4.736	2.244	1.81877E-08	5.54865E-07	Epha6	Eph receptor A6	protein_coding	13840	-
ENSMUSG00000 055723	57.07	0.235	-2.089	1.08336E-07	2.39445E-06	Rras2	related RAS viral (r-ras) oncogene 2	protein_coding	66922	-
ENSMUSG00000 055775	9.17	6.9	2.787	0.000211123	0.00119373	Myh8	myosin, heavy polypeptide 8, skeletal muscle, perinatal	protein_coding	17885	-
ENSMUSG00000 055799	23.44	4.188	2.066	6.88848E-05	0.000482382	Tcf7l1	transcription factor 7 like 1 (T cell specific, HMG box)	protein_coding	21415	HMG
ENSMUSG00000 055809	6.76	4.716	2.238	0.001390237	0.005515823	Dnaaf3	dynein, axonemal assembly factor 3	protein_coding	436022	-
ENSMUSG00000 055912	4.39	6.133	2.617	0.01519611	0.037715317	Tmem150a	transmembrane protein 150A	protein_coding	232086	-
ENSMUSG00000 055963	20.64	6.728	2.75	2.74292E-07	5.21562E-06	Triqk	triple QxxK/R motif containing	protein_coding	208820	-
ENSMUSG00000 055980	14	5.147	2.364	1.30741E-05	0.000124763	Irs1	insulin receptor substrate 1	protein_coding	16367	-
ENSMUSG00000 056025	10.92	6.777	2.761	0.000269698	0.001449224	Clca3a1	chloride channel accessory 3A1	protein_coding	12722	-
ENSMUSG00000 056031	8.91	4.435	2.149	0.000713186	0.00319662	9330154J02Ri k	-	processed_transc ript	-	-
ENSMUSG00000 056073	58.36	4.758	2.25	7.5584E-09	2.74378E-07	Grik2	glutamate receptor, 89aladinpic, kainate 2 (beta 2)	protein_coding	14806	-
ENSMUSG00000 056076	59.51	0.221	-2.181	6.75642E-06	7.2438E-05	Eif3b	eukaryotic translation initiation factor 3, subunit B	protein_coding	27979	-
ENSMUSG00000 056155	4.34	7.755	2.955	0.001010059	0.004255509	Nanos3	nanos C2HC-type zinc finger 3	protein_coding	244551	-
ENSMUSG00000 056209	52.24	0.228	-2.131	1.10906E-08	3.73196E-07	Npm3	nucleoplasmin 3	protein_coding	18150	-

ENSMUSG00000056215	8.48	5.091	2.348	0.000316294	0.001651219	Lrguk	leucine-rich repeats and guanylate kinase domain containing	protein_coding	74354	-
ENSMUSG00000056222	57.89	4.5	2.17	8.40065E-09	2.97734E-07	Spock1	sparc/osteonectin, cwcv and kazal-like domains proteoglycan 1	protein_coding	20745	-
ENSMUSG00000056290	230.73	0.243	-2.041	1.28847E-07	2.73995E-06	Ms4a4b	membrane-spanning 4-domains, subfamily A, member 4B	protein_coding	60361	-
ENSMUSG00000056383	12.66	0.124	-3.006	1.74252E-06	2.41042E-05	AI987944	expressed sequence AI987944	protein_coding	233168	zf-C2H2
ENSMUSG00000056418	13.94	0.037	-4.746	6.7819E-09	2.51785E-07	BC043934	-	lincRNA	-	-
ENSMUSG00000056459	17.46	0.243	-2.04	0.000246457	0.001351415	Zbtb25	zinc finger and BTB domain containing 25	protein_coding	109929	ZBTB
ENSMUSG00000056476	1032.43	5.208	2.381	9.1592E-10	4.74299E-08	Med12l	mediator complex subunit 12-like	protein_coding	329650	-
ENSMUSG00000056492	14.05	6.88	2.783	4.31124E-06	5.04682E-05	Adgrf5	adhesion G protein-coupled receptor F5	protein_coding	224792	-
ENSMUSG00000056531	13.66	4.169	2.06	0.003820289	0.012504766	Ccdc18	coiled-coil domain containing 18	protein_coding	73254	-
ENSMUSG00000056612	29.29	0.213	-2.23	2.34319E-07	4.58158E-06	Ppp1r14b	protein phosphatase 1, regulatory inhibitor subunit 14B	protein_coding	18938	-
ENSMUSG00000056629	14.07	0.234	-2.095	0.000636072	0.002910512	Fkbp2	FK506 binding protein 2	protein_coding	14227	-
ENSMUSG00000056665	22.69	0.074	-3.749	5.01437E-11	4.03895E-09	Them6	thioesterase superfamily member 6	protein_coding	223626	-
ENSMUSG00000056735	15.3	0.229	-2.129	0.002641983	0.009266796	A930024E05Rik	RIKEN cDNA A930024E05 gene	antisense_RNA	109202	-
ENSMUSG00000056752	34.36	5.416	2.437	1.93216E-08	5.85048E-07	Dnah9	dynein, axonemal, heavy chain 9	protein_coding	237806	-
ENSMUSG00000056758	149.32	4.585	2.197	2.58517E-09	1.16132E-07	Hmga2	high mobility group AT-hook 2	protein_coding	15364	HMGI/HMGY
ENSMUSG00000056820	52.68	0.175	-2.517	1.08306E-06	1.61641E-05	Tsnax	translin-associated factor X	protein_coding	53424	-
ENSMUSG00000056856	16.13	6.456	2.691	2.11744E-06	2.80592E-05	Jakmip3	-	protein_coding	-	-
ENSMUSG00000056899	327.05	4.126	2.045	1.51461E-07	3.16097E-06	Immp2l	IMP2 inner mitochondrial membrane peptidase-like (S. cerevisiae)	protein_coding	93757	-
ENSMUSG00000056900	19.73	5.714	2.515	3.0774E-07	5.73628E-06	Usp13	ubiquitin specific peptidase 13 (isopeptidase T-3)	protein_coding	72607	-
ENSMUSG00000057003	6.25	5.045	2.335	0.003197804	0.010829502	Myh4	myosin, heavy polypeptide 4, skeletal muscle	protein_coding	17884	-
ENSMUSG00000057103	5.12	0.233	-2.102	0.016439261	0.040195549	Nat8f1	N-acetyltransferase 8 (GCN5-related) family member 1	protein_coding	66116	-
ENSMUSG00000057113	173.81	0.106	-3.239	9.19024E-20	4.71827E-17	Npm1	nucleophosmin 1	protein_coding	18148	-
ENSMUSG00000057137	489.39	4.161	2.057	2.79414E-06	3.5308E-05	Tmem140	transmembrane protein 140	protein_coding	68487	-
ENSMUSG00000057182	11.09	5.478	2.454	2.59078E-05	0.000219076	Scn3a	sodium channel, voltage-gated, type III, alpha	protein_coding	20269	-

ENSMUSG00000057229	29.91	0.201	-2.315	4.85331E-07	8.40165E-06	Atp5sl	distal membrane arm assembly complex 2	protein_coding	66349	-
ENSMUSG00000057262	206.2	0.228	-2.136	8.7023E-11	6.43504E-09	Gm10020	-	protein_coding	-	-
ENSMUSG00000057278	60.12	0.173	-2.528	5.71729E-12	6.26427E-10	Snrpg	small nuclear ribonucleoprotein polypeptide G	protein_coding	68011	-
ENSMUSG00000057337	121.26	0.061	-4.038	7.53942E-23	5.64482E-20	Chst3	carbohydrate sulfotransferase 3	protein_coding	53374	-
ENSMUSG00000057411	23.51	0.218	-2.198	2.64768E-05	0.000223153	Fam173a	family with sequence similarity 173, member A	protein_coding	214917	-
ENSMUSG00000057497	8.48	0.198	-2.334	0.00337868	0.011314107	Fam136a	family with sequence similarity 136, member A	protein_coding	66488	-
ENSMUSG00000057604	8.83	5.046	2.335	0.005876255	0.017740327	Lmcd1	LIM and cysteine-rich domains 1	protein_coding	30937	-
ENSMUSG00000057605	133.39	0.217	-2.201	6.57925E-12	7.07919E-10	Gm6807	-	processed_pseudogene	-	-
ENSMUSG00000057657	95.14	0.11	-3.191	1.58406E-16	5.69281E-14	Rps18-ps3	-	processed_pseudogene	-	-
ENSMUSG00000057719	9.17	9.127	3.19	9.48669E-06	9.54459E-05	Sh3rf2	SH3 domain containing ring finger 2	protein_coding	269016	-
ENSMUSG00000057880	19.75	6.076	2.603	3.3178E-06	4.06754E-05	Abat	4-aminobutyrate aminotransferase	protein_coding	268860	-
ENSMUSG00000057894	19.36	0.245	-2.028	0.000298025	0.001573212	Zfp329	zinc finger protein 329	protein_coding	67230	zf-C2H2
ENSMUSG00000057982	29.83	0.23	-2.122	8.81397E-06	9.0347E-05	Zfp809	zinc finger protein 809	protein_coding	235047	zf-C2H2
ENSMUSG00000058022	11.35	8.382	3.067	7.2107E-05	0.00050046	Adtrp	androgen dependent TFPI regulating protein	protein_coding	109254	-
ENSMUSG00000058056	53.63	5.175	2.372	8.59546E-10	4.463991aladinlld	palladin, cytoskeletal associated protein	protein_coding	72333	-	
ENSMUSG00000058064	141.55	0.166	-2.59	1.59925E-05	0.000147606	Gm10036	-	protein_coding	-	-
ENSMUSG00000058070	28.45	5.416	2.437	3.22133E-10	1.87221E-08	Eml1	echinoderm microtubule associated protein like 1	protein_coding	68519	-
ENSMUSG00000058152	23.98	5.484	2.455	3.75391E-06	4.51499E-05	Chsy3	chondroitin sulfate synthase 3	protein_coding	78923	-
ENSMUSG00000058153	20.23	4.253	2.088	0.000214213	0.001206355	Sez6l	seizure related 6 homolog like	protein_coding	56747	-
ENSMUSG00000058258	5.46	0.011	-6.443	1.69837E-06	2.35296E-05	Idi1	isopentenyl-diphosphate delta isomerase	protein_coding	319554	-
ENSMUSG00000058267	15.29	0.207	-2.271	8.79851E-05	0.000587953	Mrps14	mitochondrial ribosomal protein S14	protein_coding	64659	-
ENSMUSG00000058420	9.76	5.193	2.377	0.000482957	0.002332235	Syt17	synaptotagmin XVII	protein_coding	110058	-
ENSMUSG00000058441	5.86	4.958	2.31	0.016276986	0.039863862	Panx2	pannexin 2	protein_coding	406218	-
ENSMUSG00000058470	68.19	0.138	-2.856	1.37188E-12	1.88178E-10	Gm8369	predicted gene 8369	protein_coding	666926	-

ENSMUSG00000058488	8.6	8.677	3.117	0.000172756	0.001012474	Kl	klotho	protein_coding	16591	-
ENSMUSG00000058558	1843.02	0.233	-2.102	2.12173E-06	2.80953E-05	Rpl5	ribosomal protein L5	protein_coding	100503670	-
ENSMUSG00000058600	523.09	0.243	-2.043	3.6257E-09	1.50115E-07	Rpl30	ribosomal protein L30	protein_coding	19946	-
ENSMUSG00000058638	29.32	0.149	-2.749	5.45256E-10	3.02398E-08	Zfp110	zinc finger protein 110	protein_coding	65020	zf-C2H2
ENSMUSG00000058922	13.64	0.157	-2.669	3.57712E-05	0.000285423	Gm10052	-	processed_pseudogene	-	-
ENSMUSG00000058952	5.12	6.438	2.687	0.010567816	0.028388859	Cfi	complement component factor i	protein_coding	12630	-
ENSMUSG00000059005	92.55	0.222	-2.171	4.91675E-10	2.75231E-08	Hnrnpa3	heterogeneous nuclear ribonucleoprotein A3	protein_coding	229279	-
ENSMUSG00000059049	22.19	4.209	2.074	3.2287E-05	0.000261926	Frem1	Fras1 related extracellular matrix protein 1	protein_coding	329872	-
ENSMUSG00000059070	915.54	0.202	-2.31	3.22992E-10	1.87221E-08	Rpl18	ribosomal protein L18	protein_coding	19899	-
ENSMUSG00000059327	25.29	4.184	2.065	5.62214E-06	6.26701E-05	Eda	ectodysplasin-A	protein_coding	13607	-
ENSMUSG00000059493	26.51	4.26	2.091	2.16542E-06	2.85896E-05	Nhs	NHS actin remodeling regulator	protein_coding	195727	-
ENSMUSG00000059602	46.65	4.225	2.079	8.95136E-06	9.13903E-05	Syn3	synapsin III	protein_coding	27204	-
ENSMUSG00000059657	54.27	8.348	3.061	7.26185E-07	1.16403E-05	Stfa2l1	stefin A2	protein_coding	20862	-
ENSMUSG00000059743	14.07	0.247	-2.016	0.000459238	0.002242402	Fdps	farnesyl diphosphate synthetase	protein_coding	110196	-
ENSMUSG00000059775	93.08	0.247	-2.017	0.001205873	0.004914569	Rps26-ps1	-	transcribed_processed_pseudogene	-	-
ENSMUSG00000059852	20.91	4.091	2.032	2.38815E-05	0.000204931	Kcng2	potassium voltage-gated channel, subfamily G, member 2	protein_coding	240444	-
ENSMUSG00000059854	38.89	4.969	2.313	2.59691E-05	0.000219491	Hydin	HYDIN, axonemal central pair apparatus protein	protein_coding	244653	-
ENSMUSG00000059900	671.46	4.92	2.299	6.1991E-08	1.50124E-06	Tmem40	transmembrane protein 40	protein_coding	94346	-
ENSMUSG00000059921	34.77	4.006	2.002	1.28846E-05	0.000123282	Unc5c	unc-5 netrin receptor C	protein_coding	22253	-
ENSMUSG00000059994	89.67	0.076	-3.712	5.20027E-17	1.98816E-14	Fcrl1	Fc receptor-like 1	protein_coding	229499	-
ENSMUSG00000060093	31.23	5.241	2.39	7.747E-06	8.11696E-05	Hist1h4a	histone cluster 1, H4a	protein_coding	326619	-
ENSMUSG00000060180	10.23	5.299	2.406	9.14019E-05	0.000605829	Myh13	myosin, heavy polypeptide 13, skeletal muscle	protein_coding	544791	-
ENSMUSG00000060198	18.47	0.126	-2.99	2.43952E-08	7.02496E-07	Gm11353	-	processed_pseudogene	-	-
ENSMUSG00000060206	25.65	9.068	3.181	6.17099E-08	1.49644E-06	Zfp462	zinc finger protein 462	protein_coding	242466	-

ENSMUSG00000 060257	7.84	7.49	2.905	0.000239468	0.001319129	Scrt2	scratch family zinc finger 2	protein_coding	545474	zf-C2H2
ENSMUSG00000 060261	127.84	0.23	-2.122	1.02476E-08	3.54115E-07	Gtf2i	general transcription factor II I	protein_coding	14886	GTF2I
ENSMUSG00000 060377	12.94	0.122	-3.036	5.57562E-05	0.000406609	Rpl36a-ps1	-	processed_pseud ogene	-	-
ENSMUSG00000 060424	7.99	4.74	2.245	0.009415303	0.025833498	Pantr1	POU domain, class 3, transcription factor 3 adjacent noncoding transcript 1	lincRNA	66297	-
ENSMUSG00000 060438	31.81	0.149	-2.746	3.00595E-07	5.65591E-06	Rps10-ps1	-	processed_pseud ogene	-	-
ENSMUSG00000 060445	13.91	4.347	2.12	0.000144072	0.000879358	Sycp2	synaptonemal complex protein 2	protein_coding	320558	-
ENSMUSG00000 060509	17.33	4.921	2.299	0.000232539	0.001290854	Xcr1	chemokine (C motif) receptor 1	protein_coding	23832	-
ENSMUSG00000 060586	527.99	0.023	-5.473	5.85179E-23	4.57177E-20	H2-Eb1	histocompatibility 2, class II antigen E beta	protein_coding	14969	-
ENSMUSG00000 060678	65.93	5.989	2.582	1.29656E-10	8.82492E-09	Hist1h4c	histone cluster 1, H4c	protein_coding	319155	-
ENSMUSG00000 060730	6.04	0.188	-2.411	0.002572003	0.009069138	Gm10086	-	processed_pseud ogene	-	-
ENSMUSG00000 060739	158.01	0.182	-2.461	3.42176E-12	3.99257E-10	Nsa2	NSA2 ribosome biogenesis homolog	protein_coding	59050	-
ENSMUSG00000 060798	10.99	6.094	2.607	2.60525E-05	0.000219922	Intu	inturned planar cell polarity protein	protein_coding	380614	-
ENSMUSG00000 060882	40.09	4.328	2.114	2.9857E-06	3.73031E-05	Kcnd2	potassium voltage-gated channel, Shal-related family, member 2	protein_coding	16508	-
ENSMUSG00000 060924	122.98	3.999	2	8.11711E-09	2.89398E-07	Csmd1	CUB and Sushi multiple domains 1	protein_coding	94109	-
ENSMUSG00000 060950	10.4	0.131	-2.935	2.22284E-05	0.000193144	Trmt61a	tRNA methyltransferase 61A	protein_coding	328162	-
ENSMUSG00000 060985	7.2	4.776	2.256	0.008265763	0.02326923	Tdrd5	tudor domain containing 5	protein_coding	214575	-
ENSMUSG00000 061032	70.5	0.187	-2.42	2.99821E-11	2.55331E-09	Rrp1	ribosomal RNA processing 1 homolog (S. cerevisiae)	protein_coding	18114	-
ENSMUSG00000 061082	13.02	5.221	2.384	0.00130678	0.005250788	Plac1	placental specific protein 1	protein_coding	56096	-
ENSMUSG00000 061132	59.22	0.144	-2.797	1.89107E-13	3.39806E-11	Blnk	B cell linker	protein_coding	17060	-
ENSMUSG00000 061143	68.24	4.119	2.042	1.46154E-09	7.17553E-08	Maml3	mastermind like transcriptional coactivator 3	protein_coding	433586	-
ENSMUSG00000 061167	275.18	0.202	-2.31	2.24801E-12	2.80517E-10	Rpl15-ps3	-	processed_pseud ogene	-	-
ENSMUSG00000 061171	6.2	5.189	2.375	0.010090019	0.027297132	Slc38a11	solute carrier family 38, member 11	protein_coding	320106	-
ENSMUSG00000 061315	305.7	0.175	-2.515	2.42687E-11	2.1482E-09	Naca	nascent polypeptide-associated complex alpha polypeptide	protein_coding	17938	-
ENSMUSG00000 061330	37.38	0.052	-4.264	2.17046E-15	6.00016E-13	Gm11361	-	processed_pseud ogene	-	-

ENSMUSG00000061393	8.84	5.144	2.363	0.000156699	0.00093795	Acvr2b	activin receptor IIB	protein_coding	11481	-
ENSMUSG00000061410	16.86	6.204	2.633	3.92351E-07	7.03609E-06	Zcchc14	zinc finger, CCHC domain containing 14	protein_coding	142682	-
ENSMUSG00000061411	54.11	5.792	2.534	2.09694E-09	9.7364E-08	Nol4l	nucleolar protein 4-like	protein_coding	329540	-
ENSMUSG00000061535	8.73	4.513	2.174	0.000943674	0.004020122	C1qtnf7	C1q and tumor necrosis factor related protein 7	protein_coding	109323	-
ENSMUSG00000061576	71.47	4.208	2.073	1.1798E-07	2.56036E-06	Dpp6	dipeptidylpeptidase 6	protein_coding	13483	-
ENSMUSG00000061577	15.42	0.149	-2.751	6.40586E-06	6.9509E-05	Adgrg5	adhesion G protein-coupled receptor G5	protein_coding	382045	-
ENSMUSG00000061778	12.48	0.179	-2.485	4.90617E-05	0.000367482	Mospd2	motile sperm domain containing 2	protein_coding	76763	-
ENSMUSG00000061815	10.85	4.114	2.041	0.004266888	0.013696268	Rufy4	RUN and FYVE domain containing 4	protein_coding	435626	-
ENSMUSG00000061878	8.57	5.857	2.55	0.006248343	0.018641287	Sphk1	sphingosine kinase 1	protein_coding	20698	-
ENSMUSG00000061911	28.75	4.771	2.254	1.48804E-05	0.000139046	Myt1l	myelin transcription factor 1-like	protein_coding	17933	zf-C2HC
ENSMUSG00000061928	6.63	107.53	6.749	1.79755E-10	1.16187E-08	Dsg1b	desmoglein 1 beta	protein_coding	225256	-
ENSMUSG00000062014	53.78	0.19	-2.398	8.28714E-11	6.25679E-09	Gmfb	glia maturation factor, beta	protein_coding	63985	-
ENSMUSG00000062038	5.49	0.198	-2.338	0.011629719	0.030583115	Gm10108	-	processed_pseudogene	-	-
ENSMUSG00000062044	5.52	8.908	3.155	0.000496416	0.002379343	Lmtk3	lemur tyrosine kinase 3	protein_coding	381983	-
ENSMUSG00000062098	31.23	5.163	2.368	9.15818E-06	9.29355E-05	Btbd3	BTB (POZ) domain containing 3	protein_coding	228662	-
ENSMUSG00000062151	36.17	5.156	2.366	4.99746E-05	0.000372302	Unc13c	unc-13 homolog C	protein_coding	208898	-
ENSMUSG00000062184	28.99	5.486	2.456	4.82255E-06	5.51952E-05	Hs6st2	heparan sulfate 6-O-sulfotransferase 2	protein_coding	50786	-
ENSMUSG00000062372	11.1	4.085	2.03	0.001606204	0.0062122	Otof	otoferlin	protein_coding	83762	-
ENSMUSG00000062391	4.79	7.096	2.827	0.01263793	0.032608113	4932435O22Rik	RIKEN cDNA 4932435O22 gene	lincRNA	319819	-
ENSMUSG00000062393	9.86	7.146	2.837	0.0004555	0.002229607	Dgkk	diacylglycerol kinase kappa	protein_coding	331374	-
ENSMUSG00000062456	21.37	0.246	-2.02	0.000155783	0.000933399	Rpl9-ps6	-	protein_coding	-	-
ENSMUSG00000062458	55.26	0.219	-2.191	2.07225E-08	6.20606E-07	Gm8623	predicted gene 8623	processed_pseudogene	667422	-
ENSMUSG00000062545	4.9	0.11	-3.178	0.000885246	0.003817372	Tlr12	toll-like receptor 12	protein_coding	384059	-
ENSMUSG00000062554	17.92	0.213	-2.229	0.000127433	0.000794258	Gm12751	-	processed_pseudogene	-	-

ENSMUSG00000062580	16.99	0.186	-2.427	1.12447E-05	0.000110293	Timm17a	translocase of inner mitochondrial membrane 17a	protein_coding	21854	-
ENSMUSG00000062582	7.66	0.224	-2.158	0.006663898	0.019596863	Rpl30-ps8	-	processed_pseudogene	-	-
ENSMUSG00000062683	91.25	0.198	-2.336	1.50329E-10	9.9311E-09	Atp5g2	ATP synthase, H+ transporting, mitochondrial F0 complex, subunit C2 (subunit 9)	protein_coding	67942	-
ENSMUSG00000062713	6.97	5.482	2.455	0.001482102	0.005815317	Sim2	single-minded family bHLH transcription factor 2	protein_coding	20465	-
ENSMUSG00000062727	254.63	4.952	2.308	2.84467E-12	3.40773E-10	Hist1h2bk	histone cluster 1, H2bk	protein_coding	319184	-
ENSMUSG00000062760	9.8	4.338	2.117	0.001127188	0.004657265	1810041L15Rik	shisa like 1	protein_coding	72301	-
ENSMUSG00000062866	40.17	4.089	2.032	7.53483E-07	1.19711E-05	Phactr2	phosphatase and actin regulator 2	protein_coding	215789	-
ENSMUSG00000062867	45.53	0.12	-3.063	2.53088E-12	3.13637E-10	Impdh2	inosine monophosphate dehydrogenase 2	protein_coding	23918	-
ENSMUSG00000062906	26.45	0.185	-2.433	6.49085E-06	7.00356E-05	Hdac10	histone deacetylase 10	protein_coding	170787	-
ENSMUSG00000062960	10.6	12.797	3.678	6.32364E-05	0.000448951	Kdr	kinase insert domain protein receptor	protein_coding	16542	-
ENSMUSG00000062961	5.9	6.695	2.743	0.002330368	0.008401763	Ccdc177	coiled-coil domain containing 177	protein_coding	380768	-
ENSMUSG00000062995	16.59	5.221	2.384	0.000138995	0.000853006	Ica1	islet cell autoantigen 1	protein_coding	15893	-
ENSMUSG00000063239	21.01	5.932	2.568	5.79217E-06	6.4049E-05	Grm4	glutamate receptor, metabotropic 4	protein_coding	268934	-
ENSMUSG00000063260	8.28	4.378	2.13	0.004153935	0.013391114	Syt10	synaptotagmin X	protein_coding	54526	-
ENSMUSG00000063275	31.1	4.414	2.142	1.14262E-07	2.4887E-06	Hacd1	3-hydroxyacyl-CoA dehydratase 1	protein_coding	30963	-
ENSMUSG00000063296	40.02	7.121	2.832	1.98981E-09	9.43401E-08	Tmem117	transmembrane protein 117	protein_coding	320709	-
ENSMUSG00000063297	24.66	4.284	2.099	2.95771E-05	0.000243459	Luzp2	leucine zipper protein 2	protein_coding	233271	-
ENSMUSG00000063354	53.32	4.076	2.027	5.35994E-08	1.33582E-06	Slc39a4	solute carrier family 39 (zinc transporter), member 4	protein_coding	72027	-
ENSMUSG00000063362	11.76	0.112	-3.161	1.53464E-06	2.15394E-05	Alg11	asparagine-linked glycosylation 11 (alpha-1,2-mannosyltransferase)	protein_coding	207958	-
ENSMUSG00000063412	14.91	0.035	-4.83	2.52567E-09	1.14029E-07	Gm10131	-	protein_coding	-	-
ENSMUSG00000063445	8.51	0.157	-2.669	0.00082335	0.003600579	Nmral1	NmrA-like family domain containing 1	protein_coding	67824	-
ENSMUSG00000063457	1539.02	0.24	-2.057	4.66135E-08	1.1864E-06	Rps15	ribosomal protein S15	protein_coding	20054	-
ENSMUSG00000063480	43.41	0.163	-2.613	3.18834E-09	1.36084E-07	Snu13	SNU13 homolog, small nuclear ribonucleoprotein (U4/U6.U5)	protein_coding	20826	-
ENSMUSG00000063558	6.03	7.186	2.845	0.001292949	0.005204524	Aox1	aldehyde oxidase 1	protein_coding	11761	-

ENSMUSG00000 063632	8.45	8.943	3.161	5.93197E-06	6.51538E-05	Sox11	SRY (sex determining region Y)-box 11	protein_coding	20666	HMG
ENSMUSG00000 063787	18.83	0.188	-2.412	8.47352E-05	0.000570188	Chchd1	coiled-coil-helix-coiled-coil-helix domain containing 1	protein_coding	66121	-
ENSMUSG00000 063802	17.08	0.218	-2.196	0.000237522	0.001311223	Hspbp1	HSPA (heat shock 70kDa) binding protein, cytoplasmic cochaperone 1	protein_coding	66245	-
ENSMUSG00000 063816	2.36	0.139	-2.852	0.01678472	0.040901089	Gm4987	-	processed_pseud ogene	-	-
ENSMUSG00000 063838	258.31	5.382	2.428	5.76882E-09	2.22925E-07	Cdc42ep5	CDC42 effector protein (Rho GTPase binding) 5	protein_coding	58804	-
ENSMUSG00000 063873	109.9	4.373	2.129	6.08925E-09	2.31817E-07	Slc24a3	solute carrier family 24 (sodium/potassium/calcium exchanger), member 3	protein_coding	94249	-
ENSMUSG00000 063875	14.14	0.109	-3.199	4.89692E-08	1.23412E-06	Rps6-ps1	-	processed_pseud ogene	-	-
ENSMUSG00000 063932	7.16	5.184	2.374	0.004515711	0.014285706	Poteg	POTE ankyrin domain family, member G	protein_coding	70952	-
ENSMUSG00000 064023	41.92	0.093	-3.421	6.2342E-13	9.74108E-11	Klk8	kallikrein related-peptidase 8	protein_coding	259277	-
ENSMUSG00000 064030	44.25	0.248	-2.012	7.77726E-08	1.81258E-06	Pym1	PYM homolog 1, exon junction complex associated factor	protein_coding	78428	-
ENSMUSG00000 064032	13.66	0.183	-2.453	0.000134183	0.000829711	Gm10143	-	processed_transc ript	-	-
ENSMUSG00000 064037	15.04	0.103	-3.278	1.23174E-07	2.65068E-06	Gpn1	GPN-loop GTPase 1	protein_coding	74254	-
ENSMUSG00000 064179	19.3	5.45	2.446	1.13293E-05	0.00011088	Tnnt1	troponin T1, skeletal, slow	protein_coding	21955	-
ENSMUSG00000 064220	521.04	4.153	2.054	2.18827E-06	2.88067E-05	Hist2h2aa1	histone cluster 2, H2aa1	protein_coding	15267	-
ENSMUSG00000 064246	132.43	4.459	2.157	2.53045E-05	0.000214783	Chil1	chitinase-like 1	protein_coding	12654	-
ENSMUSG00000 064262	110.34	0.138	-2.857	6.01829E-09	2.30091E-07	Gimap8	GTPase, IMAP family member 8	protein_coding	243374	-
ENSMUSG00000 064267	208.18	0.079	-3.656	4.25574E-21	2.63694E-18	Hvcn1	hydrogen voltage-gated channel 1	protein_coding	74096	-
ENSMUSG00000 064329	13.26	4.218	2.077	0.002697502	0.009421071	Scn1a	sodium channel, voltage-gated, type I, alpha	protein_coding	20265	-
ENSMUSG00000 064337	24319.5 2	6.158	2.622	1.30652E-06	1.88187E-05	mt-Rnr1	-	Mt_rRNA	-	-
ENSMUSG00000 064339	50411.3 9	5.445	2.445	4.91424E-05	0.000367805	mt-Rnr2	-	Mt_rRNA	-	-
ENSMUSG00000 064347	6.37	0.223	-2.165	0.007904494	0.022449164	mt-Ta	-	Mt_tRNA	-	-
ENSMUSG00000 064348	9.37	0.249	-2.004	0.008347016	0.023450208	mt-Tn	-	Mt_tRNA	-	-
ENSMUSG00000 064767	13.57	0.005	-7.747	2.35442E-11	2.1048E-09	Snord35b	small nucleolar RNA, C/D box 35B	snoRNA	27212	-
ENSMUSG00000 065876	18.15	8.055	3.01	1.58093E-05	0.000146206	Gm25682	-	snRNA	-	-

ENSMUSG00000 065968	6.17	7.069	2.822	0.000905171	0.003886503	Ifitm7	interferon induced transmembrane protein 7	protein_coding	74482	-
ENSMUSG00000 065979	37.85	6.534	2.708	4.77757E-09	1.8993E-07	Cpped1	calcineurin-like phosphoesterase domain containing 1	protein_coding	223978	-
ENSMUSG00000 065999	8.74	0.186	-2.426	0.004254289	0.013658266	Zfp985	zinc finger protein 985	protein_coding	433804	zf-C2H2
ENSMUSG00000 066026	62.61	5.085	2.346	1.78035E-08	5.46857E-07	Dhrs3	dehydrogenase/reductase (SDR family) member 3	protein_coding	20148	-
ENSMUSG00000 066108	7.28	8.001	3	0.000136506	0.00084147	Muc5b	mucin 5, subtype B, tracheobronchial	protein_coding	74180	-
ENSMUSG00000 066189	10.71	4.047	2.017	0.00288398	0.009946686	Cacng3	calcium channel, voltage-dependent, gamma subunit 3	protein_coding	54376	-
ENSMUSG00000 066357	25.13	0.088	-3.505	3.24214E-09	1.37078E-07	Wdr6	WD repeat domain 6	protein_coding	83669	-
ENSMUSG00000 066477	5.27	9.115	3.188	0.000333951	0.001727337	Gm16551	predicted gene 16551	processed_transc ript	100503 019	-
ENSMUSG00000 066538	20.41	0.208	-2.264	0.000295283	0.001561032	Gm6254	-	processed_pseud ogene	-	-
ENSMUSG00000 066621	89.84	0.231	-2.115	8.27975E-10	4.33786E-08	Tecpr1	tectonin beta-propeller repeat containing 1	protein_coding	70381	-
ENSMUSG00000 066629	16.91	0.196	-2.354	0.000285483	0.001517707	Rpl36-ps3	-	processed_pseud ogene	-	-
ENSMUSG00000 066677	46.02	0.147	-2.764	1.46729E-06	2.08096E-05	Ifi208	interferon activated gene 208	protein_coding	100033 459	-
ENSMUSG00000 066724	37.19	0.193	-2.374	2.99152E-07	5.64057E-06	Gm10175	-	transcribed_proc essed_pseudogen e	-	-
ENSMUSG00000 066760	6.96	8.679	3.118	0.000144302	0.000880463	Psg16	pregnancy specific glycoprotein 16	protein_coding	26436	-
ENSMUSG00000 066772	8.67	4.706	2.235	0.007517402	0.021588651	Obox3	oocyte specific homeobox 3	protein_coding	246791	Homeobox
ENSMUSG00000 066838	4.24	0.131	-2.933	0.003789554	0.01241694	Zfp772	zinc finger protein 772	protein_coding	232855	zf-C2H2
ENSMUSG00000 066842	35.61	4.791	2.26	0.000101197	0.000659803	Hmcn1	hemicentin 1	protein_coding	545370	-
ENSMUSG00000 067085	130.02	4.371	2.128	5.21074E-09	2.04436E-07	Gm10197	-	protein_coding	-	-
ENSMUSG00000 067121	36.51	0.224	-2.158	8.16233E-09	2.90433E-07	Gm7027	-	processed_pseud ogene	-	-
ENSMUSG00000 067148	38.04	0.118	-3.082	1.77535E-10	1.15585E-08	Polr1c	polymerase (RNA) I polypeptide C	protein_coding	20016	-
ENSMUSG00000 067149	107.56	0.072	-3.787	4.05923E-07	7.23538E-06	Jchain	immunoglobulin joining chain	protein_coding	16069	-
ENSMUSG00000 067158	12.53	5.289	2.403	3.95385E-05	0.000308228	Col4a4	collagen, type IV, alpha 4	protein_coding	12829	-
ENSMUSG00000 067261	10.66	7.001	2.808	0.000158192	0.000943742	Foxd3	forkhead box D3	protein_coding	15221	Fork
ENSMUSG00000 067341	31.27	0.016	-5.934	3.53027E-19	1.71447E-16	H2-Eb2	histocompatibility 2, class II antigen E beta2	protein_coding	381091	-

ENSMUSG00000067344	54.26	0.171	-2.544	1.16124E-10	8.11792E-09	Rps25-ps1	-	processed_pseudogene	-	-
ENSMUSG00000067438	12.63	7.838	2.97	9.29858E-06	9.40271E-05	Hmx1	H6 homeobox 1	protein_coding	15371	Homeobox
ENSMUSG00000067547	20.59	0.143	-2.806	4.98403E-07	8.57836E-06	Gm7666	-	transcribed_processed_pseudogene	-	-
ENSMUSG00000067599	7.97	0.24	-2.062	0.020921668	0.048779221	Klra7	killer cell lectin-like receptor, subfamily A, member 7	protein_coding	16638	-
ENSMUSG00000067614	8.7	5.552	2.473	0.003668856	0.012092017	Krt86	keratin 86	protein_coding	16679	-
ENSMUSG00000067702	4.12	0.015	-6.087	1.6566E-05	0.00015203	Tuba3a	tubulin, alpha 3A	protein_coding	22144	-
ENSMUSG00000067724	4.36	6.033	2.593	0.006745894	0.01976471	Gbx1	gastrulation brain homeobox 1	protein_coding	231044	Homeobox
ENSMUSG00000067818	2136.32	5.096	2.349	1.08827E-05	0.00010721	Myl9	myosin, light polypeptide 9, regulatory	protein_coding	98932	-
ENSMUSG00000067825	8.51	0.126	-2.993	0.000155926	0.000933944	Pex26	peroxisomal biogenesis factor 26	protein_coding	74043	-
ENSMUSG00000068079	6.62	6.404	2.679	0.013787121	0.034873419	Tcf15	transcription factor 15	protein_coding	21407	bHLH
ENSMUSG00000068105	57.15	0.066	-3.923	1.80699E-16	6.24418E-14	Tnfrsf13c	tumor necrosis factor receptor superfamily, member 13c	protein_coding	72049	-
ENSMUSG00000068120	35.59	0.17	-2.554	1.21448E-08	4.03384E-07	Gm10231	-	processed_pseudogene	-	-
ENSMUSG00000068129	18.47	0.155	-2.687	7.25176E-07	1.16345E-05	Cst7	cystatin F (leukocystatin)	protein_coding	13011	-
ENSMUSG00000068165	11.99	0.184	-2.439	0.000680952	0.003075924	Gm10233	-	processed_pseudogene	-	-
ENSMUSG00000068180	4.58	4.169	2.06	0.005663262	0.017215896	AY512915	cDNA sequence AY512915	processed_transcript	414067	-
ENSMUSG00000068196	11.81	4.147	2.052	0.002688623	0.009402856	Col8a1	collagen, type VIII, alpha 1	protein_coding	12837	-
ENSMUSG00000068245	13.13	0.141	-2.823	0.000107557	0.00069322	Phf11d	PHD finger protein 11D	protein_coding	219132	-
ENSMUSG00000068270	18.32	5.761	2.526	5.29405E-07	9.00842E-06	Shroom4	shroom family member 4	protein_coding	208431	-
ENSMUSG00000068463	16.84	0.191	-2.385	5.93305E-05	0.000426956	B630019A10Rik	-	processed_transcript	-	-
ENSMUSG00000068580	14.23	0.218	-2.199	6.44715E-05	0.000456457	Zfyve19	zinc finger, FYVE domain containing 19	protein_coding	72008	-
ENSMUSG00000068587	20.72	4.251	2.088	5.77009E-05	0.000418244	Mgam	maltase-glucoamylase	protein_coding	232714	-
ENSMUSG00000068686	74.06	4.364	2.126	7.57845E-09	2.74551E-07	Cd59b	CD59b antigen	protein_coding	333883	-
ENSMUSG00000068735	66.45	0.064	-3.968	2.27247E-16	7.70452E-14	Trp53i11	transformation related protein 53 inducible protein 11	protein_coding	277414	-
ENSMUSG00000068744	22.12	6.779	2.761	3.09383E-08	8.51347E-07	Psrc1	proline/serine-rich coiled-coil 1	protein_coding	56742	-

ENSMUSG00000068747	67	4.451	2.154	1.05983E-08	3.62055E-07	Sort1	sortilin 1	protein_coding	20661	-
ENSMUSG00000068794	11.94	4.226	2.079	0.000219386	0.001232305	Col28a1	collagen, type XXVIII, alpha 1	protein_coding	213945	-
ENSMUSG00000068893	126.92	0.146	-2.775	2.34543E-17	9.16197E-15	Sprr2a2	small proline-rich protein 2A2	protein_coding	100303744	-
ENSMUSG00000068922	26.97	0.176	-2.507	8.76403E-06	8.99376E-05	Msto1	misato 1, mitochondrial distribution and morphology regulator	protein_coding	229524	-
ENSMUSG00000068962	8.11	4.144	2.051	0.005113931	0.015835296	Zfp114	zinc finger protein 114	protein_coding	232966	zf-C2H2
ENSMUSG00000068964	3.56	0.175	-2.517	0.016492349	0.040289505	Gm6361	-	pseudogene	-	-
ENSMUSG00000069014	5.99	0.193	-2.371	0.006654182	0.019575801	Gm5641	-	transcribed_processed_pseudogene	-	-
ENSMUSG00000069072	13.23	6.757	2.756	0.000294141	0.001556365	Slc7a14	solute carrier family 7 (cationic amino acid transporter, y+ system), member 14	protein_coding	241919	-
ENSMUSG00000069117	372.45	0.096	-3.388	1.13148E-12	1.63965E-10	Gm10260	-	protein_coding	-	-
ENSMUSG00000069270	64.04	4.295	2.103	2.02403E-06	2.70207E-05	Hist1h2ac	histone cluster 1, H2ac	protein_coding	319164	-
ENSMUSG00000069274	6.28	4.984	2.317	0.001260456	0.005102305	Hist1h4f	histone cluster 1, H4f	protein_coding	319157	-
ENSMUSG00000069303	423.16	5.219	2.384	2.49684E-10	1.51645E-08	Hist1h2br	histone cluster 1 H2br	protein_coding	665622	-
ENSMUSG00000069306	134.11	6.073	2.602	5.66414E-08	1.40385E-06	Hist1h4m	histone cluster 1, H4m	protein_coding	100041230	-
ENSMUSG00000069307	251.22	6.323	2.661	1.17189E-13	2.26046E-11	Hist1h2bq	histone cluster 1, H2bq	protein_coding	665596	-
ENSMUSG00000069607	27.7	5.944	2.571	0.000901993	0.003875636	Cd300ld3	CD300 molecule like family member D3	protein_coding	382551	-
ENSMUSG00000069609	20.99	6.459	2.691	0.000539264	0.00255001	Cd300ld4	CD300 molecule like family member D4	protein_coding	100043123	-
ENSMUSG00000070319	54.98	0.189	-2.4	2.50411E-10	1.51645E-08	Eif3g	eukaryotic translation initiation factor 3, subunit G	protein_coding	53356	-
ENSMUSG00000070343	44.76	0.224	-2.156	8.83757E-09	3.10161E-07	Gm10288	-	processed_pseudogene	-	-
ENSMUSG00000070366	13.36	6.118	2.613	0.000113596	0.000721532	Plpp4	phospholipid phosphatase 4	protein_coding	381925	-
ENSMUSG00000070392	541.21	4.053	2.019	1.53821E-06	2.15601E-05	Gm20634	-	processed_transcript	-	-
ENSMUSG00000070495	3.78	4.67	2.223	0.014064441	0.035390553	Ctcfl	CCCTC-binding factor (zinc finger protein)-like	protein_coding	664799	zf-C2H2
ENSMUSG00000070501	67.64	0.049	-4.36	9.46471E-21	5.48618E-18	Ifi214	interferon activated gene 214	protein_coding	545384	-
ENSMUSG00000070576	12.43	5.943	2.571	0.000207819	0.001177917	Mn1	meningioma 1	protein_coding	433938	-

ENSMUSG00000070683	5.04	6.145	2.619	0.000995627	0.004204566	Lactbl1	lactamase, beta-like 1	protein_coding	242707	-
ENSMUSG00000070690	5.86	4.222	2.078	0.017487616	0.042281347	5830473C10Rik	RIKEN cDNA 5830473C10 gene	protein_coding	622307	-
ENSMUSG00000070720	5.92	4.418	2.143	0.005201015	0.016057911	Tmem200b	transmembrane protein 200B	protein_coding	623230	-
ENSMUSG00000070803	18.95	5.798	2.536	0.000515744	0.002460809	Cited4	Cbp/p300-interacting transactivator, with Glu/Asp-rich carboxy-terminal domain, 4	protein_coding	56222	-
ENSMUSG00000070880	49.19	4.439	2.15	2.53845E-07	4.88116E-06	Gad1	glutamate decarboxylase 1	protein_coding	14415	-
ENSMUSG00000071141	50.26	0.128	-2.969	8.48957E-11	6.3828E-09	Rpl36a-ps3	-	processed_pseudogene	-	-
ENSMUSG00000071178	8.71	5.518	2.464	0.008124944	0.022962744	Serpina1b	serine (or cysteine) preptidase inhibitor, clade A, member 1B	protein_coding	20701	-
ENSMUSG00000071302	11.74	0.192	-2.379	0.002204001	0.008036463	2610044O15Rik8	RIKEN cDNA 2610044O15 gene	protein_coding	72139	-
ENSMUSG00000071324	12.99	4.186	2.066	0.005890075	0.017770107	Armc2	armadillo repeat containing 2	protein_coding	213402	-
ENSMUSG00000071350	12.3	0.171	-2.546	0.000158007	0.000942952	Setdb2	SET domain, bifurcated 2	protein_coding	239122	MBD
ENSMUSG00000071451	22.8	0.231	-2.114	2.70557E-06	3.42832E-05	Psmg4	proteasome (prosome, macropain) assembly chaperone 4	protein_coding	69666	-
ENSMUSG00000071553	6.37	4.207	2.073	0.007037731	0.020456324	Cpa2	carboxypeptidase A2, pancreatic	protein_coding	232680	-
ENSMUSG00000071573	22.62	4.72	2.239	1.12633E-05	0.000110341	Rnls	renalase, FAD-dependent amine oxidase	protein_coding	67795	-
ENSMUSG00000071604	10.68	6.373	2.672	0.001443571	0.005697237	Fam189a2	family with sequence similarity 189, member A2	protein_coding	381217	-
ENSMUSG00000071644	459.33	0.208	-2.266	7.80549E-10	4.1131E-08	Eef1g	eukaryotic translation elongation factor 1 gamma	protein_coding	67160	-
ENSMUSG00000071654	25.95	0.16	-2.643	1.64724E-08	5.12985E-07	Uqcc3	ubiquinol-cytochrome c reductase complex assembly factor 3	protein_coding	107197	-
ENSMUSG00000071661	2.58	0.125	-2.995	0.013605415	0.034525589	Zbtb3	zinc finger and BTB domain containing 3	protein_coding	75291	ZBTB
ENSMUSG00000071716	39.03	0.171	-2.544	4.13068E-08	1.08356E-06	Apol7e	apolipoprotein L 7e	protein_coding	666348	-
ENSMUSG00000071753	6.69	5.034	2.332	0.001707888	0.006522645	C230004F18Rik	RIKEN cDNA C230004F18 gene	processed_transcript	331424	-
ENSMUSG00000071796	88.6	0.246	-2.021	1.72377E-08	5.33121E-07	6820431F20Rik	cadherin 11 pseudogene	transcribed_unprocessed_pseudogene	547150	-
ENSMUSG00000071847	42.69	5.612	2.489	1.8974E-12	2.41804E-10	Apcdd1	adenomatosis polyposis coli down-regulated 1	protein_coding	494504	-
ENSMUSG00000071856	37.27	4.273	2.095	4.75519E-06	5.4633E-05	Mcc	mutated in colorectal cancers	protein_coding	328949	-
ENSMUSG00000071984	10.02	6.166	2.624	0.000487055	0.002344468	Fndc1	fibronectin type III domain containing 1	protein_coding	68655	-

ENSMUSG00000072109	50.85	0.097	-3.367	4.82767E-07	8.37339E-06	A530040E14Rik	-	antisense_RNA	-	-
ENSMUSG00000072214	11.14	5.215	2.383	0.001166796	0.00478898	Sept5	septin 5	protein_coding	18951	-
ENSMUSG00000072494	17.23	5.988	2.582	0.017538063	0.042386207	Ppp1r3e	protein phosphatase 1, regulatory subunit 3E	protein_coding	105651	-
ENSMUSG00000072573	4.53	6.307	2.657	0.017426006	0.042189154	Gm10369	-	antisense_RNA	-	-
ENSMUSG00000072763	6.82	0.079	-3.654	4.91457E-05	0.000367805	5430403G16Rik	-	protein_coding	-	zf-C2H2
ENSMUSG00000072769	79.64	5.882	2.556	1.37236E-11	1.3258E-09	Gm10419	-	lincRNA	-	-
ENSMUSG00000072875	7.95	5.359	2.422	0.00078847	0.003469154	Gpr27	G protein-coupled receptor 27	protein_coding	14761	-
ENSMUSG00000072898	5.93	0.182	-2.461	0.004483876	0.014222553	Gm10434	-	pseudogene	-	-
ENSMUSG00000072949	18.81	5.426	2.44	1.06289E-07	2.356E-06	Acot1	acyl-CoA thioesterase 1	protein_coding	26897	-
ENSMUSG00000073008	25.51	0.109	-3.2	2.91591E-10	1.71229E-08	Gpr174	G protein-coupled receptor 174	protein_coding	213439	-
ENSMUSG00000073045	7.65	4.203	2.071	0.007768927	0.022130603	Gm5570	predicted gene 5570	lincRNA	434019	-
ENSMUSG00000073154	12.15	4.579	2.195	0.000446944	0.002196099	9330158H04Rik	RIKEN cDNA 9330158H04 gene	lincRNA	319472	-
ENSMUSG00000073414	139.2	5.734	2.52	4.20618E-09	1.71385E-07	G6b	megakaryocyte and platelet inhibitory receptor G6b	protein_coding	106722	-
ENSMUSG00000073421	655.19	0.016	-5.95	2.59011E-29	5.1713E-26	H2-Ab1	histocompatibility 2, class II antigen A, beta 1	protein_coding	14961	-
ENSMUSG00000073422	46.67	0.205	-2.288	1.3038E-06	1.88025E-05	H2-Ke6	H2-K region expressed gene 6	protein_coding	14979	-
ENSMUSG00000073430	2.01	0.032	-4.979	0.001833171	0.00691006	Gm10505	-	lincRNA	-	-
ENSMUSG00000073481	54.87	0.202	-2.305	7.73813E-10	4.09166E-08	Marc2	mitochondrial amidoxime reducing component 2	protein_coding	67247	-
ENSMUSG00000073514	39.26	4.759	2.251	2.75574E-10	1.6506E-08	Dok6	docking protein 6	protein_coding	623279	-
ENSMUSG00000073538	12.36	0.104	-3.259	1.92096E-06	2.59337E-05	E330020D12Rik	-	processed_transcript	-	-
ENSMUSG00000073551	10.53	4.925	2.3	0.000823287	0.003600579	Spink13	serine peptidase inhibitor, Kazal type 13	protein_coding	100038417	-
ENSMUSG00000073628	16.85	0.124	-3.014	4.16453E-06	4.91671E-05	Gm10552	-	antisense_RNA	-	-
ENSMUSG00000073631	9.81	0.208	-2.266	0.012955638	0.033242876	Gm10553	-	protein_coding	-	-
ENSMUSG00000073647	7.55	0.072	-3.793	1.15829E-05	0.00011281	Gm10557	-	pseudogene	-	-
ENSMUSG00000073676	49.64	0.083	-3.593	1.98471E-13	3.53101E-11	Hspe1	heat shock protein 1 (chaperonin 10)	protein_coding	15528	-

ENSMUSG00000 073894	8.46	7.799	2.963	0.00021348	0.001203268	Rbmxl2	RNA binding motif protein, X-linked-like 2	protein_coding	76572	-
ENSMUSG00000 073991	20.47	4.893	2.291	2.30585E-07	4.5184E-06	Cnbd1	-	protein_coding	-	-
ENSMUSG00000 074067	5.38	10.709	3.421	0.003045203	0.010402901	Gm10619	predicted gene 10619	lincRNA	100579 147	-
ENSMUSG00000 074071	78.42	0.198	-2.336	1.16558E-10	8.11792E-09	Fam169b	family with sequence similarity 169, member B	protein_coding	434197	-
ENSMUSG00000 074129	2941.07	0.203	-2.301	3.43922E-07	6.30606E-06	Rpl13a	ribosomal protein L13A	protein_coding	22121	-
ENSMUSG00000 074164	31.74	4.634	2.212	5.95693E-08	1.45831E-06	Vmn2r-ps54	vomeronasal 2, receptor, pseudogene 54	transcribed_unpr ocessed_pseudog ene	75835	-
ENSMUSG00000 074235	21.12	5.474	2.453	4.27697E-06	5.01651E-05	Gm10649	predicted gene 10649	bidirectional_pro moter_lncRNA	102640 923	-
ENSMUSG00000 074284	6.31	0.182	-2.455	0.007108305	0.020624758	Gm10658	-	antisense_RNA	-	-
ENSMUSG00000 074340	17.74	4.854	2.279	0.00101217	0.004260409	Ovgp1	oviductal glycoprotein 1	protein_coding	12659	-
ENSMUSG00000 074343	4.27	5.108	2.353	0.008945361	0.024786305	Gldnos	gliomedin, opposite strand	antisense_RNA	73567	-
ENSMUSG00000 074452	7.13	4.465	2.159	0.015027982	0.037370303	Pate2	prostate and testis expressed 2	protein_coding	330921	-
ENSMUSG00000 074491	8.74	5.211	2.382	9.07544E-05	0.000602426	Clec4g	C-type lectin domain family 4, member g	protein_coding	75863	-
ENSMUSG00000 074555	9.08	5.919	2.565	0.000114482	0.000725365	Gm10714	predicted gene 10714	antisense_RNA	100038 374	-
ENSMUSG00000 074570	65.36	4.061	2.022	7.02019E-08	1.66859E-06	Cass4	Cas scaffolding protein family member 4	protein_coding	320664	-
ENSMUSG00000 074607	16.43	4.95	2.308	0.001217299	0.004955517	Tox2	TOX high mobility group box family member 2	protein_coding	269389	HMG
ENSMUSG00000 074625	6.42	4.113	2.04	0.00388125	0.01266658	Arhgap40	Rho GTPase activating protein 40	protein_coding	545481	-
ENSMUSG00000 074652	9.04	6.143	2.619	0.000146902	0.000892994	Myh7b	myosin, heavy chain 7B, cardiac muscle, beta	protein_coding	668940	-
ENSMUSG00000 074657	12.39	7.178	2.843	6.48411E-06	7.00356E-05	Kif5a	kinesin family member 5A	protein_coding	16572	-
ENSMUSG00000 074764	13.09	6.347	2.666	8.64919E-07	1.34212E-05	Sel1l2	sel-1 suppressor of lin-12-like 2 (C. elegans)	protein_coding	228684	-
ENSMUSG00000 074771	6.63	4.709	2.235	0.007919741	0.022481804	Ankef1	ankyrin repeat and EF-hand domain containing 1	protein_coding	319196	-
ENSMUSG00000 074776	22.39	4.069	2.025	1.26355E-06	1.83251E-05	Gm10754	predicted gene 10754	lincRNA	100038 699	-
ENSMUSG00000 074783	8.43	19.734	4.303	5.73765E-07	9.6265E-06	AU019990	expressed sequence AU019990	lincRNA	767814	-
ENSMUSG00000 074797	12.7	0.247	-2.019	0.002223318	0.00809704	Itpa	inosine triphosphatase (nucleoside triphosphate pyrophosphatase)	protein_coding	16434	-
ENSMUSG00000 074818	5.77	7.905	2.983	0.002117311	0.007774001	Pdzd7	PDZ domain containing 7	protein_coding	100503 041	-

ENSMUSG00000074874	3195.21	4.097	2.035	3.34993E-05	0.000270053	Ctla2b	cytotoxic T lymphocyte-associated protein 2 beta	protein_coding	13025	-
ENSMUSG00000074918	184.92	4.592	2.199	3.47708E-08	9.36726E-07	Inafm2	InaF motif containing 2	protein_coding	100043272	-
ENSMUSG00000075015	13.72	4.061	2.022	0.005931037	0.01787868	Gm10801	-	protein_coding	-	-
ENSMUSG00000075033	79.44	0.234	-2.098	9.26795E-11	6.76975E-09	Nxpe3	neurexophilin and PC-esterase domain family, member 3	protein_coding	385658	-
ENSMUSG00000075054	14.36	0.211	-2.243	6.00701E-05	0.000430965	Yae1d1	Yae1 domain containing 1	protein_coding	67008	-
ENSMUSG00000075070	9.89	7.009	2.809	0.00024833	0.001358911	4932412D23Rik	RIKEN cDNA 4932412D23 gene	processed_transcript	75722	-
ENSMUSG00000075229	11.65	0.184	-2.441	0.000219147	0.001231537	Ccdc58	coiled-coil domain containing 58	protein_coding	381045	-
ENSMUSG00000075268	11.38	0.192	-2.378	0.000312401	0.001637554	Gm10819	-	processed_pseudogene	-	-
ENSMUSG00000075279	8.88	0.126	-2.987	0.00038521	0.001938348	Mrpl23-ps1	-	processed_pseudogene	-	-
ENSMUSG00000075286	28.74	4.792	2.261	4.6657E-07	8.13961E-06	Gm1968	predicted gene 1968	protein_coding	328657	-
ENSMUSG00000075330	9.15	5.395	2.432	0.001270907	0.005135355	A930003A15Rik	RIKEN cDNA A930003A15 gene	protein_coding	68162	-
ENSMUSG00000075335	3.86	11.387	3.509	7.3363E-05	0.000507424	4930588K23Rik	-	antisense_RNA	-	-
ENSMUSG00000075359	8.52	0.184	-2.441	0.001059544	0.004427661	Gm20775	-	unprocessed_pseudogene	-	-
ENSMUSG00000075389	5.8	6.474	2.695	0.005093113	0.015800786	2810410L24Rik	-	processed_transcript	-	-
ENSMUSG00000075520	102.67	4.648	2.217	1.31944E-10	8.92835E-09	Malrd1	MAM and LDL receptor class A domain containing 1	protein_coding	102635496	-
ENSMUSG00000075585	19.78	4.038	2.014	3.74891E-05	0.000295198	6330403L08Rik	-	antisense_RNA	-	-
ENSMUSG00000075602	182.75	0.155	-2.687	1.10337E-10	7.7751E-09	Ly6a	lymphocyte antigen 6 complex, locus A	protein_coding	110454	-
ENSMUSG00000076137	30.05	0.226	-2.147	7.92838E-06	8.27323E-05	Gm26384	-	miRNA	-	-
ENSMUSG00000076434	8.77	5.582	2.481	0.000145552	0.000886727	Wfdc3	WAP four-disulfide core domain 3	protein_coding	71856	-
ENSMUSG00000076436	5.44	14.411	3.849	9.97688E-05	0.000652857	Oxct2a	3-oxoacid CoA transferase 2A	protein_coding	64059	-
ENSMUSG00000076437	20.56	0.155	-2.689	1.37513E-05	0.000130113	Selenoh	selenoprotein H	protein_coding	72657	-
ENSMUSG00000076454	11.95	0.134	-2.9	2.79761E-05	0.000233598	Mir762	microRNA 762	miRNA	791073	-
ENSMUSG00000076462	14.12	0.225	-2.154	0.002896601	0.009978724	Trbv2	-	TR_V_gene	-	-
ENSMUSG00000076465	7.34	0.073	-3.776	1.47901E-05	0.000138418	Trbv5	-	TR_V_gene	-	-

ENSMUSG00000076467	19.51	0.078	-3.687	4.33633E-08	1.11632E-06	Trbv13-1	-	TR_V_gene	-	-
ENSMUSG00000076469	22.5	0.217	-2.201	1.15543E-05	0.000112653	Trbv13-2	-	TR_V_gene	-	-
ENSMUSG00000076471	5.54	0.011	-6.502	7.94334E-07	1.24876E-05	Trbv14	-	TR_V_gene	-	-
ENSMUSG00000076473	13.37	0.116	-3.109	4.29225E-06	5.03115E-05	Trbv16	-	TR_V_gene	-	-
ENSMUSG00000076483	9.33	0.145	-2.787	0.000166958	0.000986216	Trbj1-1	-	TR_J_gene	-	-
ENSMUSG00000076485	6.2	0.049	-4.356	2.83324E-05	0.00023575	Trbj1-3	-	TR_J_gene	-	-
ENSMUSG00000076490	260.58	0.173	-2.532	2.69717E-12	3.29697E-10	Trbc1	-	TR_C_gene	-	-
ENSMUSG00000076492	11.03	0.071	-3.824	5.59706E-07	9.40819E-06	Trbj2-1	-	TR_J_gene	-	-
ENSMUSG00000076496	7.57	0.183	-2.452	0.002510049	0.00889958	Trbj2-5	-	TR_J_gene	-	-
ENSMUSG00000076497	10.92	0.2	-2.322	0.001583099	0.00613737	Trbj2-7	-	TR_J_gene	-	-
ENSMUSG00000076498	347.33	0.186	-2.427	1.71409E-11	1.62965E-09	Trbc2	-	TR_C_gene	-	-
ENSMUSG00000076501	8.43	0.135	-2.889	0.001019899	0.004288923	Igkv2-137	-	IG_V_gene	-	-
ENSMUSG00000076508	10.55	0.098	-3.352	2.94243E-06	3.68976E-05	Igkv17-127	-	IG_V_gene	-	-
ENSMUSG00000076514	10.36	0.05	-4.31	2.91998E-08	8.10959E-07	Igkv17-121	-	IG_V_gene	-	-
ENSMUSG00000076522	5.46	0.011	-6.507	6.41423E-07	1.0545E-05	Igkv16-104	-	IG_V_gene	-	-
ENSMUSG00000076523	5.2	0.012	-6.37	1.76163E-05	0.000160196	Igkv15-103	-	IG_V_gene	-	-
ENSMUSG00000076538	7.92	0.008	-6.987	1.92986E-08	5.85048E-07	Igkv13-84	-	IG_V_gene	-	-
ENSMUSG00000076540	4.5	0.014	-6.139	4.31998E-06	5.05376E-05	Igkv4-80	-	IG_V_gene	-	-
ENSMUSG00000076541	3.32	0.019	-5.706	8.73396E-05	0.000584636	Igkv4-79	-	IG_V_gene	-	-
ENSMUSG00000076543	4.09	0.016	-6.003	7.75799E-05	0.00053106	Igkv4-74	-	IG_V_gene	-	-
ENSMUSG00000076545	11.94	0.088	-3.503	1.60668E-06	2.23802E-05	Igkv4-72	-	IG_V_gene	-	-
ENSMUSG00000076547	5.91	0.097	-3.366	0.000270203	0.001451503	Igkv4-70	-	IG_V_gene	-	-
ENSMUSG00000076549	11.54	0.202	-2.306	0.004389956	0.014000616	Igkv4-68	-	IG_V_gene	-	-
ENSMUSG00000076550	7.26	0.009	-6.835	2.40631E-07	4.67353E-06	Igkv4-63	-	IG_V_gene	-	-

ENSMUSG00000 076555	6.61	0.009	-6.754	2.32102E-07	4.54318E-06	Igkv4-57-1	-	IG_V_gene	-	-
ENSMUSG00000 076556	5.69	0.146	-2.776	0.000783101	0.003447215	Igkv4-57	-	IG_V_gene	-	-
ENSMUSG00000 076563	8.38	0.135	-2.886	0.011001063	0.029294325	Igkv5-48	-	IG_V_gene	-	-
ENSMUSG00000 076564	7.06	0.009	-6.838	5.45875E-08	1.35856E-06	Igkv12-46	-	IG_V_gene	-	-
ENSMUSG00000 076576	11.91	0.005	-7.594	5.92043E-11	4.6254E-09	Igkv6-32	-	IG_V_gene	-	-
ENSMUSG00000 076577	11.23	0.071	-3.821	5.29005E-07	9.00842E-06	Igkv8-30	-	IG_V_gene	-	-
ENSMUSG00000 076580	4.8	0.238	-2.073	0.010363912	0.027928784	Igkv8-27	-	IG_V_gene	-	-
ENSMUSG00000 076594	9.31	0.176	-2.506	0.001119944	0.004632659	Igkv6-13	-	IG_V_gene	-	-
ENSMUSG00000 076598	8.12	0.036	-4.791	7.78554E-07	1.22934E-05	Igkv3-7	-	IG_V_gene	-	-
ENSMUSG00000 076604	44.48	0.056	-4.163	3.23114E-16	1.05564E-13	Igkj1	-	IG_J_gene	-	-
ENSMUSG00000 076605	39.22	0.043	-4.526	4.13409E-18	1.81184E-15	Igkj2	-	IG_J_gene	-	-
ENSMUSG00000 076607	21.68	0.035	-4.837	1.29535E-14	3.2328E-12	Igkj4	-	IG_J_gene	-	-
ENSMUSG00000 076608	33.49	0.045	-4.488	5.17545E-16	1.63154E-13	Igkj5	-	IG_J_gene	-	-
ENSMUSG00000 076609	957.95	0.082	-3.603	3.84809E-06	4.611E-05	Igkc	-	IG_C_gene	-	-
ENSMUSG00000 076612	9.59	0.029	-5.096	8.07725E-08	1.86555E-06	Ighg2c	-	IG_C_gene	-	-
ENSMUSG00000 076613	15.56	0.225	-2.15	0.001203144	0.004906074	Ighg2b	-	IG_C_gene	-	-
ENSMUSG00000 076615	14.1	0.074	-3.749	3.11125E-06	3.85294E-05	Ighg3	-	IG_C_gene	-	-
ENSMUSG00000 076617	821.6	0.084	-3.574	9.5519E-18	3.99159E-15	Ighm	-	IG_C_gene	-	-
ENSMUSG00000 076618	16.45	0.047	-4.406	2.0743E-09	9.65622E-08	Ighj4	-	IG_J_gene	-	-
ENSMUSG00000 076619	12.9	0.042	-4.563	3.4705E-08	9.36359E-07	Ighj3	-	IG_J_gene	-	-
ENSMUSG00000 076620	16.56	0.046	-4.44	4.13652E-09	1.6893E-07	Ighj2	-	IG_J_gene	-	-
ENSMUSG00000 076621	14.65	0.168	-2.571	2.01511E-05	0.000179166	Ighj1	-	IG_J_gene	-	-
ENSMUSG00000 076666	3.38	0.018	-5.811	2.70976E-05	0.000227674	Ighv14-4	-	IG_V_gene	-	-
ENSMUSG00000 076672	4.94	0.012	-6.332	2.81471E-05	0.00023459	Ighv3-6	-	IG_V_gene	-	-

ENSMUSG00000 076870	16.1	10.762	3.428	1.39049E-06	1.98931E-05	Trdj1	-	TR_J_gene	-	-
ENSMUSG00000 076928	87.73	0.144	-2.793	4.54354E-14	9.71939E-12	Trac	-	TR_C_gene	-	-
ENSMUSG00000 076934	10.78	0.006	-7.405	6.17299E-09	2.34508E-07	Iglv1	-	IG_V_gene	-	-
ENSMUSG00000 076937	128.86	0.019	-5.751	3.13479E-30	8.04702E-27	Iglc2	-	IG_C_gene	-	-
ENSMUSG00000 076939	17.58	0.03	-5.077	1.73011E-12	2.26178E-10	Iglv3	-	IG_V_gene	-	-
ENSMUSG00000 076940	8.34	0.067	-3.894	2.86993E-05	0.000238173	Iglv2	-	IG_V_gene	-	-
ENSMUSG00000 078180	11.54	0.21	-2.248	0.000829389	0.00362082	Gm20900	-	processed_pseud ogene	-	-
ENSMUSG00000 078302	8.92	9.912	3.309	0.001945121	0.007248421	Foxd1	forkhead box D1	protein_coding	15229	Fork
ENSMUSG00000 078308	4.16	4.381	2.131	0.011107783	0.029517267	Gm7293	-	lincRNA	-	-
ENSMUSG00000 078377	227.1	0.207	-2.272	3.33691E-12	3.91901E-10	Gm4294	-	processed_pseud ogene	-	-
ENSMUSG00000 078453	36.06	0.194	-2.363	2.73643E-09	1.2171E-07	Abracl	ABRA C-terminal like	protein_coding	73112	-
ENSMUSG00000 078519	5.64	6.152	2.621	0.000944336	0.004021897	2310026L22R ik	-	lincRNA	-	-
ENSMUSG00000 078784	8.73	0.241	-2.052	0.012809011	0.032960779	1810022K09R ik	RIKEN cDNA 1810022K09 gene	protein_coding	69126	-
ENSMUSG00000 078789	14.96	0.238	-2.072	0.000186192	0.001076962	Dph1	diphthamide biosynthesis 1	protein_coding	116905	-
ENSMUSG00000 078810	143.99	5.549	2.472	5.67918E-11	4.47584E-09	Gp6	glycoprotein 6 (platelet)	protein_coding	243816	-
ENSMUSG00000 078861	5.59	0.199	-2.331	0.002808424	0.009736556	Zfp931	zinc finger protein 931	protein_coding	353208	zf-C2H2
ENSMUSG00000 078862	18.98	0.197	-2.34	1.37579E-05	0.000130113	Gm14326	predicted gene 14326	protein_coding	665211	zf-C2H2
ENSMUSG00000 078870	32.25	0.232	-2.108	0.000399615	0.001998518	Gm14410	-	protein_coding	-	zf-C2H2
ENSMUSG00000 078926	8	4.423	2.145	0.003962673	0.01287618	Cdc20b	cell division cycle 20B	protein_coding	238896	-
ENSMUSG00000 078995	8.1	0.171	-2.551	0.002154107	0.007884937	Zfp456	zinc finger protein 456	protein_coding	408065	zf-C2H2
ENSMUSG00000 079018	75.07	0.22	-2.183	1.30846E-08	4.26709E-07	Ly6c1	lymphocyte antigen 6 complex, locus C1	protein_coding	17067	-
ENSMUSG00000 079101	41.36	5.61	2.488	5.97045E-07	9.88784E-06	Gm2904	-	processed_pseud ogene	-	-
ENSMUSG00000 079104	17.08	0.245	-2.029	0.000244016	0.001341303	Prps1l3	phosphoribosyl pyrophosphate synthetase 1-like 3	protein_coding	328099	-
ENSMUSG00000 079137	9.35	0.117	-3.093	7.71763E-05	0.0005287	Rpl27-ps1	-	processed_pseud ogene	-	-

ENSMUSG00000079139	27.94	0.227	-2.139	9.22937E-08	2.0887E-06	Gm4204	-	processed_pseudogene	-	-
ENSMUSG00000079156	4.49	12.504	3.644	0.002347636	0.008458928	Smok3b	sperm motility kinase 3B	protein_coding	622474	-
ENSMUSG00000079157	48.95	4.231	2.081	1.27754E-07	2.72425E-06	Fam155a	family with sequence similarity 155, member A	protein_coding	270028	-
ENSMUSG00000079224	3.74	0.163	-2.615	0.010353958	0.027906146	Gm6565	-	processed_pseudogene	-	-
ENSMUSG00000079225	9.9	0.223	-2.163	0.000897651	0.003863446	Gm9531	-	transcribed_processed_pseudogene	-	-
ENSMUSG00000079235	6.95	5.001	2.322	0.004814318	0.015063291	Ccdc13	coiled-coil domain containing 13	protein_coding	100502861	-
ENSMUSG00000079278	8.64	6.402	2.679	0.000486534	0.002343843	Tmem233	transmembrane protein 233	protein_coding	545798	-
ENSMUSG00000079324	9.76	4.931	2.302	0.00147376	0.005792212	4932414N04Rik	RIKEN cDNA 4932414N04 gene	protein_coding	75721	-
ENSMUSG00000079363	62.31	0.24	-2.059	9.98554E-06	9.93523E-05	Gbp4	guanylate binding protein 4	protein_coding	17472	-
ENSMUSG00000079364	49.99	5.432	2.442	9.32725E-08	2.10554E-06	Gm3558	predicted gene 3558	protein_coding	100041874	-
ENSMUSG00000079410	42.02	5.717	2.515	3.51196E-07	6.42426E-06	Gm2897	predicted gene 2897	protein_coding	100040671	-
ENSMUSG00000079419	209.92	0.172	-2.538	1.70761E-12	2.25618E-10	Ms4a6c	membrane-spanning 4-domains, subfamily A, member 6C	protein_coding	73656	-
ENSMUSG00000079455	20.85	0.214	-2.223	0.000465722	0.002265444	Gm16026	-	transcribed_unprocessed_pseudogene	-	-
ENSMUSG00000079523	700.39	0.218	-2.2	7.94637E-09	2.85007E-07	Tmsb10	thymosin, beta 10	protein_coding	19240	-
ENSMUSG00000079543	8.06	0.067	-3.894	8.38613E-06	8.68533E-05	Igkv13-85	-	IG_V_gene	-	-
ENSMUSG00000079547	115.11	0.037	-4.771	8.67462E-22	5.99517E-19	H2-DMb1	histocompatibility 2, class II, locus Mb1	protein_coding	14999	-
ENSMUSG00000079560	54.11	4.538	2.182	2.59173E-07	4.96491E-06	Hoxa3	homeobox A3	protein_coding	15400	Homeobox
ENSMUSG00000079564	7.2	4.182	2.064	0.002494847	0.008859666	Gm11149	predicted gene 11149	antisense_RNA	100036537	-
ENSMUSG00000079575	4.3	0.132	-2.923	0.003434579	0.011450085	Rbpsuh-rs3	-	processed_pseudogene	-	-
ENSMUSG00000079584	7.55	7.648	2.935	0.000242608	0.001334382	Gm364	predicted gene 364	protein_coding	245423	-
ENSMUSG00000079593	170.78	6.436	2.686	1.12698E-11	1.13133E-09	Gm14597	RIKEN cDNA 4933416I08 gene	protein_coding	71159	-
ENSMUSG00000079677	19.23	0.206	-2.283	0.000738448	0.0032881	Fdx1l	ferredoxin 1-like	protein_coding	68165	-
ENSMUSG00000079700	66.18	5.521	2.465	7.03913E-08	1.67089E-06	Fpr3	formyl peptide receptor 3	protein_coding	14294	-

ENSMUSG00000079845	7.3	0.107	-3.231	0.000202683	0.001153996	Xlr4a	X-linked lymphocyte-regulated 4A	protein_coding	434794	-
ENSMUSG00000080002	3.77	0.154	-2.701	0.00367283	0.012101384	Gm5566	-	processed_pseudogene	-	-
ENSMUSG00000080746	46.09	0.134	-2.902	1.1825E-13	2.26046E-11	Rpsa-ps12	-	processed_pseudogene	-	-
ENSMUSG00000080816	4.28	0.015	-6.086	5.07444E-06	5.7618E-05	Gm12447	-	processed_pseudogene	-	-
ENSMUSG00000080875	5.13	0.112	-3.154	0.00046903	0.002277836	Gm7332	-	processed_pseudogene	-	-
ENSMUSG00000080877	8.79	0.007	-7.155	6.83123E-08	1.63449E-06	Rpl22-ps1	-	processed_pseudogene	-	-
ENSMUSG00000080893	6.18	0.24	-2.06	0.013880265	0.035039968	Gm15920	-	processed_pseudogene	-	-
ENSMUSG00000080969	5.87	0.051	-4.283	5.47296E-05	0.000401403	Gm12372	-	processed_pseudogene	-	-
ENSMUSG00000080977	3.67	0.018	-5.836	5.5589E-05	0.000405554	Gm13772	-	processed_pseudogene	-	-
ENSMUSG00000081023	7.81	0.106	-3.238	0.000192979	0.001109292	Gm15766	-	processed_pseudogene	-	-
ENSMUSG00000081076	5.86	0.197	-2.343	0.00408256	0.013198905	Rpsa-ps4	-	processed_pseudogene	-	-
ENSMUSG00000081189	53.74	6.885	2.783	6.70581E-07	1.0885E-05	Hspd1-ps4	-	processed_pseudogene	-	-
ENSMUSG00000081219	11.36	0.136	-2.875	1.96309E-05	0.000175389	Bambi-ps1	-	processed_pseudogene	-	-
ENSMUSG00000081308	4.5	7.808	2.965	0.000481281	0.00232602	Gm14480	-	processed_pseudogene	-	-
ENSMUSG00000081325	7.45	0.195	-2.355	0.00436945	0.013953198	Gm12599	-	processed_pseudogene	-	-
ENSMUSG00000081406	405.07	0.221	-2.177	1.86845E-10	1.19481E-08	Rps6-ps4	40S ribosomal protein S6	processed_pseudogene	105244208	-
ENSMUSG00000081520	5.3	0.099	-3.336	0.00037137	0.001878175	Gm16200	-	processed_pseudogene	-	-
ENSMUSG00000081603	7.74	0.067	-3.906	5.22744E-06	5.90767E-05	Gm14681	-	processed_pseudogene	-	-
ENSMUSG00000081695	27.16	0.196	-2.35	4.37011E-06	5.09912E-05	Rpsa-ps5	-	processed_pseudogene	-	-
ENSMUSG00000081926	36.76	0.174	-2.526	1.18449E-09	5.94528E-08	Gm15536	-	processed_pseudogene	-	-
ENSMUSG00000081992	151.2	0.247	-2.018	6.91339E-09	2.55611E-07	Gm13408	-	processed_pseudogene	-	-
ENSMUSG00000082028	2.81	0.023	-5.452	0.000390589	0.001959381	Gm1826	-	processed_pseudogene	-	-
ENSMUSG00000082064	50.42	0.156	-2.683	3.20862E-08	8.7756E-07	Rpl5-ps2	-	processed_pseudogene	-	-
ENSMUSG00000082109	15.88	0.218	-2.2	0.000158002	0.000942952	Rpl19-ps12	-	processed_pseudogene	-	-

ENSMUSG00000 082128	28.53	0.164	-2.608	9.45325E-07	1.44443E-05	Gm11824	-	processed_pseud ogene	-	-
ENSMUSG00000 082185	116.45	4.051	2.018	2.98732E-06	3.73031E-05	Gm12428	-	processed_pseud ogene	-	-
ENSMUSG00000 082193	239.26	0.182	-2.454	4.48605E-12	5.0698E-10	Rpl5-ps1	-	processed_pseud ogene	-	-
ENSMUSG00000 082195	5.55	0.155	-2.686	0.002830522	0.009791773	Gm13034	-	processed_pseud ogene	-	-
ENSMUSG00000 082465	108.34	0.235	-2.09	2.08072E-11	1.90757E-09	Rps6-ps3	-	processed_pseud ogene	-	-
ENSMUSG00000 082609	18.89	0.177	-2.501	8.66441E-05	0.00058137	Gm15464	-	processed_pseud ogene	-	-
ENSMUSG00000 082705	8.33	0.064	-3.957	1.86294E-06	2.53213E-05	Gm15616	-	processed_pseud ogene	-	-
ENSMUSG00000 082741	17.39	0.201	-2.314	7.70445E-06	8.08652E-05	Gm9703	-	processed_pseud ogene	-	-
ENSMUSG00000 082755	11.96	0.11	-3.178	2.04485E-06	2.72282E-05	Gm8692	-	processed_pseud ogene	-	-
ENSMUSG00000 082769	6.2	0.047	-4.406	2.90006E-06	3.64669E-05	Gm15793	-	processed_pseud ogene	-	-
ENSMUSG00000 082895	33.08	0.175	-2.512	6.86064E-10	3.71096E-08	Rpsa-ps9	-	processed_pseud ogene	-	-
ENSMUSG00000 082901	194.18	0.232	-2.107	1.6981E-11	1.62304E-09	Gm14323	-	processed_pseud ogene	-	-
ENSMUSG00000 082908	10.19	0.212	-2.238	0.001589464	0.006159388	Gm13736	-	processed_pseud ogene	-	-
ENSMUSG00000 082926	92.78	0.249	-2.005	2.05342E-10	1.29466E-08	Gm8172	-	processed_pseud ogene	-	-
ENSMUSG00000 082978	5.82	0.147	-2.765	0.001061108	0.004430076	Rpsa-ps11	-	processed_pseud ogene	-	-
ENSMUSG00000 083033	15.1	0.239	-2.066	0.000516447	0.0024622	Gm9083	-	processed_pseud ogene	-	-
ENSMUSG00000 083311	6.7	0.206	-2.279	0.002482963	0.008824439	Gm5643	-	processed_pseud ogene	-	-
ENSMUSG00000 083328	8.94	0.087	-3.521	9.89615E-06	9.86814E-05	Gm11826	-	processed_pseud ogene	-	-
ENSMUSG00000 083337	36.17	0.191	-2.392	1.50798E-09	7.38337E-08	Gm11539	-	processed_pseud ogene	-	-
ENSMUSG00000 083500	2.57	0.024	-5.382	0.000231696	0.001287445	Gm15470	-	processed_pseud ogene	-	-
ENSMUSG00000 083619	196.16	0.236	-2.082	3.08993E-11	2.61901E-09	Gm14414	-	processed_pseud ogene	-	-
ENSMUSG00000 083716	87.89	0.216	-2.211	2.02416E-08	6.09248E-07	Gm13436	-	processed_pseud ogene	-	-
ENSMUSG00000 083754	27.33	0.146	-2.779	1.0866E-07	2.39486E-06	Gm7803	-	processed_pseud ogene	-	-
ENSMUSG00000 083985	13.01	0.077	-3.699	2.86543E-08	7.99518E-07	Gm12468	-	processed_pseud ogene	-	-

ENSMUSG00000083992	57.81	0.114	-3.136	4.71823E-09	1.88404E-07	Gm11478	60S ribosomal protein L13a-like	processed_pseudogene	100504632	-
ENSMUSG00000084106	224.28	0.215	-2.219	9.51215E-11	6.92E-09	Gm6136	-	processed_pseudogene	-	-
ENSMUSG00000084166	4.05	0.015	-6.017	1.76646E-05	0.000160472	Gm6451	-	processed_pseudogene	-	-
ENSMUSG00000084314	11.33	0.192	-2.381	0.000230455	0.001282299	Rps15a-ps3	-	processed_pseudogene	-	-
ENSMUSG00000084349	245.37	0.211	-2.244	6.20405E-09	2.35191E-07	Rpl3-ps1	-	processed_pseudogene	-	-
ENSMUSG00000084416	202.05	0.209	-2.257	9.00267E-12	9.29707E-10	Rpl10a-ps1	-	processed_pseudogene	-	-
ENSMUSG00000084757	7.09	13.024	3.703	5.09095E-06	5.7752E-05	1700057H15Rik	-	lincRNA	-	-
ENSMUSG00000084787	4.84	4.334	2.116	0.004010793	0.013002563	Gm12436	predicted gene 12436	lincRNA	102635202	-
ENSMUSG00000084899	23.52	0.246	-2.025	6.59657E-05	0.000465203	Gm15344	-	processed_transcript	-	-
ENSMUSG00000084967	13.06	4.505	2.172	0.000315897	0.001650095	Gm12296	-	processed_transcript	-	-
ENSMUSG00000084970	23.96	8.167	3.03	6.85297E-10	3.71096E-08	1700060J05Rik	-	antisense_RNA	-	-
ENSMUSG00000084973	10.88	6.425	2.684	2.34555E-05	0.000202048	Gm13848	predicted gene 13848	lincRNA	434003	-
ENSMUSG00000084992	67.51	4.407	2.14	1.19299E-05	0.000115687	Gm13842	-	lincRNA	-	-
ENSMUSG00000085004	6.25	4.697	2.232	0.00215474	0.007885647	5430427M07Rik	RIKEN cDNA 5430427M07 gene	antisense_RNA	71349	-
ENSMUSG00000085089	8.15	0.098	-3.356	3.5871E-05	0.000285966	Arhgap15os	Rho GTPase activating protein 15, opposite strand	antisense_RNA	320872	-
ENSMUSG00000085111	58.25	0.188	-2.41	7.22461E-08	1.7059E-06	Ascl4	achaete-scute family bHLH transcription factor 4	protein_coding	67341	bHLH
ENSMUSG00000085162	8.04	4.901	2.293	0.001304617	0.005243271	Gm12295	predicted gene 12295	lincRNA	327946	-
ENSMUSG00000085213	16.44	0.24	-2.061	6.3691E-05	0.000451822	Gm13091	-	antisense_RNA	-	-
ENSMUSG00000085223	5.19	6.567	2.715	0.007855727	0.022342443	Gm11210	-	lincRNA	-	-
ENSMUSG00000085261	4.08	6.486	2.697	0.008479038	0.02376167	Gm13814	-	lincRNA	-	-
ENSMUSG00000085287	13.76	6.362	2.67	5.18194E-05	0.000382871	4833418N02Rik	RIKEN cDNA 4833418N02 gene	lincRNA	74597	-
ENSMUSG00000085297	206.78	9.813	3.295	0.001463813	0.005764464	Gm11651	predicted gene 11651	antisense_RNA	100379610	-
ENSMUSG00000085301	24.42	6.946	2.796	8.34825E-07	1.30345E-05	Gm12132	predicted gene 12132	lincRNA	102637264	-
ENSMUSG00000085323	6.8	4.436	2.149	0.004912446	0.015319637	9130410C08Rik	-	antisense_RNA	-	-

ENSMUSG00000085331	42.42	6.937	2.794	5.69651E-08	1.40799E-06	Gm11274	-	lincRNA	-	-
ENSMUSG00000085348	4.52	6.71	2.746	0.008237329	0.023196453	Myhas	-	antisense_RNA	-	-
ENSMUSG00000085385	13.25	0.238	-2.072	0.002699031	0.009424579	Snhg17	-	processed_transcript	-	-
ENSMUSG00000085402	36.72	0.196	-2.348	2.22495E-07	4.37426E-06	Gm12111	-	lincRNA	-	-
ENSMUSG00000085440	7.77	4.441	2.151	0.003529475	0.011718614	Sorbs2os	sorbin and SH3 domain containing 2, opposite strand	antisense_RNA	319940	-
ENSMUSG00000085456	10.16	4.434	2.149	0.001245176	0.005050693	Gm15398	-	lincRNA	-	-
ENSMUSG00000085498	6.3	6.579	2.718	0.000172742	0.001012474	Gm14023	predicted gene 14023	processed_transcript	100503468	-
ENSMUSG00000085510	5.68	4.363	2.125	0.015947841	0.039191295	Gm12082	-	lincRNA	-	-
ENSMUSG00000085515	5.1	5.573	2.478	0.000935797	0.003992245	C630028M04Rik	RIKEN cDNA C630028M04 gene	lincRNA	329844	-
ENSMUSG00000085516	22.58	0.177	-2.497	2.40821E-08	6.97953E-07	4930538E20Rik	RIKEN cDNA 4930538E20 gene	antisense_RNA	78200	-
ENSMUSG00000085522	9.8	4.697	2.232	0.006474483	0.019144311	4930556G01Rik	-	lincRNA	-	-
ENSMUSG00000085576	9.5	4.626	2.21	0.00185751	0.006984221	Dpy19l2	dpy-19-like 2 (C. elegans)	protein_coding	320752	-
ENSMUSG00000085720	97.6	4.587	2.198	7.12403E-09	2.61783E-07	Gm7854	predicted gene 7854	antisense_RNA	665934	-
ENSMUSG00000085721	5.14	6.505	2.702	0.00117241	0.004804982	Gm12796	-	lincRNA	-	-
ENSMUSG00000085731	7.47	0.232	-2.105	0.013366557	0.03403481	Gm16229	-	antisense_RNA	-	-
ENSMUSG00000085788	18.68	0.19	-2.397	3.51454E-06	4.27864E-05	4930470G03Rik	-	bidirectional_promoter_lncRNA	-	-
ENSMUSG00000085853	5.9	0.088	-3.508	0.000181044	0.001053832	Gm12167	-	antisense_RNA	-	-
ENSMUSG00000085887	17.29	0.154	-2.699	6.93025E-07	1.11887E-05	Arhgap27os3	Rho GTPase activating protein 27, opposite strand 3	antisense_RNA	653030	-
ENSMUSG00000085899	11.37	4.908	2.295	0.000280094	0.001494057	Gm15338	-	sense_intronic	-	-
ENSMUSG00000085911	5.93	4.647	2.216	0.00349505	0.011632257	Gm14061	-	lincRNA	-	-
ENSMUSG00000085931	22.14	4.433	2.148	1.19495E-06	1.75282E-05	Gm12648	-	lincRNA	-	-
ENSMUSG00000085945	9.67	4.647	2.216	0.007246303	0.020937259	2310014F06Rik	-	bidirectional_promoter_lncRNA	-	-
ENSMUSG00000085971	15.23	0.154	-2.698	6.53828E-06	7.04778E-05	Gm15411	-	processed_transcript	-	-
ENSMUSG00000085995	11.55	4.383	2.132	0.004293632	0.013764901	Gm2788	-	processed_transcript	-	-

ENSMUSG00000086020	21.28	4.106	2.038	6.78075E-05	0.000475579	Gm12239	-	antisense_RNA	-	-
ENSMUSG00000086026	8.42	4.158	2.056	0.001375118	0.0054717	Gm12374	predicted gene 12374	lincRNA	102636890	-
ENSMUSG00000086068	22.47	7.121	2.832	1.09364E-07	2.39913E-06	1700120G11Rik	-	lincRNA	-	-
ENSMUSG00000086123	5.64	0.096	-3.376	0.000190149	0.001096176	Gm16060	-	processed_pseudogene	-	-
ENSMUSG00000086146	14.08	4.036	2.013	0.001630958	0.006287428	Gm15729	-	antisense_RNA	-	-
ENSMUSG00000086199	60.22	5.81	2.538	2.75488E-05	0.000230673	Bcas3os1	breast carcinoma amplified sequence 3, opposite strand 1	antisense_RNA	71489	-
ENSMUSG00000086204	8.22	6.852	2.777	0.000584096	0.002722599	Gm15668	-	lincRNA	-	-
ENSMUSG00000086241	4.55	0.197	-2.347	0.018836041	0.044942878	4930483K19Rik	RIKEN cDNA 4930483K19 gene	antisense_RNA	74953	-
ENSMUSG00000086245	32.22	0.061	-4.035	4.16464E-16	1.33633E-13	Gm16170	-	antisense_RNA	-	-
ENSMUSG00000086249	22.25	4.203	2.071	1.77306E-05	0.000160991	Gm12724	-	processed_transcript	-	-
ENSMUSG00000086254	7.73	4.85	2.278	0.005229534	0.016132103	3100003L05Rik	RIKEN cDNA 3100003L05 gene	lincRNA	73076	-
ENSMUSG00000086266	4.83	4.795	2.261	0.002783705	0.009660177	Igf2os	insulin-like growth factor 2, opposite strand	antisense_RNA	111975	-
ENSMUSG00000086312	10.42	5.882	2.556	0.001203243	0.004906074	Gm15336	-	antisense_RNA	-	-
ENSMUSG00000086354	13.85	5.369	2.425	0.001156679	0.0047518	Gm13938	-	antisense_RNA	-	-
ENSMUSG00000086379	5.08	8.685	3.119	8.96074E-05	0.00059746	1700026D11Rik	RIKEN cDNA 1700026D11 gene	antisense_RNA	70371	-
ENSMUSG00000086404	4.15	11.402	3.511	0.002537939	0.008982515	Gm6787	proteasome (prosome, macropain) subunit, beta type 7 pseudogene	processed_transcript	627782	-
ENSMUSG00000086410	14.85	0.156	-2.682	1.15682E-05	0.000112727	Gm16158	-	antisense_RNA	-	-
ENSMUSG00000086427	4.17	8.199	3.035	0.011378466	0.030054736	Hoxa11os	homeobox A11, opposite strand	processed_transcript	15397	-
ENSMUSG00000086506	4.59	0.013	-6.266	5.61557E-06	6.26495E-05	Gm15911	-	antisense_RNA	-	-
ENSMUSG00000086509	15.14	5.099	2.35	0.000134008	0.000828913	Nkx2-2os	NK2 homeobox 2, opposite strand	antisense_RNA	100313531	-
ENSMUSG00000086583	585.79	0.204	-2.294	9.65719E-10	4.97221E-08	Gm15500	-	transcribed_processed_pseudogene	-	-
ENSMUSG00000086596	5.94	6.302	2.656	0.006852184	0.020023889	Susd5	sushi domain containing 5	protein_coding	382111	-
ENSMUSG00000086610	5.15	0.056	-4.154	2.18878E-05	0.000191109	Gm15408	predicted gene 15408	lincRNA	100503307	-
ENSMUSG00000086628	3.47	0.159	-2.651	0.005237307	0.016147763	Gm16157	predicted gene 16157	antisense_RNA	100504026	-

ENSMUSG00000086631	7.65	8.101	3.018	0.000219551	0.001232849	Gm12784	-	lincRNA	-	-
ENSMUSG00000086656	4.7	0.065	-3.951	0.000200238	0.001143334	Gm15701	-	antisense_RNA	-	-
ENSMUSG00000086718	17.63	0.221	-2.179	7.51533E-05	0.000517605	Gm16069	-	antisense_RNA	-	-
ENSMUSG00000086727	40.62	7.266	2.861	6.74019E-07	1.0921E-05	4931428L18Rik	RIKEN cDNA 4931428L18 gene	protein_coding	70988	-
ENSMUSG00000086745	15.98	4.373	2.129	0.013836849	0.034954919	Tspan2os	tetraspanin 2, opposite strand	antisense_RNA	78491	-
ENSMUSG00000086804	28.4	4.286	2.1	4.86536E-05	0.000365315	Gm43154	-	lincRNA	-	-
ENSMUSG00000086807	10.18	4.476	2.162	0.001060295	0.004429772	Platr21	pluripotency associated transcript 21	processed_transcript	78526	-
ENSMUSG00000086825	39.38	0.097	-3.369	8.4094E-12	8.7346E-10	Gm15675	-	lincRNA	-	-
ENSMUSG00000086899	11.71	10.491	3.391	0.000152571	0.000919916	1600002D24Rik	-	lincRNA	-	-
ENSMUSG00000086922	6.75	0.125	-2.994	0.00020206	0.001151541	Gm13835	-	processed_pseudogene	-	-
ENSMUSG00000086927	6.04	6.64	2.731	0.000170723	0.001003834	Gm11823	predicted gene 11823	lincRNA	329797	-
ENSMUSG00000087038	14.74	4.508	2.172	4.3546E-05	0.000333537	2900079G21Rik	RIKEN cDNA 2900079G21 gene	lincRNA	620760	-
ENSMUSG00000087059	6.94	9.557	3.257	5.13562E-05	0.000380387	Gm12339	-	processed_transcript	-	-
ENSMUSG00000087115	7.06	4.549	2.185	0.001496166	0.005859474	Pcsk2os2	proprotein convertase subtilisin/kexin type 2, opposite strand 2	antisense_RNA	378775	-
ENSMUSG00000087138	11.21	0.068	-3.886	5.56933E-07	9.39094E-06	Gm15545	predicted gene 15545	antisense_RNA	100502630	-
ENSMUSG00000087153	69.7	0.137	-2.872	2.78348E-14	6.41236E-12	Gm6483	-	unprocessed_pseudogene	-	-
ENSMUSG00000087170	9.44	6.545	2.71	0.001016729	0.004277594	1700001G01Rik	RIKEN cDNA 1700001G01 gene	lincRNA	75433	-
ENSMUSG00000087176	7.1	6.266	2.648	0.00158111	0.006133628	D230022J07Rik	-	processed_transcript	-	-
ENSMUSG00000087184	3.64	10.008	3.323	0.004439579	0.014121931	Gm11650	uncharacterized LOC108167931	processed_transcript	108167931	-
ENSMUSG00000087187	32.27	4.029	2.011	0.000466007	0.002266218	Gm13431	-	lincRNA	-	-
ENSMUSG00000087230	5.34	6.253	2.644	0.000323688	0.001682483	Mroh3	maestro heat-like repeat family member 3	protein_coding	76422	-
ENSMUSG00000087264	10.21	7.115	2.831	0.000138376	0.00085073	Gad1os	glutamate decarboxylase 1, opposite strand	antisense_RNA	68986	-
ENSMUSG00000087268	4.45	6.191	2.63	0.012142565	0.031644633	Gm14486	-	lincRNA	-	-
ENSMUSG00000087291	15.5	5.864	2.552	2.49371E-05	0.000212166	Gm11946	-	processed_transcript	-	-

ENSMUSG00000087306	14.41	5.338	2.416	0.003840161	0.012557572	A230004M16Rik	RIKEN cDNA A230004M16 gene	processed_transcript	102637814	-
ENSMUSG00000087354	5.98	0.194	-2.365	0.007685084	0.021954415	4930404I05Rik	RIKEN cDNA 4930404I05 gene	processed_transcript	67394	-
ENSMUSG00000087406	7.47	0.227	-2.142	0.003111913	0.010582505	E130215H24Rik	RIKEN cDNA E130215H24 gene	processed_transcript	109344	-
ENSMUSG00000087412	24.79	0.148	-2.758	2.62458E-08	7.47402E-07	Gm15501	-	transcribed_processed_pseudogene	-	-
ENSMUSG00000087459	4.89	9.634	3.268	0.003256394	0.010984447	Csmd2os	CUB and Sushi multiple domains 2, opposite strand	antisense_RNA	74989	-
ENSMUSG00000087486	13.77	0.151	-2.73	2.13988E-05	0.000187568	Gm11723	-	antisense_RNA	-	-
ENSMUSG00000087492	4.62	8.973	3.166	0.002627846	0.009226213	Gm15700	-	antisense_RNA	-	-
ENSMUSG00000087516	16.42	6.048	2.596	9.11228E-06	9.25599E-05	Tbx3os1	expressed sequence AW549542	antisense_RNA	100993	-
ENSMUSG00000087538	15.16	0.156	-2.677	1.96351E-06	2.63104E-05	Gm16341	-	antisense_RNA	-	-
ENSMUSG00000087541	7.75	0.138	-2.86	0.000626801	0.002880558	Hopxos	-	antisense_RNA	-	-
ENSMUSG00000087593	5.36	6.096	2.608	0.012061326	0.03146943	Gm16174	-	antisense_RNA	-	-
ENSMUSG00000087594	8.28	6.462	2.692	0.000255598	0.001390506	BC039771	-	processed_transcript	-	-
ENSMUSG00000087606	7.37	5.335	2.415	0.00240477	0.008609544	Gm16121	-	antisense_RNA	-	-
ENSMUSG00000087612	18.99	0.052	-4.254	1.69436E-09	8.20644E-08	A230005M16Rik	-	antisense_RNA	-	-
ENSMUSG00000087614	14.14	4.073	2.026	0.000473396	0.002293465	Gm12514	-	lincRNA	-	-
ENSMUSG00000087626	37.26	9.68	3.275	2.41744E-07	4.68598E-06	Gm15050	predicted gene 15050	antisense_RNA	100504154	-
ENSMUSG00000087658	45.41	4.436	2.149	8.35197E-05	0.000563773	Hotairm1	Hoxa transcript antisense RNA, myeloid-specific 1	antisense_RNA	105242658	-
ENSMUSG00000087691	48.39	0.088	-3.509	2.38504E-13	4.0816E-11	Gm15674	predicted gene 15674	antisense_RNA	102640368	-
ENSMUSG00000089635	5.74	4.176	2.062	0.012251851	0.031827891	Gm16559	-	antisense_RNA	-	-
ENSMUSG00000089656	12.08	12.064	3.593	2.9505E-05	0.000243236	Gm16271	-	antisense_RNA	-	-
ENSMUSG00000089671	4.18	4.513	2.174	0.020762252	0.048476728	Gm16537	-	antisense_RNA	-	-
ENSMUSG00000089683	21.01	0.238	-2.069	2.83765E-05	0.000235954	4930570N18Rik	-	antisense_RNA	-	-
ENSMUSG00000089715	21.01	0.204	-2.294	4.13327E-05	0.000319995	Cbx6	chromobox 6	protein_coding	494448	-
ENSMUSG00000089722	26.41	8.155	3.028	0.000174017	0.001018539	Cd300ld5	CD300 molecule like family member D5	protein_coding	100043125	-

ENSMUSG00000089776	7.49	9.615	3.265	0.000442895	0.00217978	Gm15684	-	antisense_RNA	-	-
ENSMUSG00000089787	8.14	4.32	2.111	0.002039285	0.0075337	Gm15825	predicted gene 15825	antisense_RNA	102635013	-
ENSMUSG00000089803	12.48	0.176	-2.506	0.000361555	0.001837568	Gm10171	-	processed_pseudogene	-	-
ENSMUSG00000089844	31.2	0.213	-2.228	3.62879E-05	0.000288011	A530032D15Rik	RIKEN cDNA A530032D15Rik gene	protein_coding	381287	-
ENSMUSG00000089857	9.5	0.238	-2.074	0.001677118	0.006435219	Zfp882	zinc finger protein 882	protein_coding	382019	zf-C2H2
ENSMUSG00000089922	5.16	9.758	3.287	0.000853188	0.003707602	Gm43517	-	protein_coding	-	-
ENSMUSG00000089964	8.16	4.844	2.276	0.002355193	0.008479356	Gm16582	-	processed_transcript	-	-
ENSMUSG00000089989	2958.55	0.205	-2.285	3.29827E-07	6.07227E-06	Gm45713	-	protein_coding	-	-
ENSMUSG00000089993	12.4	0.199	-2.329	0.00011054	0.000707872	Gm5822	-	processed_pseudogene	-	-
ENSMUSG00000089997	5.62	6.875	2.781	0.000603957	0.002794158	1810020O05Rik	Riken cDNA 1810020O05 gene	lincRNA	613264	-
ENSMUSG00000089998	15.67	0.171	-2.545	0.000111959	0.000715177	Phtf1os	putative homeodomain transcription factor 1, opposite strand	antisense_RNA	100125931	-
ENSMUSG00000090019	139.73	0.161	-2.631	3.51025E-09	1.47186E-07	Gimap1	GTPase, IMAP family member 1	protein_coding	16205	-
ENSMUSG00000090053	28.45	4.1	2.036	3.17274E-07	5.87137E-06	Palm2	paralemmin 2	protein_coding	242481	-
ENSMUSG00000090062	21.18	0.217	-2.203	3.22414E-05	0.000261674	Galnt6os	-	antisense_RNA	-	-
ENSMUSG00000090063	4.67	4.825	2.27	0.012212201	0.03175243	Dlx6os1	-	antisense_RNA	-	-
ENSMUSG00000090069	104.22	0.184	-2.442	1.14555E-09	5.77881E-08	E430024P14Rik	-	antisense_RNA	-	-
ENSMUSG00000090071	6.14	4.578	2.195	0.010832624	0.02895295	Cdk5r2	cyclin-dependent kinase 5, regulatory subunit 2 (p39)	protein_coding	12570	-
ENSMUSG00000090077	64.45	0.184	-2.441	4.85338E-09	1.92518E-07	Lime1	Lck interacting transmembrane adaptor 1	protein_coding	72699	-
ENSMUSG00000090084	8.33	7.42	2.891	0.000111201	0.000711597	Srpx	sushi-repeat-containing protein	protein_coding	51795	-
ENSMUSG00000090116	8.02	6.475	2.695	0.000297259	0.001569628	Gm15680	-	sense_overlapping	-	-
ENSMUSG00000090126	22.9	0.249	-2.005	1.0369E-05	0.000102656	4930519F09Rik	-	transcribed_processed_pseudogene	-	-
ENSMUSG00000090207	9.97	0.158	-2.659	8.70554E-05	0.000583895	4930524O07Rik	-	antisense_RNA	-	-
ENSMUSG00000090219	132.87	5.346	2.418	0.000344651	0.001769929	4930516K23Rik	olfactory receptor 643	protein_coding	259081	-
ENSMUSG00000090222	41.46	0.162	-2.628	3.12464E-09	1.34002E-07	Ifi203-ps	-	unprocessed_pseudogene	-	-

ENSMUSG00000090223	7.22	4.33	2.114	0.004384191	0.013990325	Pcp4	Purkinje cell protein 4	protein_coding	18546	-
ENSMUSG00000090291	9.24	6.957	2.799	0.000142148	0.000868795	Lrrc10b	leucine rich repeat containing 10B	protein_coding	278795	-
ENSMUSG00000090322	16.64	0.231	-2.113	0.000613273	0.002831424	Gm17090	-	antisense_RNA	-	-
ENSMUSG00000090336	31.02	6.278	2.65	9.96124E-09	3.45547E-07	4932443I19Rik	CFAP97 domain containing 2	protein_coding	403185	-
ENSMUSG00000090516	43.17	0.172	-2.543	1.96807E-10	1.24962E-08	Rps11-ps1	-	processed_pseudogene	-	-
ENSMUSG00000090608	28.29	0.243	-2.044	0.000101688	0.000662519	Gm17200	-	lincRNA	-	-
ENSMUSG00000090674	19.05	0.226	-2.143	0.000724354	0.003236984	Gm17082	-	processed_pseudogene	-	-
ENSMUSG00000090707	97.05	4.545	2.184	2.1498E-08	6.36133E-07	Gm8237	-	protein_coding	-	-
ENSMUSG00000090733	1442.08	0.216	-2.213	7.21845E-09	2.63635E-07	Rps27	ribosomal protein S27	protein_coding	57294	-
ENSMUSG00000090861	26.08	0.214	-2.224	5.6651E-06	6.30708E-05	Arf4os	-	antisense_RNA	-	-
ENSMUSG00000090877	57.16	11.197	3.485	1.42027E-12	1.9334E-10	Hspa1b	heat shock protein 1B	protein_coding	15511	-
ENSMUSG00000090958	15.79	4.451	2.154	0.000496485	0.002379343	Lrrc32	leucine rich repeat containing 32	protein_coding	434215	-
ENSMUSG00000090964	5.17	0.159	-2.652	0.005497888	0.016789862	Gm17206	-	antisense_RNA	-	-
ENSMUSG00000091055	12.25	6.508	2.702	9.66967E-05	0.000635764	Siglec15	sialic acid binding Ig-like lectin 15	protein_coding	620235	-
ENSMUSG00000091086	73.52	0.198	-2.34	1.77859E-12	2.29924E-10	Rpl6l	-	processed_pseudogene	-	-
ENSMUSG00000091144	22.71	0.175	-2.516	7.77536E-08	1.81258E-06	Phf11c	PHD finger protein 11C	protein_coding	628705	-
ENSMUSG00000091192	8.49	9.498	3.248	0.000204485	0.001162024	Sardhos	-	antisense_RNA	-	-
ENSMUSG00000091228	191.78	0.209	-2.258	6.47635E-09	2.41941E-07	Gm20390	-	protein_coding	-	-
ENSMUSG00000091275	118.53	4.835	2.273	8.12375E-08	1.86909E-06	Gm3248	-	protein_coding	-	-
ENSMUSG00000091345	8.52	4.322	2.112	0.010198693	0.027537237	Col6a5	collagen, type VI, alpha 5	protein_coding	665033	-
ENSMUSG00000091514	9.37	0.079	-3.653	1.1268E-05	0.000110341	Gm17484	-	lincRNA	-	-
ENSMUSG00000091562	4.85	5.04	2.334	0.00679868	0.019896658	Crybg3	-	processed_transcript	-	-
ENSMUSG00000091568	80.25	5.686	2.507	3.89736E-09	1.60255E-07	Gm8206	-	protein_coding	-	-
ENSMUSG00000091613	95.79	4.593	2.2	2.32261E-08	6.78616E-07	Gm17046	-	processed_pseudogene	-	-

ENSMUSG00000091625	10.99	0.117	-3.093	5.03498E-05	0.000374787	Lsm5	LSM5 homolog, U6 small nuclear RNA and mRNA degradation associated	protein_coding	66373	-
ENSMUSG00000091649	42.19	0.164	-2.611	1.04763E-08	3.58568E-07	Phf11b	PHD finger protein 11B	protein_coding	236451	-
ENSMUSG00000091655	7.65	0.187	-2.417	0.00118646	0.004849748	Gm8741	-	processed_pseudogene	-	-
ENSMUSG00000091722	14.57	4.418	2.144	0.000230498	0.001282299	Siah3	siah E3 ubiquitin protein ligase family member 3	protein_coding	380918	-
ENSMUSG00000091780	7.43	0.069	-3.866	7.92031E-05	0.000540212	Sco2	SCO2 cytochrome c oxidase assembly protein	protein_coding	100126824	-
ENSMUSG00000091849	43.15	4.297	2.103	1.53191E-06	2.15391E-05	Gm17188	-	processed_transcript	-	-
ENSMUSG00000091955	133.4	0.191	-2.385	1.19641E-12	1.67955E-10	Gm9844	-	protein_coding	-	-
ENSMUSG00000091971	50.08	11.508	3.525	5.64619E-10	3.11216E-08	Hspa1a	heat shock protein 1A	protein_coding	193740	-
ENSMUSG00000092008	4.58	12.617	3.657	0.001664278	0.006394144	Cyp2c69	cytochrome P450, family 2, subfamily c, polypeptide 69	protein_coding	100043108	-
ENSMUSG00000092035	46.05	5.21	2.381	7.35996E-08	1.73103E-06	Peg10	paternally expressed 10	protein_coding	170676	-
ENSMUSG00000092083	27.49	5.092	2.348	3.07308E-07	5.73628E-06	Kcnb2	potassium voltage gated channel, Shab-related subfamily, member 2	protein_coding	98741	-
ENSMUSG00000092086	5.42	0.205	-2.288	0.016320444	0.039932196	Gm6793	heterogeneous nuclear ribonucleoprotein A3 pseudogene	protein_coding	627828	-
ENSMUSG00000092247	5.56	5.156	2.366	0.002428367	0.008667331	Gm20426	-	transcribed_processed_pseudogene	-	-
ENSMUSG00000092457	6.58	0.127	-2.981	0.000722357	0.003230471	Gm8835	-	unprocessed_pseudogene	-	-
ENSMUSG00000092463	120.2	0.145	-2.785	2.12181E-13	3.70163E-11	Gm20489	-	protein_coding	-	-
ENSMUSG00000092586	92.93	6.192	2.63	2.21523E-09	1.01804E-07	Ly6g6c	lymphocyte antigen 6 complex, locus G6C	protein_coding	68468	-
ENSMUSG00000092593	12.01	4.425	2.146	0.001080297	0.004490367	Gm20492	predicted gene 20492	processed_transcript	102632786	-
ENSMUSG00000092609	50.08	11.508	3.525	5.64574E-10	3.11216E-08	Gm20481	-	protein_coding	-	-
ENSMUSG00000092622	10.41	4.243	2.085	0.00196424	0.007303006	Khdc3	KH domain containing 3, subcortical maternal complex member	protein_coding	66991	-
ENSMUSG00000092627	11.51	6.499	2.7	5.08366E-05	0.000377097	D130058E05Rik	-	processed_transcript	-	-
ENSMUSG00000092737	11.89	0.086	-3.54	0.000175018	0.001023061	Gm24022	-	miRNA	-	-
ENSMUSG00000092748	8.1	0.132	-2.919	0.000742487	0.003299968	Gm22175	-	miRNA	-	-
ENSMUSG00000093185	11.47	0.117	-3.09	0.000100418	0.000655916	Gm25692	-	miRNA	-	-

ENSMUSG00000093327	228.6	0.022	-5.484	4.37652E-35	1.31069E-31	Mir5107	microRNA 5107	miRNA	100628611	-
ENSMUSG00000093400	8.81	5.675	2.505	0.001341422	0.005361138	Gm20647	-	antisense_RNA	-	-
ENSMUSG00000093436	18.99	8.149	3.027	7.92632E-06	8.27323E-05	Gm20646	-	sense_overlapping	-	-
ENSMUSG00000093472	7.64	4.567	2.191	0.000810014	0.003554367	4930438E09Rik	RIKEN cDNA 4930438E09 gene	antisense_RNA	73977	-
ENSMUSG00000093497	5.35	6.158	2.623	0.001807217	0.006826547	Gm20713	-	processed_transcript	-	-
ENSMUSG00000093553	524.89	4.069	2.025	3.18216E-06	3.92721E-05	Gm20633	-	antisense_RNA	-	-
ENSMUSG00000093594	12.16	0.23	-2.117	0.002150658	0.007873914	Gm20707	-	lincRNA	-	-
ENSMUSG00000093650	13.87	6.39	2.676	8.54145E-06	8.83091E-05	Gm20631	-	antisense_RNA	-	-
ENSMUSG00000093656	29	4.37	2.128	5.68577E-08	1.40727E-06	Gm20628	-	antisense_RNA	-	-
ENSMUSG00000093662	14.71	5.286	2.402	0.000165921	0.000981381	Gm20649	-	lincRNA	-	-
ENSMUSG00000093675	8.28	4.789	2.26	0.014317723	0.035887176	Gm20618	-	lincRNA	-	-
ENSMUSG00000093686	24.77	0.244	-2.036	4.83756E-05	0.000364255	Gm4705	-	processed_pseudogene	-	-
ENSMUSG00000093695	10.62	4.938	2.304	0.001323326	0.005303041	Gm20717	-	processed_transcript	-	-
ENSMUSG00000093861	20.41	0.003	-8.33	1.35062E-14	3.32456E-12	Igkv1-110	-	IG_V_gene	-	-
ENSMUSG00000093930	12.52	0.079	-3.658	7.47006E-07	1.18892E-05	Hmgcs1	3-hydroxy-3-methylglutaryl-Coenzyme A synthase 1	protein_coding	208715	-
ENSMUSG00000093955	4.41	0.134	-2.896	0.007665795	0.021916696	Ighv1-34	-	IG_V_gene	-	-
ENSMUSG00000093973	19.44	5.257	2.394	0.00018416	0.001067818	Mrgpra2a	MAS-related GPR, member A2A	protein_coding	668727	-
ENSMUSG00000093989	244.89	4.096	2.034	2.72078E-09	1.21315E-07	Rnasek	ribonuclease, RNase K	protein_coding	52898	-
ENSMUSG00000094006	15.86	0.119	-3.076	3.411E-05	0.000274607	Igkv4-59	-	IG_V_gene	-	-
ENSMUSG00000094083	6.96	4.192	2.068	0.008208976	0.023131112	Gm1604a	predicted gene 1604b	protein_coding	381059	-
ENSMUSG00000094094	5.99	0.143	-2.807	0.002054572	0.007582379	Igkv5-45	-	IG_V_gene	-	-
ENSMUSG00000094117	11.14	0.211	-2.245	0.008161635	0.023030217	Igkv3-12	-	IG_V_gene	-	-
ENSMUSG00000094230	11.29	4.433	2.148	0.000112839	0.000719358	Gm21847	-	antisense_RNA	-	-
ENSMUSG00000094242	56.86	5.217	2.383	4.47677E-07	7.89432E-06	Gm5456	-	processed_pseudogene	-	-

ENSMUSG00000094315	2.19	0.03	-5.07	0.002133441	0.00782072	Igkv4-78	-	IG_V_gene	-	-
ENSMUSG00000094335	22.47	0.024	-5.4	3.07925E-14	6.83099E-12	Igkv1-117	-	IG_V_gene	-	-
ENSMUSG00000094356	4.84	0.013	-6.28	2.5833E-06	3.30152E-05	Igkv8-28	-	IG_V_gene	-	-
ENSMUSG00000094370	67.56	5.682	2.506	1.04332E-06	1.56228E-05	Gm3373	-	protein_coding	-	-
ENSMUSG00000094420	22.61	0.093	-3.42	3.51828E-07	6.42479E-06	Igkv10-96	-	IG_V_gene	-	-
ENSMUSG00000094433	14.46	0.174	-2.519	0.000234439	0.001298591	Igkv5-43	-	IG_V_gene	-	-
ENSMUSG00000094491	3.53	0.018	-5.791	0.000128637	0.000800926	Igkv1-133	-	IG_V_gene	-	-
ENSMUSG00000094509	5.07	0.177	-2.497	0.01174497	0.030809284	Ighv14-1	-	IG_V_gene	-	-
ENSMUSG00000094525	8.83	0.215	-2.215	0.001378751	0.005478719	Trbv12-2	-	TR_V_gene	-	-
ENSMUSG00000094546	6.12	0.095	-3.393	0.000764298	0.003377797	Ighv1-26	-	IG_V_gene	-	-
ENSMUSG00000094561	4.02	0.154	-2.697	0.018193848	0.04373582	Ighv1-22	-	IG_V_gene	-	-
ENSMUSG00000094568	7.41	0.147	-2.761	0.00045758	0.002237183	Smarce1-ps1	-	processed_pseudogene	-	-
ENSMUSG00000094619	2.48	0.026	-5.266	0.000962189	0.004087368	Trav14d-3-dv8	-	TR_V_gene	-	-
ENSMUSG00000094737	74.56	5.563	2.476	5.57735E-08	1.38425E-06	Gm17045	-	processed_pseudogene	-	-
ENSMUSG00000094796	55.97	0.157	-2.673	1.45921E-10	9.71134E-09	BC147527	cDNA sequence BC147527	protein_coding	625360	-
ENSMUSG00000094797	21.6	0.096	-3.38	2.69701E-07	5.14071E-06	Igkv6-15	-	IG_V_gene	-	-
ENSMUSG00000094814	4.08	6.019	2.59	0.005480645	0.01674005	Gm21973	-	protein_coding	-	-
ENSMUSG00000094872	6.21	0.047	-4.412	2.56632E-05	0.00021752	Igkv9-120	-	IG_V_gene	-	-
ENSMUSG00000094902	2.58	0.024	-5.353	0.000518731	0.002471564	Igkv10-95	-	IG_V_gene	-	-
ENSMUSG00000094930	13.21	0.078	-3.688	3.96455E-08	1.04763E-06	Igkv6-25	-	IG_V_gene	-	-
ENSMUSG00000095007	10.77	0.028	-5.16	1.11675E-07	2.43825E-06	Igkv12-41	-	IG_V_gene	-	-
ENSMUSG00000095041	73.68	0.219	-2.191	2.09662E-07	4.15504E-06	AC149090.1	-	protein_coding	-	-
ENSMUSG00000095079	230.46	0.199	-2.329	0.001928795	0.007201974	Igha	-	IG_C_gene	-	-
ENSMUSG00000095098	20.02	0.214	-2.227	6.5038E-05	0.000459744	Ccdc85b	coiled-coil domain containing 85B	protein_coding	240514	-

ENSMUSG00000095217	140.4	4.275	2.096	7.9263E-08	1.84015E-06	Hist1h2bn	histone cluster 1, H2bn	protein_coding	319187	-
ENSMUSG00000095335	6.57	0.009	-6.757	2.54229E-06	3.2607E-05	Igkv3-5	-	IG_V_gene	-	-
ENSMUSG00000095351	5.63	0.011	-6.534	2.09107E-06	2.78123E-05	Igkv3-2	-	IG_V_gene	-	-
ENSMUSG00000095403	100.13	0.18	-2.472	2.36712E-11	2.10568E-09	Gm21092	-	unprocessed_pseudogene	-	-
ENSMUSG00000095407	4.71	4.996	2.321	0.005357476	0.016430873	Tmem200c	transmembrane protein 200C	protein_coding	622645	-
ENSMUSG00000095478	8.05	0.067	-3.905	8.09696E-05	0.000549657	Gm9824	-	processed_pseudogene	-	-
ENSMUSG00000095493	6.55	5.637	2.495	0.001131305	0.004668905	A630023A22Rik	RIKEN cDNA A630023A22 gene	protein_coding	105518	-
ENSMUSG00000095497	3.05	0.021	-5.592	0.000169874	0.001000806	Igkv1-122	-	IG_V_gene	-	-
ENSMUSG00000095512	39.61	6.218	2.636	7.43305E-07	1.18619E-05	Gm17222	-	processed_pseudogene	-	-
ENSMUSG00000095562	143.88	0.24	-2.056	1.01674E-06	1.53141E-05	Gm21887	-	protein_coding	-	-
ENSMUSG00000095583	5.3	0.223	-2.163	0.020430659	0.04783303	Ighv14-2	-	IG_V_gene	-	-
ENSMUSG00000095612	6.28	0.139	-2.849	0.007166799	0.020747576	Ighv5-4	-	IG_V_gene	-	-
ENSMUSG00000095630	12.73	0.195	-2.358	0.000354311	0.001809724	Igkv6-23	-	IG_V_gene	-	-
ENSMUSG00000095633	3.47	0.017	-5.843	2.13716E-05	0.000187568	Igkv4-58	-	IG_V_gene	-	-
ENSMUSG00000095642	4.73	0.119	-3.074	0.000985097	0.004168916	Ighv14-3	-	IG_V_gene	-	-
ENSMUSG00000095672	4.06	0.136	-2.879	0.002578859	0.009086179	AC133103.5	-	protein_coding	-	-
ENSMUSG00000095753	3.95	0.016	-5.994	1.69698E-05	0.000155181	Igkv4-53	-	IG_V_gene	-	-
ENSMUSG00000095771	10.02	0.006	-7.31	3.0331E-09	1.31965E-07	Igkv14-111	-	IG_V_gene	-	-
ENSMUSG00000095794	17.65	0.016	-5.94	2.97912E-14	6.77617E-12	Igkv6-17	-	IG_V_gene	-	-
ENSMUSG00000095991	117.44	4.9	2.293	1.08754E-07	2.39486E-06	Gm17048	-	processed_pseudogene	-	-
ENSMUSG00000096003	62.7	5.96	2.575	1.02718E-06	1.54584E-05	Gm3500	predicted gene 3500	protein_coding	100041678	-
ENSMUSG00000096039	39.72	6.077	2.603	9.0292E-07	1.39386E-05	D830030K20Rik	RIKEN cDNA D830030K20 gene	protein_coding	320333	-
ENSMUSG00000096225	9.26	23.196	4.536	7.13455E-10	3.80418E-08	Lhx8	LIM homeobox protein 8	protein_coding	16875	Homeobox
ENSMUSG00000096336	12.09	0.005	-7.616	6.89903E-12	7.33542E-10	Igkv1-135	-	IG_V_gene	-	-

ENSMUSG00000 096401	103.74	0.167	-2.585	6.78756E-12	7.25986E-10	Gm21811	-	unprocessed_pse udogene	-	-
ENSMUSG00000 096474	9.77	0.054	-4.213	1.63742E-05	0.000150577	Gm5561	-	processed_pseud ogene	-	-
ENSMUSG00000 096490	9.36	0.032	-4.969	3.46716E-07	6.35081E-06	Igkv10-94	-	IG_V_gene	-	-
ENSMUSG00000 096544	59.73	0.202	-2.306	9.91742E-11	7.15467E-09	Gm4617	-	processed_pseud ogene	-	-
ENSMUSG00000 096579	49.72	5.456	2.448	1.48191E-07	3.11079E-06	Gm17121	-	processed_pseud ogene	-	-
ENSMUSG00000 096594	6.71	0.2	-2.32	0.003651483	0.012056871	Igkv8-19	-	IG_V_gene	-	-
ENSMUSG00000 096629	79.37	4.19	2.067	5.06253E-06	5.75387E-05	Gm3383	predicted gene 3383	protein_coding	100041 515	-
ENSMUSG00000 096632	4.05	0.015	-6.042	3.83035E-05	0.000300295	Igkv9-124	-	IG_V_gene	-	-
ENSMUSG00000 096715	13.02	0.022	-5.494	1.42593E-09	7.03917E-08	Igkv3-4	-	IG_V_gene	-	-
ENSMUSG00000 096719	33.07	5.88	2.556	1.45197E-08	4.62596E-07	Mrgpra2b	MAS-related GPR, member A2B	protein_coding	235712	-
ENSMUSG00000 096844	5.66	0.151	-2.725	0.001797355	0.006792149	Igkv6-14	-	IG_V_gene	-	-
ENSMUSG00000 096856	8.54	0.165	-2.602	0.002380071	0.008543246	Gm10778	predicted gene 10778	protein_coding	100233 208	zf-C2H2
ENSMUSG00000 096887	33.38	4.279	2.097	7.87611E-07	1.24037E-05	Gm20594	predicted gene, 20594	protein_coding	100463 512	-
ENSMUSG00000 096918	7.72	4.494	2.168	0.018588801	0.044474985	Gm16863	predicted gene, 16863	lincRNA	328646	-
ENSMUSG00000 097003	6.41	5.443	2.444	0.004746743	0.014893352	D930007P13R ik	Riken cDNA D930007P13 gene	lincRNA	432989	-
ENSMUSG00000 097009	34.62	4.008	2.003	4.00328E-06	4.7576E-05	Gm26861	-	antisense_RNA	-	-
ENSMUSG00000 097020	17.65	0.233	-2.101	2.57315E-05	0.000217893	Gm26613	-	lincRNA	-	-
ENSMUSG00000 097039	114.95	5.04	2.334	3.55024E-09	1.48062E-07	Pvt1	Pvt1 oncogene	lincRNA	19296	-
ENSMUSG00000 097042	11.87	0.133	-2.911	2.45759E-05	0.00020939	Gm17491	-	sense_intronic	-	-
ENSMUSG00000 097057	37.65	0.202	-2.307	4.3019E-08	1.10905E-06	Gm17638	-	lincRNA	-	-
ENSMUSG00000 097091	180.16	0.036	-4.799	8.51466E-30	1.9125E-26	4930526L06R ik	RIKEN cDNA 4930526L06 gene	antisense_RNA	75138	-
ENSMUSG00000 097138	11.26	5.41	2.436	0.000723942	0.003235947	Gm26625	-	lincRNA	-	-
ENSMUSG00000 097139	14.84	4.035	2.012	0.000104249	0.000676753	Gm26626	-	antisense_RNA	-	-
ENSMUSG00000 097140	7.29	0.179	-2.482	0.000891614	0.003840223	Gm26779	-	lincRNA	-	-

ENSMUSG00000097145	14.54	4.824	2.27	9.06161E-05	0.000602175	9230114K14Rik	RIKEN cDNA 9230114K14 gene	processed_transcript	414108	-
ENSMUSG00000097164	15.17	0.244	-2.037	0.000822663	0.003599324	Cep83os	-	bidirectional_promoter_lncRNA	-	-
ENSMUSG00000097176	16.49	0.185	-2.436	4.19452E-05	0.00032376	Gm26830	-	lincRNA	-	-
ENSMUSG00000097241	9.13	4.924	2.3	0.000531616	0.002518482	Gm26907	-	antisense_RNA	-	-
ENSMUSG00000097248	9.8	4.72	2.239	0.001480352	0.005811765	Gm2694	predicted gene 2694	lincRNA	100040294	-
ENSMUSG00000097250	6.99	4.311	2.108	0.01369128	0.034684706	Gm26771	-	antisense_RNA	-	-
ENSMUSG00000097252	1806.94	5.491	2.457	7.68686E-07	1.21482E-05	Gm6634	predicted gene 6634	lincRNA	625901	-
ENSMUSG00000097305	6.52	5.754	2.525	0.000271008	0.001453655	Gm17276	-	lincRNA	-	-
ENSMUSG00000097311	8.67	4.372	2.128	0.005305988	0.016303572	Gm26871	-	processed_transcript	-	-
ENSMUSG00000097325	6.27	7.228	2.854	0.00036758	0.00186215	Gm16897	predicted gene, 16897	antisense_RNA	320400	-
ENSMUSG00000097346	372.81	0.095	-3.389	1.11832E-12	1.63375E-10	Gm26619	-	lincRNA	-	-
ENSMUSG00000097352	12	0.135	-2.886	2.75794E-05	0.000230822	C920009B18Rik	RIKEN cDNA C920009B18 gene	transcribed_unprocessed_pseudogene	606736	-
ENSMUSG00000097353	10.12	0.227	-2.139	0.001167792	0.004791974	A430046D13Rik	Riken cDNA A430046D13 gene	lincRNA	654440	-
ENSMUSG00000097383	21.33	4.56	2.189	0.000124362	0.000777812	1500026H17Rik	RIKEN cDNA 1500026H17 gene	bidirectional_promoter_lncRNA	69002	-
ENSMUSG00000097384	199.32	45.633	5.512	3.56855E-05	0.000284866	Gm26815	-	lincRNA	-	-
ENSMUSG00000097407	16.2	0.173	-2.532	0.00086724	0.003750527	4933408J17Rik	RIKEN cDNA 4933408J17 gene	lincRNA	74454	-
ENSMUSG00000097412	78.94	0.214	-2.222	1.12263E-08	3.76354E-07	1810014B01Rik	RIKEN cDNA 1810014B01 gene	transcribed_unprocessed_pseudogene	66263	-
ENSMUSG00000097458	8.21	5.757	2.525	0.000410961	0.002044199	Gm26697	-	lincRNA	-	-
ENSMUSG00000097517	7.28	0.143	-2.803	0.000558071	0.002617586	Gm26695	-	lincRNA	-	-
ENSMUSG00000097520	9.45	4.965	2.312	0.002325628	0.00838804	4930488L21Rik	-	antisense_RNA	-	-
ENSMUSG00000097623	9.51	4.631	2.211	0.001387017	0.005505479	B230323A14Rik	RIKEN cDNA B230323A14 gene	antisense_RNA	319970	-
ENSMUSG00000097648	6.23	8.549	3.096	0.000986537	0.004174026	9330185C12Rik	-	lincRNA	-	-
ENSMUSG00000097675	46.27	4.529	2.179	1.2006E-07	2.59923E-06	1700101I11Rik	RIKEN cDNA 1700101I11 gene	processed_transcript	100504641	-

ENSMUSG00000097718	4.71	0.246	-2.024	0.014900799	0.037131113	Gm26896	-	lincRNA	-	-
ENSMUSG00000097722	15.22	0.245	-2.031	0.000159261	0.000948857	Gm26841	-	lincRNA	-	-
ENSMUSG00000097727	9.53	9.386	3.23	3.49911E-05	0.000280819	F630040K05Rik	RIKEN cDNA F630040K05 gene	lincRNA	433111	-
ENSMUSG00000097739	7.54	5.36	2.422	0.017750332	0.042835846	Gm2539	-	transcribed_unprocessed_pseudogene	-	-
ENSMUSG00000097763	15.58	0.226	-2.147	0.000925593	0.003953408	Gm26636	-	antisense_RNA	-	-
ENSMUSG00000097770	10.57	4.485	2.165	0.000178959	0.001042032	Gm26776	-	lincRNA	-	-
ENSMUSG00000097772	7.83	0.2	-2.322	0.00999999	0.027106626	5430416N02Rik	RIKEN cDNA 5430416N02 gene	lincRNA	100503199	-
ENSMUSG00000097797	4.9	4.876	2.286	0.00827027	0.023274626	Gm26901	-	lincRNA	-	-
ENSMUSG00000097804	6.37	4.182	2.064	0.010284941	0.027736772	Gm16685	-	antisense_RNA	-	-
ENSMUSG00000097836	14.17	4.575	2.194	0.000181597	0.001056026	Gm26903	-	antisense_RNA	-	-
ENSMUSG00000097855	188.79	34.229	5.097	2.09804E-06	2.78844E-05	A930007I19Rik	RIKEN cDNA A930007I19 gene	lincRNA	77779	-
ENSMUSG00000097864	4.26	0.135	-2.889	0.002695472	0.009420345	Gm26856	-	lincRNA	-	-
ENSMUSG00000097881	8.95	11.277	3.495	3.38119E-06	4.12749E-05	Celrr	cerebellum expressed regulatory RNA	lincRNA	98452	-
ENSMUSG00000098107	25.11	5.109	2.353	3.73582E-06	4.5053E-05	Gm27007	-	lincRNA	-	-
ENSMUSG00000098120	35.84	0.161	-2.635	3.18313E-09	1.36084E-07	Gm5914	predicted gene 5914	lincRNA	546100	-
ENSMUSG00000098129	7.63	7.643	2.934	2.9196E-05	0.000241428	Gm27040	-	antisense_RNA	-	-
ENSMUSG00000098132	6.12	5.698	2.51	0.001065736	0.004443204	Rassf10	Ras association (RalGDS/AF-6) domain family (N-terminal) member 10	protein_coding	78748	-
ENSMUSG00000098134	13.16	0.239	-2.064	0.001517466	0.005931554	Rnf113a2	ring finger protein 113A2	protein_coding	66381	-
ENSMUSG00000098144	28.99	4.735	2.243	5.18149E-08	1.29494E-06	1700029N11Rik	RIKEN cDNA 1700029N11 gene	lincRNA	69491	-
ENSMUSG00000098178	197.02	0.143	-2.805	2.48175E-11	2.18601E-09	Gm42418	-	lincRNA	-	-
ENSMUSG00000098197	63.97	0.077	-3.691	2.75269E-12	3.3421E-10	BC051537	cDNA sequence BC051537	antisense_RNA	414076	-
ENSMUSG00000098243	8.79	5.045	2.335	0.003983699	0.012932807	Gm4258	-	processed_transcript	-	-
ENSMUSG00000098257	12.88	5.9	2.561	0.00061712	0.002844801	Gm27169	-	lincRNA	-	-

ENSMUSG00000098269	13.71	0.156	-2.677	2.76557E-05	0.000231245	Mir8094	microRNA 8094	miRNA	102465886	-
ENSMUSG00000098359	3.02	0.101	-3.305	0.002406114	0.008611109	Mir7649	microRNA 7649	miRNA	102465757	-
ENSMUSG00000098537	20.2	5.564	2.476	8.97152E-06	9.15441E-05	Gm27867	-	misc_RNA	-	-
ENSMUSG00000098566	39.52	5.03	2.331	8.73226E-07	1.35267E-05	Gm27761	-	misc_RNA	-	-
ENSMUSG00000098627	7.4	4.071	2.025	0.01622469	0.039746619	4930524O08Rik	RIKEN cDNA 4930524O08 gene	lincRNA	74700	-
ENSMUSG00000098715	223.2	0.207	-2.271	1.19936E-11	1.17212E-09	Gm28053	-	protein_coding	-	-
ENSMUSG00000098739	73.43	0.178	-2.491	2.41512E-10	1.48114E-08	Gm27151	-	antisense_RNA	-	-
ENSMUSG00000098749	8.69	5.56	2.475	0.001390006	0.005515823	Gm27477	-	misc_RNA	-	-
ENSMUSG00000098760	18.51	4.888	2.289	3.01852E-05	0.000247332	Gm2164	-	antisense_RNA	-	-
ENSMUSG00000098814	17.7	0.122	-3.033	2.39328E-05	0.000205274	Igkv19-93	-	IG_V_gene	-	-
ENSMUSG00000098889	7.01	0.234	-2.097	0.005511733	0.016820708	Gm27206	-	antisense_RNA	-	-
ENSMUSG00000098914	7.29	4.222	2.078	0.009771162	0.026622898	Gm27747	-	misc_RNA	-	-
ENSMUSG00000098915	40.55	0.152	-2.722	1.44273E-09	7.10256E-08	Rpl15-ps2	-	processed_pseudogene	-	-
ENSMUSG00000099009	4.24	6.86	2.778	0.005235904	0.016146208	Rdh16f1	-	protein_coding	-	-
ENSMUSG00000099061	11.97	6.084	2.605	4.11381E-05	0.000318979	Gm28050	-	lincRNA	-	-
ENSMUSG00000099070	11.96	0.111	-3.176	1.13885E-05	0.000111399	Mir8119	microRNA 8119	miRNA	102465997	-
ENSMUSG00000099117	10.98	4.337	2.117	0.00106237	0.004433284	Gm27404	-	misc_RNA	-	-
ENSMUSG00000099338	18.43	4.173	2.061	0.000744418	0.00330691	2810030D12Rik	-	bidirectional_promoter_lncRNA	-	-
ENSMUSG00000099474	7.33	0.189	-2.401	0.013840829	0.034958548	1700097N02Rik	RIKEN cDNA 1700097N02 gene	antisense_RNA	67522	-
ENSMUSG00000099492	34.25	0.167	-2.581	1.80825E-08	5.53533E-07	Gm5525	-	processed_pseudogene	-	-
ENSMUSG00000099564	7.17	6.144	2.619	0.001458108	0.005747036	Gm28729	predicted gene 28729	protein_coding	102635744	-
ENSMUSG00000099576	5.76	4.752	2.249	0.009914717	0.026937277	Gm29040	-	lincRNA	-	-
ENSMUSG00000099632	10.05	7.194	2.847	0.000169	0.000996642	2900093K20Rik	-	antisense_RNA	-	-
ENSMUSG00000099757	221.2	0.176	-2.506	7.28762E-12	7.65797E-10	BE692007	-	processed_transcript	-	-

ENSMUSG00000 099853	8.74	7.719	2.948	2.31513E-06	3.02329E-05	Gm29328	-	lincRNA	-	-
ENSMUSG00000 099858	51.22	8.99	3.168	1.20019E-10	8.31141E-09	Gm6652	-	processed_pseud ogene	-	-
ENSMUSG00000 099911	6.64	5.63	2.493	0.003366675	0.011286528	1700044C05R ik	RIKEN cDNA 1700044C05 gene	lincRNA	73306	-
ENSMUSG00000 099930	5.4	8.313	3.055	3.92163E-05	0.000306116	Gm2396	uncharacterized LOC105245043	processed_transc ript	105245 043	-
ENSMUSG00000 100005	9.2	4.222	2.078	0.008042245	0.022764825	B130024G19 Rik	RIKEN cDNA B130024G19 gene	processed_transc ript	434198	-
ENSMUSG00000 100113	6.02	7.537	2.914	0.000679992	0.003073131	Gm28592	-	lincRNA	-	-
ENSMUSG00000 100157	50.51	6.193	2.631	0.0001387	0.000851484	2310034O05R ik	RIKEN cDNA 2310034O05 gene	lincRNA	69571	-
ENSMUSG00000 100199	15.24	0.197	-2.346	2.21465E-05	0.000192806	Gm20324	predicted gene, 20324	antisense_RNA	100504 637	-
ENSMUSG00000 100345	4.77	4.678	2.226	0.015681619	0.038701141	Gm29418	-	lincRNA	-	-
ENSMUSG00000 100371	7.06	4.504	2.171	0.007686819	0.021955881	Gm29669	-	lincRNA	-	-
ENSMUSG00000 100405	7.44	0.199	-2.328	0.008468052	0.023738289	Gm28911	-	processed_pseud ogene	-	-
ENSMUSG00000 100486	11.99	6.401	2.678	0.000138454	0.000850851	Gm4131	predicted gene 4131	protein_coding	100042 960	-
ENSMUSG00000 100580	23.07	5.058	2.339	7.8038E-08	1.81641E-06	4933436I20Ri k	-	lincRNA	-	-
ENSMUSG00000 100583	7.58	6.172	2.626	0.009282166	0.025542302	Gm29443	predicted gene 29443	antisense_RNA	102634 559	-
ENSMUSG00000 100600	10.01	4.664	2.222	0.000862233	0.003737869	A230077H06 Rik	RIKEN cDNA A230077H06 gene	antisense_RNA	320210	-
ENSMUSG00000 100632	11.66	0.087	-3.522	1.1825E-06	1.73598E-05	9430060I03Ri k	RIKEN cDNA 9430060I03 gene	antisense_RNA	100037 260	-
ENSMUSG00000 100650	6.58	4.634	2.212	0.01597378	0.039244307	Gm28807	-	lincRNA	-	-
ENSMUSG00000 100658	37.29	0.23	-2.119	0.003057327	0.0104344	F730311O21R ik	RIKEN cDNA F730311O21 gene	lincRNA	622304	-
ENSMUSG00000 100755	169.49	0.227	-2.138	2.50645E-10	1.51645E-08	Rps23-ps1	-	processed_pseud ogene	-	-
ENSMUSG00000 100807	6.08	4.001	2	0.003415193	0.011408738	Gm29521	-	processed_transc ript	-	-
ENSMUSG00000 100975	5.5	0.155	-2.686	0.001861019	0.006994487	Gm28875	-	lincRNA	-	-
ENSMUSG00000 101014	13.59	0.216	-2.209	0.000212808	0.001200235	Gm28289	-	antisense_RNA	-	-
ENSMUSG00000 101029	18.14	0.029	-5.117	3.81542E-13	6.28984E-11	Gm29439	-	antisense_RNA	-	-
ENSMUSG00000 101102	48.72	4.038	2.014	4.27541E-06	5.01651E-05	Gm28609	-	antisense_RNA	-	-

ENSMUSG00000101132	8.33	7.347	2.877	0.001076166	0.004477339	Gm8000	-	unprocessed_pseudogene	-	-
ENSMUSG00000101162	849.49	5.138	2.361	2.20427E-08	6.48258E-07	Gm26728	-	processed_transcript	-	-
ENSMUSG00000101166	31.8	0.152	-2.715	1.65137E-09	8.0416E-08	Gm28496	-	processed_transcript	-	-
ENSMUSG00000101179	7.12	7.801	2.964	0.002788787	0.009674077	Gm29455	-	lincRNA	-	-
ENSMUSG00000101298	58.85	4.514	2.174	2.07063E-07	4.11583E-06	Gm28308	-	protein_coding	-	-
ENSMUSG00000101299	6.3	4.571	2.193	0.00206761	0.007616417	Gm28175	-	lincRNA	-	-
ENSMUSG00000101308	250.96	5.203	2.379	2.26358E-10	1.40256E-08	Gm28989	-	antisense_RNA	-	-
ENSMUSG00000101621	50.94	5.172	2.371	2.83396E-10	1.67511E-08	Gm28905	-	lincRNA	-	-
ENSMUSG00000101640	20.85	4.051	2.018	0.002155508	0.007886851	Gm28376	-	antisense_RNA	-	-
ENSMUSG00000101795	16.48	0.126	-2.991	1.59853E-07	3.30161E-06	Gm5835	-	processed_pseudogene	-	-
ENSMUSG00000101840	27.89	5.324	2.413	1.8216E-05	0.000164733	Gm28294	-	antisense_RNA	-	-
ENSMUSG00000101848	5.37	9.393	3.232	0.00851183	0.023827553	4933417E11Rik	RIKEN cDNA 4933417E11 gene	lincRNA	71134	-
ENSMUSG00000101854	25.48	8.132	3.024	3.00761E-06	3.74524E-05	1700026F02Rik	RIKEN cDNA 1700026F02 gene	lincRNA	69419	-
ENSMUSG00000101892	11.52	0.236	-2.081	0.000618067	0.002848054	9130401M01Rik	RIKEN cDNA 9130401M01 gene	protein_coding	75758	-
ENSMUSG00000101895	10.5	4.132	2.047	0.004616855	0.014531487	Gm28981	-	lincRNA	-	-
ENSMUSG00000102106	13.91	4.44	2.151	0.009094517	0.025139159	2310043O21Rik	RIKEN cDNA 2310043O21 gene	lincRNA	69679	-
ENSMUSG00000102117	15	0.067	-3.898	4.23536E-08	1.0982E-06	Rpsa-ps1	-	processed_pseudogene	-	-
ENSMUSG00000102312	32.12	4.232	2.081	2.0188E-07	4.03962E-06	Pcdha3	protocadherin alpha 3	protein_coding	192163	-
ENSMUSG00000102483	17.05	4.922	2.299	0.002252957	0.008171857	Catspere1	cation channel sperm associated auxiliary subunit epsilon 1	unprocessed_pseudogene	631584	-
ENSMUSG00000102564	42.05	4.747	2.247	0.000251689	0.001374648	Gm37035	-	antisense_RNA	-	-
ENSMUSG00000102595	7.22	4.314	2.109	0.002980943	0.010218346	Gm30097	-	lincRNA	-	-
ENSMUSG00000102697	5.76	7.213	2.851	0.003557811	0.011799612	Pcdhac2	protocadherin alpha subfamily C, 2	protein_coding	353237	-
ENSMUSG00000102802	9.66	4.71	2.236	0.001170465	0.004798559	Mgam2-ps	-	transcribed_unprocessed_pseudogene	-	-

ENSMUSG00000102817	9.34	9.888	3.306	2.83341E-05	0.00023575	Gm6185	-	transcribed_unprocessed_pseudogene	-	-
ENSMUSG00000102820	8.71	4.023	2.008	0.006456883	0.019104846	Gm38353	-	lincRNA	-	-
ENSMUSG00000102824	3.05	0.194	-2.365	0.021281637	0.049477259	Pdcd5-ps	-	processed_pseudogene	-	-
ENSMUSG00000102886	15.08	5.007	2.324	0.000107427	0.000692627	Gm30667	-	lincRNA	-	-
ENSMUSG00000102912	2.85	0.022	-5.503	0.000203745	0.001158572	Gm20731	-	TEC	-	-
ENSMUSG00000103126	9.7	0.249	-2.004	0.012729354	0.032780616	Gm37387	-	lincRNA	-	-
ENSMUSG00000103286	124.03	0.133	-2.91	1.23946E-18	5.71651E-16	Gm5850	-	processed_pseudogene	-	-
ENSMUSG00000103297	4.95	0.013	-6.27	0.000124889	0.000780838	Ighv3-2	-	IG_V_pseudogene	-	-
ENSMUSG00000103367	190.55	5.687	2.508	3.28864E-09	1.38717E-07	Gm38158	-	processed_pseudogene	-	-
ENSMUSG00000103442	34.33	4.602	2.202	1.41794E-08	4.54981E-07	Pcdha1	protocadherin alpha 1	protein_coding	116731	-
ENSMUSG00000103529	41.44	5.471	2.452	3.01494E-07	5.66689E-06	A730089K16Rik	-	TEC	-	-
ENSMUSG00000103586	16.6	0.174	-2.524	1.76349E-05	0.000160284	5830405F06Rik	-	lincRNA	-	-
ENSMUSG00000103735	27.23	5.347	2.419	1.50613E-06	2.1243E-05	Gm38317	-	processed_pseudogene	-	-
ENSMUSG00000103865	14.33	5.867	2.553	2.79689E-05	0.000233598	Gm37416	-	TEC	-	-
ENSMUSG00000103927	12.19	8.026	3.005	0.001324142	0.005305132	Gm9932	-	lincRNA	-	-
ENSMUSG00000103955	23.52	4.584	2.197	1.9302E-05	0.000172642	Gm37270	-	TEC	-	-
ENSMUSG00000103965	3.7	4.393	2.135	0.02034503	0.047663605	Gm30173	-	lincRNA	-	-
ENSMUSG00000104028	124.03	0.133	-2.91	1.24071E-18	5.71651E-16	Gm5851	-	processed_pseudogene	-	-
ENSMUSG00000104148	33.67	4.493	2.168	2.42395E-08	7.00255E-07	Pcdha2	protocadherin alpha 2	protein_coding	353234	-
ENSMUSG00000104211	7.57	5.12	2.356	0.004583263	0.014438404	Gm37985	-	TEC	-	-
ENSMUSG00000104213	438.98	0.004	-7.88	3.37929E-45	3.03612E-41	Ighd	-	IG_C_gene	-	-
ENSMUSG00000104245	9.71	0.224	-2.161	0.002530644	0.008960227	Gm38155	-	antisense_RNA	-	-
ENSMUSG00000104309	4.38	0.014	-6.116	2.3562E-05	0.000202674	Gm5846	-	processed_pseudogene	-	-
ENSMUSG00000104350	10.98	0.201	-2.313	0.001261674	0.005106084	Gm38244	-	TEC	-	-

ENSMUSG00000 104413	9.28	0.164	-2.605	0.000563539	0.002638414	Gm37065	-	antisense_RNA	-	-
ENSMUSG00000 104448	5.46	4.608	2.204	0.001650908	0.006349566	Gm37256	-	lincRNA	-	-
ENSMUSG00000 104452	3.8	0.017	-5.886	7.69674E-05	0.00052747	Ighv8-8	-	IG_V_gene	-	-
ENSMUSG00000 104531	5.11	6.399	2.678	0.001361808	0.005428669	Gm43731	-	lincRNA	-	-
ENSMUSG00000 104594	11.65	0.088	-3.508	0.000208443	0.001180806	Gm24022	-	miRNA	-	-
ENSMUSG00000 104699	3.36	0.094	-3.41	0.006219438	0.018567383	Rps4x-ps	-	processed_pseud ogene	-	-
ENSMUSG00000 104748	5.86	5.212	2.382	0.008636747	0.024109633	Gm43592	-	TEC	-	-
ENSMUSG00000 104785	11.09	4.836	2.274	0.000170949	0.001003848	Gm31121	predicted gene, 31121	lincRNA	102633 249	-
ENSMUSG00000 104791	4.39	6.075	2.603	0.00242822	0.008667331	4930511M18 Rik	-	sense_overlappin g	-	-
ENSMUSG00000 104795	4.98	4.082	2.029	0.017102878	0.041529947	Gm42783	-	TEC	-	-
ENSMUSG00000 104818	51.53	4.264	2.092	3.00073E-06	3.73926E-05	Gm43661	-	lincRNA	-	-
ENSMUSG00000 104927	20.6	0.06	-4.057	1.89448E-12	2.41804E-10	Gm43388	-	lincRNA	-	-
ENSMUSG00000 104975	55.36	0.014	-6.2	1.0798E-26	1.14134E-23	Iglj2	-	IG_J_gene	-	-
ENSMUSG00000 105039	15.41	4.867	2.283	0.000867472	0.003750627	Gm32585	-	lincRNA	-	-
ENSMUSG00000 105128	10.36	0.051	-4.292	1.59276E-07	3.29347E-06	Gm42870	-	TEC	-	-
ENSMUSG00000 105140	11.5	0.114	-3.132	2.70732E-06	3.42832E-05	Gm43127	-	TEC	-	-
ENSMUSG00000 105157	10.84	6.75	2.755	0.000342806	0.001763251	Gm31831	-	lincRNA	-	-
ENSMUSG00000 105160	7.8	0.008	-7.012	1.23443E-07	2.65328E-06	A530030E21 Rik	-	TEC	-	-
ENSMUSG00000 105195	6.55	4.118	2.042	0.006675852	0.019620279	Gm43584	-	processed_transc ript	-	-
ENSMUSG00000 105231	11.1	0.006	-7.457	1.20261E-10	8.31141E-09	Iglj3	-	IG_J_gene	-	-
ENSMUSG00000 105233	5.94	0.186	-2.426	0.004559221	0.014377789	Gm20568	-	processed_pseud ogene	-	-
ENSMUSG00000 105255	13.75	0.221	-2.178	0.00030722	0.001614161	Gm42413	-	antisense_RNA	-	-
ENSMUSG00000 105324	7.19	5.874	2.554	0.004334671	0.013863107	Gm42705	-	lincRNA	-	-
ENSMUSG00000 105361	11.75	4.536	2.182	0.001377888	0.005476692	AY036118	-	lincRNA	-	-

ENSMUSG00000105388	21.49	0.134	-2.903	6.62082E-07	1.07958E-05	Rpl36a-ps2	-	processed_pseudogene	-	-
ENSMUSG00000105518	56.83	0.2	-2.324	1.02905E-07	2.29669E-06	Gm42674	-	protein_coding	-	-
ENSMUSG00000105547	67.46	0.014	-6.125	1.1644E-27	1.60947E-24	Iglc3	-	IG_C_gene	-	-
ENSMUSG00000105646	1071.78	0.077	-3.693	1.26795E-06	1.83592E-05	Gm30211	-	lincRNA	-	-
ENSMUSG00000105797	5.78	8.769	3.132	0.000814657	0.003569511	Gm5149	-	lincRNA	-	-
ENSMUSG00000105827	17.03	6.047	2.596	5.39806E-06	6.07374E-05	Hist2h2bb	histone cluster 2, H2bb	protein_coding	319189	-
ENSMUSG00000105866	130.66	0.184	-2.444	2.47521E-05	0.000210792	Gm6745	-	processed_pseudogene	-	-
ENSMUSG00000105891	16.46	4.127	2.045	0.001892071	0.007088955	A230001M10Rik	RIKEN cDNA A230001M10 gene	lincRNA	319951	-
ENSMUSG00000105906	25.7	0.088	-3.498	1.91171E-12	2.41912E-10	Iglc1	-	IG_C_gene	-	-
ENSMUSG00000105913	529.52	0.174	-2.52	7.06252E-10	3.78825E-08	Mir5121	microRNA 5121	miRNA	100628629	-
ENSMUSG00000105914	7.96	0.136	-2.88	0.000121832	0.00076465	Gm19243	-	processed_pseudogene	-	-
ENSMUSG00000106019	65.76	4.797	2.262	9.72676E-08	2.18535E-06	Gm43672	-	lincRNA	-	-
ENSMUSG00000106053	21.27	8.131	3.023	4.26832E-08	1.10356E-06	Gm20752	predicted gene, 20752	lincRNA	624759	-
ENSMUSG00000106106	209.48	0.191	-2.387	4.48449E-10	2.53402E-08	CT010467.1	-	rRNA	-	-
ENSMUSG00000106139	4.43	6.568	2.715	0.011162174	0.029618001	Gm30648	-	lincRNA	-	-
ENSMUSG00000106319	8.52	4.62	2.208	0.000775456	0.003417752	Gm42601	predicted gene, 33100	lincRNA	102635874	-
ENSMUSG00000106321	6.92	25.658	4.681	1.84583E-06	2.5146E-05	Gm43674	-	lincRNA	-	-
ENSMUSG00000106350	11.89	4.783	2.258	0.000520174	0.002477343	Gm4869	predicted gene 4869	protein_coding	101055939	-
ENSMUSG00000106423	5.04	4.516	2.175	0.003954194	0.012855601	Gm42825	-	lincRNA	-	-
ENSMUSG00000106567	116.77	0.059	-4.074	1.91763E-23	1.56627E-20	2010309G21Rik	-	antisense_RNA	-	-
ENSMUSG00000106609	12.84	4.654	2.218	0.001116264	0.004619564	Gm43181	-	TEC	-	-
ENSMUSG00000106666	1.9	0.034	-4.881	0.002574021	0.009073688	Gm43587	-	processed_pseudogene	-	-
ENSMUSG00000106668	5.85	0.011	-6.528	9.44122E-07	1.44382E-05	Iglj1	-	IG_J_gene	-	-
ENSMUSG00000106702	26.38	0.218	-2.2	2.0371E-07	4.0669E-06	Gm42846	-	antisense_RNA	-	-

ENSMUSG00000106737	125.43	5.674	2.504	1.09054E-12	1.60622E-10	Gm42962	-	antisense_RNA	-	-
ENSMUSG00000106741	7.34	5.14	2.362	0.000665621	0.003018816	4930513D17Rik	RIKEN cDNA 4930513D17 gene	lincRNA	75095	-
ENSMUSG00000106752	62.1	5.985	2.581	9.2446E-07	1.42101E-05	Gm3348	-	processed_pseudogene	-	-
ENSMUSG00000106767	4.44	0.127	-2.974	0.001475769	0.005798841	Gm42727	-	TEC	-	-
ENSMUSG00000106788	12.58	4.196	2.069	0.000152636	0.000919916	Gm43449	-	antisense_RNA	-	-
ENSMUSG00000106794	5.66	4.151	2.053	0.005474977	0.016728092	Gm40293	-	lincRNA	-	-
ENSMUSG00000106863	7.02	4.611	2.205	0.007913906	0.02246879	Gm42109	-	processed_transcript	-	-
ENSMUSG00000107071	25.08	0.239	-2.063	0.000167367	0.000988306	Gm42420	-	protein_coding	-	-
ENSMUSG00000107096	4.91	0.11	-3.185	0.000438333	0.002161472	Gm43597	-	lincRNA	-	-
ENSMUSG00000107157	66.89	5.741	2.521	8.50554E-07	1.32326E-05	Gm3170	-	processed_pseudogene	-	-
ENSMUSG00000107191	16.95	0.164	-2.611	2.0876E-05	0.000184244	Gm43579	-	antisense_RNA	-	-
ENSMUSG00000107192	8.82	6.328	2.662	0.00307091	0.010470814	Gm19583	predicted gene, 19583	lincRNA	100503184	-
ENSMUSG00000107230	7.1	0.193	-2.374	0.001286839	0.005185738	Gm19265	-	antisense_RNA	-	-
ENSMUSG00000107314	6.42	4.542	2.183	0.012399854	0.032147306	Gm20488	-	processed_transcript	-	-
ENSMUSG00000107368	5.54	0.211	-2.246	0.009140917	0.025230897	1700018A23Rik	RIKEN cDNA 1700018A23 gene	antisense_RNA	72231	-
ENSMUSG00000107383	109.97	0.169	-2.567	4.70816E-13	7.4868E-11	Gm4366	-	processed_pseudogene	-	-
ENSMUSG00000107427	13.09	4.717	2.238	0.001590994	0.006161331	Gm43948	-	antisense_RNA	-	-
ENSMUSG00000107481	8.15	0.138	-2.855	0.000600063	0.002781059	4833403J16Rik	-	lincRNA	-	-
ENSMUSG00000107486	8.38	0.232	-2.106	0.004488478	0.014228647	Trbv4	-	TR_V_gene	-	-
ENSMUSG00000107494	5.42	5.167	2.369	0.003825741	0.012517252	Gm8239	-	lincRNA	-	-
ENSMUSG00000107528	46.46	5.387	2.43	4.38451E-08	1.12587E-06	Gm44442	-	lincRNA	-	-
ENSMUSG00000107666	12.45	4.332	2.115	0.000147465	0.00089539	4933406J09Rik	-	antisense_RNA	-	-
ENSMUSG00000107724	13.81	0.238	-2.071	0.000553171	0.002597996	Gm16042	-	processed_pseudogene	-	-
ENSMUSG00000107887	4.56	6.233	2.64	0.002131349	0.007817556	Gm43950	-	lincRNA	-	-

ENSMUSG00000107960	3.07	6.5	2.7	0.01199392	0.031334507	Gm35077	-	lincRNA	-	-
ENSMUSG00000108033	233.26	4.873	2.285	5.22995E-09	2.04743E-07	Gm44284	-	antisense_RNA	-	-
ENSMUSG00000108196	28.7	4.815	2.268	2.13939E-05	0.000187568	Gm36328	-	antisense_RNA	-	-
ENSMUSG00000108276	8.28	4.364	2.126	0.017029387	0.041401847	Gm36640	-	antisense_RNA	-	-
ENSMUSG00000108289	20.84	0.093	-3.426	6.75229E-07	1.09308E-05	Gm44174	-	lincRNA	-	-
ENSMUSG00000108298	12.3	0.186	-2.423	0.000631205	0.002893399	Gm44226	-	lincRNA	-	-
ENSMUSG00000108303	4.18	4.675	2.225	0.013727123	0.034755907	Gm39075	-	lincRNA	-	-
ENSMUSG00000108348	5.63	0.188	-2.411	0.006332771	0.018824411	Gm42372	predicted gene, 42372	protein_coding	105247240	-
ENSMUSG00000108353	31.85	0.247	-2.019	2.25916E-06	2.95666E-05	Gm45205	-	TEC	-	-
ENSMUSG00000108366	40.01	0.202	-2.31	2.00773E-09	9.47529E-08	Gm5586	-	processed_pseudogene	-	-
ENSMUSG00000108402	6.59	6.745	2.754	0.005325817	0.016344938	9430064I24Rik	-	TEC	-	-
ENSMUSG00000108511	5.68	0.196	-2.35	0.007492745	0.021528163	Gm44987	-	antisense_RNA	-	-
ENSMUSG00000108609	6.81	0.217	-2.205	0.016720213	0.040754952	Gm7424	-	processed_pseudogene	-	-
ENSMUSG00000108676	8.28	5.998	2.584	0.000318369	0.001657237	1700123J17Rik	RIKEN cDNA 1700123J17 gene	lincRNA	76728	-
ENSMUSG00000108732	11.22	0.173	-2.53	0.001321755	0.005300293	2310043P16Rik	-	TEC	-	-
ENSMUSG00000108782	5.94	6.298	2.655	0.003468518	0.011548229	Gm32772	-	lincRNA	-	-
ENSMUSG00000108828	6.84	4.204	2.072	0.008879756	0.02464965	Gm34549	-	lincRNA	-	-
ENSMUSG00000108854	16.02	6.991	2.805	0.0002529	0.001380425	D830036C21Rik	-	lincRNA	-	-
ENSMUSG00000108867	3.93	4.016	2.006	0.016557683	0.040422208	Gm44643	-	lincRNA	-	-
ENSMUSG00000108981	8.86	7.083	2.824	2.32823E-05	0.000200748	Gm15262	predicted gene 15262	protein_coding	108168453	-
ENSMUSG00000108986	11.12	4.685	2.228	0.001545968	0.006023307	Gm32061	predicted gene, 32061	lincRNA	102634490	-
ENSMUSG00000109014	8.78	4.513	2.174	0.000550621	0.002590079	Gm44546	-	lincRNA	-	-
ENSMUSG00000109088	7.62	5.201	2.379	0.004845719	0.015150989	Gm44593	-	lincRNA	-	-
ENSMUSG00000109198	4.18	0.014	-6.121	4.38983E-05	0.000336093	D7Bwg0826e	-	TEC	-	-

ENSMUSG00000109260	6.25	5.586	2.482	0.012101536	0.031558896	Gm30954	predicted gene, 30954	lincRNA	102633030	-
ENSMUSG00000109266	2.36	0.026	-5.243	0.000735594	0.003279061	Gm6426	-	processed_pseudogene	-	-
ENSMUSG00000109324	40.31	0.222	-2.173	1.2235E-06	1.78741E-05	Prmt1	protein arginine N-methyltransferase 1	protein_coding	15469	-
ENSMUSG00000109510	12.23	0.199	-2.333	0.000358494	0.001825273	Gm42417	-	protein_coding	-	-
ENSMUSG00000109554	9.63	5.105	2.352	0.001464254	0.00576494	A230057D06Rik	-	lincRNA	-	-
ENSMUSG00000109656	5.5	0.095	-3.402	0.000313108	0.001640305	Gm45548	-	TEC	-	-
ENSMUSG00000109704	27.52	12.477	3.641	5.10251E-06	5.78467E-05	Gm38414	predicted gene, 38414	lincRNA	330738	-
ENSMUSG00000109799	24.15	0.182	-2.46	4.45639E-06	5.18297E-05	Gm45515	-	antisense_RNA	-	-
ENSMUSG00000109838	38.91	3.999	2	2.37615E-06	3.09174E-05	Gm45341	-	lincRNA	-	-
ENSMUSG00000109903	7.71	5.927	2.567	0.001710421	0.006530931	AC158589.1	-	protein_coding	-	-
ENSMUSG00000109933	23.1	0.243	-2.042	2.11218E-06	2.80101E-05	Gm7600	-	processed_pseudogene	-	-
ENSMUSG00000109947	12.46	0.221	-2.177	0.001365472	0.0054416	Gm45669	-	antisense_RNA	-	-
ENSMUSG00000109961	5.64	4.971	2.314	0.01218606	0.031702809	Gm31983	-	lincRNA	-	-
ENSMUSG00000109966	9.42	5.481	2.454	0.000670956	0.003038409	Gm34623	predicted gene, 34623	lincRNA	102637934	-
ENSMUSG00000109967	627.19	4.847	2.277	8.43157E-08	1.93249E-06	Gm45883	-	processed_transcript	-	-
ENSMUSG00000110057	13.84	0.13	-2.94	5.05504E-06	5.74899E-05	Gm2225	-	processed_pseudogene	-	-
ENSMUSG00000110138	4.66	4.764	2.252	0.019798462	0.046656861	4831440D22Rik	-	antisense_RNA	-	-
ENSMUSG00000110217	17.87	10.43	3.383	1.47155E-06	2.08535E-05	Gm45408	-	lincRNA	-	-
ENSMUSG00000110279	798.59	0.225	-2.151	6.01681E-09	2.30091E-07	Gm45552	-	antisense_RNA	-	-
ENSMUSG00000110286	21.14	5.994	2.583	2.39721E-05	0.000205513	Gm7706	-	lincRNA	-	-
ENSMUSG00000110333	16.52	6.109	2.611	2.12472E-06	2.8109E-05	Gm45861	POTE ankyrin domain family, member G	protein_coding	70952	-
ENSMUSG00000110344	262.2	5.241	2.39	6.95661E-09	2.5668E-07	Gm45716	-	protein_coding	-	-
ENSMUSG00000110408	33.35	6.793	2.764	1.25249E-06	1.82234E-05	Gm45881	-	lincRNA	-	-
ENSMUSG00000110423	14.97	0.139	-2.852	2.10882E-06	2.79862E-05	Gm45736	-	protein_coding	-	-

ENSMUSG00000 110455	9.9	6.072	2.602	2.33453E-05	0.000201195	Gm45904	-	antisense_RNA	-	-
ENSMUSG00000 110537	48.55	4.345	2.119	8.69468E-06	8.93833E-05	Gm4316	-	lincRNA	-	-
ENSMUSG00000 110545	6.28	0.167	-2.58	0.002601198	0.009150533	Gm7730	-	processed_pseud ogene	-	-
ENSMUSG00000 110575	19.91	0.134	-2.904	1.25649E-06	1.82637E-05	6330537M06 Rik	-	TEC	-	-
ENSMUSG00000 110629	7.89	14.95	3.902	7.64311E-05	0.000524796	4930567H12R ik	RIKEN cDNA 4930567H12 gene	lincRNA	75930	-
ENSMUSG00000 110631	13.44	4.824	2.27	0.003106889	0.010569423	Gm42047	-	lincRNA	-	-
ENSMUSG00000 110649	32.08	0.189	-2.402	7.80875E-09	2.81758E-07	Gm40466	-	unprocessed_pse udogene	-	-
ENSMUSG00000 110747	84.64	6.376	2.673	2.64389E-10	1.5889E-08	AC156031.1	-	lincRNA	-	-
ENSMUSG00000 110766	54.79	4.208	2.073	4.15681E-07	7.3881E-06	AC154295.1	-	lincRNA	-	-
ENSMUSG00000 110784	5.97	7.845	2.972	0.003240347	0.010949228	AC164881.1	-	lincRNA	-	-
ENSMUSG00000 110824	4.46	6.181	2.628	0.007580795	0.021739435	AC133947.1	-	antisense_RNA	-	-
ENSMUSG00000 110843	8.82	4.573	2.193	0.001064603	0.004440542	AC153829.1	-	lincRNA	-	-
ENSMUSG00000 110862	5.98	9.514	3.25	8.71838E-05	0.000584337	CT025619.2	-	lincRNA	-	-
ENSMUSG00000 110884	5.33	5.827	2.543	0.015780522	0.038897146	AC164161.2	-	lincRNA	-	-
ENSMUSG00000 110893	7.69	6.226	2.638	0.00115532	0.004748386	AC158596.1	-	lincRNA	-	-
ENSMUSG00000 110949	10.34	0.228	-2.13	0.006160661	0.018440932	Nudt8	nudix (nucleoside diphosphate linked moiety X)-type motif 8	protein_coding	66387	-
ENSMUSG00000 111042	13.09	6.411	2.68	4.01399E-05	0.00031224	AC099761.1	-	transcribed_proc essed_pseudogen e	-	-
ENSMUSG00000 111044	12.21	0.171	-2.549	4.10726E-05	0.000318667	AC117688.1	-	antisense_RNA	-	-
ENSMUSG00000 111052	11.22	4.432	2.148	0.014457969	0.036208397	AC158622.3	-	lincRNA	-	-
ENSMUSG00000 111091	8.74	4.725	2.24	0.002229066	0.008114684	AC153897.1	-	lincRNA	-	-
ENSMUSG00000 111118	8.96	0.183	-2.446	0.017022101	0.041389734	AC151730.1	-	processed_pseud ogene	-	-
ENSMUSG00000 111133	9.51	4.975	2.315	0.000315341	0.001648634	AC160123.2	-	processed_pseud ogene	-	-
ENSMUSG00000 111182	10.02	5.201	2.379	0.001681191	0.006445342	AC158799.1	-	lincRNA	-	-
ENSMUSG00000 111334	8.12	4.716	2.238	0.000977656	0.004144254	AC162938.3	-	processed_transc ript	-	-

ENSMUSG00000111447	30.63	4.898	2.292	3.51398E-09	1.47186E-07	AC153845.3	-	antisense_RNA	-	-
ENSMUSG00000111520	5.48	7.089	2.826	0.000493852	0.002370199	AC166902.2	-	lincRNA	-	-
ENSMUSG00000111535	10.11	4.816	2.268	0.000325142	0.001689552	AC153955.5	-	lincRNA	-	-
ENSMUSG00000111542	11.12	0.095	-3.402	1.09267E-05	0.000107466	AC107669.2	-	unprocessed_pseudogene	-	-
ENSMUSG00000111602	29.43	5.651	2.498	1.03957E-07	2.31189E-06	AC156791.1	predicted gene, 32014	lincRNA	102634431	-
ENSMUSG00000111685	20.72	4.528	2.179	1.51033E-05	0.000140954	AC124577.4	-	TEC	-	-
ENSMUSG00000111692	12.84	0.123	-3.021	2.25871E-06	2.95666E-05	AC163637.2	-	protein_coding	-	-
ENSMUSG00000111731	5.42	5.738	2.521	0.02029358	0.047592709	AC153569.2	-	lincRNA	-	-
ENSMUSG00000111853	13	5.351	2.42	0.00932847	0.025634389	AC161596.2	predicted gene, 31408	lincRNA	102633624	-
ENSMUSG00000111856	161.06	0.129	-2.949	1.18002E-17	4.71195E-15	AC163623.3	-	processed_transcript	-	-
ENSMUSG00000111933	9.7	6.419	2.682	0.00014347	0.000875982	AC108401.1	-	lincRNA	-	-
ENSMUSG00000111986	5.37	5.36	2.422	0.00801945	0.022711295	AC159888.1	RIKEN cDNA 1700008C04 gene	lincRNA	75494	-
ENSMUSG00000112063	11.07	0.006	-7.464	2.23263E-09	1.02256E-07	AC161114.1	-	processed_pseudogene	-	-
ENSMUSG00000112099	25.62	0.166	-2.588	4.45521E-09	1.799E-07	AC129329.1	-	processed_pseudogene	-	-
ENSMUSG00000112126	10.04	5.406	2.435	0.000782189	0.00344465	AC121788.3	-	lincRNA	-	-
ENSMUSG00000112129	4.9	6.594	2.721	0.001360541	0.005427349	Pbld1	phenazine biosynthesis-like protein domain containing 1	protein_coding	68371	-
ENSMUSG00000112146	6.14	5.114	2.354	0.002475302	0.008804177	AC122390.1	-	lincRNA	-	-
ENSMUSG00000112314	14.94	6.416	2.682	4.92704E-05	0.00036843	AC155712.2	-	lincRNA	-	-
ENSMUSG00000112372	10.34	4.35	2.121	0.001596196	0.006180143	AC155922.2	-	lincRNA	-	-
ENSMUSG00000112426	5.14	4.111	2.04	0.007323352	0.021125913	AC114678.1	-	antisense_RNA	-	-
ENSMUSG00000112567	6.37	0.177	-2.501	0.001698393	0.006493283	CT571245.5	-	processed_pseudogene	-	-
ENSMUSG00000112569	16.05	5.059	2.339	0.001717685	0.006554487	CT010463.2	predicted gene, 33111	lincRNA	102635888	-
ENSMUSG00000112659	9.06	4.101	2.036	0.009902806	0.026916279	AC153950.2	-	lincRNA	-	-
ENSMUSG00000112704	10.8	4.338	2.117	0.000463984	0.002258217	AC124458.1	predicted gene, 46329	lincRNA	108167972	-

ENSMUSG00000 112713	23.51	9.306	3.218	1.16043E-06	1.70776E-05	AC122301.3	-	lincRNA	-	-
ENSMUSG00000 112818	4.55	5.424	2.439	0.002641629	0.009266796	AC153830.1	-	lincRNA	-	-
ENSMUSG00000 112833	5.83	4.966	2.312	0.007559379	0.021684941	AC152949.3	-	lincRNA	-	-
ENSMUSG00000 112857	20.61	8.302	3.053	3.2748E-07	6.03537E-06	AC124556.2	-	lincRNA	-	-
ENSMUSG00000 112907	18.2	0.186	-2.426	0.00054694	0.002576526	AC164623.1	-	lincRNA	-	-
ENSMUSG00000 112908	54.48	0.209	-2.259	1.8143E-06	2.48295E-05	AC126687.1	-	processed_pseud ogene	-	-
ENSMUSG00000 113017	10.56	5.316	2.41	0.000895392	0.003854648	AC154816.1	-	lincRNA	-	-
ENSMUSG00000 113030	14.21	0.211	-2.241	4.62232E-05	0.000351199	CT009754.1	-	processed_transc ript	-	-
ENSMUSG00000 113032	13.27	8.405	3.071	0.000318213	0.0016569	AC158633.3	-	antisense_RNA	-	-
ENSMUSG00000 113061	116.98	0.153	-2.707	1.93983E-11	1.79674E-09	AL607105.1	-	protein_coding	-	-
ENSMUSG00000 113113	5.98	0.146	-2.773	0.002981975	0.010219935	AC159818.1	-	processed_pseud ogene	-	-
ENSMUSG00000 113149	240.67	0.25	-2	2.45115E-09	1.11224E-07	AC099934.3	-	protein_coding	-	-
ENSMUSG00000 113158	5.23	10.685	3.418	0.000163857	0.000972376	AC099934.4	-	lincRNA	-	-
ENSMUSG00000 113198	5.61	0.097	-3.361	0.000214677	0.001208501	AL591207.1	-	processed_pseud ogene	-	-
ENSMUSG00000 113204	13.04	0.212	-2.235	0.000496552	0.002379343	CT009718.4	predicted gene, 46430	lincRNA	108168 101	-
ENSMUSG00000 113235	8.96	4.439	2.15	0.002287117	0.008268751	AC159293.1	-	lincRNA	-	-
ENSMUSG00000 113272	7.06	4.344	2.119	0.001274447	0.005143877	CT030658.1	predicted gene, 34809	lincRNA	102638 187	-
ENSMUSG00000 113295	55.56	0.217	-2.201	2.08756E-08	6.24149E-07	CT030657.1	-	lincRNA	-	-
ENSMUSG00000 113330	8.96	4.168	2.059	0.005968544	0.017964618	AC155241.3	-	lincRNA	-	-
ENSMUSG00000 113358	8.31	4.769	2.254	0.000175861	0.00102732	CT025627.1	-	lincRNA	-	-
ENSMUSG00000 113387	11.87	6.284	2.652	4.76389E-05	0.000359825	AC162181.1	-	lincRNA	-	-
ENSMUSG00000 113389	35.77	0.106	-3.231	8.90697E-13	1.34495E-10	AC126799.2	-	processed_pseud ogene	-	-
ENSMUSG00000 113465	16.91	0.196	-2.354	0.000285645	0.00151812	AC127270.2	predicted gene, 40271	lincRNA	105244 713	-
ENSMUSG00000 113471	37.38	0.052	-4.264	2.16897E-15	6.00016E-13	CT030657.2	-	lincRNA	-	-

ENSMUSG00000113550	11.5	4.29	2.101	0.000493651	0.002369867	AC162801.2	predicted gene, 29687	lincRNA	629481	-
ENSMUSG00000113558	4.11	4.396	2.136	0.008403461	0.023582975	AC104834.1	-	protein_coding	-	-
ENSMUSG00000113630	7.1	5.138	2.361	0.006720555	0.01971894	CT010460.2	RIKEN cDNA 4930404H11 gene	lincRNA	73815	-
ENSMUSG00000113717	7.34	4.764	2.252	0.010702409	0.028686097	AC110901.1	-	lincRNA	-	-
ENSMUSG00000113769	29.9	4.932	2.302	0.001772974	0.006721215	AC132623.2	RIKEN cDNA 5033406O09 gene	lincRNA	77675	-
ENSMUSG00000113831	6.81	0.245	-2.027	0.008725898	0.024309406	AC163032.1	-	lincRNA	-	-
ENSMUSG00000113867	6.25	4.283	2.099	0.013293732	0.033892603	AC132237.3	-	lincRNA	-	-
ENSMUSG00000113895	4.49	4.233	2.082	0.004438055	0.014121931	AC123705.4	-	antisense_RNA	-	-
ENSMUSG00000113949	46.76	0.209	-2.255	4.91362E-09	1.94478E-07	Scamp4	secretory carrier membrane protein 4	protein_coding	56214	-
ENSMUSG00000114003	37.68	0.177	-2.496	1.20886E-10	8.32263E-09	AC124744.2	-	processed_pseudogene	-	-
ENSMUSG00000114014	7.43	0.178	-2.492	0.001732217	0.00659733	AC134439.3	-	antisense_RNA	-	-
ENSMUSG00000114023	6.61	0.127	-2.975	0.000331156	0.001716335	CT030657.5	-	processed_pseudogene	-	-
ENSMUSG00000114134	12.48	4.326	2.113	0.002121268	0.007786938	AC132324.1	-	lincRNA	-	-
ENSMUSG00000114135	3.85	0.016	-5.972	1.66088E-05	0.000152345	AC129022.1	-	processed_pseudogene	-	-
ENSMUSG00000114202	6.57	0.215	-2.216	0.004776859	0.014972159	AC129975.1	-	processed_pseudogene	-	-
ENSMUSG00000114206	43.78	6.27	2.648	2.89148E-09	1.26725E-07	AC159301.1	predicted gene, 30551	lincRNA	102632492	-
ENSMUSG00000114264	7.18	0.009	-6.875	2.65375E-08	7.54513E-07	CT030194.1	-	antisense_RNA	-	-
ENSMUSG00000114288	17.33	4.424	2.145	0.00022014	0.001234224	AC154461.2	-	lincRNA	-	-
ENSMUSG00000114389	11.15	11.974	3.582	7.90209E-05	0.000539281	CT030182.1	-	lincRNA	-	-
ENSMUSG00000114422	276.32	5.025	2.329	1.73694E-09	8.39006E-08	AC165281.1	-	lincRNA	-	-
ENSMUSG00000114432	16293.84	4.531	2.18	8.07658E-05	0.000548689	AC160962.1	-	protein_coding	-	-
ENSMUSG00000114446	10.17	5.066	2.341	0.002287311	0.008268751	AC123867.1	-	antisense_RNA	-	-
ENSMUSG00000114487	11733.43	4.454	2.155	6.51382E-05	0.00046009	CT010498.1	-	protein_coding	-	-
ENSMUSG00000114635	3316.47	4.184	2.065	2.60059E-05	0.000219699	CT010498.2	-	protein_coding	-	-

ENSMUSG00000114688	28.33	0.24	-2.057	2.63992E-06	3.3643E-05	AC160980.2	-	lincRNA	-	-
ENSMUSG00000114726	5.99	0.229	-2.129	0.004533728	0.014321248	AC164116.1	-	antisense_RNA	-	-
ENSMUSG00000114752	4.88	7.584	2.923	0.014937028	0.037195601	AC138329.2	-	bidirectional_promoter_lncRNA	-	-
ENSMUSG00000114755	4.95	7	2.807	0.000845641	0.003681037	Galr3	galanin receptor 3	protein_coding	14429	-
ENSMUSG00000114794	21.21	4.711	2.236	7.42316E-05	0.000512238	AC158388.3	-	lincRNA	-	-
ENSMUSG00000114828	13.8	5.646	2.497	1.00826E-06	1.52119E-05	CT009713.10	-	lincRNA	-	-
ENSMUSG00000114838	7.09	9.702	3.278	4.44211E-06	5.1697E-05	AC154537.1	-	lincRNA	-	-
ENSMUSG00000114843	8.25	8.551	3.096	3.08622E-05	0.000251845	CT010472.1	-	lincRNA	-	-
ENSMUSG00000114851	9.12	5.866	2.552	5.71616E-05	0.000415005	AC156802.2	predicted gene, 46409	sense_intronic	108168076	-
ENSMUSG00000114883	7.34	0.189	-2.402	0.018646353	0.044579073	CT025689.6	-	antisense_RNA	-	-
ENSMUSG00000114934	6.43	0.083	-3.588	9.86187E-05	0.000646093	AC123738.2	-	antisense_RNA	-	-
ENSMUSG00000114937	68.56	0.154	-2.696	1.73702E-12	2.26178E-10	AC121801.1	-	antisense_RNA	-	-

Supplementary Table 3 (A): GO analysis of differentially expressed genes (DEGs) in the biological process (BP), molecular function (MF) and cellular component (CC) in LD group using the clusterProfiler R package for determining functions of the DEGs based on BH adjusted p-values (adjusted p-value for multiple testing calculated using Benjamin-Hochberg correction method to examine the significance of pathway–term enrichment).

Column Names	Description
**Category**	BP/CC/MF
**ID**	GO ID
**Description**	GO Term Description
**p.adjust**	BH adjusted p-values (adjusted p-value for multiple testing calculated using Benjamin-Hochberg correction method to examine the significance of pathway–term enrichment)
**geneID**	Gene name/symbol

**Gene Ontology table control vs LD**				
Category	ID_LD	Description	p.adjust	geneID
BP	GO:0002377	immunoglobulin production	1.98E-12	Vpreb3/Ccr6/H2-Ab1/Igkv2-137/Igkv17-127/Igkv13-84/Igkv4-80/Igkv4-72/Igkv4-63/Iglv1/Igkv13-85/Igkv3-12/Igkv10-96/Igkv14-111/Igkv10-94/Igkv14-100/Igkv3-4/Ighd
BP	GO:0002440	production of molecular mediator of immune response	1.98E-12	Vpreb3/Slamf1/Cd74/Ccr6/H2-Ab1/Igkv2-137/Igkv17-127/Igkv13-84/Igkv4-80/Igkv4-72/Igkv4-63/Iglv1/Igkv13-85/Igkv3-12/Igkv10-96/Igkv14-111/Igkv10-94/Igkv14-100/Igkv3-4/Ighd
BP	GO:0042113	B cell activation	0.000855	Cd79a/Blk/Cdkn1a/Cd74/Cd79b/Ccr6/Blnk/Tnfrsf13c/H2-Ab1/Ighv1-74/Ighd
BP	GO:0050853	B cell receptor signaling pathway	0.002473	Cd79a/Blk/Cd79b/Blnk/Ighv1-74/Ighd
BP	GO:0050670	regulation of lymphocyte proliferation	0.002473	Blk/Slamf1/Sdc4/Cdkn1a/Cd74/Pde5a/Tnfrsf13c/H2-Ab1/Ighd
BP	GO:0032944	regulation of mononuclear cell proliferation	0.002473	Blk/Slamf1/Sdc4/Cdkn1a/Cd74/Pde5a/Tnfrsf13c/H2-Ab1/Ighd
BP	GO:0046651	lymphocyte proliferation	0.002473	Cd79a/Blk/Slamf1/Sdc4/Cdkn1a/Cd74/Pde5a/Tnfrsf13c/H2-Ab1/Ighd
BP	GO:0032943	mononuclear cell proliferation	0.002473	Cd79a/Blk/Slamf1/Sdc4/Cdkn1a/Cd74/Pde5a/Tnfrsf13c/H2-Ab1/Ighd
BP	GO:0050866	negative regulation of cell activation	0.004807	Blk/Sdc4/Cd200/Cd74/Pdgfa/Pde5a/Adgrf5/H2-Ab1
BP	GO:0002683	negative regulation of immune system process	0.011851	Vpreb3/Blk/Slamf1/Sdc4/Cd200/Cd74/H2-Ob/Pde5a/Adgrf5/H2-Ab1
BP	GO:0042060	wound healing	0.011851	Blk/Sdc4/Cdkn1a/Pdgfa/Col5a1/F11r/Ndnf/Serpinb2/Gp6
BP	GO:0016064	immunoglobulin mediated immune response	0.015997	Fcer2a/Cd74/Ccr6/H2-Ab1/Ighv1-74/Ighd
BP	GO:0002577	regulation of antigen processing and presentation	0.016902	Cd74/H2-Ob/H2-Ab1
BP	GO:0071674	mononuclear cell migration	0.017623	Slamf1/Cd200/F11r/Spns2/Ccr6/Fpr2

BP	GO:0002250	adaptive immune response	0.018866	Cd79a/Fcer2a/Slamf1/Cd74/Cd79b/Ccr6/Tnfrsf13c/H2-Ab1/Ighv1-74/Ighd
BP	GO:0002495	antigen processing and presentation of peptide antigen via MHC class II	0.022764	Cd74/H2-Ob/H2-Ab1
BP	GO:2000510	positive regulation of dendritic cell chemotaxis	0.034436	Slamf1/Ccr6
BP	GO:0022407	regulation of cell-cell adhesion	0.043386	Blk/Slamf1/Sdc4/Cd74/Ppm1f/F11r/Pde5a/Tnfrsf13c/H2-Ab1
CC	GO:0019815	B cell receptor complex	0.001736	Cd79a/Cd79b/Ighd
CC	GO:0042613	MHC class II protein complex	0.001736	Cd74/H2-Ob/H2-Ab1
CC	GO:0019814	immunoglobulin complex	0.001736	Cd79a/Cd79b/Ighv1-74/Ighd
CC	GO:0009897	external side of plasma membrane	0.001736	Cd79a/Fcer2a/Slamf1/Cd74/Cd79b/Ccr6/Fcmr/Tnfrsf13c/H2-Ab1/Ighv1-74/Ighd

Supplementary Table 3 (B): GO analysis of differentially expressed genes (DEGs) in the biological process (BP), molecular function (MF) and cellular component (CC) in HD group using the clusterProfiler R package for determining functions of the DEGs based on BH adjusted p-values (adjusted p-value for multiple testing calculated using Benjamin-Hochberg correction method to examine the significance of pathway–term enrichment).

Gene Ontology table control vs HD				
Category	ID_HD	Description	p.adjust	Genes
BP	GO:0002377	immunoglobulin production	3.044E-14	Vpreb3,Il27ra,Cd40,Tcf3,Mzb1,Cd28,Traf6,Cd27,Siglecg,Cd22,Swap70,Cd40lg,Phb,Clcf1,Ccr6,Lax1,H2-Ab1,Igkv2-137,Igkv17-127,Igkv17-121,Igkv16-104,Igkv15-103,Igkv13-84,Igkv4-80,Igkv4-79,Igkv4-74,Igkv4-72,Igkv4-68,Igkv4-63,Igkv4-57-1,Igkv4-57,Igkv5-48,Igkv12-46,Igkv6-32,Igkv8-30,Igkv8-27,Igkv6-13,Igkv3-7,Ighm,Iglv1,Iglv3,Igkv13-85,Igkv1-110,Igkv4-59,Igkv5-45,Igkv3-12,Igkv4-78,Igkv1-117,Igkv8-28,Igkv10-96,Igkv5-43,Igkv1-133,Igkv6-15,Igkv9-120,Igkv10-95,Igkv6-25,Igkv3-5,Igkv3-2,Igkv1-122,Igkv6-23,Igkv4-58,Igkv4-53,Igkv14-111,Igkv6-17,Igkv1-135,Igkv10-94,Igkv8-19,Igkv9-124,Igkv3-4,Igkv6-14,Igkv19-93,Ighd
BP	GO:0006958	complement activation, classical pathway	1.409E-08	C1qbp,Cd55,Cr2,Hc,Crp,C1rl,Cfi,Trbc2,Igkc,Ighg2c,Ighg2b,Ighg3,Ighm,Ighv14-4,Iglc2,Igha,Ighv14-2,Ighv14-3,Ighd,Iglc3,Iglc1
BP	GO:0050853	B cell receptor signaling pathway	3.253E-06	Lck,Cd79a,Blk,Gcsam,Ms4a1,Lpxn,Cd38,Cd22,Cd19,Bmx,Bcar1,Cd79b,Lat2,Blnk,Trbc2,Igkc,Ighg2c,Ighg2b,Ighg3,Ighm,Ighv14-4,Iglc2,Lime1,Igha,Ighv14-2,Ighv14-3,Ighd,Iglc3,Iglc1
BP	GO:0006959	humoral immune response	5.475E-05	Fcer2a,Sh2d1a,C1qbp,Ccl1,Cxcl14,Masp1,Cd55,Cr2,Hc,Ppbp,Pf4,Cxcl10,H2-DMa,Crp,Ccr7,C1rl,Phb,Ccr2,Gpr183,Rpl30,Cfi,H2bc12,Jchain,Cd59b,H2-Ab1,Wfdc3,Trbc2,Igkc,Ighg2c,Ighg2b,Ighg3,Ighm,Ighv14-4,Iglc2,Igha,Ighv14-2,Ighv14-3,Ighd,Iglc3,Iglc1
BP	GO:0008037	cell recognition	0.0001339	Spa17,Atp8b3,Ephb3,Cct4,Mypn,Cct5,Cd6,Ephb2,Cct6a,Cd9,Clec2i,Adam18,Adam5,Cadm1,Cct2,Prtg,Ccr7,Pla2g5,Crtac1,Cnr1,Megf8,Dscam,Epha3,Robo2,Cntn2,Ovgp1,Trbc2,Igkc,Ighg2c,Ighg2b,Ighg3,Ighm,Ighv14-4,Iglc2,Hspa1b,Igha,Ighv14-2,Ighv14-3,Ighd,Iglc3,Iglc1
BP	GO:0002504	antigen processing and presentation of peptide or polysaccharide antigen via MHC class II	0.0001725	Trem2,H2-Oa,Cd74,Traf6,Pycard,Ifi30,H2-Aa,h2-DMb2,H2-DMa,Thbs1,H2-Ob,H2-Eb1,H2-Ab1,H2-DMb1
BP	GO:0006935	chemotaxis	0.0009036	Fer,Sema6b,Nkx2-1,Ddt,Shh,Kit,Ephb3,Fzd3,Nfib,Met,Trpv4,Slamf1,Etv4,C1qbp,Lhx1,Ccl9,Sema6a,Mypn,Unc5b,Ccl1,Chga,Evl,Cxcl14,F2rl1,Angpt1,Tymp,Pla2g10,St6gal1,Smoc2,Vegfa,Trem2,Cd74,Slit1,Ablim1,Pdgfa,Unc5a,Cxcr2,Ppm1f,Nfasc,Lhx4,Lamc2,Plxna2,Lmx1a,Kif5c,Hc,Pax6,Mdk,Epha8,Ephb2,Sema3c,Draxin,Rhoh,Apbb2,Ppbp,Pf4,Flt1,Ephb4,Ptn,Ephb6,Pik3c2g,Swap70,Slit2,Il34,Bcar1,Ppib,Gbx2,Sema3f,Cxcl10,Tpbg,Prtg,P2ry12,Apbb1,Ccr7,Sema6c,Thbs1,Ccr6,Trem1,Ccrl2,Ccr10,Megf8,S1pr1,Fpr1,Cxcr5,Efna5,Ccr2,Ntf3,C5ar1,Dscam,Gpr18,Gpr183,Fpr2,Epha3,Robo2,Cntn2,Epha6,Unc5c,Xcr1,Mospd2,Kdr,Gbx1,Rpl13a,Kif5a,Foxd1,Fpr3
BP	GO:0010463	mesenchymal cell proliferation	0.0009036	Sox9,Shh,Nfib,Tbx1,Wnt2,Wnt11,Fgf9,Trp63,Vegfa,Six2,Pdgfa,Prrx1,Bmp2,Lmna,Ptn,Prrx2,Fat4,Six1,Irs1,Kdr
BP	GO:0032944	regulation of mononuclear cell proliferation	0.000982	Shh,Il27ra,Mpl,Tnfrsf13b,Blk,Slamf1,H2-M3,Sdc4,Cd40,Ikzf3,Tcf3,Il6st,St6gal1,Cdkn1a,Cd4,Mzb1,Cd74,Cd6,Cd28,Traf6,Cd1d1,Cd38,Tacr1,Clec2i,Siglecg,Cd22,Rps3,Pycard,Cd40lg,Cd209a,Pawr,H2-

				Aa,Crp,Ccr7,Igfbp2,Clcf1,Ticam1,Igf2,Ccr2,Carmil2,Vtcn1,Gpr183,Spn,Btla,Rasal3,Pde5a,Sox11,Tnfrsf13c,Cd59b,H2-Ab1,Clec4g,Ighm,Lrrc32,Ighd
BP	GO:0051480	regulation of cytosolic calcium ion concentration	0.0010176	Lck,Trpv1,Trpv4,Jph2,Ccl1,P2rx1,Chga,F2rl2,F2rl1,Il6st,Cd4,Tmem178,Ms4a1,Ms4a2,Cxcr2,Trpc3,P2ry1,Cd38,Nos1,Adcyap1r1,Tacr1,Slc8a2,Cd19,Swap70,Atp2b3,Trpc6,Thy1,Hcrtr2,Pth1r,Ank2,Trpc1,Cxcl10,Adrb1,Pawr,Pml,Ccr7,Ccr6,Htr2c,Trpv3,Tmem64,Npsr1,Ccrl2,Ccr10,S1pr1,Fpr1,Adra1a,Cxcr5,Ccr2,C5ar1,Kcnk3,Fzd9,Gpr18,Adra1b,P2ry10,Selenon,Cacna1c,Fpr2,Cemip,P2ry10b,Gtf2i,Xcr1,Kdr,Gpr174,Fam155a,Fpr3,Lime1
BP	GO:0060485	mesenchyme development	0.0019411	Sox9,Sema6b,Nkx2-1,Col1a1,Shh,Pax2,Pax3,Tbx1,Wnt2,Aldh1a2,Wnt11,Sema6a,Hnrnpab,Myocd,Zfp36l1,Gata4,Fgf9,Vegfa,Ptk7,Six2,Tcof1,Cyp26a1,Acvr1,Dab2ip,Nckap1,Pax6,Mdk,Bmp2,Wwtr1,Pitx2,Sema3c,Nos3,Sfrp1,Tbx20,Loxl2,Six4,Gbx2,Sema3f,Crb2,Pawr,Spry1,Ovol2,Fermt2,Sema6c,Zfp750,Nup133,Thbs1,Erg,Megf8,Phldb1,Foxc1,Six1,Epha3,Robo2,Tead1,Sox11,Tcf15,Foxd1,Pbld1
BP	GO:0010464	regulation of mesenchymal cell proliferation	0.0022873	Sox9,Shh,Nfib,Tbx1,Wnt2,Wnt11,Fgf9,Trp63,Vegfa,Pdgfa,Prrx1,Lmna,Ptn,Prrx2,Six1,Irs1,Kdr
BP	GO:0050670	regulation of lymphocyte proliferation	0.0022974	Shh,Il27ra,Mpl,Tnfrsf13b,Blk,Slamf1,H2-M3,Sdc4,Cd40,Ikzf3,Tcf3,Il6st,Cdkn1a,Cd4,Mzb1,Cd74,Cd6,Cd28,Traf6,Cd1d1,Cd38,Tacr1,Clec2i,Siglecg,Cd22,Rps3,Pycard,Cd40lg,Cd209a,Pawr,H2-Aa,Ccr7,Igfbp2,Clcf1,Ticam1,Igf2,Ccr2,Carmil2,Vtcn1,Gpr183,Spn,Btla,Rasal3,Pde5a,Sox11,Tnfrsf13c,Cd59b,H2-Ab1,Clec4g,Ighm,Lrrc32,Ighd
BP	GO:0070661	leukocyte proliferation	0.0024509	Shh,Cd79a,Il27ra,Mpl,Tnfrsf13b,Blk,Slamf6,Slamf1,H2-M3,Sdc4,Cd40,Ikzf3,Tcf3,Cd180,F2rl1,Il6st,Clu,St6gal1,Cdkn1a,Cd4,Trem2,Mzb1,Cd74,Cd6,Cd28,Cr2,Traf6,Cd1d1,Rps6,Cd38,Ephb6,Tacr1,Cd27,Clec2i,Siglecg,Cd22,Cd19,Rps3,Pycard,Cd40lg,Cd209a,Il34,Pawr,H2-Aa,Crp,Ccr7,Igfbp2,Clcf1,Ticam1,Igf2,Ccr2,Carmil2,Vtcn1,Gpr183,Spn,Btla,Rasal3,Pde5a,Impdh2,Sox11,Tnfrsf13c,Cd59b,H2-Ab1,Clec4g,Ighm,Lrrc32,Ighd
BP	GO:0006910	phagocytosis, recognition	0.0032097	Pla2g5,Trbc2,Igkc,Ighg2c,Ighg2b,Ighg3,Ighm,Ighv14-4,Iglc2,Igha,Ighv14-2,Ighv14-3,Ighd,Iglc3,Iglc1
BP	GO:0001657	ureteric bud development	0.0032097	Sox9,Shh,Pax2,Lhx1,Sim1,Hnf1b,Wnt1,Vegfa,Six2,Kif26b,Bmp2,Smad9,Sfrp1,Slit2,Bmper,Six4,Lzts2,Smad6,Spry1,Npnt,Fmn1,Fat4,Adamts16,Foxc1,Six1,Robo2,Foxd1
BP	GO:0072163	mesonephric epithelium development	0.0032097	Sox9,Shh,Pax2,Lhx1,Sim1,Hnf1b,Wnt1,Vegfa,Six2,Kif26b,Bmp2,Smad9,Sfrp1,Slit2,Bmper,Six4,Lzts2,Smad6,Spry1,Npnt,Fmn1,Fat4,Adamts16,Foxc1,Six1,Robo2,Foxd1
BP	GO:0072164	mesonephric tubule development	0.0032097	Sox9,Shh,Pax2,Lhx1,Sim1,Hnf1b,Wnt1,Vegfa,Six2,Kif26b,Bmp2,Smad9,Sfrp1,Slit2,Bmper,Six4,Lzts2,Smad6,Spry1,Npnt,Fmn1,Fat4,Adamts16,Foxc1,Six1,Robo2,Foxd1
BP	GO:0045933	positive regulation of muscle contraction	0.0043308	Lck,Kit,Trpv4,Smtn,Myocd,Chga,Tacr1,Cttn,Pawr,Npnt,Adra1a,Chrm3,Ptgs1,Abat,Sphk1
BP	GO:0045987	positive regulation of smooth muscle contraction	0.0043308	Lck,Kit,Myocd,Tacr1,Cttn,Pawr,Npnt,Adra1a,Chrm3,Ptgs1,Abat,Sphk1
BP	GO:0050921	positive regulation of chemotaxis	0.0044979	Shh,Met,Trpv4,Slamf1,C1qbp,Ccl1,Cxcl14,F2rl1,Smoc2,Vegfa,Trem2,Cd74,Cxcr2,Ppm1f,Mdk,Ptn,Swap70,Slit2,Il34,Cxcl10,Tpbg,P2ry12,Ccr7,Thbs1,Ccr6,Megf8,S1pr1,Ccr2,Ntf3,C5ar1,Dscam,Fpr2,Mospd2,Kdr
BP	GO:0007200	phospholipase C-activating G protein-coupled receptor	0.0054211	Chga,F2rl2,F2rl1,Cxcr2,P2ry1,Hcrtr2,Pth1r,Adra2a,P2ry12,Htr2c,S1pr1,Fpr1,Adra1a,C5ar1,Gpr18,Oprd1,Adra1b,P2ry10,Fpr2,P2ry10b,Gpr27,Gpr174,Fpr3

		signaling pathway		
BP	GO:0042113	B cell activation	0.0063722	Itfg2,Cd79a,Il27ra,Kit,Tnfrsf13b,Blk,Cd40,Ikzf3,Mfng,Tcf3,Zfp36l1,Cd180,Cdkn1a,Mzb1,Cd74,Ms4a1,Cd28,Cr2,Cdh17,Cd38,Cd27,Siglecg,Cd22,Cd19,Swap70,Cd40lg,Il2rg,Sfrp1,Pou2af1,Pawr,Phb,Cd79b,Clcf1,Lat2,Ccr6,Ticam1,Cxcr5,Fzd9,Gpr183,Lax1,Btla,Txlna,Fcrl1,Blnk,Tnfrsf13c,H2-Ab1,Trbc2,Igkc,Ighg2c,Ighg2b,Ighg3,Ighm,Ighv14-4,Iglc2,Gimap1,Igha,Ighv14-2,Ighv14-3,Ighd,Iglc3,Iglc1
BP	GO:0046651	lymphocyte proliferation	0.0063722	Shh,Cd79a,Il27ra,Mpl,Tnfrsf13b,Blk,Slamf6,Slamf1,H2-M3,Sdc4,Cd40,Ikzf3,Tcf3,Cd180,Il6st,Cdkn1a,Cd4,Mzb1,Cd74,Cd6,Cd28,Cr2,Traf6,Cd1d1,Rps6,Cd38,Ephb6,Tacr1,Cd27,Clec2i,Siglecg,Cd22,Cd19,Rps3,Pycard,Cd40lg,Cd209a,Pawr,H2-Aa,Ccr7,Igfbp2,Clcf1,Ticam1,Igf2,Ccr2,Carmil2,Vtcn1,Gpr183,Spn,Btla,Rasal3,Pde5a,Impdh2,Sox11,Tnfrsf13c,Cd59b,H2-Ab1,Clec4g,Ighm,Lrrc32,Ighd
BP	GO:0002250	adaptive immune response	0.0064892	Cd3g,Cd79a,Il27ra,Fcer2a,Sh2d1a,Cd247,Tnfrsf13b,Slamf6,Slamf1,Cd48,H2-M3,Cd40,C1qbp,Irf4,Erap1,Cd4,Vegfa,Cd74,Cd28,Tnfrsf11a,Cd55,Cr2,Hc,Traf6,Cd1d1,Sit1,Ephb6,Siglecg,Ctsc,Cd19,Pycard,Swap70,Cd40lg,Bmx,Adcy7,Cd3d,Cd226,H2-Aa,H2-DMa,Crp,Ccr7,C1rl,Phb,Stx11,Cd79b,Clcf1,Lat2,Ccr6,Serpina3g,Il23r,Ccr2,Vtcn1,Gpr183,Spn,Lax1,Btla,Cd8b1,Cd8a,Cfi,H2-Eb1,Jchain,Tnfrsf13c,H2-Ab1,Clec4g,Trbc2,Igkv5-48,Igkc,Ighg2c,Ighg2b,Ighg3,Ighm,Ighv14-4,Iglc2,Iglv2,H2-DMb1,Lime1,Igkv12-41,Igha,Ighv14-2,Ighv14-3,Ighd,Iglc3,Iglc1
BP	GO:0048565	digestive tract development	0.009114	Tcf7,Shh,Kit,Ephb3,Aldh1a2,Myocd,Hnf1b,Il6st,Gata4,Fgf9,Trp63,Six2,Spdef,Pcsk5,Pdgfa,Sox17,Nckap1,Nkx2-2,Pitx2,Sfrp1,Ovol2,Alx4,Megf8,Fat4,Yipf6,Nkx3-2,Fgfr3,Acvr2b,Sox11
BP	GO:0007411	axon guidance	0.0094621	Sema6b,Nkx2-1,Shh,Ephb3,Fzd3,Nfib,Etv4,Lhx1,Sema6a,Mypn,Unc5b,Evl,Pla2g10,Vegfa,Slit1,Ablim1,Unc5a,Nfasc,Lhx4,Lamc2,Plxna2,Lmx1a,Kif5c,Pax6,Epha8,Ephb2,Sema3c,Draxin,Rhoh,Apbb2,Ephb4,Ephb6,Slit2,Gbx2,Sema3f,Prtg,Apbb1,Sema6c,Megf8,Efna5,Ntf3,Dscam,Epha3,Robo2,Cntn2,Epha6,Unc5c,Gbx1,Kif5a,Foxd1
BP	GO:0097485	neuron projection guidance	0.0094621	Sema6b,Nkx2-1,Shh,Ephb3,Fzd3,Nfib,Etv4,Lhx1,Sema6a,Mypn,Unc5b,Evl,Pla2g10,Vegfa,Slit1,Ablim1,Unc5a,Nfasc,Lhx4,Lamc2,Plxna2,Lmx1a,Kif5c,Pax6,Epha8,Ephb2,Sema3c,Draxin,Rhoh,Apbb2,Ephb4,Ephb6,Slit2,Gbx2,Sema3f,Prtg,Apbb1,Sema6c,Megf8,Efna5,Ntf3,Dscam,Epha3,Robo2,Cntn2,Epha6,Unc5c,Gbx1,Kif5a,Foxd1
BP	GO:0002580	regulation of antigen processing and presentation of peptide or polysaccharide antigen via MHC class II	0.010745	Trem2,H2-Oa,Pycard,Thbs1,H2-Ob
BP	GO:0042273	ribosomal large subunit biogenesis	0.0111055	Nhp2,Ppan,Rsl1d1,Pes1,Nle1,Ftsj3,Gtpbp4,Rpl14,Nop16,Fastkd2,Nifk,Rpl6,Nip7,Rsl24d1,Surf6,Rpl10a,Nop2,Nop53,Wdr74,Npm1,Rpl5,Nsa2,Snu13
BP	GO:0006936	muscle contraction	0.0185384	Lck,Scn4a,Irag1,Kit,Trpv1,Met,Trpv4,Sulf1,Smtn,Myocd,P2rx1,Myh3,Chga,Gata4,Myom1,Htr7,Cacna1s,Snta1,Cd38,Nos1,Cald1,Tacr1,Grip2,Cttn,Myom2,Tbx20,Hcn4,Stac,Scn5a,Ank2,Myh2,Adra2a,Scn10a,Adrb1,Pawr,Myh6,Npnt,Kcnj2,Pkp2,Dsg2,Adra1a,Chrm3,Ptgs1,Lmod1,Adra1b,Cacna1c,Myh7,Pde5a,Myh8,Myh4,Abat,Sphk1,Tnnt1,Scn1a
BP	GO:0019081	viral translation	0.0199984	Ptbp1,Eif3d,Denr,Eif3f,Eif3l,Shfl,Dhx9,Eif3b,Eif3g
BP	GO:0040017	positive regulation of locomotion	0.0234142	Fer,Alox12,Sox9,Sema6b,Col18a1,Col1a1,Itga3,Shh,Cavin1,Hmox1,Kit,Tek,Twist2,Met,Trpv4,Sash1,Slamf1,Wnt11,Cd40,C1qbp,Sparc,Sema6a,Ccl1,Cxcl14,F2rl1,Il6st,Fgf9,Angpt1,Smoc2,Vegfa,Trem2,Cd74,Pcsk5,Pdgfa,Cxcr2,Ppm1f,Lamc2,Acvr1,Dab2ip,Pax6,Mdk,Fermt1,Bmp2,Egf,Fubp1,Epb41l4b,Sema3c,Nos3,Anxa3,Flt1,Ptn,Tacr1,Pycard,Swap70,Pak3,Plp1,Slit2,Il34,Pgr,Bcar1,Thy1,Ets1,Syde1,Adra2a,Jcad,Itga2b,Sema3f,Cxcl10,Tpbg,Pawr,P2ry12,Phpt1,Bag4,Fermt2,Ccr7,Sema6c,Thbs1,Ccr6,Megf8,S1pr1,Fam83h,Rhoj,Igf2,Ccr2,Ntf3,C5ar1,Tmsb4x,Dscam,Synpo2,Carmil2,Spn,Fpr2,Cemip,Rras2,Irs1,Sh3rf2,Mospd2,Sphk1,Kdr,Cass4

BP	GO:0021515	cell differentiation in spinal cord	0.0235828	Shh,Pax3,Lhx1,Wnt1,Ighmbp2,Lbx1,Lhx4,Nkx2-2,Tal1,Pax7,Draxin,Tbx20,Tctn1,Olig2,Gbx1
BP	GO:0060677	ureteric bud elongation	0.0268977	Hnf1b,Kif26b,Six4,Lzts2,Fmn1,Six1
BP	GO:0055074	calcium ion homeostasis	0.0326497	Lck,Trpv1,Atp7b,Trpv4,Cd40,Jph2,Fam20a,Ccl1,P2rx1,Chga,F2rl2,F2rl1,Il6st,Cd4,Tmem178,Ms4a1,Ms4a2,Cxcr2,Trpc3,P2ry1,Cd38,Nos1,Adcyap1r1,Tacr1,Slc8a2,Cd19,Swap70,Atp2b3,Trpc6,Thy1,Hcrtr2,Pth1r,Scn5a,Ank2,Trpc1,Cxcl10,Adrb1,Pawr,Trpm8,Pml,Ccr7,Ccr6,Htr2c,Trpv3,Tmem64,Npsr1,Ccrl2,Ccr10,S1pr1,Fpr1,Adra1a,Cxcr5,Atp13a5,Ccr2,C5ar1,Kcnk3,Fzd9,Gpr18,Adra1b,P2ry10,Selenon,Mtln,Cacna1c,Fpr2,Cemip,P2ry10b,Grik2,Kl,Gtf2i,Xcr1,Kdr,Slc24a3,Gpr174,Fam155a,Fpr3,Lime1
BP	GO:0072189	ureter development	0.0331652	Sox9,Shh,Pax2,Aldh1a2,Lhx1,Sox17,Lzts2,Six1
BP	GO:1902656	calcium ion import into cytosol	0.0400836	Trpv1,Trpv4,P2rx1,Ms4a1,Slc8a2,Adrb1,Pawr,Trpv3,Cacna1c,Fam155a
BP	GO:0048754	branching morphogenesis of an epithelial tube	0.0492916	Sox9,Shh,Pax2,Tek,Tbx1,Met,Wnt2,Etv4,Lhx1,Hnf1b,Angpt1,Wnt1,Vegfa,Six2,Acvr1,Mdk,Bmp2,Egf,Pitx2,Tnc,Flt1,Col4a1,Slit2,Pgr,Tbx20,Six4,Gbx2,Pml,Spry1,Clic4,Npnt,Fat4,Adamts16,Six1,Hmga2,Csmd1,Kdr,Foxd1
BP	GO:0007596	blood coagulation	0.0492916	Apoh,Tspan32,Alox12,Shh,Mertk,Blk,Plek,P2rx1,Evl,F2rl2,F2rl1,Enpp4,Pdgfa,F5,Slc7a11,P2ry1,Ephb2,Pf4,Cd9,Cd40lg,Adra2a,Itga2b,Hpse,P2ry12,F11r,Thbs1,Gp5,Gp1ba,Gp1bb,Mmrn1,Abat,Adtrp,Mpig6b,Gp6
BP	GO:2000147	positive regulation of cell motility	0.0497875	Fer,Alox12,Sox9,Sema6b,Col18a1,Col1a1,Itga3,Shh,Cavin1,Hmox1,Kit,Tek,Twist2,Met,Trpv4,Sash1,Slamf1,Wnt11,Cd40,C1qbp,Sparc,Sema6a,Ccl1,Cxcl14,F2rl1,Il6st,Fgf9,Angpt1,Smoc2,Vegfa,Trem2,Cd74,Pcsk5,Pdgfa,Cxcr2,Ppm1f,Lamc2,Acvr1,Dab2ip,Pax6,Mdk,Fermt1,Bmp2,Egf,Fubp1,Epb41l4b,Sema3c,Nos3,Anxa3,Flt1,Ptn,Tacr1,Pycard,Swap70,Pak3,Plp1,Il34,Pgr,Bcar1,Thy1,Ets1,Syde1,Adra2a,Jcad,Itga2b,Sema3f,Cxcl10,Pawr,P2ry12,Phpt1,Bag4,Fermt2,Ccr7,Sema6c,Thbs1,Ccr6,S1pr1,Fam83h,Rhoj,Igf2,Ccr2,Ntf3,C5ar1,Tmsb4x,Synpo2,Carmil2,Spn,Fpr2,Cemip,Rras2,Irs1,Sh3rf2,Mospd2,Sphk1,Kdr,Cass4
CC	GO:0019814	immunoglobulin complex	1.617E-10	Cd79a,Cd79b,Jchain,Trbc2,Igkc,Ighg2c,Ighg2b,Ighg3,Ighm,Ighv14-4,Iglc2,Lime1,Igha,Ighv14-2,Ighv14-3,Ighd,Iglc3,Iglc1
CC	GO:0042571	immunoglobulin complex, circulating	2.466E-09	Jchain,Trbc2,Igkc,Ighg2c,Ighg2b,Ighg3,Ighm,Ighv14-4,Iglc2,Igha,Ighv14-2,Ighv14-3,Ighd,Iglc3,Iglc1
CC	GO:0009897	external side of plasma membrane	1.104E-08	Itga3,Spa17,Cd3g,Cd79a,Prlr,Il27ra,Fcer2a,Kit,Trpv1,Tnfrsf13b,Wnt2,Slamf1,Cd48,Cd83,H2-M3,Scube1,Cd40,Ace,P2rx1,Il6st,Gfra2,Cd4,Cd74,Cd5,Cd6,Ms4a1,Ms4a2,Cd28,Cxcr2,Tnfrsf11a,Cd55,Cr2,Kcnj3,Cd2,Glrb,Cd1d1,Cntfr,Abcg2,Cd27,Scnn1a,Cd9,Clec2i,Cd22,Art2b,Cd19,Cd40lg,Il2rg,Thy1,Cd3d,Cd226,Itga2b,Cxcl10,Stab2,Trpm8,P2ry12,H2-Aa,Ccr7,Thbs1,Cd79b,Dlk1,Ccr6,Igsf21,Htr2c,Fcmr,Ccrl2,Ccr10,S1pr1,Cxcr5,Efna5,Il23r,Ccr2,Gp1ba,Vtcn1,Spn,Btla,Cd8b1,Cd8a,Xcr1,Kdr,Klra7,Tnfrsf13c,Cd59b,H2-Ab1,Ly6a,Trbc2,Igkc,Ighg2c,Ighg2b,Ighg3,Ighm,Ighv14-4,Iglc2,Ly6c1,Ly6g6c,Igha,Ighv14-2,Ighv14-3,Ighd,Iglc3,Iglc1
CC	GO:0042613	MHC class II protein complex	8.56E-06	H2-Oa,Cd74,H2-Aa,h2-DMb2,H2-DMa,H2-Ob,H2-Eb1,H2-Ab1,H2-DMb1
CC	GO:0031012	extracellular matrix	0.0002059	Col18a1,Col1a1,Ltbp1,Shh,Otog,Wnt2,Wnt11,Matn4,Sparc,Plod1,Dcn,Pxdn,Cd180,Fgf9,Angpt1,Col14a1,Adamts20,Smoc2,Vegfa,Adamts10,Efemp2,Col17a1,Col7a1,Col9a1,Lamc2,Ush2a,Plxna2,Lamc3,Fbln7,Col11a1,Ecm1,Tnc,Sema3c,Sparcl1,Pf4,Anxa3,Sspo,Ptn,Adamts9,Mfap5,Ctsc,Col4a1,Sfrp1,Slit2,Crispld2,Bmper,Rpsa,Tnxb,Loxl2,Fras1,Hpse,Lrrn3,Adamts2,Cpz,Tecta,P3h2,Olfml2b,Thbs1,Ltbp4,Npnt,Lad1,Lrrtm3,Egflam,Col6a6,Serpinb1a,Lrrc3b,Col6a3,Igf2,Efna5,Adamts16,Lingo1,Gp1ba,Nyx,Spn,Mmrn1,Frem1,Muc5b,Hmcn1,Col4a4,Col8a1,Col28a1,Ovgp1,Lrrc32,Col6a5
CC	GO:0030312	external encapsulating structure	0.0002059	Col18a1,Col1a1,Ltbp1,Shh,Otog,Wnt2,Wnt11,Matn4,Sparc,Plod1,Dcn,Pxdn,Cd180,Fgf9,Angpt1,Col14a1,Adamts20,Smoc2,Vegfa,Adamts10,Efemp2,Col17a1,Col7a1,Col9a1,Lamc2,Ush2a,Plxna2,Lamc3,Fbln7,Col11a1,Ecm1,Tnc,Sema3c,Sparcl1,Pf4,Anxa3,Sspo,Ptn,Adamts9,Mfap5,Ctsc,Col4a1,Sfrp1,Slit2,Crispld2,Bmper,Rpsa,Tnxb,Loxl2,Fras1,Hpse,

				Lrrn3,Adamts2,Cpz,Tecta,P3h2,Olfml2b,Thbs1,Ltbp4,Npnt,Lad1,Lrrtm3,Egflam,Col6a6,Serpinb1a,Lrrc3b,Col6a3,Igf2,Efna5,Adamts16,Lingo1,Gp1ba,Nyx,Spn,Mmrn1,Frem1,Muc5b,Hmcn1,Col4a4,Col8a1,Col28a1,Ovgp1,Lrrc32,Col6a5
CC	GO:0019815	B cell receptor complex	0.0080468	Cd79a,Cd79b,Ighm,Lime1,Ighd,Iglc1
CC	GO:0005840	ribosome	0.0086856	Mrpl2,Rps11,Mrpl49,Rps18,Rps25,Mrpl52,Mrpl58,Mrpl45,Mrpl24,Rps27a,Mrpl13,Mrps18a,Mrpl28,Mrps18b,Mrpl16,Rpl14,Mrpl44,Mrpl9,Rps20,Rps6,Elavl4,Rpl22,Rpl6,Rps3,Rps4x,Rsl24d1,Mrps22,Rpsa,Mrpl15,Mrps2,Mrpl47,Mrps26,Mrpl23,Rpl10a,Abcf1,Mtg1,Rpl22l1,Mrpl12,Dhx9,Mrps16,Npm1,Mrps14,Rpl5,Rpl30,Rpl18,Rpl36a-ps1,Rpl9-ps6,Rps15,Chchd1,Rps18-ps6,Rpl13a,Rps27,Rps18-ps5
CC	GO:0044391	ribosomal subunit	0.0086856	Mrpl2,Rps11,Mrpl49,Rps18,Rps25,Mrpl52,Mrpl58,Mrpl45,Mrpl24,Rps27a,Mrpl13,Mrps18a,Mrpl28,Mrps18b,Mrpl16,Rpl14,Mrpl44,Mrpl9,Rps20,Rps6,Rpl22,Rpl6,Rps3,Rps4x,Rsl24d1,Mrps22,Rpsa,Mrpl15,Mrps2,Mrpl47,Mrps26,Mrpl23,Rpl10a,Mrpl12,Mrps16,Npm1,Mrps14,Rpl5,Rpl30,Rpl18,Rpl36a-ps1,Rpl9-ps6,Rps15,Rps18-ps6,Rpl13a,Rps27,Rps18-ps5
CC	GO:0005852	eukaryotic translation initiation factor 3 complex	0.0120607	Eif3d,Eif3e,Eif3m,Eif3i,Eif3c,Eif3f,Eif3l,Eif3b,Eif3g
CC	GO:0005581	collagen trimer	0.034634	Col18a1,Col1a1,Dcn,Col14a1,Col17a1,Col7a1,Col9a1,Col11a1,Col4a1,Colec10,Col6a6,Col6a3,Eda,C1qtnf7,Col4a4,Col8a1,Col28a1,Col6a5
MF	GO:0004930	G protein-coupled receptor activity	3.16E-05	Fzd3,Npffr1,F2rl2,F2rl1,Slc22a22,Grm8,Htr7,Pth2r,Cxcr2,Sctr,P2ry1,Adgrl2,Adcyap1r1,Tacr1,Adgrg2,Adgrg1,Hcrtr2,Pth1r,Vipr1,Cysltr2,Adra2a,Adrb1,Sigmar1,Rorb,P2ry12,Ccr7,Ccr6,Htr2c,Gpr45,Npsr1,Ccrl2,Ccr10,S1pr4,Cnr1,S1pr1,Fpr1,Adra1a,Chrm3,Cxcr5,Ccr2,C5ar1,Fzd9,Gpr171,Gpr18,Oprd1,Adra1b,Prokr2,Htr1f,P2ry10,Gpr183,Fpr2,Adgrg4,P2ry10b,Adgrf5,Xcr1,Adgrg5,Sphk1,Grm4,Gpr27,Gpr174,Fpr3,Mrgpra2a,Mrgpra2b,Galr3
MF	GO:0034987	immunoglobulin receptor binding	3.16E-05	Jchain,Trbc2,Igkc,Ighg2c,Ighg2b,Ighg3,Ighm,Ighv14-4,Iglc2,Igha,Ighv14-2,Ighv14-3,Ighd,Iglc3,Iglc1
MF	GO:0023026	MHC class II protein complex binding	0.0006572	Cd4,H2-Oa,Cd74,h2-DMb2,H2-DMa,H2-Ob,H2-DMb1
MF	GO:0005198	structural molecule activity	0.0006572	Col18a1,Col1a1,Ltbp1,Mrpl2,Rps11,Hmox1,Mrpl49,Rps18,Otog,Rps25,Mrpl52,Tubb1,Pls3,Matn4,Mpp2,Sparc,Mrpl24,Dcn,Rps27a,Sntg2,Pxdn,Mrpl13,Col14a1,Mrps18a,Myom1,Mrpl28,Mrps18b,Mrpl16,Efemp2,Col17a1,Copg2,Col7a1,Rpl14,Col9a1,Lamc2,Bfsp1,Snta1,Epb41l1,Col11a1,Ecm1,Mrpl9,Rps20,Tnc,Rps6,Macf1,Rpl22,Rpl6,Sspo,Mfap5,Rps3,Rps4x,Plp1,Myom2,Col4a1,Slit2,Bmper,Rsl24d1,Mrps22,Rpsa,Tnxb,Mrpl15,Fras1,Nup54,Mrps2,Rims2,Mrpl47,Shank2,Tecta,Mrpl23,Rpl10a,Rpl22l1,Nup133,Mrpl12,Thbs1,Dnm3,Ltbp4,Npnt,Lad1,Jph1,Col6a6,Ncmap,Col6a3,Mrps16,Krt15,Mmrn1,Mrps14,Rpl5,Rpl30,Rpl18,Rpl9-ps6,Rps15,Hmcn1,Col4a4,Tuba3a,Col8a1,Col28a1,Rpl13a,Rps27,Col6a5
MF	GO:0003823	antigen binding	0.002456	Lck,Cd48,H2-M3,Cd40,Cd1d1,H2-Aa,Jchain,H2-Ab1,Trbc2,Igkc,Ighg2c,Ighg2b,Ighg3,Ighm,Ighv14-4,Iglc2,Igha,Ighv14-2,Ighv14-3,Ighd,Iglc3,Iglc1
MF	GO:0022836	gated channel activity	0.0039237	Scn4a,Trpv1,Gabrb2,Gabra1,Trpv4,Kcnq2,Hcn2,Gabrg2,P2rx1,Hcn1,Clic6,Scn8a,Clic5,Ccdc51,Gria4,Cacna1s,Kcnj3,Pex5l,Trpc3,Glrb,Gpr89,Gabra4,Scnn1a,Tmc5,Kcnu1,Grik4,Hcn4,Scn5a,Gabrb3,Kcnk1,Kcnh5,Scn10a,Tmem109,Trpm8,Kcnk9,Clic4,Ano2,Grid1,Kcnj2,Kcnj12,Kcnj4,Cnr1,Kcnk3,Cacna1c,Kcnj16,Gabra5,Clca3a1,Grik2,Scn3a,Kcng2,Kcnd2,Atp5g2,Hvcn1,Scn1a,Cacng3,Fam155a,Kcnb2
MF	GO:0005539	glycosaminoglycan binding	0.0039237	Apoh,Shh,Sulf1,C1qbp,Dcn,Habp4,Fgf9,Nell2,Fgf12,Fstl1,Smoc2,Vegfa,Trem2,Dpysl3,Efemp2,Slit1,Lamc2,Mdk,Fbln7,Col11a1,Rspo1,Rpl22,Pf4,Ptn,Sfrp1,Slit2,Adgrg1,Crispld2,Rspo4,Tnxb,Cxcl10,Stab2,Thbs1,Pla2g5,Stab1,Egflam,Cemip,Lipg,Jchain
MF	GO:0001653	peptide receptor activity	0.0074053	F2rl2,F2rl1,Pth2r,Cxcr2,Sctr,Adcyap1r1,Tacr1,Hcrtr2,Pth1r,Vipr1,Cysltr2,Sigmar1,Ccr7,Ccr6,Npsr1,Ccrl2,Ccr10,Fpr1,Cxcr5,Ccr2,Oprd1,Prokr2,Fpr2,Xcr1,Fpr3,Galr3
MF	GO:0005216	ion channel activity	0.0294758	Scn4a,Atp5d,Trpv1,Gabrb2,Gabra1,Trpv4,Kcnq2,Slc1a4,Best3,Hcn2,Gabrg2,P2rx1,Hcn1,Clic6,Scn8a,Clic5,Ccdc51,Gria4,Cacna1s,Kcnj3,Pex5l,Trpc3,Glrb,Gpr89,Gabra4,Scnn1a,Tmc5,Kcnu1,Trpc6,Grik4,Tmem266,Hcn4,Scn5a,Trpc1,Gabrb3,Kcnk1,Kcnh5,Scn10a,Trpm8,Kcnk9,Clic4,Ano2,Abcc8,Grid1,Kcnj2,Kcnj12,Trpv3,Kcnj4,Cnr1,Calhm6,Kcnk3,Cacna1c,Kcnj16,Gabra5,Ano5,Clca3a1,Grik2,Scn3a,Kcng2,Kcnd2,Slc24a3,Hvcn1,Scn1a,Cacng3,Fam155a,Kcnb2

MF	GO:0005272	sodium channel activity	0.0463998	Scn4a,Hcn2,Hcn1,Scn8a,Scnn1a,Grik4,Hcn4,Scn5a,Kcnk1,Scn10a,Grik2,Scn3a,Scn1a

Supplementary Table 4 (A): KEGG pathway analysis of differentially expressed genes in LD group using the clusterProfiler R package for determining functions of the DEGs based on BH adjusted p-values (adjusted p-value for multiple testing calculated using Benjamin-Hochberg correction method).

Column Header	Description
**ID**	Pathway ID
**Description**	Pathway Description
**Count**	No. of Genes involved in the pathway.
**GeneRatio**	k/n
	k = size of the‘overlap of 'a vecto’ of gene id' you input with the specific geneset (the number of genes within that list n, which are annotated to the node).
	n = size of the‘overlap of 'a vecto’ of gene id' you input with all the members of the collection of genesets (is the size of the list of genes of interest).
**BgRatio**	M/N
	M = size of the geneset (is the number of genes within that distribution that are annotated (either directly or indirectly) to the node of interest).
	N = size of all of the unique genes in the collection of genesets is the total number of genes in the background distribution (Universe).
**pvalue**	To examine the significance of pathway–term enrichment
**p.adjust**	BH adjusted p-values (adjusted p-value for multiple testing calculated using Benjamin-Hochberg correction method)
**geneID**	Entrez gene Id

**KEGG pathway analysis of Control vs LD (p.adjust <0.05)**							
**ID**	**Description**	**GeneRatio**	**BgRatio**	**pvalue**	**p.adjust**	**geneID**	**Count**
mmu05340	Primary immunodeficiency	4/38	32/6029	4.2525E-05	0.002521	12518/12265/17060/72049	4
mmu04672	Intestinal immune network for IgA production	4/38	36/6029	6.8412E-05	0.002521	15002/381091/72049/14961	4
mmu05150	Staphylococcus aureus infection	4/38	36/6029	6.8412E-05	0.002521	15002/14289/381091/14961	4
mmu04612	Antigen processing and presentation	5/38	73/6029	8.334E-05	0.002521	12265/16149/15002/381091/14961	5
mmu05310	Asthma	3/38	18/6029	0.00017662	0.004274	15002/381091/14961	3

mmu05322	Systemic lupus erythematosus	5/38	99/6029	0.00035232	0.007105	20822/15002/381091/100041230/14961	5
mmu04514	Cell adhesion molecules	5/38	126/6029	0.00106321	0.018378	20971/16456/15002/381091/14961	5
mmu05169	Epstein-Barr virus infection	6/38	196/6029	0.00126611	0.01915	14128/12575/15002/17060/381091/14961	6
mmu04640	Hematopoietic cell lineage	4/38	83/6029	0.00172653	0.023212	14128/15002/381091/14961	4
mmu05152	Tuberculosis	5/38	146/6029	0.00204938	0.024797	12265/16149/15002/381091/14961	5
mmu05330	Allograft rejection	3/38	45/6029	0.00273143	0.027079	15002/381091/14961	3
mmu05332	Graft-versus-host disease	3/38	45/6029	0.00273143	0.027079	15002/381091/14961	3
mmu05320	Autoimmune thyroid disease	3/38	46/6029	0.00290935	0.027079	15002/381091/14961	3
mmu05321	Inflammatory bowel disease	3/38	49/6029	0.00348548	0.029569	15002/381091/14961	3
mmu05145	Toxoplasmosis	4/38	102/6029	0.00366553	0.029569	12265/15002/381091/14961	4
mmu04940	Type I diabetes mellitus	3/38	52/6029	0.00412695	0.03121	15002/381091/14961	3
mmu05140	Leishmaniasis	3/38	63/6029	0.00707106	0.047533	15002/381091/14961	3
mmu05323	Rheumatoid arthritis	3/38	63/6029	0.00707106	0.047533	15002/381091/14961	3
mmu05164	Influenza A	4/38	130/6029	0.00864008	0.055024	12265/15002/381091/14961	4
mmu05416	Viral myocarditis	3/38	70/6029	0.00945696	0.057215	15002/381091/14961	3
mmu05166	Human T-cell leukemia virus 1 infection	5/38	214/6029	0.01038877	0.059859	12575/15002/381091/72049/14961	5
mmu04662	B cell receptor signaling pathway	3/38	79/6029	0.01314186	0.07228	12518/15985/17060	3
mmu04658	Th1 and Th2 cell differentiation	3/38	82/6029	0.01452856	0.076433	15002/381091/14961	3
mmu04659	Th17 cell differentiation	3/38	93/6029	0.02030498	0.102371	15002/381091/14961	3
mmu05216	Thyroid cancer	2/38	36/6029	0.02129919	0.103088	12575/18510	2
mmu05168	Herpes simplex virus 1 infection	6/38	370/6029	0.02667825	0.124156	208292/16149/15002/232855/381091/14961	6
mmu05218	Melanoma	2/38	61/6029	0.05609612	0.247569	12575/18590	2
mmu04145	Phagosome	3/38	140/6029	0.05728861	0.247569	15002/381091/14961	3
mmu04115	p53 signaling pathway	2/38	64/6029	0.06107973	0.25485	64058/12575	2
mmu05214	Glioma	2/38	67/6029	0.06621048	0.267049	12575/18590	2
mmu05202	Transcriptional misregulation in cancer	3/38	155/6029	0.07310355	0.28534	12575/18590/18510	3
mmu04512	ECM-receptor interaction	2/38	78/6029	0.08617167	0.322373	20971/243816	2
mmu00340	Histidine metabolism	1/38	15/6029	0.09058422	0.322373	140483	1
mmu04950	Maturity onset diabetes of the young	1/38	15/6029	0.09058422	0.322373	20526	1
mmu04928	Parathyroid hormone synthesis, secretion and action	2/38	85/6029	0.09970861	0.344707	12575/110385	2

mmu04064	NF-kappa B signaling pathway	2/38	91/6029	0.11175848	0.36548	17060/72049	2
mmu05215	Prostate cancer	2/38	91/6029	0.11175848	0.36548	12575/18590	2
mmu00532	Glycosaminoglycan b–osynthesis - chondroitin sulfate / dermatan sulfate	1/38	20/6029	0.11896388	0.378806	53374	1
mmu04392	Hippo signal–ng pathway - multiple species	1/38	24/6029	0.14104498	0.437601	21678	1
mmu01040	Biosynthesis of unsaturated fatty acids	1/38	26/6029	0.15188252	0.459445	20249	1
mmu04611	Platelet activation	2/38	112/6029	0.15653795	0.461978	19224/243816	2
mmu04630	JAK-STAT signaling pathway	2/38	114/6029	0.16097338	0.463757	12575/18590	2
mmu00230	Purine metabolism	2/38	120/6029	0.1744191	0.479653	110385/242202	2
mmu05418	Fluid shear stress and atherosclerosis	2/38	120/6029	0.1744191	0.479653	20971/18590	2
mmu00590	Arachidonic acid metabolism	1/38	33/6029	0.18877549	0.507596	19224	1
mmu04973	Carbohydrate digestion and absorption	1/38	35/6029	0.19902613	0.512386	20526	1
mmu05219	Bladder cancer	1/38	35/6029	0.19902613	0.512386	12575	1
mmu04930	Type II diabetes mellitus	1/38	40/6029	0.22410407	0.564929	20526	1
mmu05206	MicroRNAs in cancer	2/38	149/6029	0.24145788	0.567586	12575/18590	2
mmu04610	Complement and coagulation cascades	1/38	44/6029	0.24361441	0.567586	18788	1
mmu04923	Regulation of lipolysis in adipocytes	1/38	45/6029	0.24841686	0.567586	19224	1
mmu04024	cAMP signaling pathway	2/38	159/6029	0.26499342	0.567586	110385/243764	2
mmu04613	Neutrophil extracellular trap formation	2/38	159/6029	0.26499342	0.567586	14289/100041230	2
mmu04918	Thyroid hormone synthesis	1/38	49/6029	0.26733149	0.567586	18510	1
mmu04151	PI3K-Akt signaling pathway	3/38	288/6029	0.27196976	0.567586	12575/18590/243764	3
mmu04061	Viral protein interaction with cytokine and cytokine receptor	1/38	50/6029	0.27198724	0.567586	12458	1
mmu05217	Basal cell carcinoma	1/38	50/6029	0.27198724	0.567586	12575	1
mmu04060	Cytokine-cytokine receptor interaction	2/38	162/6029	0.27206584	0.567586	12458/72049	2
mmu04080	Neuroactive ligand-receptor interaction	2/38	165/6029	0.27913905	0.572472	243764/14289	2
mmu03320	PPAR signaling pathway	1/38	53/6029	0.28578233	0.573924	20249	1
mmu05205	Proteoglycans in cancer	2/38	175/6029	0.30269118	0.573924	20971/12575	2
mmu01212	Fatty acid metabolism	1/38	58/6029	0.30821051	0.573924	20249	1
mmu04924	Renin secretion	1/38	58/6029	0.30821051	0.573924	12722	1
mmu05213	Endometrial cancer	1/38	58/6029	0.30821051	0.573924	12575	1
mmu04917	Prolactin signaling pathway	1/38	61/6029	0.32133665	0.573924	20526	1

mmu04721	Synaptic vesicle cycle	1/38	62/6029	0.3256579	0.573924	116837	1
mmu04020	Calcium signaling pathway	2/38	186/6029	0.32847758	0.573924	18590/243764	2
mmu05230	Central carbon metabolism in cancer	1/38	64/6029	0.33422017	0.573924	20526	1
mmu04810	Regulation of actin cytoskeleton	2/38	191/6029	0.3401307	0.573924	18590/243764	2
mmu04972	Pancreatic secretion	1/38	66/6029	0.34267655	0.573924	12722	1
mmu05211	Renal cell carcinoma	1/38	68/6029	0.35102832	0.573924	12575	1
mmu04911	Insulin secretion	1/38	69/6029	0.35516536	0.573924	20526	1
mmu01524	Platinum drug resistance	1/38	70/6029	0.35927672	0.573924	12575	1
mmu05223	Non-small cell lung cancer	1/38	70/6029	0.35927672	0.573924	12575	1
mmu05203	Viral carcinogenesis	2/38	201/6029	0.36326599	0.573924	12575/100041230	2
mmu01521	EGFR tyrosine kinase inhibitor resistance	1/38	74/6029	0.37546843	0.573924	18590	1
mmu05220	Chronic myeloid leukemia	1/38	74/6029	0.37546843	0.573924	12575	1
mmu04974	Protein digestion and absorption	1/38	75/6029	0.37945369	0.573924	12831	1
mmu05032	Morphine addiction	1/38	75/6029	0.37945369	0.573924	110385	1
mmu05212	Pancreatic cancer	1/38	75/6029	0.37945369	0.573924	12575	1
mmu04540	Gap junction	1/38	78/6029	0.39126146	0.584477	18590	1
mmu04012	ErbB signaling pathway	1/38	81/6029	0.40285036	0.59445	12575	1
mmu04726	Serotonergic synapse	1/38	84/6029	0.41422433	0.594982	19224	1
mmu05210	Colorectal cancer	1/38	85/6029	0.41796856	0.594982	12575	1
mmu05222	Small cell lung cancer	1/38	85/6029	0.41796856	0.594982	12575	1
mmu01522	Endocrine resistance	1/38	87/6029	0.42538725	0.594982	12575	1
mmu04922	Glucagon signaling pathway	1/38	88/6029	0.42906199	0.594982	20526	1
mmu04670	Leukocyte transendothelial migration	1/38	89/6029	0.43271384	0.594982	16456	1
mmu05231	Choline metabolism in cancer	1/38	93/6029	0.44709514	0.607848	18590	1
mmu04066	HIF-1 signaling pathway	1/38	95/6029	0.45415205	0.60852	12575	1
mmu04725	Cholinergic synapse	1/38	96/6029	0.45764754	0.60852	243764	1
mmu04931	Insulin resistance	1/38	98/6029	0.46457325	0.611015	20526	1
mmu04152	AMPK signaling pathway	1/38	113/6029	0.51384119	0.650598	20249	1
mmu04110	Cell cycle	1/38	116/6029	0.52315088	0.650598	12575	1
mmu04068	FoxO signaling pathway	1/38	117/6029	0.52621536	0.650598	12575	1
mmu05224	Breast cancer	1/38	120/6029	0.53529419	0.650598	12575	1

mmu05226	Gastric cancer	1/38	120/6029	0.53529419	0.650598	12575	1
mmu05200	Pathways in cancer	3/38	444/6029	0.53804723	0.650598	12575/18590/18510	3
mmu04380	Osteoclast differentiation	1/38	122/6029	0.54125239	0.650598	17060	1
mmu05162	Measles	1/38	123/6029	0.54420354	0.650598	27218	1
mmu05160	Hepatitis C	1/38	126/6029	0.55294649	0.650598	12575	1
mmu04934	Cushing syndrome	1/38	127/6029	0.55582436	0.650598	12575	1
mmu04723	Retrograde endocannabinoid signaling	1/38	128/6029	0.55868418	0.650598	116837	1
mmu04921	Oxytocin signaling pathway	1/38	131/6029	0.5671565	0.650598	12575	1
mmu04072	Phospholipase D signaling pathway	1/38	132/6029	0.56994525	0.650598	18590	1
mmu04390	Hippo signaling pathway	1/38	132/6029	0.56994525	0.650598	21678	1
mmu04530	Tight junction	1/38	137/6029	0.58362866	0.659439	16456	1
mmu05161	Hepatitis B	1/38	140/6029	0.59163423	0.659439	12575	1
mmu04022	cGMP-PKG signaling pathway	1/38	142/6029	0.5968878	0.659439	242202	1
mmu04062	Chemokine signaling pathway	1/38	143/6029	0.59948985	0.659439	12458	1
mmu05225	Hepatocellular carcinoma	1/38	147/6029	0.60973548	0.664667	12575	1
mmu05034	Alcoholism	1/38	150/6029	0.61725177	0.666233	100041230	1
mmu04218	Cellular senescence	1/38	152/6029	0.62218413	0.666233	12575	1
mmu05167	Kaposi sarcoma-associated herpesvirus infection	1/38	172/6029	0.6682245	0.709256	12575	1
mmu04015	Rap1 signaling pathway	1/38	184/6029	0.69317559	0.723024	18590	1
mmu04510	Focal adhesion	1/38	186/6029	0.69715246	0.723024	18590	1
mmu04014	Ras signaling pathway	1/38	187/6029	0.69912203	0.723024	18590	1
mmu05208	Chemical car–inogenesis - reactive oxygen species	1/38	197/6029	0.7181442	0.736402	57344	1
mmu05163	Human cytomegalovirus infection	1/38	201/6029	0.72542061	0.737613	12575	1
mmu04010	MAPK signaling pathway	1/38	260/6029	0.81370221	0.820483	18590	1
mmu05165	Human papillomavirus infection	1/38	297/6029	0.85422635	0.854226	12575	1

Supplementary Table 4 (B): KEGG pathway analysis of differentially expressed genes in HD group using the clusterProfiler R package for determining functions of the DEGs based on BH adjusted p-values (adjusted p-value for multiple testing calculated using Benjamin-Hochberg correction method).

KEGG pathway analysis of Control vs HD (p.adjust<0.05)							
**ID**	**Description**	**GeneRatio**	**BgRatio**	**pvalue**	**p.adjust**	**geneID**	**Count**
mmu04640	Hematopoietic cell lineage	33/807	79/5862	7.03353E-10	2.27183E-07	16400/12502/14128/16590/12504/15001/12507/12482/16178/13136/12902/12481/12479/12494/12527/12483/12478/12500/16399/14960/15000/14998/15002/14729/14723/14724/12526/12525/14969/381091/333883/14961/14999	33
mmu05310	Asthma	12/807	15/5862	1.31414E-08	2.12234E-06	21939/15001/14126/21947/14960/15000/14998/15002/14969/381091/14961/14999	12
mmu04080	Neuroactive ligand-receptor interaction	48/807	158/5862	3.39042E-08	3.65035E-06	19116/193034/14401/14394/237362/14406/18436/14064/14063/14823/15566/14802/213527/319229/15139/18441/14658/14397/11517/21336/110637/387285/19228/22354/70086/14402/11551/11554/14803/15560/13611/12801/13609/14293/11549/12671/12273/18386/11548/15557/78826/14289/213438/110886/14806/268934/14294/14429	48
mmu05150	Staphylococcus aureus infection	19/807	39/5862	1.61463E-07	1.30381E-05	16415/17174/15001/15139/14960/15000/14998/15002/14293/12273/14289/16665/12630/14969/225256/381091/14961/14999/14294	19
mmu04672	Intestinal immune network for IgA production	15/807	33/5862	9.81823E-06	0.00056931	57916/21939/15001/12487/21947/14960/15000/14998/15002/12777/14969/381091/72049/14961/14999	15
mmu03010	Ribosome	37/807	132/5862	1.05754E-05	0.00056931	27398/27207/20084/75617/67707/78294/68537/68565/68611/94063/67115/78523/67427/20104/19934/19988/27050/20102/225215/16785/27395/118451/19935/19896/68028/56282/66483/66242/100043813/64659/100503670/19946/19899/20054/22121/100040298/57294	37
mmu04060	Cytokine-cytokine receptor interaction	39/807	149/5862	3.49177E-05	0.00150366	13608/19116/50931/17480/57916/21939/20308/20290/57266/16195/12504/85030/16178/12765/21934/11477/12156/12804/57349/56744/21940/21947/16186/76527/12163/15945/16324/12775/56708/12458/12777/12145/209590/12772/239114/13607/23832/11481/72049	39
mmu04512	ECM-receptor interaction	24/807	75/5862	3.72424E-05	0.00150366	12842/16400/20971/16419/12839/16782/23928/21923/12826/81877/16399/231470/21825/114249/245026/14729/12835/14723/14724/64176/329872/12829/243816/665033	24
mmu05340	Primary immunodeficiency	14/807	33/5862	5.05408E-05	0.001813854	16818/12518/57916/21939/12265/12504/12478/21947/16186/12500/12526/12525/17060/72049	14
mmu04514	Cell adhesion molecules	31/807	123/5862	0.000450451	0.014549569	16415/15896/69524/14991/20971/21939/12504/15001/12511/12487/269116/12481/12483/21947/245537/54725/225825/14960/15000/14998/16456/15002/242122/20737/21367/12526/12525/14969/381091/14961/14999	31
mmu04061	Viral protein interaction with cytokine and cytokine receptor	16/807	49/5862	0.000565451	0.016603709	20308/20290/57266/16195/12765/57349/56744/16186/76527/15945/12775/12458/12777/12145/12772/23832	16
mmu05322	Systemic lupus erythematosus	25/807	95/5862	0.000798014	0.021479867	21939/20822/15001/12487/15139/21947/14960/15000/14998/15002/326619/14969/319155/319184/15267/381091/319164/319157/665622/100041230/665596/14961/14999/319187/319189	25
mmu04940	Type I diabetes mellitus	15/807	50/5862	0.002194035	0.052513486	14991/15001/12487/19275/14960/15000/14998/12876/15002/14969/15893/381091/14415/14961/14999	15

mmu05323	Rheumatoid arthritis	17/807	60/5862	0.002276126	0.052513486	16415/11433/21687/11600/22339/15001/12487/21934/14254/14960/15000/14998/15002/14969/381091/14961/14999	17
mmu04727	GABAergic synapse	19/807	72/5862	0.003165204	0.068157396	14401/76257/14394/57138/432530/14406/12292/14397/57436/11513/14681/14697/66066/14402/22348/12288/110886/268860/14415	19
mmu05330	Allograft rejection	13/807	43/5862	0.003942054	0.079580216	14991/21939/15001/12487/21947/14960/15000/14998/15002/14969/381091/14961/14999	13
mmu04659	Th17 cell differentiation	22/807	92/5862	0.005775357	0.102375726	16818/12502/50931/12503/20183/16364/16195/12504/18037/15001/16186/12500/14960/15000/14998/15002/209590/19094/14969/381091/14961/14999	22
mmu01040	Biosynthesis of unsaturated fatty acids	9/807	26/5862	0.005924732	0.102375726	74559/56473/66775/83603/20249/170439/217698/30963/26897	9
mmu05320	Autoimmune thyroid disease	13/807	45/5862	0.006052803	0.102375726	14991/21939/15001/12487/21947/14960/15000/14998/15002/14969/381091/14961/14999	13
mmu04612	Antigen processing and presentation	18/807	71/5862	0.006339054	0.102375726	14991/12265/12504/15001/16149/65972/14960/15000/14998/15002/12526/12525/14969/381091/14961/14999/15511/193740	18
mmu05416	Viral myocarditis	17/807	68/5862	0.009103436	0.140019521	16415/14991/21939/15001/12487/13136/21947/14960/15000/14998/17888/15002/140781/14969/381091/14961/14999	17
mmu04610	Complement and coagulation cascades	12/807	44/5862	0.013292045	0.189492327	16415/14064/12759/17174/13136/14067/12902/15139/12273/12630/333883/20701	12
mmu04742	Taste transduction	11/807	39/5862	0.013493262	0.189492327	14394/18441/14397/20276/330953/12671/15557/12288/110886/20269/268934	11
mmu04658	Th1 and Th2 cell differentiation	18/807	77/5862	0.015013064	0.202050821	16818/12502/12503/12504/18037/15001/16186/12500/14960/15000/14998/15002/19094/14969/433586/381091/14961/14999	18
mmu05033	Nicotine addiction	9/807	30/5862	0.016331007	0.210996617	14401/14394/14406/14802/14397/140919/14402/22348/110886	9
mmu05332	Graft-versus-host disease	11/807	42/5862	0.023446043	0.291271994	14991/15001/12487/14960/15000/14998/15002/14969/381091/14961/14999	11
mmu05217	Basal cell carcinoma	12/807	48/5862	0.026191978	0.313333658	21414/107986/20423/14365/22413/22411/23805/22408/12575/12156/14371/21415	12
mmu04360	Axon guidance	31/807	160/5862	0.028240312	0.325772166	20359/20423/13845/14365/17295/20358/107449/20562/226251/107448/18845/22065/13842/13844/20348/231148/13846/13848/18481/20563/22068/22063/20350/20360/241656/13640/13837/268902/13840/22253/98932	31
mmu05171	Coronavi–us disease - COVID-19	35/807	186/5862	0.030910472	0.344278703	50528/54131/27207/20084/75617/78294/11421/16195/17174/72512/67115/15139/22034/67427/20104/19934/19988/27050/20102/225215/16785/15945/19896/68028/12273/66483/100043813/19094/100503670/19946/19899/20054/22121/100040298/57294	35
mmu05145	Toxoplasmosis	21/807	101/5862	0.032295269	0.3477124	21939/18126/12265/15001/17087/16782/23928/22034/21947/14681/14960/15000/14998/15002/19094/14969/381091/14961/14999/15511/193740	21
mmu04950	Maturity onset diabetes of the young	5/807	14/5862	0.033492375	0.348968935	30942/21410/18508/18088/378435	5
mmu05321	Inflammatory bowel disease	11/807	45/5862	0.03790601	0.37163003	15001/16186/14960/15000/14998/15002/209590/14969/381091/14961/14999	11
mmu00062	Fatty acid elongation	7/807	24/5862	0.037968393	0.37163003	74559/66775/83603/170439/217698/30963/26897	7
mmu04974	Protein digestion and absorption	16/807	74/5862	0.041495691	0.385699929	12822/12842/12818/12821/12836/12839/12814/110891/12826/245026/12835/12829/12837/213945/232680/665033	16

mmu04723	Retrograde endocannabinoid signaling	25/807	128/5862	0.04179411	0.385699929	68198/14401/14394/432530/14406/14802/227197/12292/16519/66046/14397/140919/68197/11513/14681/14697/66066/14402/14073/22348/68342/12801/12288/19094/110886	25
mmu04151	PI3K-Akt signaling pathway	48/807	278/5862	0.052973579	0.475290721	12842/16400/12447/19116/16590/21687/12567/17295/12915/22629/22027/14180/11600/16419/12575/22339/56716/72930/18590/12839/16782/23928/13645/21923/20104/108079/18127/14254/12478/16186/12826/14697/66066/81877/16399/21825/245026/14924/12835/16002/13640/18205/231991/14184/16367/16542/12829/665033	48
mmu04022	cGMP-PKG signaling pathway	26/807	139/5862	0.060639797	0.529369036	17540/12915/432530/14463/12292/18127/19092/110891/320707/16532/11513/22068/11551/11554/17888/54611/11549/18386/11548/12288/231991/140781/242202/16367/14886/98932	26
mmu05140	Leishmaniasis	13/807	61/5862	0.068524746	0.559571598	16415/18126/15001/22034/14960/15000/14998/15002/19094/14969/381091/14961/14999	13
mmu04726	Serotonergic synapse	16/807	79/5862	0.069389695	0.559571598	11684/14401/15566/12292/16519/230459/14681/14697/66066/22063/14402/15560/19224/15557/12288/16508	16
mmu00591	Linoleic acid metabolism	4/807	12/5862	0.071029212	0.559571598	26565/230459/665270/18784	4
mmu00730	Thiamine metabolism	4/807	12/5862	0.071029212	0.559571598	11636/18041/11639/229949	4
mmu04122	Sulfur relay system	3/807	8/5862	0.085363022	0.644757546	18041/233189/66965	3
mmu03030	DNA replication	8/807	34/5862	0.085834596	0.644757546	17216/68240/18972/17217/14156/69745/19891/381760	8
mmu03040	Spliceosome	23/807	128/5862	0.105477643	0.77430179	67465/20639/68988/67996/53817/69878/68092/225027/28000/73666/17149/56031/57905/50783/66055/76522/15382/68011/229279/20826/15511/66373/193740	23
mmu00590	Arachidonic acid metabolism	7/807	30/5862	0.108312047	0.777439803	11684/26565/230459/665270/21391/18784/19224	7
mmu04724	Glutamatergic synapse	18/807	97/5862	0.111550357	0.783277508	20511/76257/432530/14823/26557/14802/16519/140919/11513/14681/110637/14697/66066/22063/210274/12288/14806/268934	18
mmu00220	Arginine biosynthesis	4/807	15/5862	0.140802587	0.947484078	18126/18127/18125/18416	4
mmu00592	alpha-Linolenic acid metabolism	4/807	15/5862	0.140802587	0.947484078	26565/56473/665270/18784	4
mmu04970	Salivary secretion	12/807	63/5862	0.149380653	0.965045727	71395/432530/12494/19092/18125/320707/11513/11554/11549/12671/11548/74180	12
mmu00650	Butanoate metabolism	5/807	21/5862	0.152375641	0.965045727	15360/268860/14415/64059/208715	5
mmu03430	Mismatch repair	5/807	21/5862	0.152375641	0.965045727	68240/18972/69745/19891/381760	5
mmu05032	Morphine addiction	14/807	77/5862	0.166019166	0.99997156	14401/14394/432530/14406/16519/14397/11513/14681/14697/66066/14402/22348/54611/110886	14
mmu05166	Human T-cell leukemia virus 1 infection	34/807	210/5862	0.17370912	0.99997156	16415/16818/12502/12447/19385/12567/17222/21374/12915/14991/21939/21423/432530/12575/12504/15001/16178/50883/16186/11513/23871/12500/14960/15000/14998/69957/15002/231991/110196/14969/381091/72049/14961/14999	34
mmu00900	Terpenoid backbone biosynthesis	5/807	22/5862	0.176238909	0.99997156	19671/15360/319554/110196/208715	5
mmu04911	Insulin secretion	12/807	66/5862	0.189663758	0.99997156	12915/432530/12292/11517/16532/11513/19339/116838/20927/12671/12288/231991	12
mmu04972	Pancreatic secretion	12/807	66/5862	0.189663758	0.99997156	432530/26565/80718/319229/12494/320707/11513/22063/18784/12671/12722/232680	12

mmu05133	Pertussis	10/807	54/5862	0.20146769	0.99997156	16415/54131/18126/17087/15139/22034/66824/15900/106759/19094	10
mmu04350	TGF-beta signaling pathway	12/807	67/5862	0.204111864	0.99997156	268977/15903/13179/11477/12156/55994/18741/12163/17130/16324/21825/11481	12
mmu05169	Epstein-Barr virus infection	31/807	195/5862	0.216992344	0.99997156	12502/12447/107986/54131/14128/12503/12567/14991/22123/21939/66413/67089/12575/18037/15001/12902/22034/12478/12500/15945/14960/15000/14998/15002/215446/19094/14969/17060/381091/14961/14999	31
mmu05219	Bladder cancer	7/807	36/5862	0.218977972	0.99997156	12567/72962/12575/22339/13645/21825/14184	7
mmu05412	Arrhythmogenic right ventricular cardiomyopathy	12/807	68/5862	0.219026556	0.99997156	21414/16400/16419/12292/16905/110891/16399/67451/13511/12288/21415/54376	12
mmu04934	Cushing syndrome	20/807	121/5862	0.220291705	0.99997156	21414/12447/12567/14365/22413/12915/22411/23805/432530/22408/12575/12292/11513/13070/69544/16527/14371/12288/231991/21415	20
mmu00250	Alanine, aspartate and glutamate metabolism	6/807	30/5862	0.223986771	0.99997156	14584/11564/27053/194237/268860/14415	6
mmu04550	Signaling pathways regulating pluripotency of stem cells	17/807	102/5862	0.232881628	0.99997156	21414/15903/14365/22413/22411/23805/21423/16195/22408/11477/18508/55994/16324/14371/19094/14184/11481	17
mmu03008	Ribosome biogenesis in eukaryotes	12/807	69/5862	0.234378132	0.99997156	52530/67619/14791/69237/21453/97112/245474/102462/110816/225348/14113/20826	12
mmu01212	Fatty acid metabolism	10/807	56/5862	0.235194973	0.99997156	74559/109729085/328845/56473/66775/83603/20249/170439/30963/14979	10
mmu03410	Base excision repair	6/807	31/5862	0.247935723	0.99997156	18972/14156/69745/11545/72774/71726	6
mmu00230	Purine metabolism	19/807	117/5862	0.252455954	0.99997156	27369/432530/50773/18103/66358/11564/224794/108147/11636/11639/67054/11513/229949/215446/54611/242202/23918/16434/328099	19
mmu05414	Dilated cardiomyopathy	13/807	77/5862	0.255927581	0.99997156	16400/432530/16419/12292/16905/110891/11513/16399/11554/17888/12288/140781/54376	13
mmu04020	Calcium signaling pathway	28/807	179/5862	0.259585789	0.99997156	17295/432530/18436/18126/14180/22339/15566/18590/12292/13645/18127/12494/18125/14254/21336/110891/320707/11513/70086/11554/15560/11549/12671/11548/12288/14184/20698/16542	28
mmu00330	Arginine and proline metabolism	6/807	32/5862	0.272567731	0.99997156	20810/18126/66194/69051/18127/18125	6
mmu04913	Ovarian steroidogenesis	6/807	32/5862	0.272567731	0.99997156	432530/230459/11513/13070/217698/26897	6
mmu04623	Cytosolic DNA-sensing pathway	8/807	45/5862	0.273685556	0.99997156	54131/245841/72512/26388/66824/26939/15945/20016	8
mmu05410	Hypertrophic cardiomyopathy	12/807	72/5862	0.282728671	0.99997156	16400/11421/16419/12292/16905/108079/110891/16399/17888/12288/140781/54376	12
mmu03440	Homologous recombination	7/807	39/5862	0.285265069	0.99997156	68240/18972/69745/71711/19891/381760/268465	7
mmu04510	Focal adhesion	28/807	182/5862	0.29052872	0.99997156	12842/16400/17295/216148/170736/16419/22339/18590/12839/16782/23928/13645/21923/14254/22793/18481/12826/12927/81877/16399/241656/21825/245026/12835/16542/12829/98932/665033	28
mmu00532	Glycosaminoglycan b–osynthesis - chondroitin sulfate / dermatan sulfate	4/807	20/5862	0.292893397	0.99997156	218271/234356/53374/78923	4
mmu05164	Influenza A	20/807	129/5862	0.317947955	0.99997156	50528/54131/12567/12265/15001/66679/66824/270151/15945/14960/18854/15000/14998/15002/106759/110196/14969/381091/14961/14999	20

mmu04924	Renin secretion	9/807	54/5862	0.322298397	0.99997156	11421/12292/19092/11517/11554/16518/54611/12288/12722	9
mmu00290	Valine, leucine and isoleucine biosynthesis	1/807	3/5862	0.358804384	0.99997156	12035	1
mmu04927	Cortisol synthesis and secretion	8/807	49/5862	0.360001843	0.99997156	12915/432530/12292/11513/13070/16527/12288/231991	8
mmu04390	Hippo signaling pathway	20/807	133/5862	0.370883682	0.99997156	16415/21414/14365/22413/22411/11799/22629/23805/72293/22408/72930/12156/97064/93960/12163/241324/101543/14371/21676/21415	20
mmu05226	Gastric cancer	18/807	119/5862	0.371232091	0.99997156	21414/12447/107986/20423/14365/17295/22413/20183/22411/23805/216148/14180/22408/12575/13645/12557/14371/21415	18
mmu04611	Platelet activation	17/807	112/5862	0.371255061	0.99997156	12842/432530/18436/20619/18441/18127/19092/21391/11513/16399/70839/19224/14729/14723/14724/19094/243816	17
mmu00513	Various types of N-glycan biosynthesis	6/807	36/5862	0.375293452	0.99997156	103963/68292/208624/140481/330406/207958	6
mmu04926	Relaxin signaling pathway	16/807	107/5862	0.401580354	0.99997156	12842/12915/216148/432530/18126/22339/18127/18125/12826/11513/14681/14697/66066/231991/19094/12829	16
mmu04725	Cholinergic synapse	14/807	93/5862	0.403118936	0.99997156	16536/432530/12292/16519/11513/14681/14697/66066/16518/16515/16520/12671/12288/231991	14
mmu00061	Fatty acid biosynthesis	3/807	17/5862	0.421176307	0.99997156	109729085/328845/14979	3
mmu00534	Glycosaminoglycan b–osynthesis - heparan sulfate / heparin	4/807	24/5862	0.425409636	0.99997156	218271/83398/195646/50786	4
mmu00510	N-Glycan biosynthesis	7/807	45/5862	0.427136913	0.99997156	20440/103963/68292/208624/140481/68563/207958	7
mmu05205	Proteoglycans in cancer	25/807	173/5862	0.429369879	0.99997156	12842/20423/83383/13345/14365/17295/22413/22411/20971/13179/66911/16419/22408/12575/22339/20104/13043/109676/15442/21825/16002/14371/19094/66922/16542	25
mmu04660	T cell receptor signaling pathway	14/807	95/5862	0.435965364	0.99997156	16818/12502/12503/12567/12504/18037/12487/21947/18481/12500/241656/12526/19094/12525	14
mmu04064	NF-kappa B signaling pathway	13/807	88/5862	0.437920068	0.99997156	16818/13608/21939/17087/21934/11545/22034/21947/105844/106759/13607/17060/72049	13
mmu05222	Small cell lung cancer	13/807	88/5862	0.437920068	0.99997156	16400/12447/107986/12567/20183/18126/12575/16782/23928/22034/12826/16399/12829	13
mmu04916	Melanogenesis	11/807	74/5862	0.441995767	0.99997156	21414/16590/14365/22413/22411/432530/22408/11513/14681/14371/21415	11
mmu04392	Hippo signal–ng pathway - multiple species	4/807	25/5862	0.457892972	0.99997156	97064/101543/329628/21676	4
mmu00603	Glycosphingolipid b–osynthesis - globo and isoglobo series	2/807	11/5862	0.459721216	0.99997156	26879/14348	2
mmu00601	Glycosphingolipid b–osynthesis - lacto and neolacto series	3/807	19/5862	0.497098554	0.99997156	108105/26879/14348	3
mmu05146	Amoebiasis	11/807	77/5862	0.497485849	0.99997156	16415/12842/432530/18126/16178/16782/23928/12479/12826/226421/12829	11
mmu04015	Rap1 signaling pathway	25/807	179/5862	0.502477736	0.99997156	16415/16590/21687/17295/432530/14026/14180/11600/22339/18590/18441/13645/14254/11513/14681/12927/16399/21825/12801/14924/14293/13640/19094/14184/16542	25

mmu05235	PD-L1 expression and PD-1 checkpoint pathway in cancer	12/807	85/5862	0.510497672	0.99997156	16818/12502/12503/12504/18037/12487/22034/13645/12500/106759/19094/11682	12
mmu03460	Fanconi anemia pathway	7/807	49/5862	0.520701401	0.99997156	68240/50505/71711/230484/19891/268465/52864	7
mmu04622	RIG-I-like receptor signaling pathway	7/807	49/5862	0.520701401	0.99997156	54131/72512/22034/270151/225913/15945/19094	7
mmu03420	Nucleotide excision repair	6/807	42/5862	0.528774813	0.99997156	107986/68240/18972/50505/69745/19891	6
mmu04261	Adrenergic signaling in cardiomyocytes	17/807	123/5862	0.533420171	0.99997156	12915/432530/72930/12292/110891/320707/11513/20271/11554/17888/11549/11548/12288/231991/140781/19094/54376	17
mmu04750	Inflammatory mediator regulation of TRP channels	11/807	79/5862	0.533725325	0.99997156	11684/193034/63873/432530/14063/230459/11513/171382/15560/246788/19094	11
mmu04145	Phagosome	19/807	138/5862	0.53815195	0.99997156	16415/14991/545486/16419/15001/17533/18125/170786/14960/15000/14998/21825/15002/226421/14969/381091/22144/14961/14999	19
mmu00830	Retinol metabolism	5/807	35/5862	0.538528135	0.99997156	19378/56847/13082/11761/20148	5
mmu00280	Valine, leucine and isoleucine degradation	7/807	50/5862	0.543261534	0.99997156	15108/15360/12035/268860/11761/64059/208715	7
mmu04370	VEGF signaling pathway	7/807	50/5862	0.543261534	0.99997156	216148/22339/27371/18127/19094/20698/16542	7
mmu04977	Vitamin digestion and absorption	2/807	13/5862	0.551822686	0.99997156	65969/665270	2
mmu05224	Breast cancer	16/807	117/5862	0.553053602	0.99997156	21414/107986/16590/12567/14365/22413/22411/23805/216148/14180/22408/12575/13645/18667/14371/21415	16
mmu05216	Thyroid cancer	5/807	36/5862	0.564856309	0.99997156	21414/107986/20183/12575/21415	5
mmu03320	PPAR signaling pathway	7/807	51/5862	0.565369278	0.99997156	20183/328845/56473/26458/15360/20249/208715	7
mmu04613	Neutrophil extracellular trap formation	21/807	155/5862	0.568149949	0.99997156	16415/15139/16399/14293/12273/14723/14289/19094/326619/319155/319184/170787/15267/319164/319157/665622/100041230/665596/14294/319187/319189	21
mmu04014	Ras signaling pathway	25/807	185/5862	0.573835412	0.99997156	16590/21687/17295/216148/14180/11600/26565/22339/15566/18590/13645/14254/18481/23871/14697/66066/241656/18784/16002/13640/18205/320484/14184/66922/16542	25
mmu00970	Aminoacyl-tRNA biosynthesis	6/807	44/5862	0.576646088	0.99997156	15115/22321/104458/110960/67417/85305	6
mmu04923	Regulation of lipolysis in adipocytes	6/807	44/5862	0.576646088	0.99997156	432530/19092/11513/11554/19224/16367	6
mmu00564	Glycerophospholipid metabolism	11/807	82/5862	0.586304605	0.99997156	66358/210992/26565/14555/13139/665270/74596/236899/18784/331374/381925	11
mmu04713	Circadian entrainment	11/807	82/5862	0.586304605	0.99997156	432530/14802/16519/19092/18125/11517/11513/14681/14697/66066/12288	11
mmu05418	Fluid shear stress and atherosclerosis	15/807	112/5862	0.588452582	0.99997156	15368/63873/20971/22027/22339/18590/16178/11477/108079/18127/14872/16399/19094/11481/16542	15
mmu00440	Phosphonate and phosphinate metabolism	1/807	6/5862	0.588969032	0.99997156	236899	1
mmu04062	Chemokine signaling pathway	19/807	142/5862	0.591562985	0.99997156	20308/216148/432530/20290/57266/12765/57349/56744/11513/12927/14697/66066/15945/12775/12458/12777/12145/12772/23832	19
mmu01210	2-Oxocarboxylic acid metabolism	2/807	14/5862	0.593532368	0.99997156	170718/12035	2

mmu04915	Estrogen signaling pathway	13/807	98/5862	0.602812335	0.99997156	12915/22027/216148/432530/16519/18127/11513/14681/18667/231991/16665/15511/193740	13
mmu04310	Wnt signaling pathway	17/807	128/5862	0.603634723	0.99997156	21414/14365/22413/22411/23805/72293/22408/20671/192199/56505/20377/93960/228770/106042/319478/14371/21415	17
mmu04260	Cardiac muscle contraction	9/807	69/5862	0.62296222	0.99997156	66445/12292/110891/66694/17888/12288/333182/140781/54376	9
mmu04925	Aldosterone synthesis and secretion	10/807	77/5862	0.629721523	0.99997156	12915/432530/12292/320707/11513/13070/223604/16527/12288/231991	10
mmu05142	Chagas disease	11/807	85/5862	0.636109803	0.99997156	12502/12503/432530/11421/18126/72930/22034/14681/12500/106759/19094	11
mmu03022	Basal transcription factors	5/807	39/5862	0.638892554	0.99997156	21374/21343/24074/108143/14886	5
mmu04978	Mineral absorption	5/807	39/5862	0.638892554	0.99997156	15368/11979/110891/320707/72027	5
mmu00750	Vitamin B6 metabolism	1/807	7/5862	0.645612132	0.99997156	11761	1
mmu00983	Drug–metabolism - other enzymes	7/807	55/5862	0.648381151	0.99997156	22271/18103/22017/72962/14872/23918/16434	7
mmu05223	Non-small cell lung cancer	9/807	71/5862	0.658077349	0.99997156	107986/12567/17295/20183/12575/16574/13645/11682/16572	9
mmu05165	Human papillomavirus infection	38/807	290/5862	0.658261369	0.99997156	21414/12842/16400/12447/54131/12567/14365/22413/21374/22411/14991/17305/23805/16419/22408/12575/22339/72930/12839/16782/23928/13645/21923/74164/12826/81877/16399/21825/245026/14924/106759/12835/14371/231991/21415/433586/12829/665033	38
mmu05152	Tuberculosis	18/807	140/5862	0.661512142	0.99997156	16415/18126/12265/15001/16149/17533/22034/170786/14960/15000/14998/15002/19094/14969/20698/381091/14961/14999	18
mmu01230	Biosynthesis of amino acids	7/807	56/5862	0.667607331	0.99997156	66194/69051/170718/27053/12035/18416/328099	7
mmu05215	Prostate cancer	11/807	88/5862	0.682588756	0.99997156	50528/21414/12447/22027/12575/18590/16178/13645/13876/231991/21415	11
mmu00760	Nicotinate and nicotinamide metabolism	4/807	33/5862	0.684655912	0.99997156	50773/12494/11872/11761	4
mmu05225	Hepatocellular carcinoma	18/807	142/5862	0.685589719	0.99997156	21414/20587/107986/15368/12567/14365/17295/22413/22411/23805/216148/22408/83797/12575/14872/16002/14371/21415	18
mmu01523	Antifolate resistance	3/807	25/5862	0.689011687	0.99997156	11684/108147/26357	3
mmu00740	Riboflavin metabolism	1/807	8/5862	0.694457736	0.99997156	11433	1
mmu00670	One carbon pool by folate	2/807	17/5862	0.700972415	0.99997156	108147/665563	2
mmu05202	Transcriptional misregulation in cancer	19/807	152/5862	0.712193108	0.99997156	50528/107986/18505/17295/18507/20183/21939/18612/21423/12575/18590/16178/18509/14254/20474/18854/13876/20471/15364	19
mmu04270	Vascular smooth muscle contraction	12/807	98/5862	0.714076373	0.99997156	17540/432530/26565/12292/109624/16532/11513/18784/11549/11548/12288/98932	12
mmu05218	Melanoma	7/807	59/5862	0.721269128	0.99997156	107986/12567/17295/14180/12575/18590/13645	7
mmu04540	Gap junction	9/807	75/5862	0.722320837	0.99997156	545486/432530/18590/13645/19092/11513/11554/15560/22144	9
mmu04961	Endocrine and other factor-regulated calcium reabsorption	5/807	43/5862	0.724545268	0.99997156	110891/320707/19228/103967/16591	5
mmu05034	Alcoholism	18/807	146/5862	0.730741578	0.99997156	12915/216148/14681/14697/66066/231991/326619/319155/319184/170787/15267/319164/319157/665622/100041230/665596/319187/319189	18

mmu00130	Ubiquinone and other terpenoid-quinone biosynthesis	1/807	9/5862	0.736578099	0.99997156	52064	1
mmu00430	Taurine and hypotaurine metabolism	1/807	9/5862	0.736578099	0.99997156	14415	1
mmu00480	Glutathione metabolism	5/807	44/5862	0.743506701	0.99997156	110175/20810/11758/14872/66116	5
mmu05144	Malaria	5/807	44/5862	0.743506701	0.99997156	16415/17295/21939/21947/21825	5
mmu00565	Ether lipid metabolism	4/807	36/5862	0.750169936	0.99997156	210992/26565/665270/18784	4
mmu04929	GnRH secretion	6/807	53/5862	0.756554577	0.99997156	15166/15165/12292/16519/22063/12288	6
mmu00770	Pantothenate and CoA biosynthesis	2/807	19/5862	0.7586632	0.99997156	71743/12035	2
mmu04710	Circadian rhythm	3/807	28/5862	0.761894974	0.99997156	18143/108079/225998	3
mmu04662	B cell receptor signaling pathway	9/807	78/5862	0.764941463	0.99997156	12518/18037/68713/12902/12517/12483/12478/15985/17060	9
mmu04630	JAK-STAT signaling pathway	13/807	110/5862	0.765268431	0.99997156	19116/50931/17480/16195/12575/14199/18590/13645/12804/16186/209590/239114/11761	13
mmu04115	p53 signaling pathway	7/807	62/5862	0.768721348	0.99997156	12447/107986/12567/12575/230784/50883/21825	7
mmu04979	Cholesterol metabolism	4/807	37/5862	0.769536212	0.99997156	11818/27413/16891/20661	4
mmu04066	HIF-1 signaling pathway	11/807	95/5862	0.776305516	0.99997156	15368/21687/18126/68263/11600/12575/22339/13645/20104/18127/14254	11
mmu04110	Cell cycle	13/807	112/5862	0.78728774	0.99997156	12544/12447/17216/12567/17222/22629/140557/17217/12575/50883/12577/22137/69957	13
mmu04728	Dopaminergic synapse	13/807	112/5862	0.78728774	0.99997156	12915/72930/14802/16574/16519/14681/14697/66066/12288/231991/19094/20265/16572	13
mmu04213	Longevity regulat–ng pathway - multiple species	6/807	55/5862	0.787764955	0.99997156	432530/108079/11513/16367/15511/193740	6
mmu04921	Oxytocin signaling pathway	15/807	128/5862	0.78794842	0.99997156	432530/12575/12292/16519/108079/18127/12494/11513/14681/16518/16515/16520/12288/54376/98932	15
mmu00561	Glycerolipid metabolism	5/807	47/5862	0.794571067	0.99997156	13139/225913/16891/331374/381925	5
mmu04072	Phospholipase D signaling pathway	15/807	129/5862	0.797754226	0.99997156	16590/216148/432530/14823/14126/13139/18590/12765/13645/11513/103967/66922/20698/331374/268934	15
mmu05206	MicroRNAs in cancer	17/807	145/5862	0.798714287	0.99997156	12447/15368/14365/17295/23805/22061/12575/22339/18590/21923/14086/81877/21825/13640/14184/16367/15364	17
mmu03020	RNA polymerase	3/807	30/5862	0.802328272	0.99997156	245841/26939/20016	3
mmu04130	SNARE interactions in vesicular transport	3/807	30/5862	0.802328272	0.99997156	53330/20619/74732	3
mmu00920	Sulfur metabolism	1/807	11/5862	0.804215884	0.99997156	211389	1
mmu00514	Other types of O-glycan biosynthesis	4/807	39/5862	0.804713243	0.99997156	17305/108760/20440/233733	4
mmu02010	ABC transporters	5/807	48/5862	0.809709958	0.99997156	27413/26357/20927/74591/320631	5
mmu04371	Apelin signaling pathway	13/807	115/5862	0.817511282	0.99997156	21780/432530/18126/20104/108079/18127/18125/110891/11513/14697/66066/66922/20698	13

mmu05203	Viral carcinogenesis	23/807	195/5862	0.819948111	0.99997156	12447/20259/54131/19385/12567/21374/12915/14991/22629/16195/12575/66973/231991/326619/319155/319184/170787/319157/665622/100041230/665596/319187/319189	23
mmu04930	Type II diabetes mellitus	4/807	40/5862	0.820596996	0.99997156	20927/378435/12288/16367	4
mmu00040	Pentose and glucuronate interconversions	2/807	22/5862	0.827053717	0.99997156	20322/16591	2
mmu04657	IL-17 signaling pathway	6/807	58/5862	0.828728405	0.99997156	22027/22034/15945/239114/19094/74180	6
mmu05213	Endometrial cancer	6/807	58/5862	0.828728405	0.99997156	21414/107986/23805/12575/13645/21415	6
mmu05207	Chemical car–inogenesis - receptor activation	17/807	149/5862	0.832825883	0.99997156	12355/12915/20183/11799/22027/432530/14180/22408/22339/12292/13645/11513/18667/14872/11554/12288/231991	17
mmu01521	EGFR tyrosine kinase inhibitor resistance	8/807	76/5862	0.839950166	0.99997156	17295/216148/22339/18590/13645/20104/14184/16542	8
mmu04714	Thermogenesis	24/807	206/5862	0.841621063	0.99997156	20587/68198/66043/432530/66445/83797/56716/227197/66046/68493/20104/108079/19092/68197/11513/12163/66694/70673/68342/12801/333182/231991/19094/67942	24
mmu01240	Biosynthesis of cofactors	14/807	126/5862	0.843018612	0.99997156	71743/18103/11564/11636/18041/11639/665563/12035/67417/209176/229949/98256/52064/20148	14
mmu04975	Fat digestion and absorption	2/807	23/5862	0.845649884	0.99997156	26565/18784	2
mmu03015	mRNA surveillance pathway	9/807	85/5862	0.845792503	0.99997156	229512/53817/68092/72930/17149/108062/68219/17690/78428	9
mmu03013	Nucleocytoplasmic transport	11/807	102/5862	0.848634765	0.99997156	110750/53817/68092/73666/66679/97112/17149/269113/234865/76582/78428	11
mmu00240	Pyrimidine metabolism	5/807	51/5862	0.849831984	0.99997156	22271/50773/18103/72962/215446	5
mmu00380	Tryptophan metabolism	3/807	33/5862	0.852052434	0.99997156	209176/98256/11761	3
mmu04721	Synaptic vesicle cycle	6/807	60/5862	0.852350547	0.99997156	20511/140919/19339/22348/103967/208898	6
mmu03450	Non-homologous end-joining	1/807	13/5862	0.854502498	0.99997156	14156	1
mmu04928	Parathyroid hormone synthesis, secretion and action	9/807	86/5862	0.85531664	0.99997156	12915/20183/432530/12575/11513/19228/231991/16591/17938	9
mmu04931	Insulin resistance	10/807	95/5862	0.860455547	0.99997156	21428/14584/26458/108079/18127/244416/228966/231991/16367/105651	10
mmu04918	Thyroid hormone synthesis	5/807	52/5862	0.861543768	0.99997156	12915/22027/432530/11513/231991	5
mmu04530	Tight junction	15/807	137/5862	0.864718494	0.99997156	72058/12567/14463/72930/74192/12479/54357/108079/13043/56332/231830/16456/14924/22144/98932	15
mmu05134	Legionellosis	5/807	53/5862	0.872480142	0.99997156	16415/66824/67160/15511/193740	5
mmu03050	Proteasome	4/807	44/5862	0.873795126	0.99997156	22123/66413/67089/26441	4
mmu00500	Starch and sucrose metabolism	2/807	25/5862	0.877489217	0.99997156	70974/232714	2
mmu00563	Glycosylphosphatidylinositol (GPI)-anchor biosynthesis	2/807	25/5862	0.877489217	0.99997156	66268/56703	2

mmu03060	Protein export	2/807	25/5862	0.877489217	0.99997156	66624/93757	2
mmu04935	Growth hormone synthesis, secretion and action	10/807	98/5862	0.884523877	0.99997156	12915/216148/432530/12292/11513/12927/12288/231991/19094/16367	10
mmu04211	Longevity regulating pathway	8/807	81/5862	0.886178018	0.99997156	12915/432530/108079/230784/11513/231991/16367/16591	8
mmu00620	Pyruvate metabolism	3/807	36/5862	0.890503171	0.99997156	68263/107029/68738	3
mmu00140	Steroid hormone biosynthesis	2/807	26/5862	0.891029548	0.99997156	13070/14979	2
mmu00512	Mucin type O-glycan biosynthesis	2/807	26/5862	0.891029548	0.99997156	108760/233733	2
mmu00340	Histidine metabolism	1/807	15/5862	0.891884966	0.99997156	140483	1
mmu04614	Renin-angiotensin system	1/807	15/5862	0.891884966	0.99997156	11421	1
mmu00020	Citrate cycle (TCA cycle)	2/807	27/5862	0.903169072	0.99997156	68263/170718	2
mmu00410	beta-Alanine metabolism	2/807	27/5862	0.903169072	0.99997156	268860/14415	2
mmu05143	African trypanosomiasis	2/807	27/5862	0.903169072	0.99997156	14063/209176	2
mmu00100	Steroid biosynthesis	1/807	16/5862	0.906806948	0.99997156	16987	1
mmu04964	Proximal tubule bicarbonate reclamation	1/807	16/5862	0.906806948	0.99997156	76257	1
mmu05162	Measles	12/807	119/5862	0.909955499	0.99997156	12502/12447/54131/12567/27218/12487/22034/16186/170786/12500/15511/193740	12
mmu04810	Regulation of actin cytoskeleton	20/807	186/5862	0.910235796	0.99997156	16415/16400/20259/23805/14180/16419/14163/18590/74192/50884/13645/18481/12927/16399/241656/12671/19241/14184/66922/98932	20
mmu00051	Fructose and mannose metabolism	2/807	28/5862	0.91403631	0.99997156	20322/225913	2
mmu00640	Propanoate metabolism	2/807	28/5862	0.91403631	0.99997156	68738/268860	2
mmu05211	Renal cell carcinoma	6/807	67/5862	0.914870064	0.99997156	17295/12575/22339/18481/23871/241656	6
mmu05214	Glioma	6/807	67/5862	0.914870064	0.99997156	107986/12567/216148/12575/18590/13645	6
mmu00053	Ascorbate and aldarate metabolism	1/807	17/5862	0.919671607	0.99997156	16591	1
mmu05168	Herpes simplex virus 1 infection	39/807	342/5862	0.920143885	0.99997156	54131/217715/14991/67996/225027/15001/72512/16149/15139/22034/22696/231866/78921/22751/69020/14960/18854/15000/14998/15002/56220/330788/101197/233893/106759/22719/237775/233168/235047/14969/433804/232855/381091/232966/14961/665211/408065/14999/100233208	39
mmu04620	Toll-like receptor signaling pathway	7/807	77/5862	0.920717584	0.99997156	54131/21939/17087/22034/15945/106759/19094	7
mmu05020	Prion disease	26/807	238/5862	0.922021006	0.99997156	68198/66043/12915/545486/22123/66413/67089/66445/227197/12292/16574/15139/66046/68197/26441/66694/68342/12288/333182/231991/19094/67942/22144/16572/15511/193740	26
mmu0421–	Apoptosis - multiple species	2/807	29/5862	0.923750994	0.99997156	11799/18952	2

mmu04971	Gastric acid secretion	5/807	59/5862	0.923792143	0.99997156	432530/11513/16518/12671/16517	5
mmu04330	Notch signaling pathway	4/807	50/5862	0.927938285	0.99997156	17305/80904/68318/433586	4
mmu04920	Adipocytokine signaling pathway	5/807	60/5862	0.930286833	0.99997156	20183/18037/328845/108079/16367	5
mmu04976	Bile secretion	5/807	60/5862	0.930286833	0.99997156	432530/319229/27413/26357/11513	5
mmu00531	Glycosaminoglycan degradation	1/807	18/5862	0.930762285	0.99997156	15442	1
mmu00790	Folate biosynthesis	1/807	18/5862	0.930762285	0.99997156	110391	1
mmu04670	Leukocyte transendothelial migration	8/807	88/5862	0.931798784	0.99997156	16415/69524/74734/12927/21838/16456/19094/98932	8
mmu04960	Aldosterone-regulated sodium reabsorption	2/807	30/5862	0.932424045	0.99997156	20276/16367	2
mmu04024	cAMP signaling pathway	16/807	158/5862	0.933217692	0.99997156	20682/15166/432530/14802/12292/11517/320707/11513/330953/11554/54611/15557/12288/231991/66922/98932	16
mmu05100	Bacterial invasion of epithelial cells	6/807	70/5862	0.933671283	0.99997156	17295/216148/74192/13043/12927/103967	6
mmu05230	Central carbon metabolism in cancer	5/807	61/5862	0.936283556	0.99997156	16590/17295/68263/14184/100126824	5
mmu04068	FoxO signaling pathway	11/807	116/5862	0.937832603	0.99997156	12575/26557/13645/108079/57436/13611/13609/329934/19094/16367/15469	11
mmu03018	RNA degradation	6/807	71/5862	0.939066987	0.99997156	57316/69639/50783/227715/76522/66373	6
mmu0413–	Autophagy - other	2/807	31/5862	0.940157732	0.99997156	56716/57436	2
mmu00515	Mannose type O-glycan biosynthesis	1/807	19/5862	0.940323345	0.99997156	14348	1
mmu04114	Oocyte meiosis	9/807	99/5862	0.941225992	0.99997156	12447/17222/22629/432530/140557/11513/18667/69957/19094	9
mmu04210	Apoptosis	12/807	126/5862	0.942746925	0.99997156	13804/11799/18952/13041/11545/69601/229445/16905/13368/13032/12123/22144	12
mmu04740	Olfactory transduction	6/807	72/5862	0.944068592	0.99997156	19092/110891/243634/258808/258189/259081	6
mmu04730	Long-term depression	4/807	53/5862	0.946239171	0.99997156	19092/18125/14681/14687	4
mmu05210	Colorectal cancer	7/807	82/5862	0.946882571	0.99997156	21414/107986/11799/23805/12575/13645/21415	7
mmu05221	Acute myeloid leukemia	5/807	64/5862	0.951595458	0.99997156	21414/16590/18715/18854/21415	5
mmu00600	Sphingolipid metabolism	3/807	44/5862	0.953081189	0.99997156	70059/230379/20698	3
mmu00860	Porphyrin and chlorophyll metabolism	2/807	33/5862	0.953174556	0.99997156	15368/67417	2
mmu04650	Natural killer cell mediated cytotoxicity	8/807	93/5862	0.953754078	0.99997156	16415/16818/15896/20400/12503/12506/216148/16638	8
mmu05132	Salmonella infection	24/807	232/5862	0.953883055	0.99997156	21414/110750/545486/22027/17087/240752/74192/16574/50884/22034/80743/74734/18705/27050/66824/18481/71999/80837/226421/19094/21415/22144/98932/16572	24
mmu04010	MAPK signaling pathway	26/807	249/5862	0.954668703	0.99997156	19060/65964/16590/21687/17295/14180/11600/22339/18590/12292/22034/13537/13645/14254/26411/16002/13640/18205/12288/19094/14184/66922/16542/54376/15511/193740	26

mmu04933	AGE-RAGE signaling pathway in diabetic complications	7/807	84/5862	0.95498373	0.99997156	12842/12567/22339/18127/12826/19094/12829	7
mmu05031	Amphetamine addiction	4/807	55/5862	0.955963976	0.99997156	12915/14802/12288/231991	4
mmu04071	Sphingolipid signaling pathway	10/807	112/5862	0.956184603	0.99997156	70059/72930/14126/18127/230379/13611/13609/18386/19094/20698	10
mmu04520	Adherens junction	5/807	66/5862	0.959859308	0.99997156	14158/21414/17295/99167/21415	5
mmu00030	Pentose phosphate pathway	1/807	22/5862	0.961795173	0.99997156	328099	1
mmu01522	Endocrine resistance	7/807	86/5862	0.961957882	0.99997156	12567/216148/432530/12575/27413/11513/19094	7
mmu04340	Hedgehog signaling pathway	3/807	46/5862	0.962363576	0.99997156	20423/269878/68525	3
mmu0413–	Mitophagy - animal	5/807	67/5862	0.963487473	0.99997156	78294/57436/233204/226421/66922	5
mmu04914	Progesterone-mediated oocyte maturation	6/807	77/5862	0.963966563	0.99997156	17222/432530/11513/18667/69957/19094	6
mmu05415	Diabetic cardiomyopathy	18/807	186/5862	0.965190205	0.99997156	12842/68198/66043/14584/11421/68263/66445/227197/11545/66046/18127/68197/66694/68342/333182/19094/16367/67942	18
mmu00350	Tyrosine metabolism	1/807	23/5862	0.967074505	0.99997156	11761	1
mmu05135	Yersinia infection	11/807	125/5862	0.967203347	0.99997156	16818/54131/12504/74192/22034/66824/12927/106759/12526/19094/12525	11
mmu00982	Drug–metabolism - cytochrome P450	2/807	36/5862	0.967750542	0.99997156	14872/11761	2
mmu04216	Ferroptosis	2/807	37/5862	0.97155476	0.99997156	15368/26570	2
mmu00190	Oxidative phosphorylation	10/807	118/5862	0.972177702	0.99997156	68198/66043/66445/227197/66046/68197/66694/68342/333182/67942	10
mmu04146	Peroxisome	5/807	70/5862	0.972638755	0.99997156	18126/26458/58869/26378/74043	5
mmu04917	Prolactin signaling pathway	4/807	60/5862	0.973615582	0.99997156	19116/216148/21934/19094	4
mmu05030	Cocaine addiction	2/807	38/5862	0.974924313	0.99997156	12915/231991	2
mmu05231	Choline metabolism in cancer	7/807	91/5862	0.975318135	0.99997156	20519/13139/18590/13645/100434/236899/331374	7
mmu04722	Neurotrophin signaling pathway	9/807	111/5862	0.976943404	0.99997156	216148/170936/18037/22034/18205/19094/16367/65020/20661	9
mmu05014	Amyotrophic lateral sclerosis	34/807	329/5862	0.977191297	0.99997156	68198/66043/20511/641376/545486/22123/110084/18126/66413/67089/66445/225027/227197/16574/66679/66046/18125/68197/26441/269113/18391/17184/56087/66694/234865/68342/15382/333182/19094/237806/229279/67942/22144/16572	34
mmu04932	Non-alcoholic fatty liver disease	12/807	140/5862	0.978766859	0.99997156	68198/21428/66445/227197/66046/108079/68197/66694/68342/333182/19094/16367	12
mmu00052	Galactose metabolism	1/807	26/5862	0.978928134	0.99997156	232714	1
mmu00260	Glycine, serine and threonine metabolism	1/807	26/5862	0.978928134	0.99997156	192166	1
mmu04912	GnRH signaling pathway	5/807	73/5862	0.97962192	0.99997156	432530/12292/11513/12288/19094	5

mmu05212	Pancreatic cancer	5/807	73/5862	0.97962192	0.99997156	107986/12567/12575/22339/13645	5
mmu05200	Pathways in cancer	46/807	431/5862	0.980351415	0.99997156	21414/16400/12447/107986/20423/15368/16590/12567/14365/17295/22413/20183/22411/11799/22027/23805/432530/18126/16195/14180/22408/12575/22339/18590/16782/23928/22034/12156/13645/18715/16186/12826/11513/23871/14697/66066/14872/16399/18854/16002/209590/14371/14184/11682/21415/12829	46
mmu00270	Cysteine and methionine metabolism	2/807	40/5862	0.980544242	0.99997156	20810/12035	2
mmu05204	Chemical car–inogenesis - DNA adducts	1/807	27/5862	0.981841944	0.99997156	14872	1
mmu05417	Lipid and atherosclerosis	15/807	170/5862	0.982528951	0.99997156	54131/20183/21939/22027/22359/17087/22034/230459/18127/66824/21947/106759/19094/15511/193740	15
mmu05010	Alzheimer disease	34/807	336/5862	0.984139258	0.99997156	68198/66043/14365/22413/22411/545486/22123/23805/18126/66413/67089/66445/22408/15108/227197/12292/16574/66046/18125/68197/26441/11785/66694/68342/12671/14371/12288/333182/68318/16367/67942/72027/22144/16572	34
mmu00630	Glyoxylate and dicarboxylate metabolism	1/807	28/5862	0.984353264	0.99997156	68738	1
mmu05016	Huntington disease	27/807	277/5862	0.984437629	0.99997156	68198/66043/21780/20511/21374/545486/22123/110084/245841/66413/67089/66445/14802/227197/16574/66046/68197/26441/56087/66694/68342/333182/231991/237806/67942/22144/16572	27
mmu01524	Platinum drug resistance	4/807	65/5862	0.984460016	0.99997156	11979/11799/12575/14872	4
mmu05160	Hepatitis C	10/807	126/5862	0.985292237	0.99997156	54131/12567/22629/16341/12575/72930/22034/13645/15945/106759	10
mmu01200	Carbon metabolism	7/807	97/5862	0.985613181	0.99997156	68263/13885/107029/170718/68738/225913/328099	7
mmu04625	C-type lectin receptor signaling pathway	6/807	87/5862	0.985791761	0.99997156	56760/66824/170786/239114/19094/66922	6
mmu05208	Chemical car–inogenesis - reactive oxygen species	17/807	191/5862	0.985812101	0.99997156	68198/66043/15368/17295/66445/22339/227197/66046/13645/68197/14872/66694/68342/231583/333182/19094/67942	17
mmu00310	Lysine degradation	3/807	56/5862	0.9879968	0.99997156	18822/70673/239122	3
mmu05012	Parkinson disease	22/807	238/5862	0.988056468	0.99997156	68198/66043/545486/22123/78294/66413/67089/66445/67847/227197/16574/66046/68197/26441/66694/68342/333182/67942/72027/22144/18951/16572	22
mmu04218	Cellular senescence	12/807	148/5862	0.988498626	0.99997156	12447/12567/63873/14991/12192/14463/19355/12575/50883/23871/19094/66922	12
mmu04070	Phosphatidylinositol signaling system	6/807	90/5862	0.989379278	0.99997156	84095/13139/240752/74596/18705/331374	6
mmu04668	TNF signaling pathway	6/807	90/5862	0.989379278	0.99997156	12915/69601/15945/67384/231991/19094	6
mmu04012	ErbB signaling pathway	5/807	80/5862	0.989973519	0.99997156	216148/12575/13645/18481/241656	5
mmu04142	Lysosome	9/807	121/5862	0.990086411	0.99997156	11433/11781/56722/13041/229445/56629/13032/74105/20661	9
mmu00520	Amino sugar and nucleotide sugar metabolism	2/807	46/5862	0.991014753	0.99997156	14584/54342	2
mmu00010	Glycolysis / Gluconeogenesis	2/807	47/5862	0.992111888	0.99997156	68263/68738	2

mmu04150	mTOR signaling pathway	10/807	135/5862	0.993098722	0.99997156	14365/22413/22411/22408/56716/20104/108079/230784/14371/16367	10
mmu00980	Metabolism of xenobiotics by cytochrome P450	1/807	34/5862	0.993598244	0.99997156	14872	1
mmu04973	Carbohydrate digestion and absorption	1/807	34/5862	0.993598244	0.99997156	232714	1
mmu05161	Hepatitis B	10/807	136/5862	0.993670399	0.99997156	12447/107986/54131/12915/11799/12575/22034/106759/231991/19094	10
mmu05220	Chronic myeloid leukemia	4/807	74/5862	0.994222763	0.99997156	107986/12567/216148/12575	4
mmu05022	Pathways of neurod– generation - multiple diseases	41/807	415/5862	0.994592881	0.99997156	68198/66043/641376/14365/22413/22411/545486/22123/110084/23805/78294/18126/66413/67089/66445/22408/67847/15108/14802/227197/12292/16574/66046/22065/18125/68197/26441/18391/56087/66694/68342/12671/14371/12288/333182/19094/237806/67942/22144/18951/16572	41
mmu00071	Fatty acid degradation	1/807	36/5862	0.995248524	0.99997156	328845	1
mmu04919	Thyroid hormone signaling pathway	7/807	109/5862	0.995393234	0.99997156	20183/14463/67381/53901/17888/140781/329650	7
mmu05163	Human cytomegalovirus infection	16/807	198/5862	0.995618638	0.99997156	54131/12567/12915/14991/432530/12575/22339/72512/12765/11513/14681/12927/14697/66066/231991/19094	16
mmu05167	Kaposi sarcoma-associated herpesvirus infection	13/807	170/5862	0.995778924	0.99997156	21414/54131/12567/14991/78294/16195/12575/22339/14697/66066/106759/19094/21415	13
mmu04962	Vasopressin-regulated water reabsorption	1/807	37/5862	0.995906684	0.99997156	231991	1
mmu04141	Protein processing in endoplasmic reticulum	11/807	155/5862	0.997140724	0.99997156	12915/216080/22027/103963/68292/105245/66435/66245/228684/15511/193740	11
mmu00562	Inositol phosphate metabolism	3/807	69/5862	0.99748835	0.99997156	84095/240752/18705	3
mmu04664	Fc epsilon RI signaling pathway	2/807	56/5862	0.997594837	0.99997156	14126/19094	2
mmu04922	Glucagon signaling pathway	4/807	82/5862	0.997681032	0.99997156	68263/108079/231991/15469	4
mmu04380	Osteoclast differentiation	7/807	117/5862	0.997924721	0.99997156	16818/11433/83433/21934/22034/19094/17060	7
mmu05170	Human immunodeficiency virus 1 infection	14/807	189/5862	0.998048374	0.99997156	12502/54131/12503/14991/12504/72512/22034/18481/14681/12500/14697/66066/241656/19094	14
mmu04152	AMPK signaling pathway	6/807	108/5862	0.998315219	0.99997156	72930/108079/20249/11549/231991/16367	6
mmu04910	Insulin signaling pathway	7/807	122/5862	0.998756401	0.99997156	216148/20104/108079/244416/228966/16367/105651	7
mmu04720	Long-term potentiation	2/807	61/5862	0.998769647	0.99997156	432530/12288	2
mmu0414–	Autophagy - animal	8/807	134/5862	0.998888787	0.99997156	56716/22034/108079/57436/73683/226421/66922/16367	8
mmu05017	Spinocerebellar ataxia	8/807	134/5862	0.998888787	0.99997156	21374/22123/66413/67089/22359/22065/114896/26441	8
mmu04621	NOD-like receptor signaling pathway	9/807	145/5862	0.99892283	0.99997156	54131/72512/22034/57436/66824/270151/216877/106759/19094	9
mmu04120	Ubiquitin mediated proteolysis	8/807	138/5862	0.999258386	0.99997156	107986/17222/216080/78294/28000/22034/18854/69957	8
mmu04666	Fc gamma R-mediated phagocytosis	3/807	89/5862	0.999799244	0.99997156	20259/74192/20698	3
mmu04217	Necroptosis	5/807	128/5862	0.999956446	0.99997156	11545/66824/106759/15267/319164	5

mmu04144	Endocytosis	15/807	246/5862	0.99997156	0.99997156	71889/14991/74002/12765/74192/16574/22034/16186/67804/18854/103967/14184/16572/15511/193740	15

Supplementary Table 5: Identified metabolites/ lipids list with their error in Da.

**(A) Metabolomics**					
**Experimental m/z**	**Ret. Time**	**names**	**Theoretical m/z**	**Delta**	**Ionization mode**
76.0386	7.04	Glycine	76.0393	-0.0007	Positive
90.055	6.92	L-Alanine	90.05495	5E-05	Positive
106.0498	7.41	L-Serine	106.04987	-7E-05	Positive
114.0653	3.16	Creatinine	114.06619	-0.00089	Positive
116.0703	6.87	L-Proline	116.07061	-0.00031	Positive
120.0653	7.12	L-Homoserine	120.06552	-0.00022	Positive
123.0547	1.66	Niacinamide	123.05529	-0.00059	Positive
130.0488	7.34	Pyrrolidonecarboxylic acid	130.04986	-0.00106	Positive
130.0854	8.19	Pipecolic acid	130.08626	-0.00086	Positive
132.0765	6.92	Creatine	132.07675	-0.00025	Positive
132.102	6.18	L-Norleucine	132.10191	9E-05	Positive
133.0601	7.48	L-Asparagine	133.06077	-0.00067	Positive
133.097	8.34	Ornithine	133.09715	-0.00015	Positive
137.0703	6.21	N-Methylnicotinamide	137.07094	-0.00064	Positive
147.0766	7.34	L-Glutamine	147.07641	0.00019	Positive
147.1121	8.15	L-Lysine	147.11281	-0.00071	Positive
148.0598	7.06	L-Glutamic acid	148.06044	-0.00064	Positive
150.0573	6.64	L-Methionine	150.05833	-0.00103	Positive
156.0763	8.15	L-Histidine	156.07675	-0.00045	Positive
162.1122	6.16	L-Carnitine	162.11247	-0.00027	Positive
166.0538	7.44	Methionine sulfoxide	166.05324	0.00056	Positive
166.0864	6.2	L-Phenylalanine	166.08626	0.00014	Positive
170.0928	7.9	1-Methylhistidine	170.09241	0.00039	Positive
175.1177	8.08	L-Arginine	175.11896	-0.00126	Positive
176.1014	7.49	Citrulline	176.10296	-0.00156	Positive
182.0814	6.74	L-Tyrosine	182.08117	0.00023	Positive
188.0702	6.18	Indoleacrylic acid	188.0706	-0.0004	Positive

204.1239	3.78	L-Acetylcarnitine	204.12303	0.00087	Positive
205.0969	6.2	L-Tryptophan	205.09715	-0.00025	Positive
258.1103	7.61	Glycerophosphocholine	258.11009	0.00021	Positive
261.0354	7.01	Glucose 1-phosphate	261.037	-0.0016	Positive
343.1233	6.98	Alpha-Lactose	343.12349	-0.00019	Positive
348.0715	8.56	Adenosine monophosphate	348.07036	0.00114	Positive
89.0234	1.49	D-Lactic acid	89.02442	-0.00102	Negative
104.0335	7.36	L-Serine	104.03532	-0.00182	Negative
124.0076	6.94	Taurine	124.0062	0.0014	Negative
129.0188	3.26	Citraconic acid_Mesaconic acid	129.01933	-0.00053	Negative
129.0542	1.36	Ketoleucine	129.05571	-0.00151	Negative
130.0873	6.18	L-Leucine	130.08736	-6E-05	Negative
131.0454	7.44	L-Asparagine	131.04622	-0.00082	Negative
145.0133	2.45	Oxoglutaric acid	145.01425	-0.00095	Negative
145.0609	7.28	L-Glutamine	145.06186	-0.00096	Negative
145.0969	8.08	L-Lysine	145.09825	-0.00135	Negative
147.0281	1.72	Ribonolactone	147.02989	-0.00179	Negative
154.0611	8.05	L-Histidine	154.0622	-0.0011	Negative
157.0369	6.11	Allantoin	157.03671	0.00019	Negative
164.0719	6.21	L-Phenylalanine	164.0717	0.0002	Negative
166.0149	1.36	Quinolinic acid	166.01457	0.00033	Negative
173.009	3.28	trans-Aconitic acid	173.00916	-0.00016	Negative
173.1042	7.96	L-Arginine	173.1044	-0.0002	Negative
174.0887	7.43	Citrulline	174.08841	0.00029	Negative
179.0571	6.98	D-Mannose	179.05611	0.00099	Negative
191.019	7.2	Citric acid	191.01973	-0.00073	Negative
195.0513	6.93	Gluconic acid	195.05103	0.00027	Negative
203.0824	6.18	L-Tryptophan	203.0826	-0.0002	Negative
346.0578	8.52	Adenosine monophosphate	346.0558	0.002	Negative
**(B) Lipidomics**					

Experimental m/z	Ret. Time	Name	Theoretical m/z by LIPID MAPS	Delta	Mode
1020.8003	11.73	TG 64:14	1020.8015	0.0012	Positive
973.7261	11.95	TG 62:15	973.728	0.0019	Positive
996.8016	12.54	TG 62:12	996.8015	0.0001	Positive
998.8178	12.66	TG 62:11	998.8171	0.0007	Positive
961.8227	14.36	TG 60:7	961.8219	0.0008	Positive
968.7684	11.95	TG 60:12	968.7702	0.0018	Positive
953.7598	12.95	TG 60:11	953.7593	0.0005	Positive
970.7849	12.35	TG 60:11	970.7858	0.0009	Positive
972.8033	12.56	TG 60:10	972.8015	0.0018	Positive
929.7577	13.21	TG 58:9	929.7593	0.0016	Positive
948.8004	12.97	TG 58:8	948.8015	0.0011	Positive
950.8161	13.3	TG 58:7	950.8171	0.001	Positive
937.8238	14.83	TG 58:5	937.8219	0.0019	Positive
958.8806	14.87	TG 58:3	958.8797	0.0009	Positive
942.7555	11.78	TG 58:11	942.7545	0.001	Positive
944.7718	12.18	TG 58:10	944.7702	0.0016	Positive
932.7713	13.65	TG 57:9	932.7702	0.0011	Positive
942.849	14.16	TG 57:4	942.8484	0.0006	Positive
922.7843	12.82	TG 56:7	922.7858	0.0015	Positive
924.802	13.26	TG 56:6	924.8015	0.0005	Positive
930.8479	14.41	TG 56:3	930.8484	0.0005	Positive
916.7408	11.62	TG 56:10	916.7389	0.0019	Positive
891.742	13.3	TG 55:7	891.7436	0.0016	Positive
881.7606	13.01	TG 54:5	881.7593	0.0013	Positive
900.7998	13.5	TG 54:4	900.8015	0.0017	Positive
863.7137	12.75	TG 53:7	863.7123	0.0014	Positive
867.7444	13.72	TG 53:5	867.7436	0.0008	Positive
884.771	12.8	TG 53:5	884.7702	0.0008	Positive
886.7845	13.28	TG 53:4	886.7858	0.0013	Positive

890.8159	14.19	TG 53:2	890.8171	0.0012	Positive
866.7237	11.9	TG 52:7	866.7232	0.0005	Positive
868.7378	12.19	TG 52:6	868.7389	0.0011	Positive
870.7543	12.56	TG 52:5	870.7545	0.0002	Positive
872.7102	11.14	TG 52:4	872.7103	0.0001	Positive
857.7577	13.52	TG 52:3	857.7593	0.0016	Positive
876.8014	13.97	TG 52:2	876.8015	0.0001	Positive
878.8189	14.46	TG 52:1	878.8171	0.0018	Positive
856.7146	10.89	TG 51:5	856.7154	0.0008	Positive
860.7683	13.26	TG 51:3	860.7702	0.0019	Positive
862.7854	13.69	TG 51:2	862.7858	0.0004	Positive
846.7526	13	TG 50:3	846.7545	0.0019	Positive
850.7849	14	TG 50:1	850.7858	0.0009	Positive
832.7405	12.73	TG 49:3	832.7389	0.0016	Positive
838.7875	14.26	TG 49:0	838.7858	0.0017	Positive
816.7068	11.98	TG 48:4	816.7076	0.0008	Positive
740.6749	12.45	TG 42:0	740.6763	0.0014	Positive
712.6444	11.92	TG 40:0	712.645	0.0006	Positive
684.6143	11.31	TG 38:0	684.6137	0.0006	Positive
489.3567	3.49	ST 30:3;O5	489.3574	0.0007	Positive
492.4058	7.41	ST 30:2;O4	492.4047	0.0011	Positive
429.3721	7.45	ST 29:2;O2	429.3727	0.0006	Positive
592.4577	7.56	ST 29:2;O;Hex	592.4572	0.0005	Positive
457.2956	2.59	ST 28:5;O5	457.2948	0.0008	Positive
512.3358	3.55	ST 28:2;O7	512.3582	0.0008	Positive
568.4587	7.65	ST 27:0;O;Hex	568.4572	0.0015	Positive
460.2684	2.31	ST 26:6;O6	460.2694	0.001	Positive
369.2405	9.22	ST 24:5;O3	369.2424	0.0019	Positive
369.2412	13.6	ST 24:5;O3	369.2424	0.0012	Positive
371.2574	8.08	ST 24:4;O3	371.2581	0.0007	Positive
389.2681	8.06	ST 24:3;O4	389.2686	0.0005	Positive

391.2831	6.23	ST 24:2;O4	391.2843	0.0012	Positive
408.3091	5.5	ST 24:2;O4	408.3108	0.0017	Positive
426.32	10.07	ST 24:1;O5	426.3214	0.0014	Positive
363.2183	4.29	ST 21:3;O5	363.2166	0.0017	Positive
347.2209	1.67	ST 21:3;O4	347.2217	0.0008	Positive
302.2474	4.92	ST 20:3;O	302.2478	0.0004	Positive
338.232	4.37	ST 19:2;O4	338.2326	0.0006	Positive
798.5392	8.8	SQDG 30:0	798.5396	0.0004	Positive
288.2895	2.75	SPB 17:0;O2	288.2897	0.0002	Positive
274.2736	2.27	SPB 16:0;O2	274.274	0.0004	Positive
829.7157	10.88	SM 43:1;O2	829.7157	0	Positive
811.6683	9.5	SM 42:3;2	811.6687	0.0004	Positive
813.6846	10.04	SM 42:2;2	813.6844	0.0002	Positive
815.6986	10.68	SM 40:3;4	815.7	0.0014	Positive
799.6673	9.75	SM 40:3;3	799.6687	0.0014	Positive
785.6542	9.54	SM 40:2;2	785.6531	0.0011	Positive
804.6948	10.42	SM 40:1;O2	804.6953	0.0005	Positive
757.6236	9.64	SM 38:2;O2	757.6218	0.0018	Positive
727.5736	7.59	SM 36:3;O2	727.5748	0.0012	Positive
729.5909	8.14	SM 36:2;2	729.5905	0.0004	Positive
717.5906	14.89	SM 35:1;O2	717.5905	0.0001	Positive
701.5577	7.45	SM 34:2;2	701.5592	0.0015	Positive
703.5751	8.07	SM 34:1;2	703.5748	0.0003	Positive
705.5896	8.33	SM 34:0;2	705.5905	0.0009	Positive
687.5454	13.95	SM 33:2;O2	687.5435	0.0019	Positive
689.5603	7.72	SM 33:1;O2	689.5592	0.0011	Positive
852.5863	9.53	SHexCer 38:1;O3	852.5865	0.0002	Positive
824.5563	8.89	SHexCer 36:1;O3	824.5552	0.0011	Positive
854.6275	8.67	PS O-42:4_PG O-42:6	854.6269	0.0006	Positive
856.6428	9.3	PS O-42:3_PG O-42:5	856.6426	0.0002	Positive
828.6113	8.67	PS O-40:3_PG O-40:5	828.6113	0	Positive

800.5812	9.21	PS O-38:3_PG O-38:5	800.58	0.0012	Positive
802.5972	7.94	PS O-38:2_PG O-38:4	802.5956	0.0016	Positive
772.5503	7.19	PS O-36:3_PG O-36:5	772.5487	0.0016	Positive
730.5003	8.24	PS O-33:3	730.5017	0.0014	Positive
734.5332	12.82	PS O-33:1_PG O-33:3	734.533	0.0002	Positive
721.5109	9.01	PS O-31:2	721.5126	0.0017	Positive
707.4979	9.36	PS O-30:2	707.497	0.0009	Positive
709.5116	14.07	PS O-30:1	709.5126	0.001	Positive
911.6493	11.66	PS 44:5	911.6484	0.0009	Positive
915.6801	12.15	PS 44:3	915.6797	0.0004	Positive
899.6487	11.6	PS 43:4	899.6484	0.0003	Positive
903.6811	12.31	PS 43:2	903.6797	0.0014	Positive
907.711	13.26	PS 43:0	907.711	0	Positive
891.6811	12.69	PS 42:1	891.6797	0.0014	Positive
879.68	12.78	PS 41:0	879.6797	0.0003	Positive
848.636	10.23	PS 40:0_PG 40:2	848.6375	0.0015	Positive
796.5107	7.67	PS 37:5_PG 37:7	796.5123	0.0016	Positive
804.5734	8.74	PS 37:1_PG 37:3	804.5749	0.0015	Positive
805.5692	8.87	PS 36:2	805.5702	0.001	Positive
789.5404	8.15	PS 35:3	789.5389	0.0015	Positive
695.3528	9.76	PS 25:4;O3	695.3515	0.0013	Positive
808.6229	10.02	plasmenyl-PE 42:4	808.6215	0.0014	Positive
748.5282	8.37	plasmenyl-PE 38:6	748.5276	0.0006	Positive
758.6064	10.18	plasmenyl-PE 38:1	758.6058	0.0006	Positive
724.49	9.67	plasmenyl-PE 36:4	724.4912	0.0012	Positive
724.5275	8.6	plasmenyl-PE 36:4	724.5276	0.0001	Positive
730.5362	7.52	plasmenyl-PE 36:1	730.5381	0.0019	Positive
730.5748	9.56	plasmenyl-PE 36:1	730.5745	0.0003	Positive
702.5418	8.9	plasmenyl-PE 34:1	702.5432	0.0014	Positive
847.6768	12.05	PG O-42:1	847.6786	0.0018	Positive
807.5521	7.09	PG O-40:7	807.5534	0.0013	Positive

733.5384	13.97	PG O-34:2	733.5378	0.0006	Positive
831.6104	9.64	PG 40:2	831.611	0.0006	Positive
850.6533	10.87	PG 40:1	850.6532	0.0001	Positive
821.6279	8.94	PG 39:0	821.6266	0.0013	Positive
822.6204	10.29	PG 38:1	822.6219	0.0015	Positive
723.5164	13.33	PG 32:0	723.5171	0.0007	Positive
698.497	7.38	PG 29:0	698.4967	0.0003	Positive
670.4644	7.37	PG 27:0	670.4654	0.001	Positive
642.4337	6.73	PG 25:0	642.4341	0.0004	Positive
754.5742	9.4	PE O-38:4	754.5745	0.0003	Positive
717.5539	7.23	PE O-34:3	717.5541	0.0002	Positive
691.5376	7.14	PE O-32:2	691.5385	0.0009	Positive
790.5726	8.12	PE 40:7	790.5745	0.0019	Positive
762.5988	9.38	PE 38:7	762.6007	0.0019	Positive
768.554	8.94	PE 38:4	768.5538	0.0002	Positive
772.584	8.53	PE 38:2	772.5851	0.0011	Positive
772.6211	9.73	PE 38:2	772.6215	0.0004	Positive
774.5451	8.39	PE 38:1	774.5432	0.0019	Positive
754.5385	8.58	PE 37:4	754.5381	0.0004	Positive
742.5372	7.47	PE 36:3	742.5381	0.0009	Positive
744.5546	7.85	PE 36:2	744.5538	0.0008	Positive
744.5891	8.62	PE 36:2	744.5902	0.0011	Positive
746.5696	8.4	PE 36:1_PC 33:1_PA 38:2	746.5694	0.0002	Positive
716.5224	8.42	PE 34:2	716.5225	0.0001	Positive
718.5753	9.07	PE 34:1	718.5745	0.0008	Positive
686.4742	8.27	PE 32:3	686.4755	0.0013	Positive
664.4907	8.22	PE 30:0	664.4912	0.0005	Positive
820.6221	8.92	PC O-40:6	820.6215	0.0006	Positive
826.6698	9.88	PC O-40:3	826.6684	0.0014	Positive
794.6063	8.49	PC O-38:5	794.6058	0.0005	Positive
798.6369	9.82	PC O-38:3	798.6371	0.0002	Positive

782.6064	9.8	PC O-37:4_PE O-40:4	782.6058	0.0006	Positive
719.5705	7.84	PC O-31:2	719.5698	0.0007	Positive
723.6016	14.06	PC O-31:0_PE O-34:0	723.6011	0.0005	Positive
705.5551	7.5	PC O-30:2	705.5541	0.001	Positive
693.5538	13.13	PC O-29:1_PE O-32:1	693.5541	0.0003	Positive
878.5703	7.59	PC 44:12	878.5694	0.0009	Positive
905.7662	12.84	PC 43:0	905.7681	0.0019	Positive
856.5845	7.62	PC 42:9	856.5851	0.0006	Positive
858.6007	8.97	PC 42:8	858.6007	0	Positive
860.6151	9.18	PC 42:7	860.6164	0.0013	Positive
864.6459	9.37	PC 42:5	864.6477	0.0018	Positive
870.6947	10.68	PC 42:2	870.6946	0.0001	Positive
854.5676	8.92	PC 42:10	854.5694	0.0018	Positive
850.6675	9.78	PC 41:5	850.6684	0.0009	Positive
854.5692	7.19	PC 41:3	854.5694	0.0002	Positive
830.5679	7.35	PC 40:8	830.5694	0.0015	Positive
832.5851	8.68	PC 40:7	832.5851	0	Positive
834.5998	9.04	PC 40:6	834.6007	0.0009	Positive
833.5815	8.52	PC 39:8_PE 42:8	833.5803	0.0012	Positive
818.5701	7.56	PC 39:7	818.5694	0.0007	Positive
822.6012	9.21	PC 39:5_PE 42:5	822.6007	0.0005	Positive
824.6537	9.8	PC 39:4	824.6528	0.0009	Positive
826.6319	9.36	PC 39:3	826.632	0.0001	Positive
830.6635	10.26	PC 39:1	830.6633	0.0002	Positive
804.5541	7.24	PC 38:7	804.5538	0.0003	Positive
806.5703	7.81	PC 38:6	806.5694	0.0009	Positive
808.5866	8.08	PC 38:5	808.5851	0.0015	Positive
810.6005	8.72	PC 38:4	810.6007	0.0002	Positive
812.6153	9.03	PC 38:3	812.6164	0.0011	Positive
818.6045	8.83	PC 38:0	818.6058	0.0013	Positive
790.5363	8.94	PC 37:7	790.5381	0.0018	Positive

792.5888	7.95	PC 37:6	792.5902	0.0014	Positive
811.5971	9.42	PC 37:5_PE 40:5	811.598	0.0011	Positive
796.62	9.16	PC 37:4	796.6215	0.0015	Positive
802.631	9.73	PC 37:1_PE 40:1	802.632	0.001	Positive
829.57	8.72	PC 36:5;O2_PS 38:4	829.5702	0.0002	Positive
782.5698	8.04	PC 36:4	782.5694	0.0004	Positive
786.5059	8.03	PC 36:2	786.5068	0.0009	Positive
786.5995	8.87	PC 36:2	786.6007	0.0012	Positive
788.6174	9.4	PC 36:1	788.6164	0.001	Positive
790.6325	10.01	PC 36:0	790.632	0.0005	Positive
766.5375	7.85	PC 35:5	766.5381	0.0006	Positive
766.5739	8.36	PC 35:5	766.5745	0.0006	Positive
768.5538	7.71	PC 35:4	768.5538	0	Positive
787.5959	8.34	PC 35:3	787.596	0.0001	Positive
774.601	9.08	PC 35:1	774.6007	0.0003	Positive
776.6535	10.41	PC 35:0	776.6528	0.0007	Positive
754.5371	8.1	PC 34:4	754.5381	0.001	Positive
758.5694	8.19	PC 34:2	758.5694	0	Positive
760.585	8.76	PC 34:1	760.5851	0.0001	Positive
748.5831	9.03	PC 33:0	748.5851	0.002	Positive
704.4482	8.28	PC 30:1	704.4497	0.0015	Positive
679.4306	9.69	PC 25:3;O2_PE 28:3;O2	679.4293	0.0013	Positive
666.4352	4.84	PC 25:1;O2_PS 27:0	666.4341	0.0011	Positive
627.3999	9.67	PC 21:1;O2	627.398	0.0019	Positive
319.284	4.86	MG O-15:0;O	319.2843	0.0003	Positive
336.3102	4.86	MG O-15:0;O	336.3108	0.0006	Positive
376.343	5.46	MG 18:0_MG O-18:1;O	376.3421	0.0009	Positive
331.2851	5.48	MG 16:0_MG O-16:1;O	331.2843	0.0008	Positive
526.294	3.84	LPE 22:6	526.2928	0.0012	Positive
478.2942	4.1	LPE 18:2	478.2928	0.0014	Positive
606.4497	6.59	LPC 24:1	606.4493	0.0004	Positive

608.4631	7.3	LPC 24:0	608.465	0.0019	Positive
594.4482	6.95	LPC 23:0	594.4493	0.0011	Positive
568.3396	3.73	LPC 22:6	568.3398	0.0003	Positive
572.3716	4.57	LPC 22:4	572.3711	0.0005	Positive
578.4172	5.92	LPC 22:1	578.418	0.0008	Positive
580.4323	6.61	LPC 22:0	580.4337	0.0014	Positive
542.3234	3.99	LPC 20:5	542.3241	0.0007	Positive
544.3384	3.91	LPC 20:4	544.3398	0.0014	Positive
546.3544	4.35	LPC 20:3	546.3554	0.001	Positive
548.3716	4.74	LPC 20:2	548.3711	0.0005	Positive
550.3856	5.23	LPC 20:1	550.3867	0.0011	Positive
552.4018	5.89	LPC 20:0	552.4024	0.0006	Positive
538.3861	5.53	LPC 19:0	538.3867	0.0006	Positive
518.3235	3.29	LPC 18:3	518.3241	0.0006	Positive
520.3395	3.99	LPC 18:2	520.3398	0.0003	Positive
522.3546	4.6	LPC 18:1	522.3554	0.0008	Positive
524.3705	5.18	LPC 18:0	524.3711	0.0006	Positive
508.3379	4.24	LPC 17:1	508.3398	0.0019	Positive
510.3544	4.82	LPC 17:0	510.3554	0.001	Positive
494.3237	3.56	LPC 16:1	494.3241	0.0004	Positive
496.3399	4.45	LPC 16:0	496.3398	0.0001	Positive
482.3234	5.28	LPC 15:0	482.3241	0.0007	Positive
482.359	4.76	LPC 15:0	482.3605	0.0015	Positive
468.3089	3.17	LPC 14:0	468.3085	0.0004	Positive
454.2923	2.6	LPC 13:0	454.2928	0.0005	Positive
683.4973	7.94	LPS O-28:1;O_PS O-28:0	683.497	0.0003	Positive
655.4662	9.07	LPS O-26:1;O	655.4657	0.0005	Positive
583.3195	9.82	LPS O-22:0;O_LPG 22:1	584.3922	0.0012	Positive
540.3656	4.67	LPS O-20:0	540.366	0.0004	Positive
512.334	1.7	LPS O-18:0	512.3347	0.0007	Positive
503.3089	2.31	LPS O-15:0;O	503.3092	0.0003	Positive

537.2931	4.47	LPS 18:3	537.2935	0.0004	Positive
515.3086	4.49	LPS 16:0_LPS O-16:1;O	515.3092	0.0006	Positive
555.3638	9.05	LPG 21:0	555.3656	0.0018	Positive
488.2991	2.31	LPG 15:0	488.2983	0.0008	Positive
460.2659	4.14	LPG 13:0	460.267	0.0011	Positive
460.2677	13.84	LPG 13:0	460.267	0.0007	Positive
446.2528	14.76	LPG 12:0	446.2513	0.0015	Positive
564.4009	5.54	LPE 24:1	564.4024	0.0015	Positive
504.3093	5.26	LPE 20:3	504.3085	0.0008	Positive
536.4088	5.56	LPC O-20:1	536.4074	0.0014	Positive
508.3748	4.88	LPC O-18:1	508.3761	0.0013	Positive
413.2782	8.87	LPC O-10:1	413.2775	0.0007	Positive
812.697	10.87	HexCer 42:1;2	812.6974	0.0004	Positive
798.6835	10.59	HexCer 41:1;O2	798.6817	0.0018	Positive
784.6671	10.3	HexCer 40:1;2	784.6661	0.001	Positive
731.5776	9.57	HexCer 34:2;O3	731.578	0.0004	Positive
701.5655	14.16	HexCer 33:2;O2	701.5674	0.0019	Positive
888.6414	9.12	Hex2Cer 36:2;O2	888.6407	0.0007	Positive
892.6708	9.95	Hex2Cer 36:0;O2	892.672	0.0012	Positive
551.5029	13.52	DG O-32:2	551.5034	0.0005	Positive
738.6048	12.21	DG 44:8	738.6031	0.0017	Positive
742.6326	12.38	DG 44:6	742.6344	0.0018	Positive
714.6037	12.46	DG 42:6	714.6031	0.0006	Positive
687.4975	10.31	DG 42:11	687.4983	0.0008	Positive
686.5699	9.38	DG 40:6	686.5718	0.0019	Positive
696.6514	9.47	DG 40:1	696.65	0.0014	Positive
639.4966	9.07	DG 38:7	639.4983	0.0017	Positive
660.556	9.43	DG 38:5	660.5561	0.0001	Positive
664.5874	9.62	DG 38:3	664.5874	0	Positive
634.5396	9.06	DG 36:4	634.5405	0.0009	Positive
636.5556	9.61	DG 36:3	636.5561	0.0005	Positive

640.5872	10.71	DG 36:1	640.5874	0.0002	Positive
625.5754	8.01	DG 36:0	625.5765	0.0011	Positive
591.4964	10.08	DG 34:3	591.4983	0.0019	Positive
610.5392	9.61	DG 34:2	610.5405	0.0013	Positive
597.5468	10.71	DG 34:0	597.5452	0.0016	Positive
569.513	10.09	DG 32:0	569.5139	0.0009	Positive
925.52	7.96	CL 36:4	925.5202	0.0002	Positive
506.3615	4.35	CerP 26:1;O2_LPC O-18:2	506.3605	0.001	Positive
506.3615	5.17	CerP 26:1;O2_LPC O-18:2	506.3605	0.001	Positive
632.634	11.52	Cer 42:2;O	632.634	0	Positive
622.6114	10.91	Cer 40:1;O2	622.6133	0.0019	Positive
642.603	11.27	Cer 39:0;O4_DG 36:0	642.6031	0.0001	Positive
584.5611	11.59	Cer 36:0;O3	584.5612	0.0001	Positive
568.566	9.67	Cer 36:0;O2	568.5663	0.0003	Positive
540.5334	8.87	Cer 34:0;O2	540.535	0.0016	Positive
714.6179	13.27	CE(22:6)	714.6183	0.0004	Positive
694.6485	14.36	CE(20:2)	694.6496	0.0011	Positive
664.6017	13.46	CE(18:3)	664.6027	0.001	Positive
666.6185	13.99	CE(18:2)	666.6183	0.0002	Positive
668.633	14.5	CE(18:1)	668.634	0.001	Positive
668.6556	11.19	CE(18:1)	668.6551	0.0005	Positive
699.606	14.36	CE 22:5	699.6075	0.0015	Positive
675.6088	13.91	CE 20:3	675.6075	0.0014	Positive
696.6648	14.51	CE 20:1	696.6653	0.0005	Positive
647.5769	14.58	CE 18:3	647.5762	0.0008	Positive
649.5905	13.96	CE 18:2	649.5918	0.0013	Positive
642.6179	14.58	CE 16:0	642.6183	0.0004	Positive
628.6028	10.31	CE 15:0	628.6027	0.0001	Positive
302.1961	1.22	CAR 8:1;O	302.1962	0.0001	Positive
305.2439	13.61	CAR 8:0	305.2435	0.0004	Positive
279.1567	4.03	CAR 4:1;O2	279.1551	0.0016	Positive

448.3422	4.18	CAR 20:4_ST 27:3;O4_ST 28:2;O4	448.3421	0.0001	Positive
204.1218	1.2	CAR 2:0	204.123	0.0012	Positive
458.3467	4.51	CAR 18:1;O2	458.3476	0.0009	Positive
426.3596	4.66	CAR 18:1	426.3578	0.0018	Positive
431.3855	5.49	CAR 17:0	431.3843	0.0012	Positive
396.3091	3.16	CAR 16:2_ST 23:1;O4	396.3108	0.0017	Positive
447.3431	7.47	CAR 16:1;O2	447.3429	0.0002	Positive
400.3411	4.55	CAR 16:0	400.3421	0.001	Positive
370.294	2.85	CAR 14:1	370.2952	0.0012	Positive
191.0197	1.14	FA 6:2;O5	191.0197	0	Negative
119.0338	1.2	FA 4:0;O2	119.035	0.0012	Negative
113.0238	1.21	FA 5:2;O	113.0244	0.0006	Negative
161.0442	1.21	FA 6:1;O3_FA 5:1;O	161.0456	0.0014	Negative
179.0552	1.22	FA 6:0;O4_FA 5:0;O2	179.0561	0.0009	Negative
225.0604	1.22	FA 6:0;O4	225.0616	0.0012	Negative
205.0521	1.24	FA 11:6;O2_FA 10:6	205.0506	0.0015	Negative
203.035	1.27	FA 10:7	203.035	0	Negative
103.0403	1.29	FA 4:0;O	103.0401	0.0002	Negative
391.2119	1.68	FA 22:6;O4	391.2126	0.0007	Negative
420.2508	2.15	CerP 20:1;O2	420.2521	0.0013	Negative
293.1759	2.34	FA 16:4	293.1758	0.0001	Negative
239.1276	2.45	FA 12:3	239.1289	0.0013	Negative
619.2898	2.76	LPI 20:4	619.2889	0.0009	Negative
512.2993	3.2	LPS O-17:0;O_LPC 14:0_LPE 17:0_LPE O-17:1;O_PC O-14:0	512.2994	0.0001	Negative
562.3168	3.29	LPC 18:3	562.3151	0.0017	Negative
538.3156	3.57	LPS 19:0_LPS O-19:1;O_LPC 16:1_LPE 19:1	538.3151	0.0005	Negative
612.3302	3.77	LPC 22:6	612.3307	0.0005	Negative
524.2781	3.89	LPE 22:6	524.2783	0.0002	Negative
588.3314	3.93	LPC 20:4	588.3307	0.0007	Negative
528.3108	3.93	LPE 22:4	528.3096	0.0012	Negative
526.3141	4	LPC 15:0_LPE 18:0_LPE O-18:1;O	526.3151	0.001	Negative

564.3289	4.02	LPC 18:2	564.3307	0.0018	Negative
500.2784	4.03	LPE 20:4	500.2783	0.0001	Negative
476.2798	4.13	LPE 18:2	476.2783	0.0015	Negative
552.3296	4.25	LPS 20:0_LPC 17:1_LPE 20:1	552.3307	0.0011	Negative
464.2788	4.29	LPC 14:1_LPE 17:1	464.2783	0.0005	Negative
526.294	4.35	LPE 22:5	526.2939	0.0001	Negative
590.3456	4.36	LPC 20:3	590.3464	0.0008	Negative
614.3455	4.38	LPC 22:5	614.3464	0.0009	Negative
540.3304	4.48	LPS 20:6	540.3307	0.0003	Negative
616.3617	4.48	LPC 22:4	616.362	0.0003	Negative
480.3087	4.49	LPE 18:0	480.3096	0.0009	Negative
625.2995	4.51	PA 24:2;O3	625.2995	0	Negative
566.3478	4.62	PS 20:0	566.3464	0.0014	Negative
584.3587	4.67	LPS O-20:0	584.3569	0.0018	Negative
478.2936	4.71	LPE 18:1	478.2939	0.0003	Negative
592.3601	4.76	LPC 20:2_PC O-20:2	592.362	0.0019	Negative
526.3509	4.77	LPC O-16:0	526.3514	0.0005	Negative
436.285	4.81	LPE O-16:1	436.2834	0.0016	Negative
554.3456	4.84	LPS O-20:0;O_LPC 17:0_LPE 20:0_PC O-17:0	554.3464	0.0008	Negative
552.3667	4.88	LPC O-18:1	552.3671	0.0004	Negative
466.2941	4.92	LPE 17:0	466.2939	0.0002	Negative
305.198	4.96	FA 14:0;O2	305.197	0.001	Negative
399.2749	4.99	FA 21:2;O2	399.2752	0.0003	Negative
594.3778	5.24	LPC 20:1_PC O-20:1	594.3777	0.0001	Negative
277.2182	5.26	FA 18:3	277.2173	0.0009	Negative
548.2986	5.28	LPS 20:2_LPE 20:3	548.2994	0.0008	Negative
327.2327	5.33	FA 22:6	327.233	0.0003	Negative
395.2204	5.35	LPA 15:0	395.2204	0	Negative
333.2291	5.47	FA 16:0;O2	333.2283	0.0008	Negative
379.2321	5.47	FA 18:0;O6	379.2338	0.0017	Negative
375.277	5.48	MG 16:0_MG O-16:1;O	375.2752	0.0018	Negative

303.2341	5.51	FA 20:4	303.233	0.0011	Negative
253.2159	5.51	FA 16:1	253.2173	0.0014	Negative
582.377	5.54	LPS O-22:0;O_LPC 19:0_LPE 22:0_PC O-19:0	582.3777	0.0007	Negative
279.2336	5.66	FA 18:2	279.233	0.0006	Negative
325.2387	5.69	FA 19:2;O2_FA 18:2	325.2384	0.0003	Negative
409.1862	5.69	FA 20:6;O4	409.1868	0.0006	Negative
393.2087	5.69	FA 24:10	393.2071	0.0016	Negative
329.2493	5.79	FA 22:5	329.2486	0.0007	Negative
465.3059	5.79	ST 27:1;O;S	465.3044	0.0015	Negative
549.2687	5.79	ST 28:5;O8	549.2705	0.0018	Negative
305.2494	5.86	FA 20:3	305.2486	0.0008	Negative
596.3942	5.89	LPS O-23:0;O	596.3933	0.0009	Negative
622.4099	5.89	LPS O-25:1;O_LPC 22:1	622.409	0.0009	Negative
331.2646	6.08	FA 22:4	331.2643	0.0003	Negative
301.2377	6.18	FA 17:0;O2_FA 16:0	301.2384	0.0007	Negative
255.232	6.18	FA 16:0	255.233	0.001	Negative
511.4749	6.18	FA 31:0	511.4732	0.0017	Negative
449.3107	6.19	FA 22:0;O4	449.312	0.0013	Negative
493.3315	6.22	LPA 22:0	493.33	0.0015	Negative
281.2481	6.25	FA 18:1	281.2486	0.0005	Negative
327.2532	6.25	FA 19:1;O2_FA 18:1	327.2541	0.0009	Negative
582.4123	6.27	LPC O-20:0	582.414	0.0017	Negative
494.3634	6.42	LPE 19:0	494.3616	0.0018	Negative
239.0568	6.88	FA 10:5;O2	239.0561	0.0007	Negative
283.2646	6.96	FA 18:0	283.2643	0.0003	Negative
619.29	7.01	LPI 20:4_PA 25:4;O2	619.2889	0.0011	Negative
881.5185	7.15	PI 38:6	881.5186	0.0001	Negative
822.5307	7.22	PC 36:6	822.5291	0.0016	Negative
652.456	7.32	LPC 24:0_LPE O-27:1;O	652.4559	0.0001	Negative
951.5236	7.38	PI 40:8	951.524	0.0004	Negative
719.5344	7.39	SM 32:1;O2	719.5345	0.0001	Negative

798.5279	7.39	PC 34:4	798.5291	0.0012	Negative
883.5336	7.4	PI 38:5	883.5342	0.0006	Negative
818.5543	7.45	PS O-36:2	818.5553	0.001	Negative
760.4911	7.47	PE 38:7	760.4923	0.0012	Negative
740.5275	7.59	PE 36:3	740.5236	0.0027	Negative
868.5334	7.63	PS 39:5	868.5345	0.0011	Negative
871.5335	7.66	PI 37:4	871.5342	0.0007	Negative
765.5806	7.71	PA 41:4	765.5804	0.0002	Negative
337.3127	7.72	FA 22:1	337.3112	0.0015	Negative
733.5518	7.73	SM 33:1;O2	733.5501	0.0017	Negative
847.535	7.79	PI 35:2	847.5342	0.0008	Negative
712.4934	7.82	PE 34:3	712.4923	0.0011	Negative
763.5525	7.85	DG 44:9	763.5518	0.0007	Negative
876.5775	7.87	PC 40:7	876.576	0.0015	Negative
969.513	7.99	CL 36:4	969.5111	0.0019	Negative
788.5217	8.07	PE 40:7	788.5236	0.0019	Negative
627.5001	8.08	DG 37:5	627.4994	0.0007	Negative
747.5647	8.09	SM 34:1;O2	747.5658	0.0011	Negative
750.5648	8.1	PC O-31:0_PE O-34:0	750.5655	0.0007	Negative
773.5825	8.14	SM 36:2;O2	773.5814	0.0011	Negative
742.5386	8.19	PE 36:2	742.5392	0.0006	Negative
768.5555	8.24	768.5555_8.24	768.5549	0.0006	Negative
662.475	8.24	PE 30:0	662.4766	0.0016	Negative
730.4664	8.28	PS 32:2	730.4665	0.0001	Negative
887.5671	8.29	PI 38:3	887.5655	0.0016	Negative
773.5338	8.4	PG 36:2_PA 38:2	773.5338	0	Negative
782.4981	8.42	PS 36:4_PC 33:5_PE 36:5_PE O-40:2	782.4978	0.0003	Negative
913.5818	8.45	PI 40:4	913.5812	0.0006	Negative
868.5697	8.51	PS O-40:5	868.5709	0.0012	Negative
748.5287	8.52	plasmenyl-PE 38:5	748.5287	0	Negative
774.5442	8.52	plasmenyl-PE 40:6	774.5443	0.0001	Negative

377.2707	8.52	FA 23:4;O2_FA 22:4	377.2697	0.001	Negative
595.3868	8.52	ST 27:1;O4;Hex	595.3852	0.0016	Negative
553.3389	8.54	ST 29:2;O7	553.3382	0.0007	Negative
756.556	8.54	PE 37:2	756.5549	0.0011	Negative
806.545	8.55	SHexCer 36:1;O2	806.5458	0.0008	Negative
518.3501	8.55	CAR 22:5	518.3487	0.0014	Negative
884.5674	8.55	PS 40:4	884.5658	0.0016	Negative
808.5489	8.57	PC O-36:6	808.5498	0.0009	Negative
722.5124	8.6	plasmenyl-PE 36:4	722.513	0.0006	Negative
790.5405	8.69	PE 40:6	790.5392	0.0013	Negative
778.5616	8.71	PC 32:0	778.5604	0.0012	Negative
854.5925	8.72	PC 38:4	854.5917	0.0008	Negative
718.5383	8.74	PE 34:0	718.5392	0.0009	Negative
744.5646	8.74	HexCer 34:1;O2	744.5631	0.0015	Negative
698.5121	8.8	plasmenyl-PE 34:2	698.513	0.0009	Negative
775.5952	8.8	SM 36:1;O2	775.5971	0.0019	Negative
700.5289	8.89	plasmenyl-PE 34:1	700.5287	0.0002	Negative
864.6138	8.95	PC O-40:6	864.6124	0.0014	Negative
796.5868	9.04	PE 40:3	796.5862	0.0006	Negative
762.5663	9.06	PS O-35:0_PC O-32:1_PE O-35:1	762.5655	0.0008	Negative
936.5969	9.1	PS 44:6	936.5971	0.0002	Negative
744.5563	9.12	PE 36:1	744.5549	0.0014	Negative
776.5603	9.18	plasmenyl-PE 40:5	776.56	0.0003	Negative
768.5544	9.26	PE 38:3	768.5549	0.0005	Negative
874.5827	9.38	PS 39:2	874.5815	0.0012	Negative
786.6024	9.41	PE 39:1	786.6018	0.0006	Negative
900.5989	9.42	PS 41:3	900.5971	0.0018	Negative
824.5817	9.43	PS O-40:4_PE O-40:5	824.5811	0.0006	Negative
756.562	9.44	HexCer 35:2;O2	756.5631	0.0011	Negative
829.643	9.45	PE 42:0	829.644	0.001	Negative
819.6132	9.47	PG 39:0_PA 41:0	819.6121	0.0011	Negative

702.5454	9.48	PE O-34:1	702.5443	0.0011	Negative
855.6616	9.5	SM 42:3;O2	855.6597	0.0019	Negative
939.6176	9.54	PI 38:0	939.6179	0.0003	Negative
858.6248	9.55	PC 38:2	858.623	0.0018	Negative
770.5707	9.62	PE 38:2	770.5705	0.0002	Negative
866.6294	9.69	PC O-40:5	866.6281	0.0013	Negative
802.5756	9.71	plasmenyl-PE 42:6	802.5756	0	Negative
846.6236	9.72	PC 37:1	846.623	0.0006	Negative
900.5749	9.75	PC 42:9	900.576	0.0011	Negative
788.6165	9.94	PE 39:0	788.6175	0.001	Negative
609.5603	9.96	CE 15:0	609.5616	0.0013	Negative
834.6226	10	PC 36:0	834.623	0.0004	Negative
862.6547	10.01	PC 38:0	862.6543	0.0004	Negative
730.5759	10.06	PE O-36:1	730.5756	0.0003	Negative
857.6767	10.06	SM 42:2;O2	857.6753	0.0014	Negative
847.6421	10.09	PG 41:0_PA 43:0	847.6434	0.0013	Negative
886.6556	10.12	PC 40:2	886.6543	0.0013	Negative
844.644	10.23	PS 40:1	844.6437	0.0003	Negative
808.622	10.44	PE O-42:4	808.6226	0.0006	Negative
859.6927	10.71	SM 42:1;O2	859.691	0.0017	Negative
806.593	10.88	PC 34:0	806.5917	0.0013	Negative
712.6463	11.19	Cer 42:0;O3	712.6461	0.0002	Negative
711.6286	11.51	CE 19:0	711.6297	0.0011	Negative

Supplementary Table 6: List of significantly changed metabolites and lipids in LD group and HD group using univariate analysis (two tailed student's t test).

	C vs. LD					C vs. HD				
**Identified significant features**	**t.stat**	**p.value**	**FDR**	**Fold Change**	**log2(FC)**	**t.stat**	**p.value**	**FDR**	**Fold Change**	**log2(FC)**
L-Asparagine	-2.6619	0.037431	0.26154	1.3515	0.43453	-3.4943	0.012915	0.22285	1.462	0.54798
L-Histidine	-10.14	0.000053505	0.045827	1.5587	0.64036	-7.4977	0.00029105	0.076702	1.6839	0.75178
L-Phenylalanine	-4.2451	0.0054094	0.15872	1.5203	0.60432	-2.9414	0.0259	0.2762	1.3179	0.39822
L-Proline	-3.1704	0.01931	0.21814	1.3829	0.4677	-2.6654	0.037254	0.33151	1.5501	0.63237
L-Serine	-3.5615	0.011906	0.19588	1.2807	0.35695	-4.0359	0.0068343	0.18298	1.4257	0.51167
Methionine sulfoxide	-3.198	0.018647	0.21676	2.2306	1.1574	-6.1731	0.00083041	0.097188	2.1805	1.1246
Alpha-Lactose	-3.2662	0.017115	0.214	1.7259	0.78733	–	–	–	–	–
L-Leucine	-2.5503	0.043476	0.24713	1.5974	0.67568	–	–	–	–	–
L-Methionine	-3.6806	0.010324	0.19045	1.2126	0.27807	–	–	–	–	–
L-Norleucine	-5.53	0.0014731	0.13066	1.4505	0.53653	–	–	–	–	–
L-Tryptophan	-2.4527	0.049609	0.29153	1.414	0.49974	–	–	–	–	–
Maltotriose	-3.3699	0.015045	0.2071	1.9492	0.96287	–	–	–	–	–
Ribonolactone	-3.7378	0.0096479	0.15658	1.7224	0.78444	–	–	–	–	–
1-Methylhistidine	–	–	–	–	–	-2.831	0.02992	0.29545	1.2772	0.35302
Creatine	–	–	–	–	–	-4.4707	0.0042343	0.16814	1.4608	0.54676
D-Lactic acid	–	–	–	–	–	-2.4651	0.048783	0.36355	1.5143	0.59866
Glycerophosphocholine	–	–	–	–	–	-2.7987	0.031219	0.29869	1.2919	0.36953
L-Arginine	–	–	–	–	–	-5.0437	0.0023482	0.14084	1.369	0.45311
L-Homoserine	–	–	–	–	–	-2.6041	0.040438	0.34123	1.455	0.54106
L-Lysine	–	–	–	–	–	-3.9778	0.0073016	0.18412	1.5075	0.59212
Ornithine	–	–	–	–	–	-3.9324	0.0076918	0.18412	1.6627	0.73356
Pipecolic acid	–	–	–	–	–	-3.8534	0.0084264	0.18993	1.4876	0.57301
PC 34:4	3.055	0.022	0.436	0.462	-1.114	5.864	0.001	0.046	0.295	-1.762
PS 43:4	–	–	–	–	–	9.415	0	0.094	0.299	-1.742
CAR 16:2_ST 23:1;O4	4.172	0.006	0.259	0.348	-1.523	4.357	0.005	0.17	0.342	-1.549
PE 38:7	3.524	0.012	0.436	0.556	-0.846	3.736	0.01	0.119	0.343	-1.544

DG 37:5	–	–	–	–	–	6.897	0	0.04	0.404	-1.307
PC 36:6	2.65	0.038	0.495	0.607	-0.72	4.699	0.003	0.063	0.413	-1.277
CAR 14:1	3.393	0.015	0.307	0.616	-0.698	7.328	0	0.109	0.432	-1.212
CAR 18:1	2.911	0.027	0.335	0.624	-0.681	5.29	0.002	0.135	0.432	-1.211
ST 24:3;O4	–	–	–	–	–	4.071	0.007	0.181	0.459	-1.124
PE 40:7	2.703	0.035	0.495	0.622	-0.685	4.906	0.003	0.062	0.472	-1.083
TG 52:7	–	–	–	–	–	3.257	0.017	0.278	0.5	-0.999
PS 44:5	–	–	–	–	–	2.556	0.043	0.421	0.533	-0.908
PC 32:1	3.306	0.016	0.307	0.719	-0.475	5.601	0.001	0.131	0.539	-0.892
PE 38:5	3.693	0.01	0.413	0.717	-0.479	3.847	0.008	0.107	0.559	-0.84
plasmenyl-PE 36:1	–	–	–	–	–	3.304	0.016	0.27	0.582	-0.78
PS O-36:3_PG O-36:5	–	–	–	–	–	3.596	0.011	0.222	0.627	-0.672
PS 37:5_PG 37:7	–	–	–	–	–	2.807	0.031	0.356	0.631	-0.665
FA 16:1	–	–	–	–	–	5.133	0.002	0.057	0.634	-0.658
MADAG 50:8+NH4	–	–	–	–	–	4.08	0.007	0.181	0.639	-0.646
PS 34:0	–	–	–	–	–	4.23	0.006	0.082	0.656	-0.607
PE 36:3	–	–	–	–	–	2.622	0.039	0.407	0.665	-0.588
PC O-30:2	3.302	0.016	0.307	0.715	-0.484	3.995	0.007	0.188	0.667	-0.584
LPC 24:0_LPE O-27:1;O	–	–	–	–	–	2.564	0.043	0.317	0.685	-0.547
SM 43:1;O2	–	–	–	–	–	5.565	0.001	0.131	0.686	-0.543
PE 38:3	–	–	–	–	–	2.625	0.039	0.31	0.687	-0.541
PC 42:9	2.451	0.05	0.367	0.699	-0.517	2.857	0.029	0.35	0.688	-0.54
PE 38:4	4.003	0.007	0.341	0.782	-0.354	7.076	0	0.038	0.689	-0.538
PC 41:3	4.159	0.006	0.259	0.652	-0.616	6.103	0.001	0.113	0.692	-0.531
PC 35:4	–	–	–	–	–	5.449	0.002	0.132	0.693	-0.53
PE O-32:2	3.385	0.015	0.307	0.62	-0.689	4.605	0.004	0.161	0.694	-0.527
PE 40:6	–	–	–	–	–	3.585	0.012	0.137	0.695	-0.526
PA 41:4	2.505	0.046	0.496	0.776	-0.366	4.548	0.004	0.069	0.707	-0.5
PS 39:5	–	–	–	–	–	6.06	0.001	0.044	0.714	-0.486
PE 36:1_PC 33:1_PA 38:2	–	–	–	–	–	3.602	0.011	0.222	0.72	-0.474
CE(18:3)	3.106	0.021	0.328	0.667	-0.584	2.761	0.033	0.358	0.731	-0.451

CE 20:3	3.073	0.022	0.328	0.693	-0.53	3.734	0.01	0.203	0.742	-0.431
CAR 16:0	4.713	0.003	0.216	0.598	-0.741	2.945	0.026	0.333	0.749	-0.416
SM 40:3;3	–	–	–	–	–	4.662	0.003	0.161	0.75	-0.416
PE 37:4	2.451	0.05	0.367	0.876	-0.191	6.16	0.001	0.113	0.76	-0.396
PE 38:6	2.948	0.026	0.333	0.783	-0.352	2.809	0.031	0.356	0.761	-0.394
ST 29:2;O7	–	–	–	–	–	6.658	0.001	0.044	0.764	-0.388
PC 36:2	3.588	0.012	0.284	0.753	-0.409	3.154	0.02	0.285	0.767	-0.383
CAR 20:4_ST 27:3;O4_ST 28:2;O4	–	–	–	–	–	2.495	0.047	0.426	0.768	-0.382
PC 38:7	–	–	–	–	–	3.397	0.015	0.256	0.777	-0.365
PC 40:8	4.623	0.004	0.216	0.738	-0.439	4.696	0.003	0.161	0.777	-0.364
CAR 22:5	–	–	–	–	–	3.571	0.012	0.138	0.782	-0.355
PE 40:4	–	–	–	–	–	3.032	0.023	0.317	0.79	-0.34
PC O-29:1_PE O-32:1	3.793	0.009	0.283	0.554	-0.851	4.937	0.003	0.155	0.791	-0.338
PG O-40:7	–	–	–	–	–	2.491	0.047	0.426	0.8	-0.322
PC 37:7	8.304	0	0.075	0.883	-0.179	2.512	0.046	0.426	0.801	-0.32
PC 36:4	4.402	0.005	0.235	0.85	-0.235	6.485	0.001	0.113	0.802	-0.318
PC O-31:0_PE O-34:0	2.839	0.03	0.335	0.725	-0.465	2.601	0.041	0.315	0.803	-0.317
LPC 20:4	4.725	0.003	0.293	0.766	-0.385	4.129	0.006	0.086	0.81	-0.304
PC 36:3	–	–	–	–	–	4.487	0.004	0.069	0.814	-0.296
PC 38:5	–	–	–	–	–	3.457	0.014	0.246	0.818	-0.29
PC 35:5.1	–	–	–	–	–	2.5	0.047	0.426	0.844	-0.245
CE 18:3	–	–	–	–	–	2.755	0.033	0.358	0.85	-0.234
lysoPE 22:4	3.211	0.018	0.436	0.802	-0.318	2.482	0.048	0.333	0.856	-0.225
FA 23:4;O2_FA 22:4	–	–	–	–	–	3.296	0.016	0.179	0.867	-0.206
PE 40:7	3.282	0.017	0.307	0.912	-0.133	5.29	0.002	0.135	0.872	-0.198
TG 38:0	3.352	0.015	0.307	0.862	-0.215	-2.511	0.046	0.426	1.098	0.134
PC O-31:2	–	–	–	–	–	-2.483	0.048	0.426	1.1	0.138
SM 34:1;2	–	–	–	–	–	-3.183	0.019	0.284	1.12	0.164
PC O-38:5	–	–	–	–	–	-3.902	0.008	0.198	1.155	0.208
LPG 21:0	3.042	0.023	0.328	0.859	-0.219	-2.85	0.029	0.35	1.175	0.233

lysoPC 20:2	–	–	–	–	–	-2.621	0.04	0.407	1.179	0.238
DG 42:11	-2.592	0.041	0.367	1.158	0.211	-5.71	0.001	0.126	1.18	0.239
PG 39:0	–	–	–	–	–	-4.609	0.004	0.161	1.193	0.255
plasmenyl-PE 42:4	-3.285	0.017	0.307	1.21	0.275	-3.253	0.017	0.278	1.231	0.3
PC O-40:6	-2.491	0.047	0.367	1.114	0.155	-3.729	0.01	0.203	1.237	0.307
DG 36:1	–	–	–	–	–	-2.469	0.049	0.426	1.253	0.325
MG O-15:0;O.1	–	–	–	–	–	-2.553	0.043	0.421	1.256	0.329
LPC 15:0_LPE 18:0_LPE O-18:1;O	–	–	–	–	–	-2.686	0.036	0.303	1.288	0.365
PC O-38:3	–	–	–	–	–	-2.77	0.032	0.358	1.334	0.415
PS O-42:3_PG O-42:5	–	–	–	–	–	-5.088	0.002	0.15	1.34	0.422
LPS O-20:0;O_LPC 17:0_LPE 20:0_PC O-17:0	–	–	–	–	–	-2.816	0.031	0.273	1.355	0.439
DG 38:7	–	–	–	–	–	-2.692	0.036	0.378	1.429	0.515
LPE 18:0	–	–	–	–	–	-2.557	0.043	0.317	1.443	0.529
CerP 26:1;O2_LPC O-18:2.1	–	–	–	–	–	-2.608	0.04	0.41	1.479	0.564
LPC 20:1_PC O-20:1	–	–	–	–	–	-2.589	0.041	0.315	1.53	0.613
LPE 18:2	–	–	–	–	–	-2.653	0.038	0.303	1.538	0.621
lysoPC 15:0.1	–	–	–	–	–	-6.341	0.001	0.113	1.556	0.638
FA 21:2;O2	-3.461	0.013	0.436	1.676	0.745	-4.366	0.005	0.077	1.638	0.712
LPC O-16:0	-3.352	0.015	0.436	1.342	0.425	-10.306	0	0.017	1.698	0.764
LPC O-20:1	-3.103	0.021	0.328	1.569	0.65	-4.169	0.006	0.181	1.728	0.789
lysoPE 19:0	-5.211	0.002	0.234	1.841	0.881	-6.245	0.001	0.044	1.775	0.828
PE O-42:4	-5.549	0.001	0.218	1.657	0.729	-5.789	0.001	0.046	1.895	0.922
Cer 36:0;O3	–	–	–	–	–	-2.552	0.043	0.421	2.138	1.096
LPC O-18:1	-4.145	0.006	0.259	1.457	0.543	-8.013	0	0.109	2.166	1.115
LPC O-18:1	-9.93	0	0.036	1.491	0.576	-21.362	0	0.001	2.199	1.137
LPC O-20:0	-3.171	0.019	0.436	1.802	0.849	-7.481	0	0.038	2.472	1.306
CerP 26:1;O2_LPC O-18:2	-3.264	0.017	0.307	1.693	0.76	-8.045	0	0.109	2.651	1.407
PC 30:0	3.383	0.015	0.307	0.802	-0.319	–	–	–	–	–
CAR 2:0	2.698	0.036	0.349	0.518	-0.948	–	–	–	–	–
CE 18:2	4.925	0.003	0.216	0.671	-0.576	–	–	–	–	–

CE(18:2)	6.126	0.001	0.143	0.655	-0.611	–	–	–	–	–
CE(20:2)	4.586	0.004	0.216	0.728	-0.457	–	–	–	–	–
DAG 40:4	3.828	0.009	0.283	0.825	-0.278	–	–	–	–	–
DG 36:3	-2.865	0.029	0.335	1.487	0.573	–	–	–	–	–
DG 38:3	-2.879	0.028	0.335	1.426	0.512	–	–	–	–	–
DG 44:6	2.499	0.047	0.367	0.549	-0.865	–	–	–	–	–
DG 44:9	4.338	0.005	0.323	0.754	-0.406	–	–	–	–	–
LPS O-22:0;O_LPC 19:0_LPE 22:0_PC O-19:0	-2.529	0.045	0.495	1.561	0.642	–	–	–	–	–
lysoPC 18:2	2.484	0.048	0.367	0.828	-0.272	–	–	–	–	–
lysoPC 20:5	2.481	0.048	0.367	0.839	-0.254	–	–	–	–	–
lysoPC 22:4	2.466	0.049	0.367	0.707	-0.5	–	–	–	–	–
lysoPC 22:6	2.782	0.032	0.343	0.759	-0.398	–	–	–	–	–
lysoPC 24:0	4.896	0.003	0.216	0.809	-0.306	–	–	–	–	–
MADAG 54:10+NH4	-2.903	0.027	0.335	1.564	0.645	–	–	–	–	–
MADAG 54:11+NH4	-2.699	0.036	0.349	1.492	0.578	–	–	–	–	–
MADAG 58:12+NH4	-2.511	0.046	0.367	1.38	0.464	–	–	–	–	–
PE 36:4	2.846	0.029	0.454	0.828	-0.272	–	–	–	–	–
PE 42:0	2.869	0.028	0.449	0.835	-0.26	–	–	–	–	–
PG 38:1	2.486	0.047	0.367	0.768	-0.38	–	–	–	–	–
PG 40:1	3.262	0.017	0.307	0.77	-0.378	–	–	–	–	–
PG O-34:2	4.212	0.006	0.255	0.707	-0.501	–	–	–	–	–
PS 41:0	-2.885	0.028	0.335	1.696	0.762	–	–	–	–	–
PS 42:1	-2.483	0.048	0.367	1.462	0.548	–	–	–	–	–
PS 44:6	-2.482	0.048	0.496	1.402	0.487	–	–	–	–	–
PS O-30:1	3.41	0.014	0.307	0.789	-0.342	–	–	–	–	–
PS O-33:1_PG O-33:3	3.151	0.02	0.328	0.655	-0.61	–	–	–	–	–
SM 33:2;O2	4.584	0.004	0.216	0.653	-0.615	–	–	–	–	–
SM 34:3;4_SM 36:1;2	3.288	0.017	0.307	0.754	-0.407	–	–	–	–	–
SM 36:1;O2	2.614	0.04	0.495	0.71	-0.495	–	–	–	–	–
SM 36:2;2	3.633	0.011	0.284	0.798	-0.326	–	–	–	–	–

SM 36:2;O2	3.241	0.018	0.436	0.669	-0.58	–	–	–	–	–
SM 36:3;4_SM 38:1;2	3.011	0.024	0.328	0.626	-0.676	–	–	–	–	–
SM 36:4;4_SM 38:2;2	2.932	0.026	0.333	0.762	-0.392	–	–	–	–	–
SM 40:3;4	4.644	0.004	0.216	0.665	-0.589	–	–	–	–	–
SM 42:1;O2	3.07	0.022	0.436	0.848	-0.238	–	–	–	–	–
TG 50:3	-2.938	0.026	0.333	1.663	0.734	–	–	–	–	–
TG 51:2	-3.12	0.021	0.328	1.965	0.975	–	–	–	–	–
TG 51:3	-3.127	0.02	0.328	1.99	0.993	–	–	–	–	–
TG 52:1	-2.505	0.046	0.367	1.511	0.595	–	–	–	–	–
TG 52:3	-3.632	0.011	0.284	1.543	0.626	–	–	–	–	–
TG 52:5	-2.543	0.044	0.367	1.87	0.903	–	–	–	–	–
TG 53:2	-3.069	0.022	0.328	1.72	0.782	–	–	–	–	–
TG 53:4	-2.824	0.03	0.336	1.776	0.829	–	–	–	–	–
TG 53:5	-3.063	0.022	0.328	1.746	0.804	–	–	–	–	–
TG 53:5.1	-2.643	0.038	0.36	1.792	0.841	–	–	–	–	–
TG 53:7	-2.554	0.043	0.367	1.962	0.972	–	–	–	–	–
TG 54:5	-2.51	0.046	0.367	1.528	0.611	–	–	–	–	–

Supplementary Table 7: Pathway analysis of dysregulated metabolites in LD group (A) and HD group (B) compared to Control group (p values shows pathway enrichment analysis based on Global Test and pathway impact values shows the pathway topology analysis).

(A) Pathway analysis of C vs LD (p-value<0.05, impact>0.02)								
	Total	Expected	Hits	Raw p	#NAME?	Holm adjust	FDR	Impact
Aminoacyl-tRNA biosynthesis	48	0.44622	8	1.52E-09	8.8173	1.28E-07	1.28E-07	0.16667
Cysteine and methionine metabolism	33	0.30677	2	0.035954	1.4443	1	1	0.1263
Phenylalanine, tyrosine and tryptophan biosynthesis	4	0.037185	1	0.036705	1.4353	1	1	0.5
Valine, leucine and isoleucine biosynthesis	8	0.074369	1	0.072156	1.1417	1	1	0
Phenylalanine metabolism	12	0.11155	1	0.10639	0.97309	1	1	0.35714
Glycerolipid metabolism	16	0.14874	1	0.13945	0.85557	1	1	0.01402
Histidine metabolism	16	0.14874	1	0.13945	0.85557	1	1	0.22131
beta-Alanine metabolism	21	0.19522	1	0.17918	0.74671	1	1	0
Sphingolipid metabolism	21	0.19522	1	0.17918	0.74671	1	1	0
Alanine, aspartate and glutamate metabolism	28	0.26029	1	0.23195	0.63461	1	1	0
Glyoxylate and dicarboxylate metabolism	32	0.29748	1	0.26066	0.58392	1	1	0.04233
Glycine, serine and threonine metabolism	34	0.31607	1	0.27465	0.56123	1	1	0.20661
Arginine and proline metabolism	38	0.35325	1	0.30188	0.52017	1	1	0.0778
Valine, leucine and isoleucine degradation	40	0.37185	1	0.31513	0.5015	1	1	0
Tryptophan metabolism	41	0.38114	1	0.32167	0.49258	1	1	0.14305

(B) Pathway analysis of C vs HD (p-value<0.05, impact>0.02)								
HD	Total	Expected	Hits	Raw p	#NAME?	Holm adjust	FDR	Impact
Aminoacyl-tRNA biosynthesis	48	0.47809	7	1.14E-07	6.9449	9.54E-06	9.54E-06	0.16667
Arginine and proline metabolism	38	0.37849	4	0.000386	3.4133	0.032048	0.016217	0.25841
Arginine biosynthesis	14	0.13944	2	0.007867	2.1042	0.64511	0.21536	0.13705
Histidine metabolism	16	0.15936	2	0.010255	1.989	0.83069	0.21536	0.22131
Lysine degradation	25	0.249	2	0.024345	1.6136	1	0.40899	0
Phenylalanine, tyrosine and tryptophan biosynthesis	4	0.039841	1	0.039288	1.4057	1	0.5188	0.5

Glycine, serine and threonine metabolism	34	0.33865	2	0.043233	1.3642	1	0.5188	0.20661
Biotin metabolism	10	0.099602	1	0.095527	1.0199	1	1	0
Phenylalanine metabolism	12	0.11952	1	0.11358	0.9447	1	1	0.35714
Ether lipid metabolism	20	0.1992	1	0.18248	0.73878	1	1	0
beta-Alanine metabolism	21	0.20916	1	0.19073	0.71957	1	1	0
Sphingolipid metabolism	21	0.20916	1	0.19073	0.71957	1	1	0
Pyruvate metabolism	22	0.21912	1	0.19891	0.70135	1	1	0.08398
Alanine, aspartate and glutamate metabolism	28	0.27888	1	0.24636	0.60843	1	1	0
Glutathione metabolism	28	0.27888	1	0.24636	0.60843	1	1	0
Glyoxylate and dicarboxylate metabolism	32	0.31873	1	0.27652	0.55827	1	1	0.04233
Cysteine and methionine metabolism	33	0.32869	1	0.28388	0.54686	1	1	0.02184
Glycerophospholipid metabolism	36	0.35857	1	0.30555	0.51491	1	1	0.04814

Supplementary Table 8: Differentially expressed genes common in both radiation groups.

(Two-tailed p-value calculation using Wald statistics and Log2 fold change by maximum-likelihood estimate).

		LD			HD		
**Gene name**	**Gene description**	**log2Fold Change**	**Upregulation or Downregulation**	**p-value (adj)**	**log2Fold Change**	**Upregulation or Downregulation**	**p-value (adj)**
**4930486L24Rik**	**RIKEN cDNA 4930486L24 gene**	2.179	2.179	0.00273	2.804	2.804	1.90757E-09
**Adgrf5**	**adhesion G protein-coupled receptor F5**	3.842	3.842	0.00509	2.783	2.783	5.04682E-05
**Armcx6**	**armadillo repeat containing, X-linked 6**	2.022	2.022	0.04792	2.681	2.681	0.001640411
**Bcas3os1**	**breast carcinoma amplified sequence 3, opposite strand 1**	2.275	2.275	0.03437	2.538	2.538	0.000230673
**Blk**	**B lymphoid kinase**	-2.307	-2.307	2.3E-07	-3.63	-3.63	9.10614E-19
**Blnk**	**B cell linker**	-2.044	-2.044	0.00016	-2.797	-2.797	3.39806E-11
**Ccr6**	**chemokine (C-C motif) receptor 6**	-2.49	-2.49	0.00081	-2.536	-2.536	4.74191E-05
**Cd200**	**CD200 antigen**	-2.153	-2.153	0.02696	-2.724	-2.724	0.000401773
**Cd74**	**CD74 antigen (invariant polypeptide of major histocompatibility complex, class II antigen-associated)**	-2.064	-2.064	0.00262	-5.317	-5.317	7.45352E-24
**Cd79a**	**CD79A antigen (immunoglobulin-associated alpha)**	-2.016	-2.016	0.00097	-6.132	-6.132	8.511E-20
**Cd79b**	**CD79B antigen**	-2.03	-2.03	0.0002	-7.173	-7.173	1.39832E-41
**Cdkn1a**	**cyclin-dependent kinase inhibitor 1A (P21)**	2.206	2.206	0.03427	2.482	2.482	0.000399111
**Chst3**	**carbohydrate sulfotransferase 3**	-2.387	-2.387	3E-06	-4.038	-4.038	5.64482E-20
**Ciita**	**class II transactivator**	-2.569	-2.569	2.3E-07	-4.601	-4.601	2.72629E-22
**Clca3a1**	**chloride channel accessory 3A1**	2.594	2.594	0.01346	2.761	2.761	0.001449224
**Dexi**	**dexamethasone-induced transcript**	-2.047	-2.047	0.00026	-3.229	-3.229	4.83481E-17
**F11r**	**F11 receptor**	2.015	2.015	0.00368	2.204	2.204	3.11409E-05
**Fcer2a**	**Fc receptor, IgE, low affinity II, alpha polypeptide**	-2.327	-2.327	0.00017	-5.381	-5.381	1.48172E-21

**Fcmr**	**Fc fragment of IgM receptor**	-2.185	-2.185	7.7E-05	-5.804	-5.804	2.07289E-25
**Fcrla**	**Fc receptor-like A**	-2.001	-2.001	1.3E-05	-4.782	-4.782	3.16726E-24
**Fpr2**	**formyl peptide receptor 2**	2.114	2.114	0.00171	2.766	2.766	4.21174E-07
**Gm15050**	**predicted gene 15050**	2.284	2.284	0.02651	3.275	3.275	4.68598E-06
**Gp6**	**glycoprotein 6 (platelet)**	2.185	2.185	0.00056	2.472	2.472	4.47584E-09
**H2-Ab1**	**histocompatibility 2, class II antigen A, beta 1**	-2.459	-2.459	0.00039	-5.95	-5.95	5.1713E-26
**H2-Eb2**	**histocompatibility 2, class II antigen E beta2**	-2.227	-2.227	0.00304	-5.934	-5.934	1.71447E-16
**H2-Ob**	**histocompatibility 2, O region beta locus**	-2.412	-2.412	3.2E-06	-5.902	-5.902	1.97027E-24
**Hist1h4m**	**histone cluster 1, H4m**	2.06	2.06	0.00187	2.602	2.602	1.40385E-06
**Hnmt**	**histamine N-methyltransferase**	2.358	2.358	0.02389	3.158	3.158	9.39632E-06
**Mest**	**mesoderm specific transcript**	2.208	2.208	0.00249	3.061	3.061	2.5645E-13
**Mfap3l**	**microfibrillar-associated protein 3-like**	2.694	2.694	2.9E-07	3.254	3.254	1.70134E-15
**Mir5107**	**microRNA 5107**	-2.317	-2.317	1.3E-05	-5.484	-5.484	1.31069E-31
**Nyap2**	**neuronal tyrosine-phophorylated phosphoinositide 3-kinase adaptor 2**	2.011	2.011	0.01978	2.339	2.339	8.0219E-05
**Pde5a**	**phosphodiesterase 5A, cGMP-specific**	2.038	2.038	0.00109	2.663	2.663	6.43504E-09
**Pdgfa**	**platelet derived growth factor, alpha**	2.211	2.211	7.9E-05	2.263	2.263	8.50722E-07
**Peg10**	**paternally expressed 10**	2.015	2.015	0.01812	2.381	2.381	1.73103E-06
**Plp1**	**proteolipid protein (myelin) 1**	2.279	2.279	0.00104	2.381	2.381	2.26711E-07
**Popdc2**	**popeye domain containing 2**	2.256	2.256	0.00183	2.649	2.649	1.40256E-08
**Ppm1f**	**protein phosphatase 1F (PP2C domain containing)**	-3.647	-3.647	0.00952	-4.163	-4.163	2.15394E-05
**Ptgs1**	**prostaglandin-endoperoxide synthase 1**	2.329	2.329	0.00047	2.879	2.879	1.15454E-09
**Rgs18**	**regulator of G-protein signaling 18**	2.352	2.352	0.00883	2.976	2.976	5.12078E-09
**Satb2**	**special AT-rich sequence binding protein 2**	2.42	2.42	0.04601	2.167	2.167	1.12811E-05
**Scd1**	**stearoyl-Coenzyme A desaturase 1**	-2.134	-2.134	1.3E-05	-4.841	-4.841	1.5353E-17
**Scn4a**	**sodium channel, voltage-gated, type IV, alpha**	-2.414	-2.414	0.00031	-2.786	-2.786	8.91112E-06
**Sdc4**	**syndecan 4**	-2.261	-2.261	0.00232	-2.55	-2.55	0.000188854
**Sh3bgrl2**	**SH3 domain binding glutamic acid-rich protein like 2**	2.045	2.045	0.00362	2.802	2.802	1.14879E-09

**Slamf1**	**signaling lymphocytic activation molecule family member 1**	2.043	2.043	0.01036	2.413	2.413	2.45212E-07
**Slfn4**	**schlafen 4**	2.467	2.467	0.0041	2.228	2.228	0.000741934
**Smim3**	**small integral membrane protein 3**	2.063	2.063	0.00235	2.806	2.806	2.81577E-08
**Srpk3**	**serine/arginine-rich protein specific kinase 3**	-2.313	-2.313	0.00253	-4.013	-4.013	1.48062E-07
**Sycp1**	**synaptonemal complex protein 1**	2.55	2.55	0.03417	2.476	2.476	0.002065869
**Tnfrsf13c**	**tumor necrosis factor receptor superfamily, member 13c**	-2.551	-2.551	2.3E-07	-3.923	-3.923	6.24418E-14
**Triqk**	**triple QxxK/R motif containing**	2.705	2.705	0.00688	2.75	2.75	5.21562E-06
**Trove2**	**TROVE domain family, member 2**	2.208	2.208	0.00322	2.306	2.306	4.85907E-07
**Ttc34**	**tetratricopeptide repeat domain 34**	4.286	4.286	0.00693	4.818	4.818	3.06796E-06
**Ttc9**	**tetratricopeptide repeat domain 9**	-3.605	-3.605	0.00342	-2.852	-2.852	0.001376546
**Vill**	**villin-like**	2.707	2.707	0.0302	3.012	3.012	0.006042759
**Vpreb3**	**pre-B lymphocyte gene 3**	-2.338	-2.338	0.00114	-3.512	-3.512	2.01472E-09
**Zfp772**	**zinc finger protein 772**	-4.146	-4.146	0.02329	-2.933	-2.933	0.01241694
